# *ZmCCA1a* on Chromosome 10 of Maize Delays Flowering of *Arabidopsis thaliana*

**DOI:** 10.3389/fpls.2020.00078

**Published:** 2020-02-20

**Authors:** Yong Shi, Xiyong Zhao, Sha Guo, Shifeng Dong, Yanpeng Wen, Zanping Han, Weihuan Jin, Yanhui Chen

**Affiliations:** ^1^College of Agronomy/National Key Laboratory of Wheat and Maize Crop Science, Henan Agricultural University, Zhengzhou, China; ^2^Crop Research Institute, Anhui Academy of Agricultural Sciences, Hefei, China; ^3^College of Agronomy, Henan University of Science and Technology, Luoyang, China; ^4^College of Life Sciences, Henan Agricultural University, Zhengzhou, China

**Keywords:** *maize*, *ZmCCA1*, overexpression, circadian rhythms, photoperiodic flowering

## Abstract

Maize (*Zea mays*) is a major cereal crop that originated at low latitudes, and thus photoperiod sensitivity is an important barrier to the use of tropical/subtropical germplasm in temperate regions. However, studies of the mechanisms underlying circadian regulation in maize are at an early stage. In this study we cloned *ZmCCA1a* on chromosome 10 of maize by map-based cloning. The gene is homologous to the Myb transcription factor genes *AtCCA1*/*AtLHY* in *Arabidopsis thaliana*; the deduced Myb domain of ZmCCA1a showed high similarity with that of AtCCA1/AtLHY and ZmCCA1b. Transiently or constitutively expressed ZmCCA1a-YFPs were localized to nuclei of *Arabidopsis* mesophyll protoplasts, agroinfiltrated tobacco leaves, and leaf and root cells of transgenic seedlings of *Arabidopsis thaliana*. Unlike AtCCA1/AtLHY, ZmCCA1a did not form homodimers nor interact with ZmCCA1b. Transcripts of *ZmCCA1a* showed circadian rhythm with peak expression around sunrise in maize inbred lines CML288 (photoperiod sensitive) and Huangzao 4 (HZ4; photoperiod insensitive). Under short days, transcription of *ZmCCA1a* in CML288 and HZ4 was repressed compared with that under long days, whereas the effect of photoperiod on *ZmCCA1a* expression was moderate in HZ4. In *ZmCCA1a-*overexpressing *A. thaliana* (*ZmCCA1a-ox*) lines, the circadian rhythm was disrupted under constant light and flowering was delayed under long days, but the hypocotyl length was not affected. In addition, expression of endogenous *AtCCA1/AtLHY* and the downstream genes *AtGI*, *AtCO*, and *AtFt* was repressed in *ZmCCA1a-ox* seedlings. The present results suggest that the function of *ZmCCA1a* is similar, at least in part, to that of *AtCCA1/AtLHY* and *ZmCCA1b*, implying that *ZmCCA1a* is likely to be an important component of the circadian clock pathway in maize.

## Introduction

The photoperiod and its annual changes with seasons provide exogenous signals that organisms use to predict upcoming environmental changes. All organisms, from microorganisms to humans (Aronson et al., 1994), have evolved ubiquitous circadian clock systems to respond to photoperiod information. In plants, the circadian clock adjusts physiological and biochemical responses at different developmental stages for prediction and adaptation to daily and seasonal changes in the environment (Suárez-López et al., 2001; Yanovsky and Kay, 2002; Más, 2004). An estimated 30%–50% of the transcriptome in *Arabidopsis* (*Arabidopsis thaliana*) rosettes grown under a 12 h/12 h (light/dark) cycle showed diurnal rhythmic expression (Bläsing et al., 2005).

In general, the circadian clock regulatory system comprises an input pathway, an oscillator, and an output pathway (Esther et al., 2009; Wang et al., 2011). Among these elements, the oscillator, which functions through multiple negative feedback loops, is the core component that maintains the circadian clock period of approximately 24 h. The rhythmicity and period of the oscillator can be entrained and reset by environmental signals received and transmitted by input pathway components. The oscillator then transmits these messages to downstream components of the output pathway.

The proteins AtLHY (LATE ELONGATED HYPOCOTYL), AtCCA1 (CIRCADIAN CLOCK-ASSOCIATED 1), and *Arabidopsis* pseudo-response regulators (PRRs), including PRR1 (TOC1; TIMING OF CAB 1), PRR3, PRR5, PRR7, and PRR9, are important members of the oscillator feedback loop and their transcripts show circadian oscillation. AtCCA1/AtLHY (AtCCA1 and AtLHY) are highly homologous Myb transcription factors with partially redundant functions, and their transcripts accumulate to peak levels at dawn. Through formation of the CDD (COP10–DET1–DDB1) complex, AtCCA1/AtLHY repress transcription of target genes by binding to the evening elements in the promoter region (Alabadí et al., 2001; Lau et al., 2011; Nagel et al., 2015; Kamioka et al., 2016).

Peak accumulation of *PRR9*, *PRR7*, *PRR5*, and *TOC1* transcripts is observed at discrete times in the sequence of morning (ZT2-6), midday (ZT6-13), afternoon (ZT13), and evening (Nakamichi et al., 2010; Fujiwara et al., 2008; Gendron et al., 2012; Huang and Mas, 2012; Wang et al., 2013). These PRRs, which may repress gene expression by forming a complex with TOPLESS/TOPLESS-RELATED (TPL/TPR) and histone deacetylase, repress transcription of *AtCCA1/AtLHY* in temporal order through the mid-morning to the evening through binding to the promoter (Wang et al., 2013). In addition, *TOC1* is activated by REV8 through binding to the promoter region of *TOC1* (Hsu et al., 2013; Rugnone et al., 2013; Xie et al., 2014).

The negative loops, based on *AtCCA1/AtLHY* and *PRR* genes feedback regulation, play essential roles in the circadian clock function of *Arabidopsis*. High and constant expression levels of *AtLHY* or *AtCCA1* in *Arabidopsis* disturb rhythmic expression of clock-regulated genes and repress transcription of endogenous *AtCCA1/AtLHY. Arabidopsis* plants overexpressing *AtLHY* or *AtCCA1* show an elongated hypocotyl, late flowering, and arrhythmic leaf movements (Wang et al., 1997; Schaffer et al., 1998; Alabadí et al., 2001; Mizoguchi et al., 2002; Kim et al., 2003; Thain et al., 2004). Repression or deletion of *AtLHY* and/or *AtCCA1* activity results in a shortened period of circadian rhythms. Single mutants of *AtLHY* or *AtCCA1* show identical flowering times under long days (LD) and an early-flowering phenotype under short days (SD) compared with wild-type plants (WT) (Mizoguchi et al., 2002). Double mutants of *AtCCA1/AtLHY* showed earlier flowering than the WT and the progenitor single mutants under both LD and SD, and could not sustain the oscillations under constant light (LL) and constant dark (DD) conditions (Alabadí et al., 2001; Mizoguchi et al., 2002; Shogo et al., 2007; Yusuke et al., 2007).

A type of basic helix-loop-helix family transcription factor, PIFs (PHYTOCHROME-INTERACTING FACTORs), is a major integrator of endogenous and exogenous signals in the regulation of plant growth and development. These proteins may bind to the promoters of *AtCCA1/AtLHY* through the G-box elements and the affinity is enhanced by addition of sucrose (Martinez-Garcia et al., 2000; Oh et al., 2012; Paik et al., 2017). Furthermore, AtCCA1/AtLHY physically interact with DET1 (Lau et al., 2011) and DET1 might interact with PIFs (Dong et al., 2014). Thus, AtCCA1/AtLHY, DET1 and PIFs have been proposed to form a transcription factor complex to regulate gene repression (Paik et al., 2017), by this mechanism *AtCCA1/AtLHY* may be involved in plant growth and development. Chromatin immunoprecipitation followed by deep sequencing revealed that AtCCA1 targets genes that are involved in a large number of biological processes and stress responses in addition to circadian clock regulation (Nagel et al., 2015).

In contrast to *Arabidopsis*, little is known about the circadian clock regulation system in maize (*Zea mays*). Relatively few maize genes have been identified or cloned, and for the majority of these genes the biological functions remain to be identified or require further study (Hayes et al., 2010). Although the wild ancestor of maize, teosinte, is a SD plant, maize inbred lines display a broad range of sensitivities to photoperiod (Russell and Stuber, 1983). Although sensitivity has been reduced with cultivar selection for LD in temperate areas, maize materials that originated in tropical and subtropical areas are usually sensitivity to day length (Stephenson et al., 2019). For example, delayed flowering and failure to flower are the predominant phenotypes for some tropical lines grown at high latitudes, which limit their adoption in temperate areas.

Many studies have mapped quantitative trait loci (QTLs) associated with the photoperiod response or flowering time in maize. A major QTL in bin 10.04 on chromosome (Chr) 10 has been identified consistently in maize in a variety of genetic backgrounds (Ducrocq et al., 2009; Wang et al., 2010). At least three candidate genes (*ZmCCT*, *PHYTOCHROME INTERACTING FACTOR3*, and *ZmCCA1a*) have been identified in this region (Yang et al., 2013; Ku et al., 2016). Our research is focused on the functional analysis of ZmCCA1a because it is a homolog of *AtCCA1/AtLHY*, which is a core component of the central oscillator in *Arabidopsis*. Given that two *CCA1* (or *LHY*) genes are located on Chr4 and Chr10 in the maize genome and the one-to-one homology with *AtCCA1/AtLHY* is ambiguous, these two maize *CCA1* (or *LHY*) genes were named arbitrarily (Hayes et al., 2010; Ko et al., 2016). To avoid confusion, the *CCA1* (or *LHY*) genes on Chr10 and Chr4 in maize were designated *ZmCCA1a* (Zm00001d024546) and *ZmCCA1b* (Zm00001d049543), respectively (Ko et al., 2016). Wang et al. (2011) identified a *ZmCCA1* gene through homology-based cloning to be a homolog of *AtCCA1*/*AtLHY* with a conserved function, which indicated that it might be a core component of the maize circadian clock pathway. However, sequence alignment implied that the gene cloned by Wang et al. (2011) was *ZmCCA1b*, not *ZmCCA1a* as asserted by the authors. In *ZmCCA1b*-overexpressing (*ZmCCA1b*-ox) *Arabidopsis* plants, circadian rhythmicity of *CAB2* expression was diminished under LL. However, *ZmCCA1a* overexpression in *Arabidopsis* had a lesser impact on circadian rhythmicity of *CAB2*. Noting that *ZmCCA1a* cloned by Ko et al. (2016) was truncated without the Myb domain, the function of the protein was probably compromised. In addition, *ZmCCA1a* and *ZmCCA1b* are diurnally upregulated in maize hybrids compared with expression patterns in the corresponding inbred parents, thus the two genes may be involved in the biomass heterosis of maize hybrids (Ko et al., 2016). In the present study, we cloned *ZmCCA1a* using a map-based cloning procedure. The expression pattern of *ZmCCA1a* transcripts showed a circadian rhythm in the maize inbred lines CML288 (photoperiod sensitive) and HZ4 (photoperiod insensitive). Overexpression of *ZmCCA1a* in *Arabidopsis* disrupted the circadian rhythm, delayed flowering, and affected expression of endogenous *AtCCA1/AtLHY* and related downstream genes. We speculate that *ZmCCA1a* acts as a core component of the circadian clock in maize.

## Results

### QTL Containing *ZmCCA1a* Gene was Fine-Mapped to a 2.6-Mb Region in Bin10.04 on Chr10 in Maize

**Figure 1:**
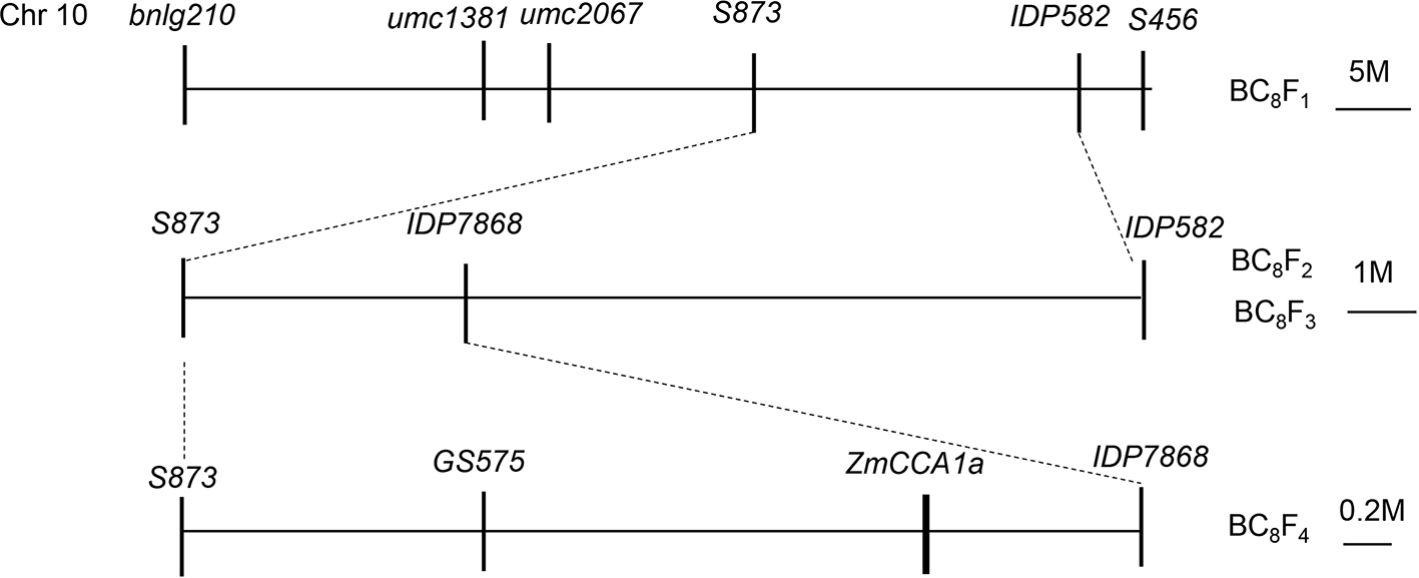
Sequential fine mapping of *ZmCCA1a* to a 2.6-Mb region on Chr10 of the maize BC_8_F_4_ mapping population. The *ZmCCA1a* locus was initially mapped between the markers S873 and IDP582 on Chr10, then fine mapped between the markers GS575 and IDP7868 with a physical distance of 2.6 Mb.

Major QTLs that affect flowering time have been mapped to bin10.04 on Chr10 in maize (Ducrocq et al., 2009; Wang et al., 2010). Recently, using progenies derived by backcrossing (with CML288 as the non-recurrent parent and HZ4 as the recurrent parent), we identified a QTL for flowering time in the same region on Chr10 and further divided it into three linked QTLs including the candidate genes *ZmCCA1a*, *PIF3*, and *ZmCCT*. The QTL containing *ZmCCA1a* was located between the molecular makers *S873* and *IDP582*. To further localize this QTL, we generated mapping populations derived from selfing of the BC_8_F_1_ population. The populations comprised BC_8_F_2_ with 1682 plants, BC_8_F_3_ with 112 plants, and BC_8_F_4_ with 1,350 plants. Screening with molecular markers (**Supplementary Table 1**) localized *ZmCCA1a* to a 2.6-Mb region on Chr10 between the markers *GS575* and *IDP7868* (**Figure 1**).

### ZmCCA1a, ZmCCA1b, and AtCCA1/AtLHY Show High Sequence Identity Within the Myb Domain

The bacterial artificial chromosome (BAC) clone of CML288 containing *ZmCCA1a* was screened and sequenced (**Supplementary Table 2**). This BAC sequence was aligned to the *ZmCCA1a* sequence from the maize “B73” reference genome and primers specific for *ZmCCA1a* were designed from the deduced *ZmCCA1a* sequence in CML288. The *ZmCCA1a* coding sequences (CDSs) in CML288 and HZ4 (**Supplementary Table 3**) were amplified by PCR using the designed primers. *ZmCCA1b* was cloned using a homology-based cloning procedure from the maize inbred lines CML288 and HZ4 (**Supplementary Table 3**). The deduced ZmCCA1a and ZmCCA1b amino acid sequences in CML288 and HZ4 showed 95.18% and 98.61% similarity, respectively, and 100% similarity within the Myb domain for both pairs of proteins (**Supplementary Figures 1** and **2**). Therefore, *ZmCCA1a* and *ZmCCA1b* from CML288 were chosen for further study.

The current annotation indicated that the *ZmCCA1* CDS encodes a protein of 718 amino acids. Alignment of ZmCCA1a with ZmCCA1b, AtCCA1, and AtLHY indicated that the proteins shared 87.24%, 32.87%, and 36.26% amino acid sequence identity. Each protein contained a single Myb domain (amino acids 22–75) that showed more than 94.34% similarity among the proteins. Similar to ZmCCA1b and AtCCA1/AtLHY, in ZmCCA1a, two of the three tryptophan residues conserved in the majority of Myb proteins were conserved; the third residue was replaced by alanine in all four proteins (**Supplementary Figure 3**). Wang et al. (2011) reported that ZmCCA1b was homologous to AtCCA1, and here we showed that ZmCCA1a was highly similar to ZmCCA1b and AtCCA1/AtLHY (**Supplementary Figures 3** and **4**).

### ZmCCA1a is Localized in the Nuclei of Plant Cells *In Vivo*

**Figure 2:**
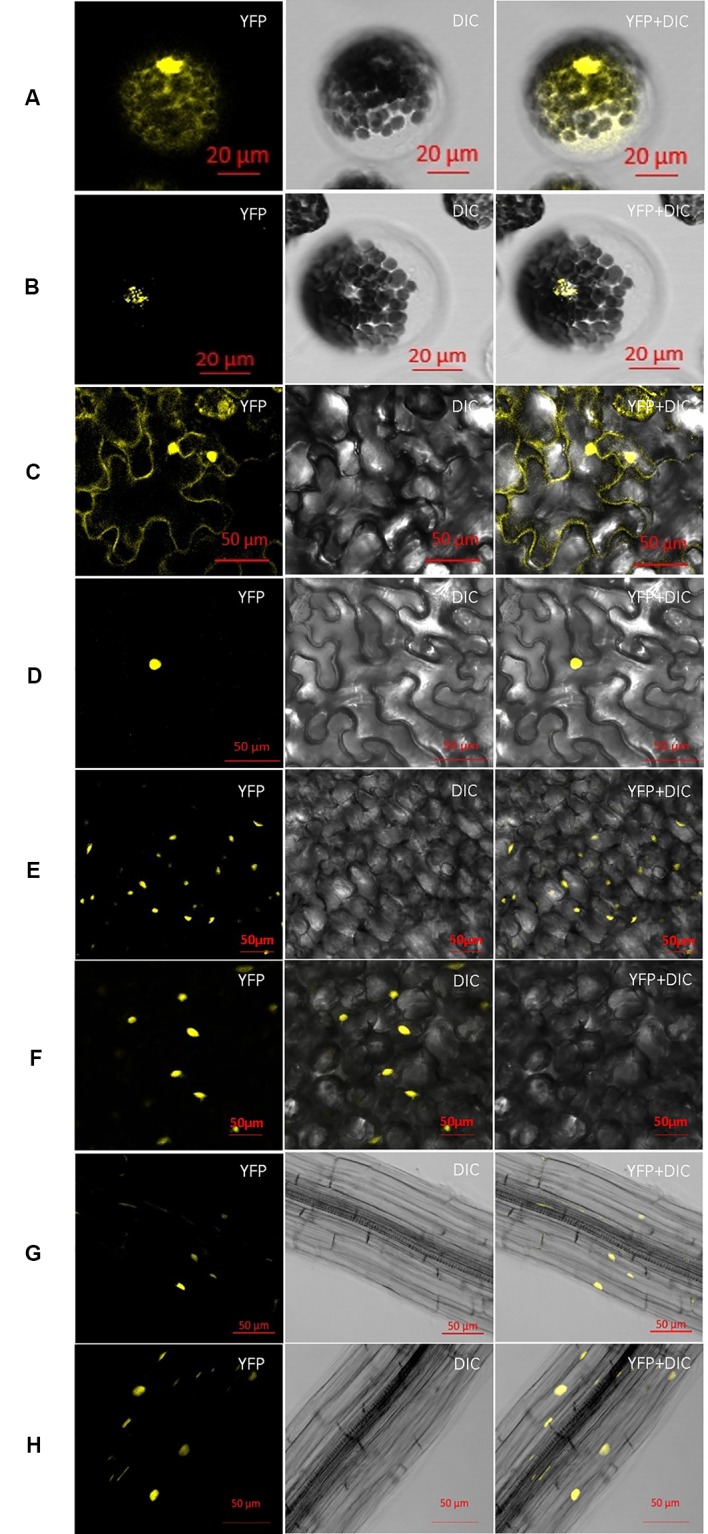
Subcellular localization of ZmCCA1a-YFP in different types of plant cells. Binary vectors encoding YFP **(A)** and ZmCCA1a-YFP **(B)** were transformed into mesophyll protoplasts of Arabidopsis. Agrobacterium tumefaciens strain GV3101 containing the YFP **(C)** or ZmCCA1a-YFP **(D)** constructs was used to agroinfiltrate *Nicotiana benthamiana* leaves. The *GV3101* strain containing the YFP **(E, F)** or ZmCCA1a-YFP **(G, H)** constructs was also used to generate transgenic Arabidopsis plants. The protoplast cells and plant tissues were examined under a confocal microscope. At least 10 F_1_ transgenic Arabidopsis lines were examined for visualization of the subcellular localization of ZmCCA1a-YFP signal. E and F, G and H indicate subcellular localization of ZmCCA1a-YFP signal in leaves and roots of two different ZmCCA1a-ox lines respectively. YFP, yellow fluorescent protein; DIC, differential interference contrast image.

Transiently expressed AtCCA1/AtLHY has been detected previously in the nuclei of plant cells (Wang et al., 1997; Carré and Kim, 2002; Lu et al., 2009; Seo et al., 2012). ZmCCA1b was targeted to the nuclei of onion epidermal cells (Wang et al., 2011). Given the high sequence similarity between ZmCCA1a and AtCCA1/AtLHY or ZmCCA1b, we speculated that ZmCCA1a would also be localized to the nuclei. To verify this prediction, *35S::ZmCCA1a-YFP* and *35S::YFP* expression constructs were infiltrated into leaves of *Nicotiana benthamiana* or transformed into mesophyll protoplasts of *Arabidopsis*. Visualization of the YFP signals by confocal microscopy showed that, in both transformants, YFP was dispersed throughout the cells but ZmCCA1a-YFP was localized only in the nuclei (**Figures 2A–D**). Subcellular localization of constitutively expressed ZmCCA1a was also examined. The *35S::ZmCCA1a-YFP* construct was transformed into *Arabidopsis* using the floral dip method and transgenic positive plants were checked. ZmCCA1a-YFP was localized exclusively in the nuclei of leaf and root cells (**Figures 2E–H**). These results were predictable given the high sequence similarity between ZmCCA1a and AtCCA1/AtLHY or ZmCCA1b, especially in the Myb region. It is reasonable to conclude that ZmCCA1a may act as a transcription factor similar to AtCCA1/AtLHY and is involved in gene expression regulation.

### Expression Pattern of *ZmCCA1a* Shows Circadian Rhythm

**Figure 3:**
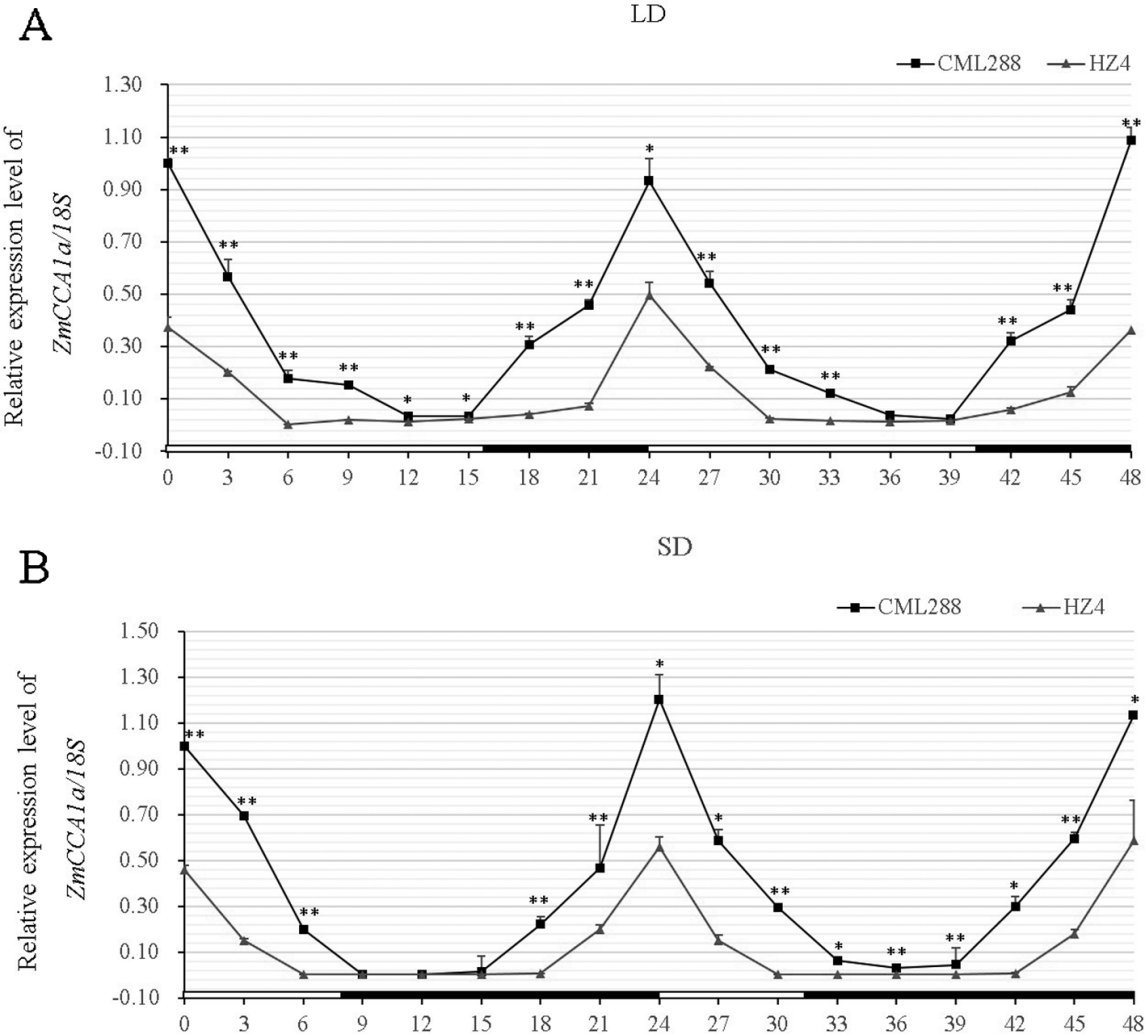
Diurnal rhythmic pattern of *ZmCCA1a* expression in leaves of maize inbred lines CML288 and HZ4. Maize CML288 and HZ4 seedlings were grown under long-day (LD; 16 h/8 h light/dark) **(A)** or short-day (SD; 8 h/16 h light/dark) **(B)** photoperiod regimes. Leaf samples were harvested from seedlings at the five-leaf stage at ZT0 and thereafter at 3-h intervals for 48 h. Leaves of three seedlings of each line were collected and mixed for RNA extraction. Experiments were performed three times with similar results. Relative transcript levels of *ZmCCA1a* were normalized to the expression level at ZT0 in CML288 under LD or SD. Expression levels of *ZmCCA1a* are shown as the average of three repeats. Day and night conditions are indicated with white and black bars on the horizontal axis. Error bars indicate standard error (n = 3). Student's *t-*est was used to analysis the significant differences in expression levels of *ZmCCA1a* between CML288 and HZ4. Significance levels of p < 0.05 and p < 0.01 were marked with * and **, respectively.

The expression patterns of *AtCCA1/AtLHY* and *ZmCCA1b* show circadian rhythm (Wang et al., 1997; Schaffer et al., 1998; Hayes et al., 2010; Wang et al., 2011; Ko et al., 2016). To determine the expression pattern of *ZmCCA1a*, leaf samples were harvested from five-leaved seedlings of CML288 and HZ4 growing under LD and SD conditions at Zeitgeber time 0 (ZT0), then at 3-h intervals for 48 h. Transcript levels were quantified by quantitative real-time PCR (qRT-PCR). In CML288 under LD, the *ZmCCA1a* transcripts showed a diurnal rhythm with peak expression in the morning (ZT0), and then decreased to the lowest level after approximately 12 h of light exposure before increasing again to the highest level the next morning (**Figure 3**). In previous studies, *ZmCCA1a* or *ZmCCA1b* transcript levels peaked at ZT3 or ZT4 (Hayes et al., 2010; Ko et al., 2016), which we speculated may reflect differences in the plant materials used because maize germplasm resources of different origins show substantial genetic differences. The present results are consistent with those of Wang et al. (2011), who showed that *ZmCCA1b* transcripts peaked at ZT0 under both SD and LD in the same maize inbred lines used in the present study. In addition, we could not rule out that imperceptible environmental factors might affect the photoperiod rhythm in maize differently in different studies. Under SD, the CML288 *ZmCCA1a* transcripts exhibited a similar diurnal rhythmic pattern, but the peak transcript level was much lower than that under LD (**Figure 3A**).

In HZ4 under LD and SD, *ZmCCA1a* transcript levels showed similar diurnal rhythmic patterns to those observed in CML288, but the peak in transcript levels in the morning was approximately 40% lower under LD (**Figures 3A, B**). Furthermore, the peak transcript levels were similar in HZ4 under LD and SD.

Promoter sequence alignment of *ZmCCA1a* in CML288 and HZ4 indicated low homology of the sequence upstream of the translation initiation site (ATG) (−538 bp and the upstream sequence). However, the sequence between −537 and −1 bp upstream of ATG showed extremely high identity, with discrepancy in only two nucleotides. An A to T (−533 bp) transversion and G to A (−371 bp) transition were observed in HZ4 compared with the sequence in CML288 (**Supplementary Figure 5**). Whether these nucleotide discrepancies resulted in the difference in *ZmCCA1a* expression pattern in CML288 and HZ4 requires further study.

### Overexpression of *ZmCCA1a* Delays Flowering of *Arabidopsis*

**Figure 4:**
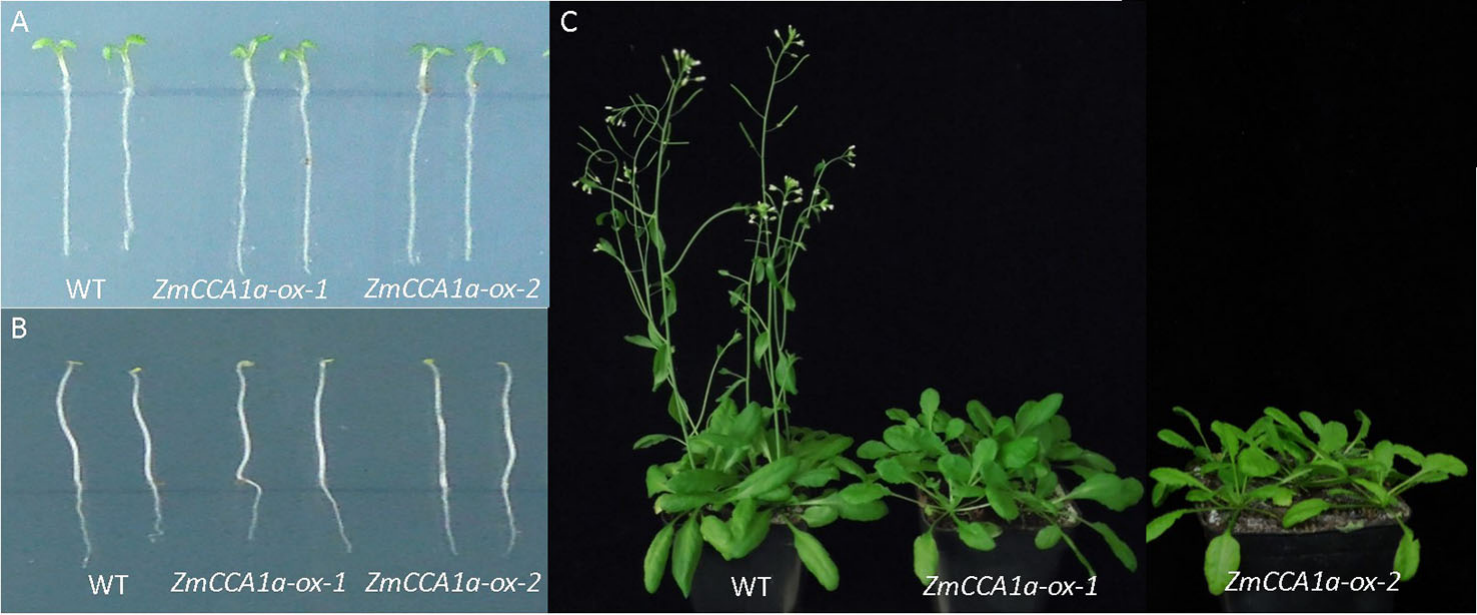
Hypocotyl-length and flowering-time phenotypes of *ZmCCA1a-ox* plants. **(A, B)** show the identical length of the hypocotyl of wild-type and *ZmCCA1a-ox* plants under long days (LD) and constant dark (DD). **(C)** Under LD, *ZmCCA1a-ox* plants showed delayed flowering compared with the WT. WT: wild-type; *ZmCCA1a-ox*: plant overexpressing *ZmCCA1a*. *ZmCCA1a-ox-1* and *ZmCCA1a-ox-2* indicate two different *ZmCCA1a-ox* lines. The phenotypes of lines from eight transgenic events were identified and showed late flowering compared with the WT, of which seven showed a hypocotyl length identical to that of the WT, and one showed a slightly longer hypocotyl. Two representative transgenic lines with the late-flowering and identical hypocotyl-length phenotypes compared with the WT are shown.

The circadian clock affects multiple plant phenotypes, including hypocotyl length and flowering time. Changes in expression of circadian-related genes may cause variation in these phenotypes. Overexpression of *AtCCA1/AtLHY* or *ZmCCA1b* is known to be associated with long-hypocotyl and late-flowering phenotypes in *Arabidopsis* (Schaffer et al., 1998; Alabadí et al., 2001; Mizoguchi et al., 2002; Kim et al., 2003; Thain et al., 2004; Wang et al., 2011). To investigate the effects of *ZmCCA1a* on these phenotypes, the T-DNA region containing the *35S::ZmCCA1a* expression cassette in the binary vector pBI121 was transformed into *Arabidopsis* using the floral dip method. The *ZmCCA1a* sequence was detected by PCR amplification in positive transgenic lines but not in WT plants. Expression of *ZmCCA1a* was verified by qRT-PCR. Under LD, the *ZmCCA1a-ox* and WT plants showed identical hypocotyl length (**Figure 4A**). Light is an inhibitory factor on hypocotyl growth (Somers et al., 1991; Quail et al., 1995; Filip et al., 2010). Thus, to eliminate the effect of light on hypocotyl elongation, *ZmCCA1a-ox* and WT seeds were germinated and grown in DD. The etiolated seedlings of both genotypes had hypocotyls of identical length (**Figure 4B**), which indicated that overexpression of *ZmCCA1a* did not impair hypocotyl elongation of *Arabidopsis*. Under LD, the flowering time of *ZmCCA1a-ox* plants was delayed by 5–8 days compared with that of WT plants (**Figure 4C**). The expression level of *ZmCCA1a* relative to that of endogenous *AtCCA1* and *AtLHY* at ZT0 was 7.08 and 7.08 in *ZmCCA1a-ox-1* seedlings, and 1.74 and 1.33 in *ZmCCA1a-ox-2* seedlings, respectively. However, the flowering time of the two *ZmCCA1a-ox* lines was similar (approximately 6 days later than the WT). Thus, we speculated that *ZmCCA1a* RNA transcripts accumulated in the two *ZmCCA1a-ox* lines and exceeded the maximum requirement threshold for this gene.

### ZmCCA1a Neither Forms Homodimers Nor Interacts With ZmCCA1b

**Figure 5:**
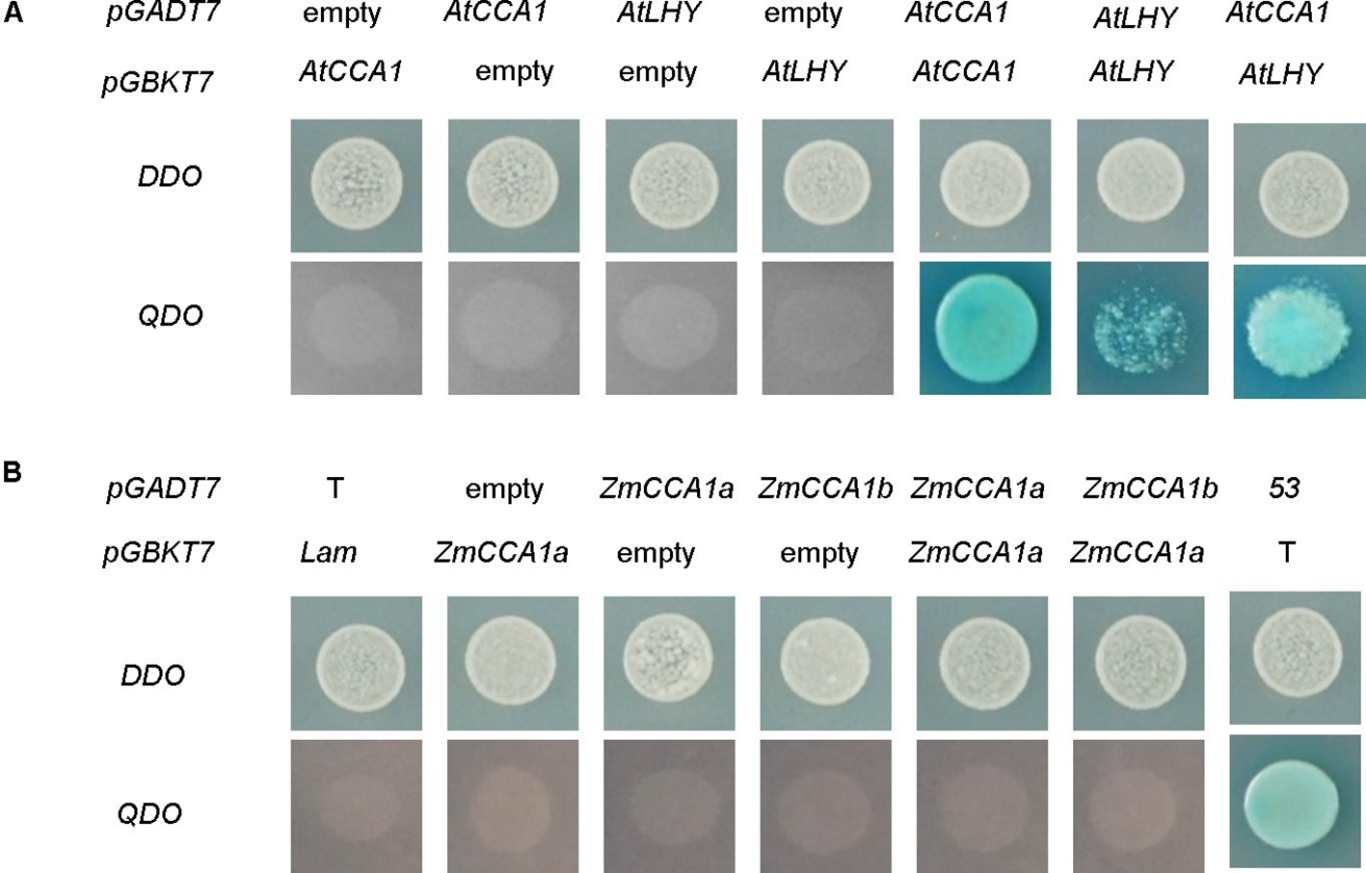
Protein interaction analysis of AtCCA1/AtLHY and ZmCCA1a/ZmCCA1b. Yeast Y2HGold cells were co-transfected with vectors fused with *AtCCA1/AtLHY* **(A)** and *ZmCCA1a/ZmCCA1b* **(B)**. The cells were grown on plates with double dropout medium (DDO; SD agar plate lacking leucine and tryptophan) to check for transformation, or quadruple dropout medium (QDO; SD agar plate lacking adenine, histidine, leucine, and tryptophan) with aureobasidin A (150 ng/ml) and Xα-Gal (40 µg/ml) to test for interaction. pGADT7, AD cloning vector; pGBKT7, DNA-BD cloning vector; Empty, empty vector of pGADT7 or pGBKT7; 53, pGBKT7-53; T, pGADT7-T; Lam, pGBKT7-Lam. Lam + T and 53 + T are the negative and positive controls, respectively.

Heterodimerization between clock components plays important roles in circadian systems (Dunlap, 1999; Edery, 1999; Iwasaki et al., 1999; Reppert and Weaver, 2002; Lowrey and Takahashi, 2004; Meyer et al., 2006). Protein interaction studies showed that AtCCA1 and AtLHY can form homodimers and heterodimers (Lu et al., 2009; Seo et al., 2012; **Figure 5A**). However, interaction between AtLHYs was weak because the growth of the yeast cells (Y2H) was repressed to some extent by 150 ng/ml aureobasidin A. These differences might have resulted from the different sensitivities of analysis systems used in the different studies. For example, Lu et al. (2009) used immunoprecipitation of YFP- and MYC-tagged AtLHY co-expressed in infiltrated leaves of *Nicotiana benthamiana*. An additional reason might be the involvement of other proteins (not expressed in yeast) that affect the strength of the interaction between AtLHY proteins, because AtCCA1 proteins have been detected in a larger complex containing other unknown proteins (Xavier et al., 2004). We performed a protein interaction assay of ZmCCA1a with itself and with ZmCCA1b using a yeast two-hybrid assay system. The results indicated that ZmCCA1a could not form homodimers nor interact with ZmCCA1b (**Figure 5B**).

### Circadian Rhythms are Disrupted and Expression of Circadian-Related Genes are Affected in *ZmCCA1a-ox* Plants

**Figure 6:**
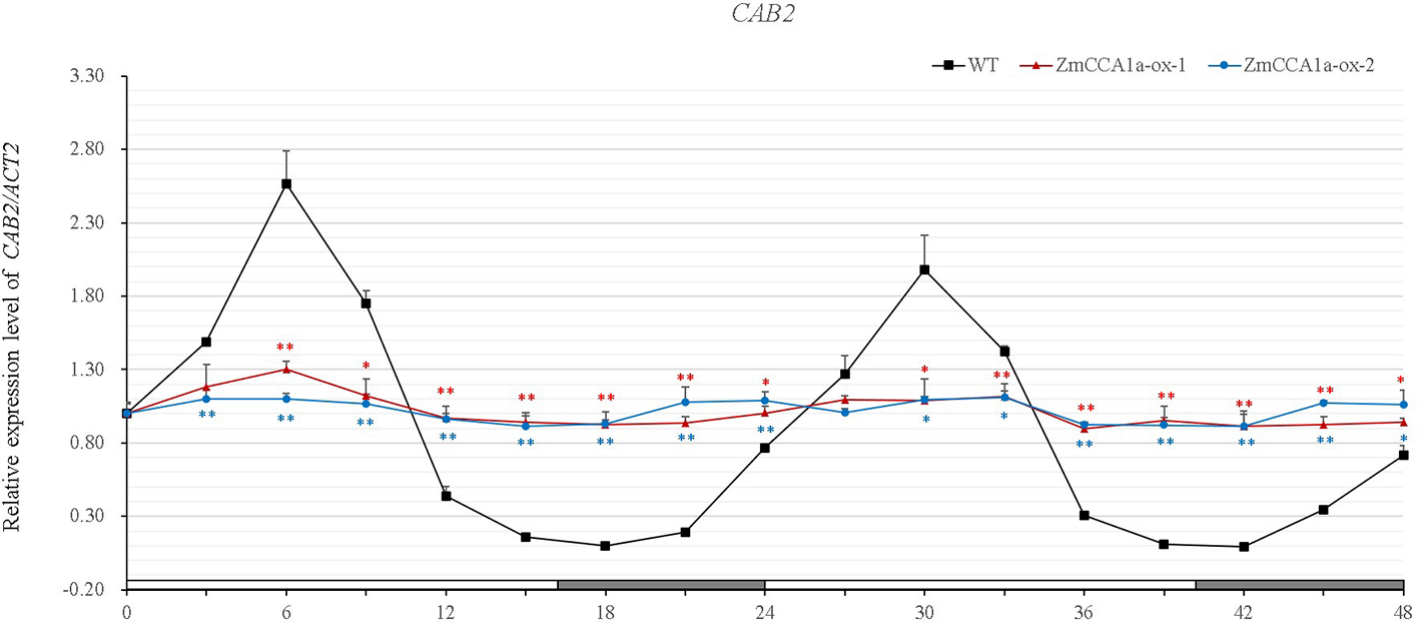
Circadian oscillations in accumulation of *CAB2* transcripts in the wild type and *ZmCCA1a-ox* lines. *Arabidopsis* seeds were grown on MS plates under long days for 10 days, and then transferred to continuous light (LL) after the start of the light period on day 11. Seedlings (at least 10 plants for each line) were harvested at ZT0 of day 12 and thereafter at 3-h intervals for 48 h. Experiments were performed three times with similar results. Relative transcript levels were normalized to the expression level of *CAB2* in the wild type (WT) at ZT0. Expression levels of *CAB2* are shown as the average of three repeats. *ZmCCA1a-ox-1* and *ZmCCA1a-ox-2* indicate two lines of transgenic *Arabidopsis* overexpressing *ZmCCA1a*. Day and subjective night conditions are indicated with white and gray bars on the horizontal axis. Error bars indicate standard error (n = 3). Student's *t* test was used to analysis the significant differences in expression levels of target genes between WT and *ZmCCA1a-ox* lines. Significance levels of p <0.05 and p < 0.01 were marked with * and **(red for *ZmCCA1a-ox-1* and blue for *ZmCCA1a-ox-2*), respectively.

*AtCCA1/AtLHY* are involved in light- and circadian-mediated regulation of gene expression. This regulation is disrupted in *AtCCA1*-overexpressing *Arabidopsis* under LL (Wang and Tobin, 1998). Recently, Ko et al. (2016) observed that *CAB2:LUC* expression rhythmicity is also dampened in *ZmCCA1*b-ox lines of *Arabidopsis*. To determine the effect of *ZmCCA1a* on circadian rhythmicity, we investigated the expression pattern of CAB2 (Lhcb1*1) in WT and *ZmCCA1a-ox* plants under LD and LL. Results of qRT-PCR analyses indicated that transcript accumulation of *CAB2* showed rhythmic expression in the WT and *ZmCCA1a-ox* lines under LD, although rhythmicity was compromised in *ZmCCA1a-ox* lines (**Supplementary Figure 6**). However, under LL, *CAB2* transcript accumulation showed a circadian rhythm in the WT, but the expression rhythmicity was abolished in *ZmCCA1a-ox* plants (**Figure 6**). Thus, *ZmCCA1a* was implicated in the circadian rhythm regulation of *Arabidopsis*.

**Figure 7:**
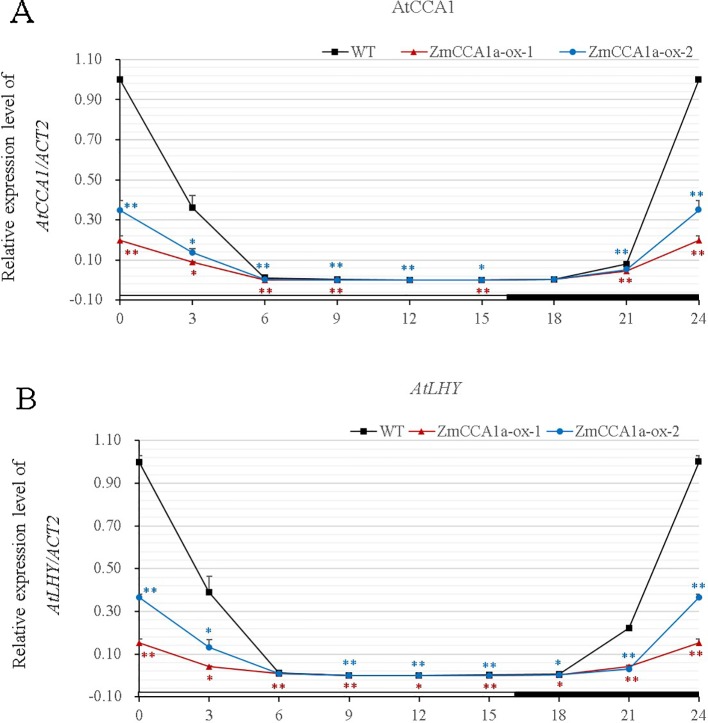
Overexpression of *ZmCCA1a* in *Arabidopsis* repressed expression of endogenous *AtCCA1*/*AtLHY*. Wild type (WT) and *ZmCCA1a-ox* plants were grown under long day (LD) conditions. Leaves of 3-week-old seedlings were harvested at ZT0 and thereafter at 3-h intervals for 24 h. Leaves of 10 seedlings of each line were collected and mixed for RNA extraction. Experiments were performed three times with similar results. Relative transcript levels were normalized to the expression levels of the corresponding genes in the WT at ZT0. Expression levels of *AtCCA1* **(A)** and *AtLHY* **(B)** are shown as the average of three repeats. *ZmCCA1a-ox-1* and *ZmCCA1a-ox-2* indicate two different *ZmCCA1a-ox* lines. Day and night conditions are indicated with white and black bars on the horizontal axis. Error bars indicate standard error (n = 3). Student's *t* test was used to analysis the significant differences in expression levels of target genes between WT and *ZmCCA1a-ox* lines. Significance levels of p <0.05 and p < 0.01 were marked with * and **(red for *ZmCCA1a-ox-1* and blue for *ZmCCA1a-ox-2*,) respectively.

High *AtCCA1* or *AtLHY* expression levels repress the transcription of endogenous *AtCCA1* and *AtLHY* in *Arabidopsis* (Schaffer et al., 1998; Wang and Tobin, 1998). To investigate the effect of *ZmCCA1a* on *AtCCA1*/*AtLHY* expression, we investigated the expression pattern of these two genes. Results of qRT-PCR analyses indicated that the transcriptional patterns of *AtCCA1*/*AtLHY* were extremely similar and showed a robust circadian rhythm in the WT. However, expression peaks of the two genes were much lower in *ZmCCA1a-ox* plants but were still rhythmic, albeit with a subtle waveform (**Figure 7**).

**Figure 8:**
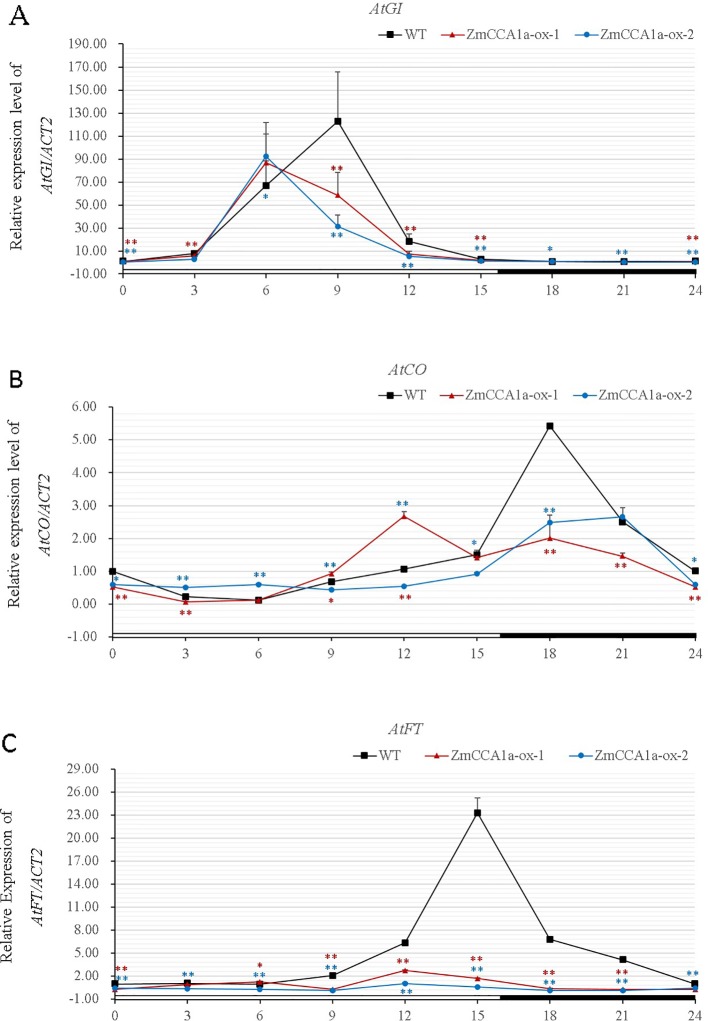
Effects of overexpression of *ZmCCA1a* in *Arabidopsis* on expression of downstream genes. Wild type (WT) and *ZmCCA1a-ox* plants were grown under long day (LD) conditions. Leaves of 3-week-old seedlings were harvested at ZT0 and thereafter at 3-h intervals for 24 h. Leaves of ten seedlings of each line were collected and mixed for RNA extraction. Experiments were performed three times with similar results. Relative transcript levels were normalized to that of the corresponding genes in the WT at ZT0. Expression levels of *AtGI* **(A)**, *AtCO* **(B)**, and *AtFT* **(C)** are shown as the averages of three repeats. *ZmCCA1a-ox-1* and *ZmCCA1a-ox-2* indicate two different *ZmCCA1a-ox* lines. Day and night conditions are indicated with white and black bars on the horizontal axis. Error bars indicate standard error (n = 3). Student's *t* test was used to analysis the significant differences in expression levels of target genes between WT and *ZmCCA1a-ox* lines. Significance levels of p <0.05 and p < 0.01 were marked with * and **(red for *ZmCCA1a-ox-1* and blue for *ZmCCA1a-ox-2*), respectively.

*AtCCA1/AtLHY* are core components of the oscillator in *Arabidopsis*. The genes transduce photoperiod signals to downstream genes in the circadian clock pathway. The expression of *AtGI*, *AtCO*, and *AtFT*, which are major components of the output pathway, is regulated directly or indirectly by *AtCCA1/AtLHY* (Zhang et al., 2014). The three aforementioned genes are involved in regulation of flowering time of *Arabidopsis*. Wang et al. (2011) reported that, in plants overexpressing *ZmCCA1b*, expression levels of *AtGI*, *AtCO*, and *AtFT* were lower than those in the WT. We found that overexpression of *ZmCCA1a* repressed expression of the three genes and changed the phase of their expression rhythm (**Figure 8**). This finding is consistent with the delayed-flowering phenotype of the *ZmCCA1a-ox* plants.

## Discussion

*AtCCA1/AtLHY* are major components of the circadian clock pathway in *Arabidopsis*. As core components of negative feedback loops of the oscillator, the genes regulate the expression of numerous downstream genes and participate in maintenance of the circadian clock rhythm (Schaffer et al., 1998; Green and Tobin, 1999; Alabadı´ et al., 2002; Esther et al., 2009; Lu et al., 2009; Seo et al., 2012; Nagel et al., 2015). The maize genome contains two *CCA1* (*LHY*) genes: *ZmCCA1a* (on Chr10) and *ZmCCA1b* (on Chr4). Wang et al. (2011) cloned and characterized *ZmCCA1b* and observed that it was a homolog of *AtCCA1* with a conserved function. *ZmCCA1b* dampened the circadian rhythmicity of *CAB2* expression in *Arabidopsis (*Ko et al., 2016), thus it may be a core component of the maize circadian clock. Furthermore, *ZmCCA1b* may be involved in biomass heterosis of maize hybrids (Ko et al., 2016).

In contrast, *ZmCCA1a* has been rarely studied. We cloned *ZmCCA1a* by map-based cloning. The putative ZmCCA1a amino acid sequence contained a single Myb domain at the N-terminus, which showed more than 94% similarity with the Myb domains of *ZmCCA1b* and *AtCCA1*/*AtLHY*. These results indicated that these four genes may share conserved functions. The sequence similarity between the ZmCCA1a and ZmCCA1b amino acid sequences was 87.24%. This probably resulted from the genome doubling that occurred during maize evolution (Schnable et al., 2011). A gene synteny analysis may throw further light on this issue.

Previous studies indicated that transiently expressed ZmCCA1b and AtCCA1/AtLHY were localized to the nuclei in different plant species (Wang et al., 1997; Lu et al., 2009; Wang et al., 2011; Seo et al., 2012). As a putative transcription factor and in view of its similarity to the other three homologs, we predicted that ZmCCA1a would be localized to the nuclei of plant cells. This was confirmed by subcellular localization analysis of transiently expressed ZmCCA1a-YFP in mesophyll protoplasts of *Arabidopsis* and agroinfiltrated tobacco leaves, and constitutively expressed ZmCCA1a-YFP in transgenic *Arabidopsis* plants.

The transcript level and circadian rhythm of *ZmCCA1a* were observed in two maize inbred lines that differed in photoperiod sensitivity (photoperiod-sensitive CML288 and photoperiod-insensitive HZ4). Similar to *AtCCA1/AtLHY*, *ZmCCA1a* transcripts showed a circadian rhythm with peak expression observed close to the start of the light period under both LD and SD, which implied *ZmCCA1a* may be involved in circadian regulation. Peak expression of *ZmCCA1a* was distinctly repressed by SD in CML288 but was little affected in HZ4 (**Figure 3**). The low peak transcript level of *ZmCCA1a* in HZ4 might partially explain the relative insensitivity of HZ4 to photoperiod. Transgenic *Arabidopsis* plants overexpressing *AtLHY/AtCCA1* or *ZmCCA1b* show delayed flowering (Schaffer et al., 1998; Wang and Tobin, 1998; Mizoguchi et al., 2002; Wang et al., 2011), which agrees with the present finding that flowering time was delayed in *ZmCCA1a-ox* plants (**Figure 4C**). Ko et al. (2016) showed that overexpression of *ZmCCA1b* reduced growth of *Arabidopsis* and maize. However, we did not observe reduced growth of *ZmCCA1a-ox Arabidopsis* plants compared with WT plants. This may reflect the partially redundant functions of *ZmCCA1a* and *ZmCCA1b*. In addition, Ko et al. (2016) judged the growth vigor by the dried aerial biomass; we could not rule out the possibility that the phenotypic identification in our study was not sensitive enough to detect a difference in biomass between WT and *ZmCCA1a-ox* plants. Accordingly, a more detailed biomass quantification of WT and *ZmCCA1a-ox* plants might resolve this issue.

The hypocotyl length of *ZmCCA1a-ox* plants was identical to that of WT plants under LD and DD conditions (**Figures 4A, B**), which was unexpected, given the high conservation among the amino acid sequences of *ZmCCA1a*, *ZmCCA1b*, and *AtCCA1/AtLHY*, especially in the Myb domain. Overexpression of *CCA1β*, which encodes an AtCCA1 alternative splicing variant that lacks the Myb DNA-binding motif but still interacts physically with AtCCA1 and AtLHY, represses the elongated hypocotyl phenotype in *AtCCA1*-overexpressing plants (Seo et al., 2012). This finding suggested that the amino terminal containing the Myb domain plays important roles in maintaining the biological function of *AtCCA1*, including promotion of hypocotyl elongation. mCCA1, which mimics the unphosphorylated form of AtCCA1 and cannot physically interact with AtCCA1 and AtLHY, retains the ability to promote hypocotyl elongation, thereby confirming that phosphorylation of AtCCA1 is dispensable for this phenotype (Xavier et al., 2004). Overexpression of *ZmCCA1a* (lacking the Myb domain) showed a compromised function in the circadian rhythmicity of *CAB2* expression in *Arabidopsis* and had less effect on the biomass inhibitory function than *ZmCCA1b* in *Arabidopsis* and maize; thus the Myb domain is required for the clock function (Ko et al., 2016). In addition, since the C-terminal sequences vary considerably between ZmCCA1a and AtCCA1/AtLHY or ZmCCA1b, we concluded that the Myb domain (or the amino terminal region containing the Myb domain of ZmCCA1a) is essential for the function of ZmCCA1a in the circadian clock, but the hypocotyl elongation-promoting activity requires both the Myb domain and amino acids outside this domain.

An AtCCA1 mutant (a *CCA1*-null line) of *Arabidopsis* shows shortened expression periods of several circadian-related or circadian-regulated genes, such as *AtLHY*, *CAT2*, and Lhcb1*1, although the function of *AtLHY* was unaffected (Green and Tobin, 1999). One explanation is that heterodimers of AtCCA1 and AtLHY perform functions that are not exhibited by AtCCA1 monomers or homodimers formed by AtCCA1. However, the yeast two-hybrid assay system showed that ZmCCA1a did not form homodimers nor interact with ZmCCA1b (**Figure 5**). Lu et al. (2009) proved that the amino acids in positions 136 to 316 of AtCCA1 were important for formation of heterodimers with AtLHY. The sequence similarity between ZmCCA1a and AtLHY (12.6%) or AtCCA1 (13.7%) in this region is quite low, which might explain the absence of ZmCCA1a homodimers and heterodimers with ZmCCA1b. Determining the biological function of the interaction between AtCCA1 and AtLHY and the absence of heterodimers between ZmCCA1a and ZmCCA1b is an interesting problem for future studies to address.

In *ZmCCA1a-ox* plants, the circadian rhythm was disrupted under LL (**Figure 6**) and expression of endogenous AtCCA1 and AtLHY was repressed (**Figure 7**). Furthermore, expression of *AtGI*, *AtCO*, and *AtFt*, which are involved in circadian regulation of flowering time (Locke et al., 2006; Shim and Imaizumi, 2014; Shim et al., 2017), was significantly repressed in *ZmCCA1a-ox* plants (**Figure 8**). This finding is consistent with the delayed flowering phenotype. Because *ZmCCA1a-ox* lines showed no other phenotypes compared with the WT, such as reduced growth in *ZmCCA1b-ox Arabidopsis* lines (Ko et al., 2016), the delayed flowering of *ZmCCA1a-ox* lines reflects indirect effects derived from the entire clock disruption. These results indicate that ZmCCA1a shares a conserved function with AtCCA1/AtLHY, at least partially, because the WT and *ZmCCA1a-ox* plants showed delayed flowering but similar hypocotyl lengths. Thus, ZmCCA1 might be a major component of the circadian clock pathway in maize. In view of the partially redundant biological function in *Arabidopsis* and sequence diversity (mainly at the C-terminus) between *ZmCCA1a* and *AtCCA1*/*AtLHY*, it would be intriguing to determine (1) the exact sequence in the C-terminus of ZmCCA1a that is responsible for the absence of homodimer or heterodimer formation, which is observed in AtCCA1 and AtLHY, and the biological meaning of the interactions; (2) how much of the *ZmCCA1a* function in *Arabidopsis* can be extended to maize, its native host; and (3) the stage at which the function of *ZmCCA1a* is regulated, given that *AtCCA1* can be regulated both post-transcriptionally and post-translationally, such as by alternative splicing and protein interaction and phosphorylation (Xavier et al., 2004; Esther et al., 2009; Seo et al., 2012). *AtCCA1* is involved in the circadian regulation of temperature responses in *Arabidopsis* (Seo et al., 2012) and *ZmCCA1a* might play an important role in linking the photoperiod to stress tolerance responses (Su et al., 2018; Tian et al., 2019). Thus, it will be informative to determine the role of *ZmCCA1a* in stress tolerance responses and how photoperiod and stress tolerance responses are linked.

## Materials and Methods

### Plant Materials and Fine Mapping of the QTL Containing *ZmCCA1a*

The maize inbred line Huangzao 4 (HZ4; photoperiod insensitive), which is a representative of the Tangsipingtou heterotic group in China, was stored in Yanhui Chen's laboratory. The maize inbred line CML288 (photoperiod sensitive) was provided by the National Maize and Wheat Improvement Center, Mexico. *Arabidopsis thaliana* ecotype Columbia was used for generation of transgenic plants.

Previously, for mapping of flowering time related traits, CML288 (non-recurrent parent) and HZ4 (recurrent parent) were crossed to develop mapping populations, including multiple populations from BC_1_F_1_ to BC_8_F_1_. Responses of CML288 and HZ4 to LD and SD conditions were described by Wang et al. (2011). The QTL containing *ZmCCA1a* was mapped between the markers *S873* and *IDP582* on Chr10. To further narrow this genomic region, mapping populations derived from selfing of the BC_8_F_1_ population (comprising BC_8_F_2_ with 1,682 plants, BC_8_F_3_ with 112 plants, and BC_8_F_4_ with 1,350 plants) were developed.

For the development of markers for fine mapping, the genomic sequence flanked by the markers *S873* and *IDP582* on Chr10 of maize “B73” were obtained from the Maize Genetics and Genomics Database (ftp://ftp.gramene.org/pub/gramene/release-61/fasta/zea_mays/dna/). Simple sequence repeats (SSRs) were searched using SSR Hunter (Li and Wan, 2005) and primers for selected SSRs were designed using Primer Premier 5.0 software (Premier Biosoft International, Palo Alto, CA, USA). The primer sequences and the corresponding physical position on Chr10 of B73 are presented in **Supplementary Table 1**.

### Field Experiments

All field experiments were performed at the experimental farm of Henan Agricultural University, Zhengzhou, Henan Province (34.86°N, 113.60°E) in summer of each year (2011–2015). The soil type of the farm is sandy clay. The study area experiences a temperate-subtropical, wet-subhumid monsoon climate. The growth cycle extended from 5 June to 15 October, during which the maximum, minimum, and average temperatures were 30°C –35°C, 13°C –23°C, and 19°C –26°C, respectively. The average photoperiod in Zhengzhou is 14.25 h during this period.

The maize mapping populations were planted in the field in 4 m-long rows with spacing of 0.67 m between rows and 0.25 m between plants. The flowering time was used for mapping of the CML288 *ZmCCA1a* allele. The flowering time was evaluated as days to pollen shedding (DPS), and days to tasseling (DT). DPS was calculated from the date of sowing to the date of first anther dehiscence from the central spike. The DT was calculated from the date of sowing to the date of tassel emergence from the uppermost leaf. The CML288 ZmCCA1 allele delayed both the DPS and DT of HZ4 under LD (in Zhengzhou).

### PCR Amplification of *ZmCCA1a* and *ZmCCA1b*

The BAC sequences from the CML288 genome containing *ZmCCA1a* were screened using primers designed from the *ZmCCA1a* sequence of maize “B73” (GenBank: AC215881) and sequenced. To amplify *ZmCCA1a*, the *ZmCCA1a* CDS of B73 (GenBank: EU954568.1) was aligned to BAC sequences from the maize inbred line CML288 containing the *ZmCCA1a* region; primers specific for *ZmCCA1a* (ZmCCA1a-FP and ZmCCA1a-RP; **Supplementary Table 4**) were designed from the deduced *ZmCCA1a* sequence of CML288.

To amplify *ZmCCA1b*, the *ZmCCA1b* CDS (GenBank: EU955544.1) was used as the reference sequence to design the primers *ZmCCA1b*-FP and *ZmCCA1b*-RP (**Supplementary Table 4**). The *ZmCCA1b* and *ZmCCA1a* CDSs were amplified by PCR using the designed primer pairs and the cDNAs were reverse-transcribed from total mRNA extracted from CML288 and HZ4.

### Yeast Two-Hybrid Analysis

For the assays of homodimer or heterodimer formation by AtCCA1 and/or AtLHY, the CDSs of *AtCCA1* (1827 bp) and *AtLHY* (1938 bp) were cloned into the pGBKT7 and pGADT7 vectors. For the assays of homodimer or heterodimer formation by *ZmCCA1a* and/or *ZmCCA1b*, the CDS of *ZmCCA1a* (2157 bp) was cloned into the pGBKT7 and pGADT7 vectors and the CDS of *ZmCCA1b* (2163 bp) was cloned into the pGADT7 vector. Genes were PCR amplified using the primers listed in **Supplementary Table 4** and homology-based cloning was carried out using the Hieff Clone™ One Step Cloning Kit (Cat No. 10911, Shanghai, China) to obtain the target constructs. The obtained constructs were verified by sequencing.

Yeast two-hybrid (Y2H) analysis was performed using the Matchmaker Y2Hsystem (Clontech, Mountain View, CA, USA). Before the analysis, we tested bait constructs for autoactivation and toxicity by spreading transformed competent Y2HGold cells on SD/−Trp plates and SD/−Trp/X-a-Gal plates (with 0, 100, 125, 150, 200, 300, or 500 ng/ml aureobasidin A). The results indicated that all bait constructs showed no toxicity to yeast cells and 150 ng/ml aureobasidin A effectively inhibited the growth of yeast cells and provided a clear background. For Y2H analysis, Y2HGold yeast cells were co-transfected with vectors fused with *AtCCA1* and/or *AtLHY* or *ZmCCA1a* and/or *ZmCCA1b*, and were grown on plates with double dropout medium (lacking tryptophan and leucine) to check for transformation, or quadruple dropout medium (lacking tryptophan, leucine, histidine, and adenine) with 150 ng/ml aureobasidin A and X-α-Gal (40 µg/ml) to test for interaction. The plasmids pGBKT7-Lam + pGADT7-T and pGBKT7-53 + pGADT7-T were used as negative and positive controls, respectively.

### Phylogenetic Analysis of Selected ZmCCA1a and ZmCCA1b Proteins

Amino acid sequences were aligned using ClustalW and the phylogenetic tree was generated by the neighbor-joining method based on 1,000 bootstrap repetitions by MEGA X (https://www.megasoftware.net/). The deduced amino acid sequences were downloaded from NCBI (http://www.ncbi.nlm.nih.gov/).

### Nuclear Localization of *ZmCCA1a* in Different Plant Cell Systems

To generate the vector encoding ZmCCA1a-YFP (yellow fluorescent protein), *ZmCCA1a* CDS was amplified using the primers ZmCCA1a-FP and ZmCCA1a-RP (**Supplementary Table 4**) with cDNA of CML288 as the template. The PCR products of *ZmCCA1a* were cloned into the binary vector pBI121 (*pBI121-P35S-YFP*) (kindly provided by Dr Lei Zhao). The pBI121 vector contains kanamycin and hygromycin resistance screening genes for bacteria and plants, respectively. The cloning was carried out using a homology-based cloning method (Hieff Clone™ One Step Cloning Kit, Cat No. 10911, Shanghai, China) and the *pBI121-P35S-ZmCCA1a-YFP* construct was obtained. *pBI121-P35S-YFP* and *pBI121-P35S-ZmCCA1a-YFP* were introduced into *Agrobacterium tumefaciens* strain GV3101 to agroinfiltrate *Nicotiana benthamiana* leaves and to generate transgenic *Arabidopsis* plants.

Mesophyll protoplasts were isolated from WT *Arabidopsis* plants and transfected following the procedure described by Wu et al. (2009).

Agroinfiltration of *N. benthamiana* was carried out on leaves of 3- to 4-week-old plants and transfected in accordance with the method of Shi et al. (2014).

Transgenic *Arabidopsis* plants were generated using the floral dip method (Clough and Bent, 2010).

Protoplasts and plant tissues were visualized under a confocal microscope (Zeiss LSM710). At least 10 T_1_ transgenic *Arabidopsis* lines were examined for visualization of the subcellular localization of YFP signal. Wavelengths for excitation and emission of YFP were 488 nm and 525 nm, respectively.

### Generation of Transgenic *Arabidopsis* Plants Overexpressing *ZmCCA1a*

To generate transgenic *Arabidopsis* plants overexpressing *ZmCCA1a* (*ZmCCA1a-ox*), the CDS of *ZmCCA1a* (2,157 bp) was amplified using the primers ZmCCA1a-ox-FP and ZmCCA1a-ox-RP (**Supplementary Table 4**), followed by homology-based cloning with the Hieff Clone™ One Step Cloning Kit (Cat No. 10911, Shanghai, China) into the binary vector pCambia-1300-221, which contains kanamycin and hygromycin resistance screening genes for bacteria and plants, respectively. The resultant construct was verified by sequencing and transfected into *Agrobacterium tumefaciens* strain GV3101. *Agrobacterium* transformation of *Arabidopsis* was performed as described by Clough and Bent (2010). Transgenic plants were confirmed by PCR using *ZmCCA1a-*specific primers. Overexpression of *ZmCCA1a* was verified by qRT-PCR analysis using *ZmCCA1a-*specific primers (**Supplementary Table 2**). Eight transgenic *Arabidopsis* lines expressing ZmCCA1a were used for flowering-time and hypocotyl-length phenotyping. Three of the eight lines were selected at random for gene expression analysis and representative results for two lines were presented.

### Photoperiod Treatment and Sampling

Maize kernels were sown in pots (30 cm diameter × 40 cm height) containing nutrient soil (loamy soil:perlite:vermiculite (w/w/w); 3:1:1) and grown at 28°C in an artificial climate chamber (PRX-1000B, SaiFu, Ningbo, China). Photoperiod treatments were 16 h/8 h (light/dark) for LD and 8 h/16 h (light/dark) for SD, supplied by LED lighting with intensity of 1,000 lux. Leaf samples of seedlings at the five-leaf-stage of each line (three for each line) were collected and mixed at the beginning of the light period (Zeitgeber time 0: ZT0) and thereafter at 3-h intervals for 48 h. Samples were frozen immediately in liquid nitrogen and stored at −80°C.

For germination and growth of *Arabidopsis* plants, seeds were sterilized as described by Yong et al. (2014) and plated on Murashige and Skoog (MS) medium supplemented with appropriate antibiotics (MS + 100 mg/L timentin for the WT; MS + 100 mg/L timentin + 20 mg/L hygromycin for transgenic plants). Sterilized seeds were imbibed at 4°C for 2 days, and then cultured at 22°C under DD or LD conditions supplied by LED lighting with intensity of 1,000 lux. Hypocotyl length was measured after 5 days of culture. Seedlings were transferred to nutrient soil when 10–14 days old.

For qRT-PCR analysis of gene expression (except *CAB2*), leaves of seedlings at the 3-week stage of each line (10 individuals for each line) were collected and mixed for RNA extraction. For analysis of *CAB2* transcripts, *Arabidopsis* seedlings were grown on MS plates under LD for 10 days, then divided into two pools and treated with LD or shifted to LL at the start of the light period on day 11. Seedlings were harvested at ZT0 on day 12 then at 3-h intervals (for 24 h for plants treated with LD and for 48 h for plants treated with LL).

### Quantitative Real-Time PCR Analysis

Total RNA was extracted using the TransZol reagent following the manufacturer's instructions (Cat No. ET101, TransGen Biotech, Beijing, China). Genomic DNA removal and reverse transcription (with oligo-dT primers) of the RNA samples were performed using the TransScript^®^ One-Step gDNA Removal and cDNA Synthesis SuperMix Kit (Cat No. AT311, TransGen Biotech). The qRT-PCR was performed with the TransStart^®^ Top Green qPCR SuperMix Kit (Cat No. AQ131, TransGen Biotech). Primers used for qRT-PCR of the target genes and internal controls (*18S* for maize and *ACT2* for *Arabidopsis*) are listed in **Supplementary Table 2**.

Experiments were performed three times and each analysis was carried out with three technical repeats. The transcript level in each repeat was presented as the average of three technical repeats.

## Data Availability Statement

All datasets for this study are included in the article/**Supplementary Material**.

## Author Contributions

YC, YS, and WJ conceived the project. YS and WJ drafted the manuscript. XZ performed fine mapping of *ZmCCA1a* and cloning of related genes. SG, SD, YW, and ZH performed the gene functional analysis. All authors read and approved the final manuscript.

## Funding

This work was supported by grants from the National Natural Science Foundation of China (No. 31601318) and the Science and Technology Project in Henan Province (No. 182102110344).

## Conflict of Interest

The authors declare that the research was conducted in the absence of any commercial or financial relationships that could be construed as a potential conflict of interest.
