# Supplementary material for: ZmCCA1a on Chromosome 10 of Maize Delays Flowering of Arabidopsis thaliana
Source: Front Plant Sci. 2020 Feb 20;11:78. doi: 10.3389/fpls.2020.00078 (PMC7044342; doi:10.3389/fpls.2020.00078)
Supplement: Supplementary file 1 [file DataSheet_1.pdf]

ZmCCA1a on Chromosome 10 of Maize Delays Flowering of *Arabidopsis thaliana*

Yong shi[1†], Xiyong Zhao[3†], Sha Guo[1], Shifeng Dong[1], Yanpeng Wen[1], Zanping Han[4], Weihuan Jin[2*], Yanhui Chen[1*]

[1]College of Agronomy/National Key Laboratory of Wheat and Maize Crop Science, Henan Agricultural University, Zhengzhou, Henan, China

[2]College of Life Sciences, Henan Agricultural University, Zhengzhou, Henan, China

[3]Crop Research Institute, Anhui Academy of Agricultural Sciences, Hefei, Anhui, China

[4]College of Agronomy, Henan University of Science and Technology, Luoyang, Henan, China

† These authors contributed equally to this work.

*** Correspondence:**

Yanhui Chen, Agricultural College, Henan Agricultural University, Zhengzhou, Henan, China

e-mail: chy9890@163.com

Weihuan Jin, College of Life Sciences, Henan Agricultural University, Zhengzhou, Henan, China

e-mail: Jinjinwh@163.com

Keywords: *maize, ZmCCA1,* overexpression, circadian rhythms, photoperiodic flowering

Supplementary Table 1 Primer sequences for DNA markers used for fine mapping of the locus containing *ZmCCA1a*.

Maker Name	Primer Name	Primer Sequence	RefGen_v4 position in B73
bnlg210	bnlg210F	GCCTCGCACCAAGACATAATA	27656215
	bnlg210R	TGCCCCATTTGAGTAGACTTC	
S873	S873F	GCTGGACCACCATCACAAC	76273093
	S873R	ACACATAGACGAAGTTGAAGCA	
S334	S334F	TGCTGGTGAGCCTCTTAGTC	77071150
	S334R	TGTTGCTGCCAAGATACACTC	
QCCA5-2	QCCA5-2F	CTGTTCTTGCGACCTTGAGAT	77699557
	QCCA5-2R	AATTCCAGAGCCATATGAGCAT	
GS575	GS575F	CTTCTACCAGTGTGCCCAAG	77548207
	GS575R	GACATGCTCCATCTGCCCTA	
IDP7868	IDP7868F	GTTGGTTCTTCTGGCATTCG	80160229
	IDP7868R	GCACCCGTTGAATATGTCG	
S147	S147F	GATCCATCATCCATCGTCAG	83253972
	S147R	CAGTTTCTGCCACAGCTAG	
S951	S951F	TCTATTCACGCTTGTGCTTATG	84831086
	S951R	GTCTTGCGTCTGCCACTATA	
S165	S165F	TCATCGTTGTCTTCGTGATAGA	87264986
	S165R	TTGTCAATCTTGGTTGCTTGG	
S748	S748F	TGACAGCATTATCATCACCCAT	87426862
	S748R	ACCAGCGTTAACTGCTAGTG	
GS617	GS617F	ACGACGGTGACAGACAATTAG	87620093
	GS617R	CAGACCTTTGTTTGCCTCCT	
PIF3 1-2	PIF3 1-2F	CAAGTCGTCGATGCTGGAG	87874663

Maker Name	Primer Name	Primer Sequence	RefGen_v4 position in B73
	PIF3 1-2R	ATCCTGTTTGTTTGGCTCTCTC	
IDP582	IDP582 F	ACCGATGTTGCAAAGAAAGG	91161576
	IDP582 R	GGTATAGCTTTGGCATTGGG	
3-7	3-7F	CGCCCTGCTCCACTTCATA	94802843
	3-7R	TGGCACCATCCTCTCCGTC	
S456	S456F	TTGCTGTTGCTTTCGTCGTC	95626643
	S456R	TCCACTTCCTCTCCCACCTC	
GS668	GS668F	GCACTGGGTGTACCTACAG	87977497
	GS668R	ACCTCCGTCCATCAATCCA	
QCCA4-2	QCCA4-2F	ACTTTGTTTCAGGGAATGGTCT	77699393
	QCCA4-2R	GCCAGAGCAAGCAGTCAAG	
umc1330	umc1330F	AGCAAAGAAGCCAAAGAGCAACT	123897197
	umc1330R	GTCACCACCGTCTGCTGGTA	

Supplementary Table 2 Genomic sequence of the BAC clone containing *ZmCCA1a* on chromosome 10 in the maize inbred line CML288. The *ZmCCA1a* CDS sequence is highlighted in yellow.

TTAGGTCCTAAGAACTTGGTATTGTCCACCTACGAAAAGGAATATCTAGCCATTTTATTTGCAGTTGATCACTGGCGCCCTTACCTCCAACATGGAGAATTTTTTATCA
AGACTGATCAGCAAGCCTAACCCATCTGGAAGACCAAAAGTTGTCGACTATCTGGCAACAAAAAGCCTTAACCAAGCTGTTGGGTCTCCAATTCCGCATTATATATA
AGAAAGGAGTGGAAAACCGTGTGGCAGATGCTCTTTCTAGACGGCCAAACCTATGCACTGATGGTTCCAAACTGCAACTCAACGTGTTAGCAACTTCGACAGTGG
TACCAAGTTGGCTCACCGAGGTGACTAACAGCTATGAGGAGAATGAGGAAGCTAAGAGCATTCTGAGTACGCTTGTTGCAGGGGATACAATAGAGCCATTCACTT
TGGTATCAGGAGTGATCAAGCATAAAGGAAGGATTTGGCTGGGTAGCAGTAACCAAGTGCAATTAAAGGTGATGACTGCCCTTCATGACAGTCCTATAGGAGGTC
ATTCTGGGTTCCCGGTAACCTATCATCGCATCAAGAAGCTGTTTTACTGGGCTGGCATGAAAGGAAAGATCAAGGAGTTCGTGCAGTCTTGTGAGATTTGCACAAA
GGCCAAGGCCGACAGAAATAGATACCCCGGTCTGCTATTGCCATTACTCATACCGGATCAGGCTTGGCAAGTTATTAGCTTGGATTTTATCTCTGGATTACCTACATC
CCGACGCTTCAACTGCATATTGGTGGTAGTAGATAAATTTTCAAAATATGCTCACTTCCTAGCCATGTCCCATCCTTTTACTGCTCTATCTGTGGCCAAGCTGTTCCTAT
CAGAGGTCTACAAACTCCATGGCCTACCATTGTCTATCATCTCTGACAGGGATCCAATATTCACCAGTAACTTGTGGCAGGAATTGTTTAAGTTGGTGGGCACAAAA
TTATGCCTCAGTAGCGCATATCACCCCCAATCTGATGGCCAAACTGAACGGGTCAACCAATGTGTGGAAGCTTATCTTCGATGCTTTGTCCATGGCTGCCCGAAACA
GTGGTCCAATTGGCTATCTCTTGTAGAGTTTTGGTATAATACTTGTTTTCACACTGCACTGGGTCAGTCCCCGTTTGAGGTTTTGTATGGCCACACTCCATCCCAGTT
AGGACTGTCAACTATTGAACAATGTCAAAGTGCTGATCTGCAAACATATCTGGCAACAAGACAGCTTATGCTCCAACAGGCCAAGTTACATCTTCAAAGGGCCCAA
GATCGGATGAAAAAACAAGCTGACAAAGGAAGATCTGAAAGGGTGTTTCAAGTGGGACAACGTGTATTTTTGAAACTGCAGCCATTTTGTCAGAGTTCGATGGG
GAGCAGATTGAACGCTAAATTGTCATTTTGCTATTTTGGACCGTTTCTGATCACAAAACAGGTGAATCCCGTGGCTTATGAACTGGCATTACCAGAAGGAAGTGCG
ATTCATCCGGTCTTTCATGTGTCCCAACTTAAATCAGCCGAAGGAACTCGTATCACAACCGATGCAACCATGCCAGACCTGCTGCAGGAGCTGAAAATACCCACAG
AGGTGCTGGAGTCTAGGCTGCTTCGCAAAGGTAACAAGGTAATAGCTCAACTCCTAATCTGATGGTCTAACTGGCCGGCTTCATTGTCTACCTGGGAAGATGAACA
CGCCATCAAGCAGCAGTTCCCACGGGCTCCAGCTTGGGGACAAGCTGTCTGTCAAGGGGAAGGGGATGTTAGCAACACAGGGCACCTGAACACCGGGAAGGAT
GTTGCGATGGAAGAGGCGGACGCTGAGGTTCAGGAAGCAAAGAGGAGCACTCGGGCTAAAAGGTCGAATCCAAAGTATATAGGAAATGTGTGGATGAATTAGG
CCCATGTAACGGGCAGCCCATGGCAGTTATATAAAAGCCTGGGTTGTAATGGAGAAGACAGACTTTGGCTGGCGGAACTGGAACACGAAAGAACCGCGTACCTG
ATCGCGATACGGATGTGTTGCCGCCTCCACTGTCAAAACCCCAGTAGATCCACTACGTAGCTAACACCTACCGTCCATTGGTCATATACTTGGACCTTAGGACTTGTA
GCGCTTCTAGGGTATATGAGCTTAGGTTTGTACCCTTTTTGGTTGATAGGGATCCTACAACCTTAGAAAGATTTTTAACATCGTGTTGCATTGCAAGAGGATTTCCAT
TCAGCCATGACACACAACGGGATCGCCCTACAACGACATATATAGAGTATGTCCCATAGAGGACATTGTGTCAGCTAGAGTAGAGCAGACACGCCCCTACCTCACCT
GCTTGCCAAGGTTGGCAGACTTTTAGGGTGGTCTAGGTTTTTTTAACGTAATATCAGAATCTCTGTCTCTCAATTAAAAAGAAATAGAATCGACCATGTTTTAAAGA

AAACCGGACCCAAAACCTAGTTCAAAAGTTCTATGTCTCACTCTAGATATAATTGGACCAGAGTACTTTCAGTTCACCTTTGGAGGGAGGCCTAACATGTTTGTGCC
AGTCTAGTCACGGAGCGCTAGGTTGTGGTTTATAGAGACTACTACCAGGTTATACACCTTATGCTACAGCACCATGGACCCCTAACACCCTCACGGTGAATACGCTC
CAAGTATAGATAGCTCATGTATAGAGATGGCAACGGGGAATTCTCCGTCGGGGAATAACTCTCCATACACGTCACCGCGACGTAAAACATTATCCGCGGGGATCCCC
ATGAACGCTTGTGGGGACATTTCTTCCCTATCCTCGTTCCCCGCGGGGATAATCCCCGACGGGTATCTCCGCCCCGTTTAAATTATAATTAGGACATATATCCTTTGTT
ATTATTGTAAAACATGTTCACTTATACATATTGTTAGATGTAATAAATCATTATGTGTACATCAAAATGAACACATTTGTACAATGATTGATCTCTTGACAAGATAATTATT
TATTTTTATTATTAACTAGTACACGAAAATATCATCACGTGTAGGTTTAACGGATCCTCGCGGCGGACGGGGATGGGGAATGCTTCTCCATCCCATCCAAGTTTACCC
GTCGCGGGAGAGAAGAATTTTCTCCATTTATATACACGTGTGGAAAGAAACTTCTCCATCTCTGTTCCATAACTGAGGAATTCCCCACGGGGAATCAAGGATCGAG
ACGCATTGTCATCTCTACTCATGTAGATATTGTTGAATAATTAGACAAATTCCAAATTAAATTCCGAATATAAATCATGACCAAATCAGAAGAACTGAAATAAAAAGCC
AAATCAGATGATGCGTACTGATTAGACTTACTGATAGATGGCGCGCGCCGGAATCAGCAGGGTCGACGATGTCGAAGTAGCGAAATCAGCAGGGTCGACGAGGTC
AGAAGATCACGAGCAGTCGCGTGAAGACGCTTCCCAAAAACCTTATTCGCCCTCTCCCGATGCAGGATCTAGAAGACGAAGGGTTCCGGAGACCTGCTCTCCTGA
TCGCAGATGCACCTCTGCGGTCGAGACGAAGGGAACTAAAAGGCGGCTCAGCTATGAAGAGAGGCGAAAGCGAAACCGGGATGATTTGGAGGCTGGCTGCCG
GGCTCCTTTTATAGGGCCGAGTCCGCGACCCCCGAGCTTATCCATCCACAAAAATCTCGCGATCAGTTGAGATTTTATAGCGATCGGTTAGGATAAACGTAACAGCC
AAGAAACCAAAAAAATCAAACGGCAAAAGGTAGTCGCGCCCCCGCAAGGCGAGGGGCCGGATTTCGGCGGACCATTCAAGCGCATGTCGTGCGCCCGCGCCCT
TCGGCCAGGCCAGGCCAGGCCAGGCCAGGCCAGGCGAGGCGAGGCCAGGCGAGCGAGCGCGCGCGTGTGGCTCTCCACACTCTCTCCTCTCATCCATGACT
TGGTGAGTGAGTGTGGCTTCCATATTTAAGCTAGCTCTACTCCACTAGAACTAGCAATATGGTGCTATTGGTTCCACCTATTCCCCTAGCCATACACTTATATGGGCTT
TTGAGATTTTCCTCGGATTTATTTGAATTTCTTAACTGGGCCTAGCCCATAAATCCTAACAATCCCCCACCAGATCTCAAATGTCCATCTGTAGTTTTCGCCACTGTTCA
CTACTGTTTAATATACTAGTTTTTCAGCAGAGACTGTTAAGTTGAACTTCCGCCTAGAACTCCAAGCTACACCAACCCACAACTTGGACAATGGACTATGCCTTGAAT
TGCAAGTTTTGCGTGAATGGGTTTCACTTGAAGTCATGACCAGTACTTGGCTACCAGTAGTCCCCTTCTCGGGTGGAGCATATATGTTGTACTCCAAGGTCTCTTCAT
GAGTTTACTAGAGATCACCCAAATCTCATAGATTGCGACGTTAGACAGTCTAACTCATATAGGTGTGTTCTTTCAAAGAATGTTCTGCAGGACAACATCTTCGCTAAT
ACAAGCCAACAGAACACATTAAGGCACAAAGCCATCCTGCCTTACAGCATTTGAGAGTATTGCATCTTTACTTAGAGAGGGTCAAAGGTTACTCTCCTCAGTTCAC
CAATGGCTTGTTCTTCCCAGGACCTAATTCACGGGATCTCCGATCACATAGACTGGGTTTCCACCATGGCAACTTTATTCGGGTCTCATACCCATCTCTCTTGATGCG
ATTTCTATCACATTACATGGTAGTCCCTTGGTAAAAGGATCTGCCAGATTTTTATCTATTGAAATATAAGTCACACTTATAACTCCGGAGTTTCTCAACTTTCTGACAGA
CTTCAATCGTCTTTTGACATGTCTTGATGACTTTCCATTATCCTTAGAACTCGTCACTTTAGCAATCACTGTCTGATTGTCACAGTTCATAAGGATAGCTGGTATTGGTT
TCTCAACCACCGGCAAGTCCATCAAGAGTTCACGCAACCATTCTGCCTCAACGGTTGCTGTGTCAAGTGCAGCTAGCTCAGCTTCCATGGTTGACCTCGTCAAGAT
GGTCTGCTTGCATGACCTCCATGATACCGCACCTCTACCAATAGTAAAGAATAACCACTGGTGGCATAAAGCTCATCTACATCAGATATCCAGTTCGAATCACTATATC
CCTCAAGTACTGCATGCTGACCAGAATAGTGAATTCCATAACTCATTGTACCTTGCAGGTAGCGCATAACCCGCTCAAGTGCATGTCAATGATCAGTCCCGGGGTTT

GACATGAACCTACTCAATTTGCTCACAGCAAACGAGATATCGGGCCTTGTTGCACCAGCAAGATACATGAGTGAACCAACAATCTGAGAGTATCTCAATTGGTCTAA
ACCGATTCTCTTGTTCTTTCGCAGTGTCACACTGGGATCATAAGGTGTTGGAGAAGGTTTGCACTCAGAGAAGCCAAATTGCTTCAAAACCTTTTCAACATAGTGA
GATTGCGAGAGAGTAATCCCACCATCTGCCTTAATCAGCTTGATGTTTAGAATCACATCAGCTTCTCACAGATCTTTCATATCAAAACTCTTTGATAGAAAAGACTTG
ACTTCATTGATCACATCAATGTTTGTGCCAAATATCAATATATCATCAACATATAAGCACAATATAACTCCTTCGCCCCCACCACAGCGATAATATACACACCTGTCTGCC
TCATTAATGGCAAAGCCTGCAGACGTTAGAGTCGTGTCAAACTTCTCATGCCACTGCTTTGGTGCTTGCTTCAGACCATACAAAGATTTCAATAACTTGCACACCTT
GCTTTCTTGACCCTTTACTACAAATCCATCAGGTTGTTCCATATAGATTTCCTCGTCCAGCTCTCCATTAAGAAAAGCTGTCTTTACATCCATCTGATGAACAAGGAGA
CCATACGAGGCAGCCAAAGAAAGTAGTACTCGAATAGTAGTCATTCTAGCAACAGGTGAGTAAGTATCAAAGAAGTCTTCTCCTTCTTTCTGAGTATAGCCTTTAGC
CACAAGCCTAGCCTTGTACTTTTCAATTGTACCATCAGGCTTGAGCTTCTTTTTAAACACCCACTTACAACCCACAGGTTTGCATCCATAGGGTCGATCAGTGACTTC
TCACGTACCATTTGAAAGAATGGAGTCCATCTCATTATGAACTGCTTCTTTCCAATCATCTGCATCTGGAGATGCAAATGCTTCTGCAATGGTAGTAGGAGTATCGTC
CACAAGGTACACAATGAAATCATCACCAAAGGATTTTTCAACCCTTTGTCTCTTGCTCCTTTTAGGAGCATCATTGTCATCCTCCTCTAGGACAATTTCATGTGGCTGT
TCAAAACTCTCAATAGGTGTACTATGTTCAGGAGTTATCTCAGAAGAATATCTAGAATTGCTATGAATGTCTTTCATTGGAAATATATGTTCAAAGAAAGTAGCATCAC
GTGATTCCATAATAGTATCAACATACACATCAGGAACTTCAGATTTAACTAGTAAAAATCTATATGCTATGCTACACGAAGCATATCCAAGAAAGACACAATCCACTAT
CCTTGGACCAAGCTTGCGCTTTTTATTAATTGGTACATTGACTTTCGCCATGCACCCCCAAGTGCGCAAGTATGAAAGTGATGGTTTTCTCCCAACCCACTTCTCATA
AGGGGTTTTCTCTTCTTTGCCCATAGGAATTCTATTCAGAACATGACATGAAGTCAGGACTGCCTCCCCCCACCATGCCTTAGATAAACCACAAGTGTCTAACATGG
CATTCACCAGGTCAGTCAACGTACGGTTTTTCCTTTCAGCAATCCCGTTTGACTCGGGTGAATAGGGAGGAGTCCTCTCATGAATAATGCCATGTTCTGCACAGAAA
TCATCAAAGACTTTGGGAAAGAACTTGCCACCATGATCTGATCTAAGACGTTTGATCTTTCTCTCTAGTTGGTTTTCAACCTCAGCCTTATAGATTTTAAAGTAGTCTA
AAGCCTCATCTTTAGTTTTTAGCAAATATACATAGCAAAATCTAGACGCATCATCAATCAATGTCATGAAGTATCTCTTACCACCCTTTGTCAACACACCATTCATCTCA
CAAAGATCAGAATGTATGAGTTCTAGCGGTGCCAGGTGTCTCTCCTCAGCAGCCTTATGAGGCTTTCGAGGTTGCTTCGACTGCACACAACTATGGCACTTAGAAC
CTTTGACTATGGTGATATTCGAAATTAAACTCATGGTTGCAAGCCGAGACATAGAGCCAAAATTAATATGACACAAACGAGAATGCCAAATACTCGCAAGATCATCA
ACATTAGCACAAATATGGTTCACAGACTTATTATTGAAATCTAACAAAGAAAAGCGGAACAAGCCTCCGCAATCATAGCCTTTACCAATAAATTGTCCAGACTTGATG
GACACAACTAATTTATTGGACTCCAAAACTACCTTGAACCCATCTCTACATAGAAGGGTTCCGCTAACGAGATTCTTGTGTATAGAAGGGACATGATGCACGTTCTTC
AGCTGCACGATCTTTCCCAAAGTAAACTTCAGATCCACTGTGCCAGTGCCATGAACAGAAGCATGTGACCCATTCCCCATTAGCACGGAAGAATCCTGGGCGCCCT
GATAAGAAGAGAACAAGTTAATGTCAGAATACACATGAACATTAGCACCAGTATCAAGCCACCAACTAGGTGATTGAAATACTAAGAAGATGAAAGGTAAATTACC
ATACCCTTTGTCTTACTCATTGCTAGCGACCACTGTGTTGACATTGCCCTTTTTGCCACGGCGATCCGCTCGATCGGGACAATCCTTGGCAAAATGACCCGCCTCGCC
ACATGTGAAACATGTCAATTTAGCCTTGTTCTTCTTCTTTTTGAAGTTGGTAGTTTTGTTGGGCTTGTTAGATTTTGGCTTTCCTTTCCCCTTGTTGTGGTTCCTTTGA
ACCATGTTGGCGCTGGAGTGGCCCTCGCCTCCTTTAGATCTTGTGTCCTTAGCCCGAGCTTTCTCCTCAACATCCAGAGACGCTATCAGATTTTCAACTGATATCTCC

TGTCTCTTATGTTTCAGAGATGTGGCGAAGTTCCTCCATGTAGAAGGCAACTTTGCAATAATGCACCTAGCCACAAATTGGTCAGGAAGGACTATCTTAAGGTGGTC
GAGCTCCTTGGCTATACACTGTATTTCATGAGCTTGCTCTACAATAGAGCGATTATCAACCATCTTATAATTATGAAAGCTCTCCATGATATACAGGTCACTGCCAGCAT
CTGATGCACCATACTTAGTAGTAAGTGCATCCCACAACTCTTTCCCGTCTGTGTACTGAATATTCGCATCAACCAGACGGTCAACAAGGGCGCTAAGAACGGCTCCC
GTAAAGATAGTATTGGCATGGTCGTACTCTTTCTCCTGTTCAGGAGTCAGTGGACCCTTAGGTCTGCCTTTACTAATATGGAAGACATTCATAGCAGTAAGCCAGAG
CGTGGCCTTGACTTGCCATCTCTTAAAGTGCATACCATTGAACTTTTCTGGCTTGAGCGCATCGGCAAAAGCAGCCATAGAAAAACTAGGAAATTGTCTACAATAAG
GTTTTTGGATTGTTGAATAATTAGACAAATTCCAAATTAAATTCCGAATATAAATCATGACCAAATCAGAAGAACTGAAATAAAAAACCAAATCAGATGATGCGTACT
GATTAGACTTACTGATAGATGGCGCGCGCCGGAATCAGCAGGGTCGACGATGTCAGAAGATCACGAGCAGTCGCGTGAAGACGCTTCCCAAAACCTTATTCGCCC
TCTCCCGGTGCAGGATCTAGAAGACGAAGGGTTCCGGAGACCTGCTCTCCTGATCGCAGATGCACCTCTGCGGTCGGAGGAACGAAGGGAACTAAAAGGCTGG
CTATACGAGCATGTCGTGCGCCCGCGCCTTTCGCCCCGCCCCGCCCCGCCGAGCGAGCGCACGCGTGTGGCTCTCCACACTCTCTCCTCTCATCCATGACTTGATGA
GTGAATGTGGCTTCCATATTTAAGCTAGCTCTACTCCACTAGAACTAGCAATATGGTATTATTGGTTCCACCTATTCCCCTAGTCATACACTTATATGGGCTTTTGAGAT
TTTCCTAGGATTTATTTGAATTTCTTAACTGGGCCTAGCCTATAAATCCTAACAGATATTGTCTTCTAACCCCGTTCGGTTGTGTGTATGTGGCCAGTCTCCTAACAGAT
ATTGGTTACTTGTATGGGTGAGCCTGGCTCGTATCTAGTGAGCCTGGCTCAGACTGGACTGGCTGAGTGCATGCAGTATAGCGTGTTTGGTTGTGTGTATGTGGCC
AGTCTGGTTCACAAGAGATGTTGTTTGGTTGCTTGCATGAGTACATGGTGCATGAGTTAAAAAAATTGTTAAATAAATTAAAAAATCTATTAATAGACACTAATTATAT
GGTAAATTGCTTAATAGATACTAATTACATGTTAATATACACTAATTATATATTAATCTTAATCACGTAATTTTTTTATTTGAAATATATATAGTATGAGTGGTCTAGTTTCG
TTCGCAAAAATGTCAGGAACGCGGATATGAAATCGGTTCGGTGATTAAATCTTTTAATTAGACGATATAATGAAAAAACCAATTAAAAACGTGTCTCGATATGCGTGC
ACGAGTAAAGAGCTGGCTGAGCGGAGCCTGGCCGCGCGCGTGCGGCCGCGCGATGCGTTTCTGCCGGGCCTGGCTCGCGGCGCTTTAAGTTCCGTGCTAGCCTA
TCTCGTGCGATACAACGAACCAAACAAACACTACTTTTTGCATGTGTAGATGCAGCTGCACCACCGGAGAAGGCAACCAAACGCCCCCCAAGTGACTCGACGCTA
GTACGTCATCTTCTAACTGATGAGGAGTCACCAAACACCAAAATTATAAAGAACACCGACAACAAATCTTATGATACCAAAGCTATACATATATATTTCCAAAAACTGA
TTGCCAAACACAGAAGTACTTGATTTTGGACAAATTTGGAATGACAACTACTATATATGAAATGCAGCGAGTAACTAGAAGTTCACAATGATGGGATCCTAAAAATA
AATAATTTTATGAGCTTTAAAATTTATCCAAAAAACAACATTCTATAAATTCATGGGACACTATAATGCGGAGGCTAATCACAGGTGCATACAATATTAACTACTAAATC
TAGGCGATATCCTCCGGAAATGAAAAGAAGATTGAGGTACATGCATGCGTCCTTTTCCAGGCCTCTTTAATCTCTCCTAAAATTTTCACGAAATTTATTTATTTTGGG
ACGGTGGAATTACATATGTACTTCAACAAATGGCGTGAACCTCTCTAGTCATTAATGGTTCGCGTGATTCCTGTGTGGTGGCCCAATTTAAGAGCCAGACGTACATAT
ATAGAAGGTTTCAATAAACAATTGATTACATCCTAAAATAACCAAGCAGAAAATGTAACTGCGAGAGAGAACTAATTATTTTCCAAACAACTAGTCCCTGATCTACAC
ATCCCGATCAATATATAAGTATGGCCGCGCCGGAACAGGATTACAGAAACCTAGCACACCCAAACACACCAACGTCCAAACATAGGAGCAAGTGAGCAAGCAACC
ATTGGAGGCACTTAGCTTGTGGTGCTCATAGCTAGGAATCATACATGGGCAGTAGTAGAGCTCTGTTCTTGGCAACCGCCATGGCCGTGGCCATGGCCATGGTCTT
CTCACCCTACTACCTTGCCGTGCAAGGGCTAAAAGGTCGTGCATACATTATTAATCCCTTAATTTATATTCAATATCAACTTTGCAAAGGATGTCTATATATAACGTCTTC

CTTAGTTTTGTCGTAGTGCAGTAAACTGTTTCGACTTGCATGGAGGGAAATGCGACAAAGAGACGTGCACCATGGTTTGCAAGGCGACCGGGTACGTCGACCCTG
TCGTGAAATGCTCCAACCCCCCTGGGAAAAGCGGCCAGTGCTGTTGCTTTGTGAAGTGCTGCGGGGGAGATGCTTCGGTTCCAAGCCTGCTGTGAGACTCGTAC
ATATTGGTAGCTAGCTATATACGCTGCTTGCGTGATCTGAATAAATGGTTGATCTGAATAAATGGTTCCTAATAAATCTTTGACTATTGTTTCTTCTCGTTAATTATATATG
TACAGAACGTCAACAAATTTTGTAATGCTATTTAAGTTCATTTACGAATCTTGTCGAAGTCATTCCACATATCAGATGTGAAAGCTCTATTTCTTACCCGAGAACTAGT
AGGCGTGTCCTCTTGTCGAAGTCATTCCACATATCAGATGTGGAAGCTCTATTTCTTACCCGAGAACTAGTATGTGTGTCTGTCTCCTCGACCTCTGCCACATCAGGG
TCGGAATCTATAATGATAGGATCAGTTGAAGGAATTGCAACCTCACTGCCACCGATTTGGCCAGCCACGGTGTCGTCATCGGCGACCATCAAGCACGTACAATCAC
AAGCACAATATTAACAACTTATCAAAGTCGGAGCTCCAGATCGATCCCTCACGACTATAAACACAGAACAAGAAGGCACTAGATTAGGGTTCGAACTCAGATCACA
AGTACATATCATAAGCACAAGCACCAAATTAAGAACTTACCAAAGTTGGAGCACCATATCGGTCCCCTCACGACTACAAACACAGAACAAGAAGGCACTAGATTAG
GGTTCTAACACATATCACAAGCACAAGCAAAAGATTAGCAACTTACCAAAGTCATAGCACCAGATCGGTCCCTCACGGCTACAAACACATAACAAGAAGGCACTAG
ATTAGGGAGCACAAACACAGATCACAAGCACAAGCACAAGATTAGCAACTTACCAAAGTTGGAGCACAAGATCGATTCCTCACGGCTACGAACACAGAACAAAA
GGCACTAGATTAGGGTTCGCCGGTTCGAACACAGATCTAGAGGCGCCGAGGGAGGCGGTGCTTTCACAATCGCTAGAGGGTTCGGGAGAGACCGAGTGAGAG
AGGAGCTTGAGAGAGAGAGAGAATGAGAGAGGAGGCGGGGACGGTGTGGGGGGCGGAGGTCGCGGGGCTATGTGTTTATGGATGTTGTGCATAGTCGTGTTT
CTGACCTAATCGGGTCATGCCCATGTTGTCCCATTGTGCCCCCTCGCCGCCCCAAGCACGTCACTAGGGGTCGGACTGTGCCGGCACGGACCCGCCCACTGTGTCG
TGCCCCGTGTCAGTCCGGTTAACCAGACACTATTGACCATCTATAGCACCAGTTAAGTAGCAATTATAAGATTAATTTGTCGATTTTTTATTAGGCCAGAAAGAGTATA
TTGAGAAACAAACTACTTCTACTCAAATATTAGAGTGTTTGGTTCTATAGACTAAAGTTTAGTATATGTCACATCGGATATTGGAATGCCAAGTACGAGCATTAGATAT
AATCAAATTACCAAACTACTCCCTCTGTCCGGATGACTTAGACGTTGTAGGTTTTTAAGACATATTAAGGAAGAAGCAAAATGACACATGTAACCCTGCTTTGTTTTG
ATTAAGCGTTGATTACAACAAAAAAAAGGTAATTAATACTCAACGCCTTGATTACAACAAAACTAGGTTGCGACGAGTGATTTGGCCGGTGGTGGTGGGAATATGA
GATGCGAACTGAACGGATAAGGATAGAGATGACACCTTCACGCTGTGTGAACAAAGAAGCTGGGTTGCGGGGTGCGTCAAAGTGCATGGACAAGTTAAGTGGG
TTAAAACGCCTAAGTCATTCGAACGGAGGGGAGCAAACACTCTAGTATTACCAAACTAGTAATTACATAAATGACGAAACACATTTGTAAGCATTATTTTAATTTAGG
ATCAGTAAAATGTTTACTGTAACATTACTTGAACGAATTATAAAGTAATTTGGTTTAATATATTCATCTTATCGTTTAATTCTTATCTCTGTAATTAATTTTATAATTAGAAA
ATATATAATACTTTTAACTAACATATCTCTACTATACTTAAAGCAACAGTTTTGTCGGCGTTTCGAGACCGGGGGGTCCCTGGGCCGACGAGTGAGTGTCGCCGCGT
GCCCCAGCCCAGATGGGTCGAGCGCGAGGGCGAGCGCGAAGGGGGAGAGCGAGGCGACCGGAGACCGGCGCGAGAGAGGTGGGAATCCCGCGGCCTTCGT
GTTCGTCCCGCGCCCAGGTCGGATGCGCTTGCAGTAGGGGGTTACAAGCGTCCACGCGGGAGAGGGAAGCGAGTTGCTCCAAGCGAGCGCCTGTCTCGTCCTC
GTCCCCGCGCGCCCAACCTTCTCTAAGAGGGCCCTGGTCCTTCCTTTTATAGGCGTAAGGAGAGGATCCAGGTGTACAATGGGGGGTGTAGCAGAGTGCTACGTG
TCTAGCGGAGGAGAGCTAGCGCCCTAAGTACATGCCGTTGTGGCAGCCGGAGAGATTTTGGCACCCAGCTGGTGTGATGTCGTGGCCGTCGGAGGAGCGATGG
AGCCTGGCGGAGGGACAGCTGTCGGAGCGGTTGGGTCCTTGCTGACGTCCTCTTGCTTCCGTAAGGGGGCTGAGAGCCGCCGTCGTCACAGAGTATGCGGGGC

ACCATCATTGCCTATCTGGCGGAGCTAGCCAGATGGGACGCCGGTCTTGTTCCCTGCGGCCCGAGTCAGCTCGGGGTAGGGTGATGATGGCACCTCCTGTTGACG
TGACTGGCCTGCGCCCTAGGTTGGGCGATGTGGAGGCTCCTCCGAAGCCGAGGTCGAGTCTGTCTTCCGTGGCCGAGGCCGAGTCCGAGCCCCTGGGTCGGGC
GAGGCGGAGGTCGTCGGCTGAAGCCAGGGCGAAGTCCGAGCCCTGGGGTCGGGCGAAGCGGAGTTCGTCGTCTTCTGGGACTTAGCCCGAGTCCGAGCCCTG
GGTCGGCGGAGCGGAGTTCGCCGTCTTCCGGGACTTAGCCCGAGTCCGAGCCCTGGGTCGGCGGAGCGGAGTTCGCCGTCTTCCGGGACTTAGCCCGAGTCCG
AGCCCTGGGTCGGGCGGAGCGGAGTTCGCTGTCTTCCGGGACTTAGCCCGAGTCCGAGCCCTGGGTCGGCGGAGCGGAGTTCGCCGTCTTCCGGGACTTAGCC
CGAGTCCGAGCCCTGGGTTGGGCGGAGCGGAGTTCGTCGTCTTCTGGGACTTAGCCCGAGTCCGAGCCCTGGGTCGGGCGGAGCGGAGTTCGCCGTCTTCCGG
GACTTAGCCCGAGTCCGAGCCCTGGGTTGGGCGGAGCGGAGTTTCCTGTGGCGCCTTCGGCAGGGCCTGACTGCCTGTCAGCCTCACTCTGTCAAGTGGCACAG
CAGTCGGTGTGGCGCAGGCGGCGCTGTCCTTCTGTCAGGCCGGTCAGTGGAGCGGTGAAGTGACGGCGGTCACTTCGGCTCTGCCGGGGGGCGCGCGTCAGG
ATAAAGGTGTCAGGCCACCTTTGCATTAAATGCTCCTGCGATTTGGTCGGTCGGTGCGGCGATTTTGTCAGGGTTGCTTCTTAGCGAAGGTAGGGCCTCGGGCGA
GCCAGAAATATGTTCGCCGTCGGAGGGGGGCCTCGGGCGAGACGGAAATCCTCCGGGGTCGGCTGCCCTTGTCCGAGGCTAGGCTCGGGCGAGGCATGATCGA
GTCGCTCGAATGGACTGATCCCTGACTTAATCGCACCCATCAGGCCTTAGCAGCTTTATGCTGATGGGGGTTACCAGCTGAGAATTAGGAGTCTTGAGGGTACCCC
TAATTATGGTCCCCGACAGTAGCCCCCGAGCCTCGAAGGGAGTGTTAGCACTCGCTTGGAGGCTTTCGTCGCACTTTTTTGCAAGGGGACCAGCCTTTCTCGGTT
GCATTTTGTTCCGGTGAGTGCACGCGAGCGCACCCGCCGGGTGTAGCCCCCGAGGCCTCGGAGGAGTGGTTTCACTCCTTCGAGGTCTTAATGCCTCGCGTAATG
CTTCGGCTGGTCTGGTTGTTCCCTCATGCGAGCTGGCCGTAGCCCGGGTGTACGGTCGGGTCCCAAGTTCTCGGGCTGATATGTTGACGTTGTCAACGGTTCGGCC
GGAGCCGGGTTTGCGAGAGCAGCCCCCGAGCCTCTGCACAGGGCGAGAGGGCGATCAGGGACAGACTCGGCTTTTTTACATACGCCCCTGCGTCGCCTTTTCGC
AAGGAGGAGGGGGGAAAGCGCCATGTTGCCCTCGATGGGCGCCGAACATGGTGTCTCCGGTGAGCTGCAAGCGGGTAATCCGAGTGGACGTCTGTGCCCCGTT
CGTTAGGGGTCGGCTAGGGGCCCAGAGGCACGCCCAAAAGTACCAGCGGGTGATCTGCCGGACCCGGTCCCCTGGCGACGGGGTCCGAGGGCTCGATGCCTCC
CTCTGATGGGATTCCGTTACAAGATCGTTCCCGCTGGTCTCGGAAATGTCCTAGGGTACCTCGGGAGCGCAGCTCGAGCCTTGGTTATGTATCGAACGTACCCATG
GTCATCCCTCGCTCGGCGTCTGAGGCGGCTGTGAACCCTTCGGGGGCCAGCCTTCGAACCCCTGATCAGTAATGGGCGCGGAGCCCGAGTAGCCTGAGGCGGCC
GTGGAACCCTTCCGAGGGGCCGGCCTTCGAACCTCTGACCAGTAGTGGGTGTAGGGCCCACGCGATCTGAAGCGGCTGTCGAACCCTTCCGGGGGGCCACCTTC
GAACCTCTGATCAGTAGGGAGGCTCGGAGCCTGGTTCCTTCATGGGGAAGGATCCTTTTCGGGGTATCCCCCTTTCCTGGTCCCTGTTGCAAGAGAGAGAAAGAG
GAAAAAAGGAAAAGGATACGAAATCGAACGACGCGGCGTACCTTTACTGACGCGGTCATTATGGCGAAGGCGAAGCGTCGCCCGCTTCTCCTGCCAGAGGCGCC
GCCTGTCCCGCCGCGGAGTTAATGCGACGGAGCGAGTGGTTGGCGGGGCGGCCGTTGCGCGTGCGCGAGCCGTTCGAGGAATGGATCACGGGCGCGTTGTCTT
CATGCCGTGAGAGGGGGTTCTCTCGCTGCCCCCGGATGGGACGTGAGCCTGGCTGACGACGTGACCGCTGCTCCTGCCCGCCTGCCACCGTCATTACTGCCGGCC
CATTTTTGGCCGCACTGACCGCCGCGCCAGGCTGGCGCTGCTGGGTCGTGCGCTGGGTCGCCTCGAGTCGCGGTATTGGTTCCACAATCGAGGAGGCGCGGTGG
TGGCGCAAGTGGCGGTGCAGCTGCATGCACGATGCGTTCGGCGCGCCGGTTGCATGACGCGTGGGCCTGGGCCTCCAGGCTGGGCGTGTTGGGAGTCGGAGA

AGGGCGTCCACTTGGCGCGGTTGCATGCCGCCTGCATGGCTGTCCGCCCCTTTCGCCCGCTGGTCTGGGCAAAAGTGGAGGGTCACTTGTAACCGCTGGGTGGT
CGTGCGCACCGCGCGCGGCGGTTTGGCTTCTTCTGCTCTGAGCCGGCTTGCATGACATGTGGGACCCAGCCCCTGCGCCGCAGGGGAGGACCTCAGAGTGTGTT
GGAGAAGACTCAGCCCGCGACGGTTGGGGGTGCAAGTAGGGAGAGTCGCCTTTAAAAGGAGGGTGACCCCCTTCGGAAGGCGACCATGTCTTCGCGCTCCCTT
ATGCATCGTGTCTTTCCACCTTCCAAGCCTTCGGATGGGGGATATCCGCCGTTTTTCCGCCCTGTCGTTGGGGGGAACGCAACTCCGCGGGAGTTGGTACCTTTCA
GCCATCGTTCGGCTTCAAGGATTTTCATCACACAGCCCGGTTGCACCCCTCCGCTGGTGGTCACCCAAGACGGTGACCTCCAGCTCCTGGATGGGGAGGGGCAA
GCCGGGCTGTGATCTCGATCCCGCCCTCAGCGTCGAGGGTGTTCGTCATCCTCGCTGGGGCGGGGAGCGAGGCGAGCCGGGGCTCTACCTCCTACGCGGGTCGG
CGGGCCACCTCTTCTTCCAGCTTCTGGTGGTGGCAATCGCCCTCCAGCTCTGTGGCGGAGACGTCCTCCAGCCATGCCGGGGAAGGCGAACTGTTGCTGCCCAGC
TAGGATGCAACATTCCGCCCTCTTCCTTGCATTCGCGGCGAGGACGACAGCGAGGATCTGCCGGTGCGCTTGGGAGCGGCCCGCTCTTTGGCTTTAATAGCTGGT
TCACGTCCCTTGAGCGGGAACGCGAACGAGAGCCCTCCGGCGGCCGCGTTCACCCGGGACCATGGCTGCTGCTGCAGAGGTCGCTGGCGGGTCTCCCGATGTTC
GTCGCCCCGCAGGCTCGAGGTTGCTCATACCCGCGGGGACGGAACCAGAGTTCCGTTTGTACTGGCACTTTGAATGCCAGTGTTGTTGTTCATTGTGGCTGTCGA
GGCCTGAACATGTATGTAATTTTGGCACGGAGCCATGTTTTTCCTCATTTTTGAGCACTAAGACTCGCCTGTTGGTTGTCTGAACCGCTTCACCAAGCGTGAGTCGC
CCCGTGTCAAGGTGACGAGTGAGGTATCCGTATCCCGGAGGTGTAGGAGTCCCTCGGCTCGGTCGGCCTTGTTGCCCGAGGCTCCTCTAGCTTAGTTAAAGGGAC
CCCTTGGCCGCTCTTGGATGAGCCGAGGCCAGGGGTAGCGATATCAGCATGAACAGGGGCAGAGTCGGCTCAAAAATGAAACTTGGTTGGTCGGAGCCTAGCC
GGGTCGTCCGTTGGCGGGACCGACGCTGGAGTTGACCAGCCGACGCCTCGGGTCGGGCTGGCGCCCTTGGTAGAATGCTGGCCGAGGCCCCGGGGCGAACGG
CCGAGCCGCTCGCTCGGGCCGGGTTCCTGGAGGGGACCCTGGCAGCGATTGCCCGGGCGTGGCGATGGCGTCGTCCTTCAGAGTGGAGATCCTCGGACCGCGT
CGCCGTCCGAGGCTAGGTCGGACCTTGCCGATGGTGTCGTCGATGCCGAGGGTGCTGCTGCCCCCTTCCAGCGTCAAGACCTGAGCCTGCAGGATCAGATTGTCT
TGTAGCGTGTGTCTCCTGCGGCCGCTGAGGCCAGAACACACCCTTGCTGTGTTGTAAAGCTGCGTCCCTTTTCCTCTTGTTTCGAGTATCTGGACTTTTTTGTCGGT
AACAGGGATGTTTGTGCGAGCGAGAGTTGCTTCTCGCGGAAGGTGATGAGTGAGGTATCCATATCCCGGAGGCGTAGGAGTCCCTCGGCTCGGTCGGCCTTGCC
GCTTACGCGCACTTTTGCCCGTCCATGAGGCTCTGTCACCGACTCAGTCGAGAAGGCTCGAAGGATCGCTTTGGCAGAAGAACTTCCGAACGTGAAGACTTGTTC
GGTCCGCGGAATCACTTATTCGAACGTGAGTTACTTATCGCAGAAGGTGATGAGTGAGGTATCCGTATCCCGGAGGCGTAGGAGTCCCTCGGCTCGGTCAGCCTT
GGCTGCTTACGTGTACTCCGTCGTTTTCAGGATCCACTTTTCGAAGTAGTCAAAAAGCACGAAAGACATTCTGGCAGAAGAGATCTTTTTTCGAGGAAAATTTCAA
CGCAGAGGGGGTTCCCCCCCTTTTAGCCCCCGAGGGAGGGTCGGGNNNNNNNNNNNNNNNNNNNNNNNNNNNNNNNNNNNNNNNNNNNNNNNN
NNNNNNNNNNNNNNNNNNNNNNNNNNNNNNNNNNNNNNNNNNNNNNNNNNNNNCCGACCCTTCCTTGATGACTAAACTTTGCGTGGGTGCGAGGTATATG
AACAACTTGAAAACATCTTAAGGGTAGAAGCGACGTAGCTGTTGGATGTTCCAAGCGTTGCCGTAGACCTTGCCTTGGCTGTTGGCCAGCTTGTACGTTCCGGGC
TTCAGAACCTTGGCGATGACGAACGGTCCCTCCCAGGGGGGCGTGAGCTTGTGCCTCCCTCGGGGGTCTTGTCACAGCCGAAGCACCAGGTCGCCCACCTGGAG
GTCTCGGGACCGGACCCCTCGGGCGTGGTAGCGTCGCAGGGACTGCTGGTACCGCGCCGAGTGTAGTAAGGCCTTGTCCCGAGCCTCTTCCAGCGAGTCTTCTC

GGCTAGCTTGGTTGCTTTGATCGCTGTAGGCCCTCGTCCTCGGGGAGCCGTATTCCAGGTCAGTGGGCAAGACGGCCTCGGCCCCGTAGACTAGGAAGAACGGC
GTGAAACTCGTGGCTCGGCTCGGCGTTGTCCTCAGGCTCCAGACCATCGAGGGGAGCTCCTTCATCCATCGCTTGCCGAACCTGTTGAGGTCGTTGTAGATCCGA
GGCTTGAGCCCTTGTAGAATCATGCCGTTGGCACACTCTACTTGCCCATTTGACATGGGATGAGCCACGGCGGCCCAGTCCACCTGGATGTGGTGATCCTCGTAGA
AGTCCAAGAACTTTCTGCCGGTGAACTGGGTGCCGTTGTCGGTGATGATGGAGTTTGGGACCCCGAAGCGATGGATGATGTTGGTGAAGAACGCCACCGCCTGC
TCGGACCTGATGCTGTTCAGAGGTCGGACCTCGATCCACTTGGAGAATTTGTCGATGGCGACCAGCAGGTGCGTGTAGCCCCCGGGTGCCTTCTGCAAGGGGCC
GACGAGGTCCAGACCCCACACAGCAAAGGGTCAGGTGATGGGTATCGTCTGCAGAGCCTGAGCGGGCAGGTGGGTCTGCTTCGCATAGAATTGACACCCTTCGC
AGGTGCGGACAATTCTAGTGGCGTCGGCCACCGCCGTCGGCCAGTAGAAGCCCTGTCGGAAGGCCTTCCCGACAAGGGCTCGAGGCGCTGCGTGATGGCCGCA
AGCCCCCGAGTGTATCTCCCGCAGGAGTTCCTGACCTTCGGCGACGGAGATGCATTGCTGGAGGATGCCTGAGGGGCTGCGGTGGTAGAGCTCCTTTTCTTCGCC
CAGCAAGACGAACAACTTGGCACGGCGCGCCACCCGCAGAGCCTCGGCTCGGTCGAGGGGTAGCTCTCCTTGGTGGAGATATCGCAGGTACGGGGTCTGCCAG
TCTCGATCAGGCGTGGCCCCGCTTCGCTCCTCCTCGACGCACAGTGCCTCACCCTCGGGGGCCGAGGGTACCTCGGGCTGAACCGAGGGCGCCTCGACCCGAGC
TGAGGGTGCCTCGGGCTGTGCCGAGGGTACCTCGGGTTGGGCCGAGGGCGCCTCAGGCTCGGGCGTGTCGTCGATCTTGACGGAGGGTTGATGCAGATCCCGG
GAGAAGACGTCTGGGGGAACCGTTGTTCGCCCCGAGGCTATTTAGCCAGCTCGTCCGCAGTCTCGTTGTAGCGCCGAGCGATGTGGTTAAGCTCGAGCCCGTAG
AACTTGTCTTCCAGGCGCCGAACCTCATCGCAGTAGGCCTCCATCTTCGGATCGCGGCAGTGGGAGTTCTTCATGACTTGGTCGATGACGAGCTGCGAGTCACCG
CGGGCGTTGAGGCGTCGGACCCCTAGCTCGATGGCAATCCGCAACCCGTTGACCAGAGCCTCATACTCAGCCACATTGTTGGATGCCGGGAAATGGAGGCGTAG
CACATAGCGTAGGTGTTTCCCGAGGGGCGAGATGAAGAGTAGGCCCGCGCCGGCTCCCGTCTTCATCAGCGACCCGTCGAAAAACATGGTCCAGAGCTCCGGTT
GGATCGGAGCCGTCGGTAGCTGGGTGTCGACCCATTCGGCCACGAAGTCCGCCAAAACCTGGGACTTGATGGCCTTCCGAGGGGCGAACGAGATTGTCTCGCCC
ATGATTTCCACTGCCCACTTTGCAATTCTACCCGAGGCCTCTCAGCACTGGATGATCTCCCCCAGGGGGAAGGATGACACCACAGTTACCGGATGAGACTCGAAGT
AGTGTCGCAACTTCCGCCGCGTCAGGATCACTGCATACAACAGCTTCTGAACTTGTGGGTAGCGGATCTTGGTTTCGGACAGTACCTCGCTGACGAAGTAAACTG
GCCTCTGAACGGGCAATGCGTGCCCCTCTTCTTGCCTCTCGACCACAATCGCGGCGCTAACCACCTGAGTGGTCGCGGCGACGTAGACCAAGAGGGCTTCTCCGT
CAGCTGGAGGCACCAAGATAGGCGCCTTTGTGAGGAGCGCCTTTAAGTTCCTAAGAGCTTCCTCGGCCTCAGGGGTCCAAGTGAAGCACTCGGCCTTCCTTAAG
AGGCGGTACAGAGGCAGACCTCTTTCGCCGAGGCGTGAGATGAAGCGGCTCAGAGCCGCGAGACATCCCATGACCCTCTGTACGCCCTTCAAGTCCTTGATGGG
CCCCATGCTGGTGATGGCTGCGATCTTCTCCGGGTTGGCTTCGATGCCCCGCTCGGAGACGATGAACCCCAAGAGCATGCCTCGGGGCACCCCGAAGACACACTT
CTCAGGATTGAGCTTGACGCCTTTCGCCTTGAGACATCGGAATGTCACTTCAAGGTTGGAAAAGGAGGTCGGAAGCTTTCCTCGTCTTGACCACGATGTCATCAA
CGTAGGCCTCGACCGTGCGGCCAATGTGTTCGCCGAACACATGGTTCATGCACCGCTGGTACGTCGCGCCCGCATTCCTCAAGCCGAACGGCATGGTGACATAGC
AGTACATGCCGAAGGGCGTGATGAAAGAAGTCGCGAGCTGGTCGGACTCTTTCATCCTGATTTGATGATACCCTGAGTAGGCATCGAGGAAAGACAGGGTTTCAC
ACCCAGCAGTGGAATCCACGATTTGATCAATGCAAGGCAGAGGGTAGGGAACCTTCGGACATGCTTTGTTGAGACCAGTGTAGTCTACACACATCCGCCATTTCCC

CCTTTCTTTCTCACAAGCACAGGGTTGGCAAGCCATTCGGGATGGAATACCTCTTTGATGAACCCTGCCGCCATTAGCTTGTGTATCTCCTCGCCTATGGCTTTGCGC
TTCTCTTCGTCGAATCGGCGCATAGGCTGCTTGACGGGTCGGGCTCCGGCCCGAATATCCAGCGAGTGCTCAGCGACATCCCTCGGTATGTCGGGCATATCTGAGG
GACTCCACGCGAAGACGTCGGCGTTCGCGCGGAGAAAGTCAACGAGCACTGCTTCCTATTTGGGATCGAGCCCGGAGCCGATCCGGATCTGCTTGGAGGCGTCG
CCGCTGGGGTCGAGGGGGACGGCCTTAACCGTCTCCGCTGGCTCGAAGTTGCCGGCATGACGCTTCACGTCTGGCACCTCCTTAGAGAGGCTTTCCAGGTCGGC
GATGAGGGCCTCGGCGTACTCCACGCACTCCACGTCGCATTCGAACGCGTGTTTGTACGTGGGGCCGACGGTGATGACCCCGTTGGGGCCCGCCATCTTGAGCTT
CAGGTAGGTGTAGTTGGGGACGACCATGAACTTCACGTAGCATGGCCTTCCCAGTACCGCGTGGTAGGTTCCTCGGAACCCGACCACTTCGAACGTCAGAGTCTC
CCTTCGGAAGTTGGAGGGCGTTCCGAAGCAGACGGGAAGGCCGAGTCGTCCGAGGGGCTGGACGCGCTTCCCGGGAATGATCCCATGGAAGGGCGCAACGCC
TGCTCGGACGGAGGACAGATCAACACGCAGGAGCCTGAGGGTCTCGGCGTAGATGATGTTGAGGCTGTTGCCTCCGTCCATAAGGACCTTGGTGAGCCTGACGT
CGCCGATGACGGGGTCGACGACGAGCAGGTATTTCCCCGGGCTCGGCACGTGGTCGGGGTGGTCGGCTTGGTCGAAGGTGATGGGCTTGTCGGACCAGTCTAG
GTAGACTGGCGCCGCCACCTTCACCGAGCAGACCTCCCGGCGCTCTTGCTTGCGATGCCGAGCCGAGGCATTCGCCGCTTGCCCACCGTAGATCATGAAGCAGTC
GCGGACCTCGGGGAACTCTCCTGCTTGGTGATCTTCCTTCTTGTCGTCGTCGCGGGCCCTGCCACCCTCCGCGGGTGGCCCGGCCCTGTGGAAGTGGCGCTGAA
GCATGACGCACTCCTCAAGGGTGTGCTTGACGGGCCCCTGATGATAGGGGCACGGCTCCTTGAGCATCTTGTCGAAGAGGTTGGCACCTCCGGGGGGTTTCCGA
GGGTTCTTGTACTCGGCGGCGGCGACAAGGTCCGCGTCGGTGGCGTCGCGTTTCGCTTGCGACTTCTTCTTGCCTTTCTTCTTGGCGCCGCGCTGAGTTGACGCC
TCGGGAGCATCTTCCGACGGGCGGCCCTGGGGCTGCTTGTCCTTTCGGAAGATAGCCTCGACCGCCTCCTGGCCGGAGGCGAACTTGGTGGCGATGTCCATCAG
CTCGCTCGCTCTGGTGGGGGTCTTGCGACCCAACTTGCTCACCAGGTCGCGGCAGGTGGTGCCGGCGAGGAACGCGCCGATAACATCCGAGTCGGTGATGTTGG
GCAGCTCGGTGCGCTGCTTCGAGAATCGCCGGATGTAGTCCCGGAGAGACTCTCCCGGCTGCTGCCGGCAGCTTCGGAGGTCCCAGGAATTCCCGGGGCGCAC
GTACGTGCCATGGAAATTGCTGGCGAAAGCTTGGACTAGGTCGTCCCAGTTGGAGATCTGCCCTGGAGGCAGGTGCTCCAACCAGGCGCGAGCGGTGTCGGAG
AGGAACAGGGGGAGGTTGCGGATGATGAGGTTGTCATCGTCCGTTCCACCCAGTTGGCAGGCCAGGCGTAGTCCGCGAGCCACAGTTCCGGTCTCGTCTCCCCC
GAGTACTTTGTGATAGTAGTCGGGGGTCAGAACCGGGTCGGGAACGGTGCCCGTCGGATGGCCCAACTGAAGGCCTGCGGACCGGGCGGTTCGGGCGAAGGA
CTCCGATCCTCCCCACTGTCGTAGTGTCCCCCACACCTGGGGTGGTAGCCTCAGCGCACCCTCTCGTCGAGGTGGGCCCGACGGTCGTGATGATGGTGCTCGTTGC
CGAGGCGACCCGGGGCCGCAGGCGCGGTGTTGCGCGTGCGCCCGGTGTAGACCGAGGCTTCCCGCATGAATCAGGAAGTCGCGGCATGAGGTTCCGAGGGGT
ACCCCTGCCTTCGGGAGGCAGAGCTCTCGGCCCGTCGGACCGCGGCGCCTTCCAGGAGATTCTTGAGCTCCCCCTGGATTCACCGACCCTCGGTGGTTGATGGCT
CCGGCATCGCGCGGAGAAGCATCGCTGCTGCAGCCAGGTTCTGGCCGACCCCACTAGATGCGGGTGGTGGCCTGACCCTGACGTCGTCGGCGACGCGGTGCTG
GAAACCCTGGGGCAGATGACGTATTTCTCCGGCCGGGGGTTGGCCCGCCCATGCCTGCCCGACGTCCCGGCGGATCGGCTCAAGCGCTCCTGCTCCCTCGTCGA
GCCTGGCCTGCACTTCGCGGATTTGCTCGAGCTGTGGGTCATGGCCCCCCGCCTGAACGGGGACCACAGCTAGCTCCCGTGGGATGTCAACGCGGGGCACCGGC
CTAGGGAGATCACCGTCCTTCGGCATGCCGAGATGATTGCCTTCGGAGGGACCCCCTAGATCGACGTGGAAACATTCGCGGCTTGGGCCGCAGTCCTCATCGTCG

AGGCCGCGGCTACCGTCGGAACAGTCGGAGAGGCAGTAGTCACATGCGGTCATGAAGTCCCGCATGGCACTGGGGTCGCCGAGTCCAGAGAAACCCCAACAGA
TGCTGGGTTCGTCATCTTCCTCGGACCCGGAGGGCCCGTAGGTCGAGACGTCCGTCAACCGGTCCCAAGGCGACCGCATGCGAAACCCCAGAGGGTTTGGACTC
GCCTCTACGAGAGCGCCCGCCAAAGCGAGGTCGCTAGGCGGGTTGAGGCTGAATCCAAATGACGTGGGATGGGAATCGGTCGGTACCTCTTGGTCGACGAGTG
GAGATAAAGTCACGTCGGGGACTGACTGCACCGTCGTCTCAGGTACGAGGGCGACGTCCAGCAAGCTTTTTGCAAGCGTGCTGGCGTCGTCCGCTTGCTCGGGA
TTGGCGTGTCGCGGGGAGACGGCGCTCGTCTTCGTCTCAAGCGCGAAGTCGATACCCGGTGCGCCCCCCGTTGGGGTGCCGGCGCCGTCGACTTGCTCGACAGC
CGATGAGGCGCTGCCTCCTGCTTGGCCTTGGCTGCCCTGCCTTCCCCTCCGTCGGCGGGGAAGAGGGCAGGATGAGCTCGAAGGTTGTTCTTCCACCACGCGGG
GAAGACGTCGTCGATTCCGCCGCCAGCGGGCGGGCTGTCGGCCGCCATTGTCGTTGTCGCGCGGCGGTGGAAGGAGTATCATGTCGTAGCTGCCGTCGAAGGAC
ATGAACTCAAGACTCCCGAAACGGAGCACCGTCCCGGGTTGGAGAGGTTGCTGGAGACTACCCATCTGGAGCTTGACGGGAAGCTGTTCGTCAACACGCAGCA
GGCCCCTATCTGGCGCGCCAACTGTCGGCGTTTCGAGACCGGGGGGTCCCTGGGCCGACGAGTGAGTGTCGCCGCGTGCCCCAGCCCAGATGGGTCGAGTGCA
AGGGCGAGCGCGAAGGGGGGAGAGCGAGGCGGCCGGAGACCGGCGTGAGAGAGGTGGGAATCCCGCGGCCTTCGTGTTCGTCCCGCGCCCAGGTCGGGTG
CGCTTGCAGTAGGGGGTTACAAGCGTCCACGCGGGAGAGGGAAGCGAGTGGCTCCAAGCGAGCGCCTGTCTCGTCCTCGTCCCCGCGCGGCCAACCTTCTCTAA
GAGGGCCCTGGTCCTTCCTTTTATAGGCGTAAGGAGAGGATCCAGGTGTACAATGGGGGGTGTAGCAGAGTGCTACGTGTCTAGCGGAAGAGAGCTAGCGCCCT
AAGTACATGCCGTTGTGGCAGTCGTAGAGATTTTGCCACCCAGCTGGTGTGATGTCGTGGCCGTCGGAGGAGCGATGGAGCCTGGCGGAGGGACAGCTGTCGG
AGCGGTTGGGTCCTTGCTGACGTCCTCTTGCTTCCGTAAGGGGGCTGAGAGCCGCCGTCGTCACAGAGTATGCGGGGCGCCATCATTGCCTATCTGGCGGAGCTA
GCCAGATGGGACGCCGGTCTTGTTCCCTGCGGCCCGAGTCAGCTCGGGGTAGGGTGATGATGGCGCCTCCTGTTGACGTGACTGGCCTGCGCCCTAGGTTGGGC
GATGTGGAGCCTCCTCCGAATCCGAGGTCGAGTCTGTCTTCCGTGGCCGAGGCCGAGACCGAGGCCCTGGGTCGGGCGAGGCGGAGGTCGTCGGCTGAAGCCA
GGGCGGAGTCCGAGCCCTGGGGTCGGGCGAAGCGGAGTTCGTCGTCCTCTGGGACTTAGCACGAGTCCGAGCCCTGGGTCGGGCGGAGCGGAGTTCGCCGTC
TTCCGGGACTTAGCCCGAGTCCGAGCCCTGGGTCGGGCGGAGCGGAGTTCGCCGTCTTCCGGGACTTAGCCCGAGTCCGAGCCCTGGGTCGGGCGGAGCGGA
GTTCGCCGTCTTCCGGGACTTAGCCCGAGTCCGAGCCCTGGGTTGGGCGGAGCGGAGTTCGTCGTCTTCTGGGACTTAGCCCGAGTCCGAGCCCTAGGTCGGGC
GGAGCGGAGTTCGCCGTCTTCCGGGACTTAGCCCGAGTCCGAGCCCTGGGTTGGGCGGAGCGGAGTTTCCTGTGGCGCCTTCGGCAGGGCCTGACTGTCTGTC
AGCCTCACTCTGTCAAGTGGCACAGCAGTCGGTGTGGCGCAGGCGGCGCTGTCCTTCTGTCAGGCCGGTCAGTGGAGCGGCGAAGTGACGGTGGTCACTTCGG
CTCTGCCGGGGGGCGCGCGTCAGGATAAAGGTGTCAGGCCACCTTTGCATTAAATGCTCCTGCGATTTGGTCGGTCGGTGCGGCGATTTTGTCAGGGTTGCTTCT
TAGCGAAGGCAGGGCCTCGGGCGAGCCGGAAATATGTTCGCCGTCGGAGGGGGGCCTCGGGCGAGACGGAAATCCTCCGGGGTCGGCTGCCCTTGTCCGAGG
CTAGGCTCGGGCGAGGCGTGATCGAGTCGCTCGAATGGACTGATCCCTGACTTAATCGCACCCATCAGGCCTTAGCAGCTTTATGCTGATGGGGGTTACCAGCTGA
GAATTAGGAGTCTTGAGGGTACCCCTAATTATGGTCCCCGACAAGTTTCAACGGCCGTCATATGTCATATTTTTACGAATAATCTCTCACATCTATCTCAAATTAACCAT
GCATCTCTGCATGCAACCTCCACGTCCGGCCCGTTAGCCAGAATATTAAAAAAAGGTGCAGAAGATGGGGTTTGAACCTAACCCTGATGGAAAAAGGACAGGAG

ACACTAAGTGAAGTCGTCTAACCAATAAAACATCACGGTCAATTGTTTTTAATATTGAATGAATTGTACATATGTATATACGAGTTTTATAAAATAAAAAATATATAATTG
TGCTGGATCGGATCAGTACTACGGGCCGAGGCTACGACCCAAACACGACACGACGATCGTGTCAGGCTGGCCCAGACAATATTAAATGGGTCGTGCCCCGGACCA
GCCCGCTAGACACAGCCTATTTGGTCATTTATACCTCCACACGATAATGATGATCCTCACCTCACTTGTCGTCACCTCGTTCGTCATCCTGCTTCTTTTCTCTCTCCTCT
CATGCTTCCGCCTCCGCGCCACCCATTCTTGGCAGAGGGCGTGGGAGCGGGCACCTCCTCCTCGTATTCGTCCGACACCAGCCCCAACACCGACCCGTGCAGTGG
CTATTGCACGTCCACGAGGGTGTTTCACAGCATGCACGCACTATCGTTTTCGTCGTCGTCGGACGTCCCGTTTGTCTTCCCGGCCTTCGCCCTGTTCTTCCTCCCCAA
CATGCTGCCCCTGCCCACGGTCACAGCCGCGAGCCGACCGACGCTCGTCGACGCGGGTATGTGCGAGTCGGTCTTGCTCTTGTCTTTTCTTCGTGCGTGCCGCCG
GGGAGGACACGATGGTGCCTGCCACGCTTATGTTATTTTGGCGATGACGAGTCCAGATCTGAGGTATTAGACAATCAAGGCCTGTAGCGCGGCAACAGTCGGCAT
ATGCTACGGAGTTGGTGGTGGTGTTGTGTCGCCTCGGCGGGTCTAGGGCTAGAAGGCACTCGCATTCTCTCGCCCTTTTCAGGCTGACGCCTGGTACGGGTGCCT
CTCGGTTTGTAGCTGCGCTGGCTGGGCTTCTTTCTTCGCTTGCGTGCTCTCTCCCTGTATGTATCTAGCGGTATACTACCTATGTCGCCTCCAGATGTCAATGTCTTCG
TCGTGTCCTTCTCCGGCAACGAACATGTATGACTGTCTCTTCTCTTCCATTCCTGCTCTTGTGGGGGAGGCGAGGGATGGTGATCCCTGATTTGCTGTCTATGATACC
AATTCGATAGCTCGGCATCGTCTATCTACATGATATTTCCTTTACCACGATGTCGACTCGGTTAGTCAAATAATAAAAATATTATGCGGAGTGCAGAGACAATCAATAA
AAAATCTTGAGATCTTTTTGGTAAATAGTTTACGTGGTTGTTATTGTGAACCGTCGTAACGTATGGGTAACGGACCAGTAAACATTTAATACGATAGAGTAGGTAAGC
AAACAATACGGGCACTTTGAGGACGATAGCCCGCAGCGACCGACGCTTGTCACCTGCACGGGTACGCTGCGGAGCTTGCTGGCGTCGCCGTGACCGCGAATAAA
AAGAAAGCGCCCCGCCACGTAACGTCCTACCGTGCACCACGAAGATAAGCCACCAGTCCAGCCTGCGGGCCCCACGAGCTCCCTCGCCTCCCGCACGTCCCATCG
CCACGTGCACGCCCTCCAGGATCGCAGTGGCCGGCTGGCCGCAGCCCGCTTGGGCCCCACCGCCTGGCGCGTTTTTAGGGCATGTACAGTGGAGAGACACCAAA
ACGGTTCTCCAAGCACAGGAGACAACTAAGAGACTCTATTGTACAATGGAGTGTCTATAAACATAGTCTATTAATAAATACAGAATTAAATGTATTTGTATAGCATCAG
ATCGATAGAACAGACGACAAATTCGTACAGTGGGAAGTAAGGCGTCTGTTGTTACTTGGTTTACGAGCCTGAGGCGTCTCTTCACGGAGAGACGGCTCTAAGATT
TTTTTGCAAATAACCCCCTAAAACACCTTAAGAGCCCCCACATTAAACACCACTGTACACGCCTTTAGGCCCGCACACGCGCCCTGGCCCGCGCCGGGCCCACCCT
GCCCTTCGCAAGCCGCACGATGTGACAGAGCGCGCATCCCTGCCGTCCACGCGCCCGCGCGACGCTGGCCACGAGTTCCCGTGCGGCGATCGGACGGTGGCGG
TGGCGATGGCGAGGCCGGAGCGCGGGCAGAACGGACGGCACGGGATCGCGTCGCGATGAGGGTCACGGCAGAGGAAATGGCGGCCACAGGTAACACAGGTT
CGCCGTTCGGCGTTCGCCGGCGCCCTGGAAAAAGCGCAGGCCAGGTGGGTCCTGCGTGCAGCCACGCGCAGGCCACTCCGCGTGTTCCCAACCGCAAATTATTC
ATTATTGTCAGTGACGTTTTATTTTTTTCAGGAGGGTCCCTGCTGAAGCCTGGATTTGCCTGTGGCTACCGTGGATGTCCGTTTTCTGGCTGCATTTTACTGTTCACA
GAACAGCTTTGATTAATTGGGCGTGCTGTTTAATATCTATCTATTTATTTATTCTTGAGAATGAAATGCTCCGAGAGAGGGAACATGAATTTCCGTATTTTGTTTACCA
ACCAGCACAACTATTTAAATTTGAAAAATATAGAAATTATTTTTAAAATTTTAAGATACACTATGCAACAAGCATATGTTACTATTGCATATATTCTAAGTTACATAAAAA
TCCTGCATATATTCTATGATACATAAAAAAAATATGAATCCTATGTATTAAGTATAGATTATATATTCAAGTCCGTGACATGAAATACAACAACAACAACAAAGTCTTTTTG
TCCCAAGCACGTTGGTTAGGCTAGAGATGAAACCTCATATGAAAACCTTAGAGCTCAACCCCCAAAGAAAAGAAAAGGGAGACAAAGGCAAAGGAGAAACAAA

AAACGACGAAACGGGGAAACACATAAAAAGAGATAAAACCCACAAGAACGAGCAAGATCTAAAGGGGCACAAGAAAAGGTCAAACGATTAAGGAGGAAAAGC
GAAACTACAAATCAGGGTTCTGGCACGTGAATTGCACACTTCCACTCCTTCCTATTCACGGCGAGCTCTTTATCAATATTCCACTCCTTCAGGTCCCTCTTCACAGCC
TCTGTCCACGTCAAGGTTGGTCGACCTCTACCTCCCTTCACATTCTCGGGACACCTAATTATTCCGATATGCACTGGTGCCTCTTCAGGTCTTCGTTGGATATGTCTAA
ACCATCTCAAACGATGTTGCATAAGCTTCTCCTCAATTGGCGCCACTCCTACTCTATCTCTCGTATATCATCATTCCAGACTCGATCTCTCCTTGTGTGGCCACATATCCA
GCGCAACATACGCATCTCTGCCACACTTAGTTGTTGGACATGCCGTCTTTTAGTGGGCCAACATTCTGCTCCATACAACATAGCCGGCCGGATTGTTGTCCTGTAGA
ATTTGCCTTTTAGTTTGTGTGGCACCCGGGGGTCGCATAAGACACCCATAGCTTGTCGCCACTTCAACCATCCAGCTTTAATTCTATGACTGGCATCCTCATCGATGT
CTCCCTCCTTATGAAGCATTGATCCTAAGTAACGAAAAGTGTCTTTCTTGGGTACCACTTGCCCATCAAGACTAACATCGCCATCCTCATACCCCATGGCACTGAAATC
ACACTTCATATACTCTGTTTTAGACCTACTAAGCCTAAAACCTTTTGCTTCCAGCGTCTGCCTCCACAATTCCAACTTTTGTGAAATACCACTCCTACTCTCCTCAATAA
GTACCACATTGAAAGGGAATTAGGCTTACACCTAGTTCCTAAATAATTTTGGTGGTTGAATTGCCCAACACAAATAATTGGACTAACTAGTTTGCTCTAATGTATAAG
TTATACAGGTGTAAAAGGTTCACACTTAGCCAATAAAAAGATCAAATATTGGAGTCAACAAAGGAGCAAAGGGACAACCGAAGGCACCTCTGGTCTAGGGCACCG
GACTGTCCGGTGTACAGAGGACTCCAACTCAAACTTGCCACCTTCGGGAATTTCCAGAGGCACTCCGCTATAATTCACCGGACTGTCCGGTGTACACTGGACAGTG
TCCGGTGCTCCAAGGAAGAGCGGCCTCCGGAACTCGCCAGCCTCGGGAAAACGCAGCGACTGCTCCGCTATAATTCACCGGACATGTCCGGTGTACACCGGACT
GTCCGGTGAACCAGCAGAGCAACGACTACTTCGCGCCAACGGTCACCTGCAGGCGCATTCAATGCGCACCAGAAGCGCGCAGAAGTCAGGCACGTCCATACCGG
CGCACCGGACATTGAACAGTACATGTCCGGTGCGCCACCGGACATCCAGGCGGGCCCAGAAGTCAGAACTCCAACGGTCGAACTGCAACGGCTTGGTGACGTG
GCTGGCGCACCGGACATATCCGGTGTACACCGGACTGTCCGGTGCACCATACGACAGACAGCCTCCACCAACGGTCATGTTTGGTGGTTGNNNNNNNNNNNNN
NNNNNNNNNNNNNNNNNNNNNNNNNNNNNNNNNNNNNNNNNNNNNNNNNNNNNNNNNNNNNNNNNNNNNNNNNNNNNNNNNNNNNNNNNNNNNNNNNNCAC
CCCACCATTCATTGCATCCAAGTTTTCCAGCTTCCAACCACTATACAAGAGCTAGCATTCATTGCAAAGCACACCAAAAGAGATCAAATCCTCTCCCAACTCCACACA
AAGCTTTAGTGACTAGAGAGAGTGATTTGCAGTGTTCATTTGAGCTCTTACGCTTGGATCGCTTCCTTTCTTTGGCATTCTTTCTTGAGATCATACTCACTTGTAACT
GAGGCAAGAGACACCAATTGTGTGGTGGTCCTTGCGGGAAGTTTGATTCCCAAGTGATTTGAGAAGAGAAGCTCACTCGGTCCGAGGGACCGTTTGAGAGAGG
GAAGGGTTGAAAGAGACCCGGCCTTTATGGTCTCCTCAACGGGGAGTAGGTTTGCAAGAACCGAACCTCGGTAAAACAAATCATTGTGTCACACTCCTTATTTGC
TTGTGATTTGTTTTGCACCCTCTCTCGCGGACTCGATTTTAACGCTAACCCGGCTTGTAGTTGTGTTTATATTTGTAAATTTCAGTTTCGCCCTATTCACCCCCCCTCTA
GGCGACTATCAATTGGTATCAGAGCCCGGTGCTTCATTAGAGCCTAACCGCTCGAAGTGATGTCGGGAGATCACACCAAGAGGGAGATGGAGACCGGCGACAAG
CCCACTACGAGCCAAGGGAGCACTTCATCGGAAGAGTCCCGCACCAAGAGGAAGGAGAAGAAGAAGGACTCCTCCAAACGGAAGGAGAAAAGATCTTCTTCCT
CACATCACAAAGAGAAGAAGGAAAAATCTTCTTCCCACAAGTCGCATCGGAAAGGCGACAAGCACAAGAGGATGAGGAAGGTGGTCTACTACGAGACTGACAC
TTCATCAACATCGACCTCCGACTCTGATGCGCCGTCCGTAACTTCTAAGCGCCAAGAGCGCAAGAAGTTTAGTAAGATCCCCTTACACTATCCTCGTACATCTAGACA
TACTCCATTACTTTCCGTTCCATTAGGCAAACCGCCAACTTTTGATGGCGAAGATTATGCTAGGTGGAGTGATTTAATGAAATTTCATCTAACCTCACTCCACAAAAG

TATATGGAATGTTGTTGAGTTTGGAGCACAGGTACCATCCGTAGGGGATGAAGACTACGATACGGACGAAGTGGCCCAAATCGAGCACTTCAACTCTCAAGCTACA
ACCATACTCCTCGCCTCTCTAAGCAAGGAGGAGTACAACAAGGTGCAAGGGTTGAAGAATGCAAAGGAGATTTGGGACCTACTCAAGACCGCGCACGAGGGTG
ATGAACTCACCAAGATCACCAAGCGGGAAACGATCGAGGGGGAGCTCGGTCGCTTCCGTCTTCGCCAAGGGGAGGAGCCACAAGATATGTACAACCGGCTCAA
AACCTTGGTGAACCAAGTGCGCAACCTCGGGAGCAAGAAATGGGATGACCACGAGGTGGTTAAGGTTATTTTGAGATCACTTATCTTCCTTAACCCCACTCAAGTT
CAATTAATTCGTGGTAATCCTAGATATACATTAATGACCCCCGAGGAAGTTATCGGGAATTTTGTGAGCTTTGAATGTATGATCAAGGGCTCAAAGAAGATCAACGA
GCTTGATGATCCCTCCACGTCCGAAGCACAACCGGTGGCTTTCAAGGCGACGGAGGAGAAGAAGAAGGAGTCTACACCAAGTAGACAACCAATCGACGCTTCAA
AGCTCGACAACGAGGAGATGGCTTTAATCATCAAAAGCTTTCGCCAAATCCTCAAGCAACGGAAGGGGAAGGATTACAAATCCCGTTCCAAGAAGGTTTGCTACA
AGTGTGGTAAGCCCGGTCACTTTATTGCTAAATGTCCATTATCAAGTGACAGTGACAGGGATAACGACAAGAAGGGCAAGAGGAGAGAAAAGAAGAGGTACCAC
AAGAAGAGGGGCGGCGACGCCCACGTGTGCCGCGAGTGGGACTCCGACGAGAGCTCCACCGACTCCTCCTCCGACGAGGACGCCGCCAACATCGCCGTCACCA
AGGGACTCCTCTTCCCCAACGTCGGCCACAAGTGCCTCATGGCAAAGGACGGCAAAAAGAAGAAGGTTAAATCTAAATCCTCCACTAGATATGAGTCTTCTAGTGA
TGATAATGCTAGTGATGAGGAAGATAATTTGCGTACCCTTTTTGCCAACCTTAACAAGGAACAAAAGGAAAAATTAAATGAATTAATTAGTGCTATTCATGAAAAGG
ATGACCTTTTGGATTCTCAAGAGGACTTCCTAATTAAGGAAAATAAGAAACATGTTAAGGTTAAAAATGCTTATGCTCTAGAAATTGAGAAATGTGAAAAATTATCTA
GTGAGCTAAGCACTTGCCATGAGACAATAGATAACCTTAGAAATGAAAATGCTAATTTGTTAGCTAAGGTTGATTCTCATGTTTGTAATGTTTCAATTACCAATTCTAG
AAATAATAATGATGATTTACTTGCTAGGATTGAAGAATTGAACATTTCTCTTGCTAGCCTTAGGATTGAAAATGAAACATTGCTTGCTAAGGCTAAAGATTTTGATGT
TTGCAATGCTACTATTTCTGACCTTAGAACTAAGAATGATATGCTACATGCTAAGGTTGTAGAATTAAAATCTTGCAAACCCTCTACATCTACTATTGAGCACACTTCTA
TTTGTACTAGATGTAGAGATGTTGATATTAATGCTATTCATGATCAGATGTCTTTAATTAAACAACAAAATGATCACATAGCAAAATTAGATGCTAAAATTGCCGAGCAT
AACTTAGAGAATGAAAAATTTAAATTTGCTCGTAGCATGCTTTATAATGGGAGACGCCCTGGCATCAAGGATGACATTGGCTTCCAAAGGGGAGACAATGTCAAAC
TTAATGCCCCTCCTAAGAAACTGTCCAACTTTGTTAAGGGCAAGGCTCCCATGCCTCAGGATAACGAGGGTTACATTTTATACCCTACCGGTTATCCCGAGGACAAA
ATTAGGAGAATTCATTCTAGGAAGTCTCACTCTGGCCCAAATCATGCTTTTATGTATAAGGGTGAGACATCTAGTTCTAGGCAACCAACCCATGCTAAGTTTCCTAAA
AGGAAAACTCCTAGTGCATGAAATGAACATAGCATTTCATTTAAGACTTTTGATACATCATATGTTTTAACTAACAAATCCGGCAAAGTAGTTGTCAAATATGTTGGG
GGCAAGCACAAGGGGTCAAAGACTTGTGTTTGGGTACCCAAAGTTCTTGTGTCTAATGCCAAAGGACCCAAAACCATTTGGGTACCTAAAGTCAAGAACTAAAAT
TGTTTTGTAGGTTTATGCATCCAGGGGCTCAAGTTGGATACTCGACAGCGGGTGCACAAACCATATGACAGGGGAGAAAAGGATGTTCTCCTCCTATGAGAAAAA
CCAGGATCCCCAACGAGCTATCACATTCGGGGATGGAAATCAAGGTTTGGTCAAAGGTCTTGGTAAAATAGCTGTATCTCCTGACCATTCTATTTCCAAAGTTTTTCT
TGTAGATTCATTAGATTACAATTTGCTTTCTGTATCTCAATTATGCAAAATGGGCTACAACTGTCTCTTCACTGATGTAGGTGTCACTGTCTTTAGAAGAAGTGACGAT
TCAATAGCATTTAAGGGAGTGTTAGAGGGTCAGCTATACTTGGTAGATTTTGATAGAGCTGAACTCGACACTTGCTTAATTGCTAAGACTAACATGGGATGGCTCTG
GCATCGCCGTCTATCACATGTTGGAATGAAGAATCTTCATAAGCTTCTAAAGGTAGAACACATTTTAGGACTAACAAATGTTCATTTTGAGAAAGACAGGGTTTGTA

GCGCATGTCAAGCCGGGAAGCAAGTTGGCACTCATCATCCACACAAGAACATCATGACTAGTGACAGGCCACTGGAGCTTCTACACATGGACCTATTCGGCTCGAT
CGCTTACATAAGCATCGGCGGGAGTAAGTACTGTCTAGTTATTGTGGATGATTATTCTCGCTTCACTTGGGTGTTCTTTTTGCAGGAAAAATCTCATACCCAAGAGAC
CTTAAAGGGATTCTTGAGACGGGCTCAAAATGAGTTCGGCTCAAGGATCAAGAAAATTAGAAGCGACAATGGGACGGAGTTCAAGAACTCTCAAATTGAAGGCT
TTCTTGAGGAAGAGGGCATCAAGCATGAGTTCTCTTCTCCCTACACTCCACAACAAAATGGTGTAGTGGAGAGGAAGAATCGAACTCTCTTGGACATGGCAAGAA
CCATGCTTGATGAATACAAGACACCGGATCGGTTTTGGGCCGAGGCGGTCAACACCGCCTGCTACGCCATCAACCGGTTATATCTACACCGAATCCTCAAGAAGAC
ATCCTATGAACTCCTAATCGGTAAAAAGCCAAACATTTCATATTTTAGAGTTTTTGGTAGCAAATGTTTTATTCTTGTCAAAAGAGGTAGAAAATCTAAATTTGCTCCT
AAAACTGTAGAAGGCTTTTTACTAGGATATGACTCAAACACAAGGGCATATAGAGTCTTTAACAAGTCCTCAGGACTTGTTGAAGTTTCTTGTGACGTTGTGTTTGA
TGAGACTAACGGCTCTCAAGTAGAGCAAGTTGATCTTGATGAGATAGGTGAAGAACAGGCTCCATGCATCGCGCTAAGGAACATGTCCATTGGGGATGTGTGTCC
TAAGGAATCCGAAGAGCCTCCAAATGCACAAGATCAACCATCCTCCTCCACGCAAGCATCTCCACCAACTCAAAATGAGGATGAGGCTCAAGTTGATGAAGTAGA
AGATCAAGCAAATGAGCCACCTCAAGATGACGGCAATGATCAAGGGGGAGATGCAAATGATCAAGACAAGGAGGATGAAGAACAAAGGCTGCCACACCCAAGA
GTCCACCAAGCAATCCAACGAGATCACCCCGTCGACACCATCCTCGGCGACATTCATAAGGGGGTAACTACTCGATCTCGTGTTGCACATTTTTGTGAGCATTACTC
TTTTGTTTCCTCTATTGAGCCACACAGGGTAGAGGAAGCACTACAAGATTCGGATTGGGTGATGGCGATGCAAGAGGAGCTCAACAACTTCACGAGGAACGAGG
TATGGCATTTAGTTCCACGTCCTAACAAAATGTTGTAGGAACCAAGTGGGTCTTCCGCAACAAGCAAGACGAGCATGGTGTGGTGACAAGGAACAAAGCTCGACT
CGTGGCCAAGGGGTATTCACAAGTCGAAGGTTTGGATTTTGGTGAAACCTATGCACCCGTAGCTAGGCTTGAGTCAATTCGTATATTATTGGCCTATGCTACTTACC
ATGGCTTCAAGCTTTACCAAATGGACGTGAAGAGTGCCTTCCTCAACGGACCAATCAAGGAGGAGGTCTATGTTGAGCAACCTCCCGGCTTTGAAGACAGTGAGT
ACCCTAACCATGTCTACAAGCTCTCTAAGGCGCTTTATGGGCTCAAGCAAGCCCCAAGAGCATGGTATGAATGCCTTAGAGATTTCCTTATTGCTAATGGCTTCAAA
GTCGGAAAGGCCGATCCTACTTTATTCACTAAAACTCTTGAGAATGATTTGTTTGTATGCCAAATTTATGTTGATGATATTATATTTGGGTCTACTAACGAATCTACATG
TGAAGAATTTAGTAGGATCATGACACAAAAATTCGAGATGTCCATGATGGGGGAGTTGAAGTATTTTCTAGGATTTCAAGTGTAGCAACTCCAAGAGGGCACCTTC
ATTAGCCAAACGAAGTACACTCAAGACATTCTAAACAAGTTTGGGATGAAGGATGCCAAGCCCATCAAGGCACCCATGGGAACTAATGGGCATCTCGACCTCGAC
ACGGGAGGTAAGTCCGTGGATCAAAAGGTATACCGGTCGATGATAGGTTCTTTACTCTATTTATGTGCATCTCGACCGGATATTATGCTTTCCGTATGCATGTGTGCA
AGATTCCAAGCCGACCCTAAGGAAGCTCACCTTACGGCCGTAAAACGAATCTTAAGATATTTGGCGTACACTCCTAAGTTTGGGCTTTGGTATCCTAGGGGATCCAC
TTTTGATTTAATTGGTTTTTCGGATGTCGATTGGGCGGGGTGCAAAATCAATAGAAAGAGCACATCGGGGACTTGCCAGTTCTTGGGAAGATCCTTGGTGTCTTGG
GCTTCAAAGAAGCAAAATTCGGTCGCTCTTTCCACCGCCGAAGCCGAGTACATTGCCGCAGGCCATTGTTGCGCGCAATTGCTTTGGATGAGGCAAACCCTGTGG
GACTACGGTTACAAATTAACCAAAGTCCCTTTGCTATGTGATAATGAGAGTGCAATCAAAATGGCGGATAATCCCGTCGAGCATAGCCGCACTAAACACATAGCCAT
TCGGTATCACTTTTTAAGGGATCACCAACAAAAGGGAGATATCGAGATTTCATATATTAACACTAAAGATCAATTAGCCGATATCTTTACCAAGCCTCTTGATGAACA
AACCTTTAACAAACTTAGGCATGAGCTAAATATTCTTGATTCTAGAAACTTCTTTTGTTGACTTGCACATATAGCTCATTTATATACCTTTGATCATGTCTCTTTCATATG

CTATGACTAATGTGTGTCCAAGTCCATTTCAAACCAAGTCATAGGTGTATTGAAAGGGAATTGGAGTCTTCGGCAAAGACAAAGGCTTCCACTCCGTAACTCATCCT
TCGCCGTCGCTCCAAGTCACTCTCCATCTTTAAGGGAGAAAGAAAAAGGACTTCGTCTTTGGTATAATCTTAACTCATTTATTTATGACCAAAGGGGAAGAAAGTAC
TTCGAGGGCTCTAATGATTCCGTTTTTGGCGATTCATGCCAAAGGGGGAGAAAGTATGAGCCCAAAGCAAAAGGACCGCACCACCACCAATTTCAAAAGATTAGT
TTTTCAAAGAGTATTTTCAATTGGTATCTTATTGTGTTCAAAAGGGGGAGAAAGTAGTATTTCAAAAAATGATATATCAAAACCCTCTTGAACACTAAGAGGTGGATC
TCATTTAGGGGGAGCTTTGTTTAGTCAAAGGAAAAGCATTTGAAACAGGGGGAGAAAATTTCAAATCTTGAAAATGCTTCGTAAAATCTTACTCATTCACCTTTGA
CTATTTGCTAAAGAACTTTTAAAAGGATTTACAAAAGATTTTGCAAAACATGTGGTGCAAGCGTGGTCCAAAATGTTAAATAAGAAAGAAACGATCCATGCATATCT
TGTAAGTATTTATATTGGCTCAATTCCAAGCAACATTTGCACTTACATTATGCAAACTAGTTCATTTATGCACATTTATATTTGCTTTGGTTTGTGTTGGCATCAATCACC
AAAAAGGGGGAGATTGAAAGGGAATTAGGCTTACACCTAGTTCCTAAATAATTTTGGTGGTTGAATTGCCCAACACAAATAATTGGACTAACTAGTTTGCTCTAGT
GTATAAGTTATACAGGTGTAAAAGGTTCACACTTAGCCAATAAAAAGATCAAATATTGGAGTCAACAAAGGAGCAAAGGGACAACCGAAGGCACCTCTGGTCTAG
GGCACCGGACTGTCCGGTGTACACCGGACAGTGTCCGGTGCACCAGAGGACTCCAACTCAAACTTGCCACCTTCGGGAATTTCCAGAGGCACTCCGCTATAATTC
ACCGGACTGTCCGGTGTACACCAGACAGTGTCCAGTGCTCCAAGGAAGAGCGGCCTCCGGAACTCGCCAGCCTCGGGAAAACGCAACGGCTGCTCCGCTATAAT
TCACCGGACATGTCCGGTGTACACCGGACTGTCCGGTGAACCAGCAGAGCAACGGCTACTTCGCGCCAACGGTCACCTGCAGGCGCATTCAATGCGCGCCAGAA
GCGCGCAGAAGTCAGGCACGTCCATACCGGCACACCGGACATTGAACAGTACATGTCCGGTGCGCCACCGGACATCCAGGCGGGCCCAGAAGTCAGAACTCCAA
CGGTCGAACTGCAACGGCTTGGTGACGTGGCTGGCGCACCGGACATGTCCGGTGTACACCGGACTGTTCGGTGCACCATACGACAGACAGCCTCCACCAATGGT
CATGTTTGGTGGTTNNNNNNNNNNNNNNNNNNNNNNNNNNNNNNNNNNNNNNNNNNNNNNNNNNNNNNNNNNNNNNNNNNNNNNNNNNNNNNNNNNNNNN
NNNNNNNNNNNNNNNNNNNNNNCACCCCACCATTCATTGCATCCAAGTTTTCCAGCTTCCAACCACTATACAAGAGCTAGCATTCATTGCAAAGCACACCAAA
AGAGATCAAATCCTCTCCCAACTCCACACAAAGCTTTAGTGACTAGAGAGAGTGATTTGCAGTGTTCATTTGAGCTCTTGCGCTTGGATCGCTTCCTTTCTTTGGCA
TTCTTTCTTGAGATCATACTCACTTGTAATTGAGGCAAGAGACACCAATTGTGTGGTGGTCCTTGCGGGAAGTTTGATTCCCAAGTGATTTGAGAAGAGAAGCTCA
CTCGGTCCGAGGGACCGTTTGAGAGAGGGAAGGGTTGAAAGAGACCCGGCCTTTGTGGTCTCCTCAACGGGGAGTAGGTTCGCAAGAACCGAACCTCGGTAA
AACAAATCATTGTGTCACACTCCTTATTTGCTTGTGATTTGTTTTGCACCCTCTCTCACGGACTCGATTTTAACGCTAACCCGGCTTGTAGTTGTGTTTATATTTGTAAA
TTTCAGTTTCACCCTATTCACCCCCCCTCTAGGCGACTATCACACATCATCGGCAAAAAACATTATCGGGGACCATAATTAGGGGTACCCTCATTACTCCTAATTCTCA
GCTGGTAACCCCCATCAGCACAAAGCTGCAAAGGCCTGATGGGTGCGATTAAGTCAGGGATCGATCCGTTCGAGGGACTCGATCGCGCCTCGCCCGAGCCTAGCC
TCGGGCAAGGGCAGCCGACCCCGGAGGATCTCCGCCTCGCCCGAGACCCCCCTCCAGCGACGAACATACTTCCGGCTCGCCCGAGGCCCAGTCTTCGCCAAGAA
GCAACCCTGGCCAAATCGCCACGCCAACCGACCAGATCGCAGGAGCATTTAATGCAAAGGTGGCCTGACACCTTTATCCTGATGCGCGCCTTTCAGTCGACAGAG
CCGAAGTGACCGCAGTAACTTCGCCACTCCACTGACCGGCCTGACAGAAGGACAGCGCCGCCTGCGCCGCTCCGACTGCTGCGCCACTCGACAGAGTGAGGCTT
ACAGGCAGTCAGGCCTGGCCTCAGGCACCATAGGAAACTCCGCTTCGCCCGACCCAAGGCTCGGACTCGGGCTCACCCCCGGAAGACGGCGACCTCCGCTCCGC

CCGACCCAGGGCTCGGACTCGGGCTTAGCCCCGGAAGACGGCGAACTCCGCTCCGCCCGACCCAGGGCTCGGACTCGGGCTAAGCCCCGGAAGACGGCGAAC
TCCGCTCCGCTCGACCTAGGGCTCAGACTCGGGCTAAGCCCCGGAAGACGGCGAACGCCGCTCCGCCGCCCGACCCAGGGCTCGGACTCGGGCTAAGCCCCTG
AAGACGGCGAACTTCGCTCCGCCCGACCCAGGGCTCGGACTCGGGCTCAGCCCCGGAAGACGGCGAACTCCGCTCTGCCCGACCCAGGGCTCGGACTCGGGCT
CAGCCCCTGAAGACGACGAACTCCGCTTCGCCCGACCCCAGGGCTCGGACTCAGCCCTGGCCTTCGCCGACAGTCTCCGCCTCGCCCTACCCAGGGGCTCGGAC
TCGACCTTGGCCTCGGAAGACAGACTCGACCTCGACCTCGGAGGAGCCTCCACATCGCCCAACCTAGGGCGCGGACCGACCACGTCAACAGGAGGCGTCATCAT
TACCCTACCCCAAGCTGACTCAGGCTACGGGGAACAAGACCGGCGTCCCATCTGGCTCGCTCCGCCAGATAAGCAATGATGGCGCCCCGCACGCTCTATGACGAT
GGCGGCTCTCAGCCCCCTTACGGAAGCAAGAGGACGTCAGCAAGGACTCGACAGCCCTGACAGCTGTCCTTCCGCCAGGCTCCAGCGCTCCTCCGACGGCCACG
ACACCACACGAACCGGGTGCCAAAACCTCTCCGGCTGCCACGACGGCATGTACTTAGGGCGCTAGCTCTCCTCCGCTAGACACGTAGCACTCTGCTACACCCCCCA
TTGTACACCTGGATCCTCTCCTTACGCCTATAAAAGGAAGGACCAGGGCCCTCTTACAGAGGGTTGGCCGCGCGGGGAAGAGGACGGGACAGGCGCTCGCGTG
AGGCCTCTCTCTCCCTCTCCCGCGTGGACGCTTGTAACCCCCTACTGCAAGCGCACCCGACCTGGGCGCGGGACGAACACGAAGGCCGCGGGATTTCCACCTCTC
TCTCACGCCCGTCTCCAGCCGCCTTTTCCCCCCTTCGCGCTCGGCCTCGCGCCGACCATCTGGGCTGGGGCACGCGACGACATTTCACTCGTCGGCCCAGGGACC
CCCCGGTCTCGAAACGCCGACAGTTGGCGCGCCAGGTAGGGGCCTGCTACATGTTGACGAACAGCTTCCCGTCAAGCTCCAGATGGGCAGTCTCCAGCAACCTCT
CCAGCCCGGGACGGTGCTCCGTTTCGGGAGTCTTGAGTTCATGTCCCTCGACGGCAGCTACGACATGATACTCCTTCCCCCGTCGTGCGACAGCGACAATGGCGG
CCGACAGCCCGCCCGCCGGCGGCGGAATCGACGACGTCTTCCCCGCGTGGTGGAAGAACAACATTCGAGCTCACCCCGACCTCTCCCCCACCGACGGAGGAGG
AGGCGGGGCAACCAAGGCCAAGCAGGAGGCGGCCCCTCGTCGGCTGTCGAGCAAGTCGACGGTGTCGGCGCCCCAACGGGGGGCGTGTCGGGCATCGACCTC
GCGTTTGAGACGAAGGCGAGCGCCGTCTCCCCGCAACACGCCAATTCCGAGCAAACGGACGACGCCAGCACGCTCGCGAAAGGCTTGCTGGGCGTCACCCTCG
TACCTGAGACGACGGTGCAATCAGTCCCTGATGTGACTTCATCGCCGCCCGTCGACCAAGAGGTACCGACCGTTTCCCATCTCACGCCCCTTGGATTCAGCCTCGA
CCCGCCAAGCGGCTTCGCTTTGGTGGACGCTCTCGTAGAGGCGAGTCCAAACCCTCTGGGGTATCGTATGCGGTCACCCTGGGACCGGCTGACGGACGTCTCGA
CCTACGGGCCCTCAGGGTCCGAGGAAGATGACGAGCCCGACTTCTGTTGGGATTTCTCCGGACTTGGTAACCCCAGTGCCATGCGGGACTTCATGACCGCATGCG
AGTACTGCCTTTCCGACTGTTCCGACGGTAGCCGCAGCCTCGGCGACGAGGACTGCGGCCCAAGTCGTGAATGTTTCCACGTCGATCTAGGGGGTCCCTCCGAAG
GCAACCATCTCGGTATGCCGGAGAACGGTGATCTCCCTAGGCCTGTGCCTCGCGTTGACATCCTACGGGAGCTAGCTGTGGTCCCCATTCAAGCGGGGGGTCATG
ACCCACAGCTTGAGCAAATCCGCGGGGTGCAGGCCAGGCTCGACGAGGGAGCAGGAGCACTTGAGCCGATCCGCCGGGACGTCGGGCAGGAATGGGCGGGC
TAGCCTCCGGCCGGAGAAATGCGTCATCTCCCCCAGGGTATCCAGCACCGCATCGCCGACGATGTTAGGGTAAGGCCGCCACCCGCTTCTAGTGGGGTCGGCCAG
AACCTGGCTGCAGCGGCAATGCTTCTCCGCGCGATGCCGGAGCCATCAACCACCGAGGGGCGGCGAATCCAGGGAGAGCTCAAGAATCTCCTGGAGGGCGCCG
CGGTCCGACGGTCCGAGAGCTCCGCCTCCCGGAGGCAGGGATACCCCTCGGAACATCGCACCGCGACTTCCTGATTCATGCGGGAAGCCTCGGTCCGCACCGGG
CGCACGCGCAACACAGCGCCTGCGGCCCCGGGTCGCCTCGGCAACGAGCACCATCACCGCGACCGTCGGGCCCACCTCGATGAGAGGGTGCGCCGAGGCTACC

ACCCCAGGCGTGGAGGACGCTACGACAGCGGGGAGGATCGGAGTCCCTCGCCCGAACCACCCGGTCCGCAGGCCTTCAGCCGTGCCATACGACGGGCGCCGTT
CCCGACCCAGTTCCGACCCCCGACTACTATCACAAAGTACTCGGGGGAGACGAGACCGGAACTGTGGCTCGCGGACTACCGGCTGGCCTGCCAACTAGGTGGAA
CGGACGATGACAACCTCATCATCCGCAACCTCCCACTGTTCCTCTCCGACACCGCTCGCGCCTGGTTGGAGCACCTGCCTCCGGGGCAGATCTCCAACTGGGACG
ACCTGGTCCAGGCCTTCGCCGGCAATTTCCAGGGCACGTACGTGCGCCCCAGAAACTCCTGGGACCTTCGAAGCTGCCGGCAGCAGCCGGGAGAGTCTCTCCGG
GACTACATCCGGCGATTCTTGAAGCAGCGCACCGAGCTGCCCAACATCACCGATTCGGATGTCATCGGCGCGTTCCTCTCCGGCACCACCTGTCGCACCCTAGTGA
GCAAGCTGGGTCGCAAGACCCCCACCAGGGCGAGCGAGCTGATGGACATCGCCACCAAGTTCGCCTCTGGCCAGGAGGCGGTTGAGGCTATCTTCCGGAAGGA
CAAGCAGCCCCAGGGCCGCCGACCGGAAGATGCCCCCGAGGCGTCAACTCAGCGCGACACCAAGAAGAAGGGCAAGAAGAAGTCGCAAGCGAAACGCGACG
CCGCCGACACGGACCTTGTCGCCGCCGCCGAGTACAAGAACCCTCGGAAACCCCCTGGAGGTGCCAACCTCTTCGACAAGATGCTCAAGGAGCCGTGCCCCTATC
ACCAGGGGCCCGTCAAGCACACCCTTGAGGAGTGCGCCATGCTTCGGCGCCACTTCCACAGGGTCGGGCCACCCGCGGAGGGTGGCAGGGCTCGCGACGACG
AAAAGAAGGAAGATCACCAGGCAGGAGAGTTCCCCGAGGTCCGTGACTGCTTCATGATCTACGGTGGGCAAGCGGCGAACGCCTCGGCTCGGCACCGCAAGCA
AGAACGTCGGGAGGTCTGTTCGGTGAAGGTGGCGGCGCCAGTCTACCTAGACTGGTCTGACAAGCCCATCACCTTCGACCAAGCCGACCACCCCGACCACGTGC
CGAGCCCGGGGAAATACCCGCTCGTCGTCGACCCCGTCATCGGCAACGTCAGGCTCACCAAGGTCCTCATGGACGGAGGCAGCAGCCTCAACATCATCTACGCCG
AGACCCTCGGGCTCCTGCGTGTTGATCTGTCCTCGGTCCGGGCAGGCGTTGCGCCTTTCCACGGGATCGTCCCCGGGAAGCGCGTCCAGCCCCTCGGACAACTCG
ACCTTCCTGTCTGCTTCGGAACGCCCTCCAACTTCCGAAGGGAGACCCTCACGTTCGAGGTGGTCGGGTTCCGAGGAACCTACCACGCAGTACTGGGGTGGCCAT
GCTACGCGAAGTTCATGGCCGTCCCCAACTACACCTACCTCAAGCTCAAGATGCCGGGCCCCAACGGGGTCATCACCGTCGGCCCCACGTACAAACACGCGTTCG
AATGCGACGTGGAGTGCGTGGAGTACGCCGAGGCCCTCGCCGAATCCGAGGCCCTCATCGCCGACCTGGAGAGCCTCTCTAAGGAGGTGCCAGACGTGAAGCG
TCACGCCGGCAACTTCGAGCCAGCGGAGACGGTTAAGTCCGTCCCCCTCGACCCCAGCAGCGACGCCTCCAAGCAGATCCGGATCGGCTCCGAGCTCGATCCCA
AATAGGAAGCAGTGCTCGTCGACTTTCTCCGCGCGAATGCCGACGTTTTCGCGTGGAGTCCCTCGGACATGCCCGGCATACCGAGGGATGTCGCCGAGCACTCGC
TGGACATCCGAGCCGGAGCCCGACCCGTCAAGCAGCCTCTGCGCCGATTCGACGAAGAAAAGCGCAGAGCCATAGGCAAGGAGATCCACAAGCTAATGGCGGT
AGGGTTCATCAAAGAGGTATTCCATCCCGAATGGCTTGCCAACCCTGTGCTTGTGAGAAAGAAAGGAGGGAAATGGCGGACGTGTGTAGACTACACTGGTCTAA
ACAAAGCATGTCCGAAGGTTCCCTACCCTCTGCCTCGCATCGATCAAATCGTGGATTCCACTGCTGGGTGCGAAACCCTGTCTTTCCTCGATGCCTACTCAGGGTAT
CACCAAATCAGGATGAAGGAGTCCGACCAGCTCGCGACTTCTTTCATCACGCCCTTCGGCATGTACTGCTATGTCACCATGCCGTTCGGCTTGAGGAATGCGGGCG
CGACATACCAGCGGTGCATAAACCATGTGTTCGACCAACACATTGGCCGGATGGTCGAGTCCTACGTCGATGACATCGTAGTCAAGACGAGGAAAGCCTCCGACC
TCCTTTCCGACCTTGAAGTGACATTCCGATGTCTCAAGGCGAAAGGCGTGAAGCTCAATCCCGAAAAGTGTGTCTTCGGGGTGCCCTGAGGCATGCTCTTGGGGT
TCATCGTCTCCGAGCGGGGCATCGAAGCCAACCCGGAGAAGATCGCAACCATCACCAGCATGGGGCCCATCAAGGACTTGAAAGGCATACAGAGAGTCATGGGA
TGTCTTGCGGCTCTGAGCCGCTTCATCTCACGCCTCGGCGAAAGAGGCCTGCCTCTGTACCGCCTCTTAAGGAAGGTCGAGTGCTTCACTTGGACCCCTGAGGCC

GAGGAAGCCCTCGGGAACCTGAAGGCGCTCCTCACGAATGCGCCCATCTTGGTGCCCCCAGCTGCCGGAGAAGCCCTCTTGATCTACGTCGCCGCGACCACTCA
GGTGGTTAGCGCCGCGATTGTGGTTGAGAGACGAGAAGAGGGGCATACATTGCCTGTCCAGAGGCCAGTCTACTTCATCAGCGAGGTACTGTCCGAAACCAAAA
TCCGCTACCCACAAGTTCAGAAGCTGCTGTACGCGGTGATCCTGACGCGGCGGAAGTTGCGACACTACTTCGAGTCTCATCCGGTAACTGTGGTGTCATCCTTCCC
CCTAGGGGAGATCATCTAGTGCCGAGAGGCCTCAGGTAGAATCGCAAAGTGGGCGGTGGAAATCATGGGCGAAACAATCTCGTTTGCCCCTCGGAAGGCCATCA
AGTCCCAGGTCTTGGCGGACTTCATGGCTGAATGGGTCGACACCCAGCTCCCAACAGTTCCGATCCAGCCGGAACTCTGGACCATGTTCTTCGATGGGTCGCTGAT
GAAGACGGGAGCGGGCGCAGGCCTGCTCTTCATCTCGCCCCTCGGAAAGCATCTATGCTACGTGCTACGCCTCCATTTCCCGGCGTCCAACAATGTGGCTGAGTAC
GAGGCTCTGGTCAACGGGTTGCGGATCGCCATCGGGCTAGGGGTCCGACGCCTCGACGCTCGCGGTGACTCGCAGCTCGTCATCGACCAAGTCATGAAGAACTC
CCACTGCTGTGACCCGAAGATGGAGGCCTACTGCGATGAGGTTCGGCGCCTAGAAGACAAGTTCTACGGGCTCGAGCTCAACCACATCGCCCGGCGCTACAACG
AGACTGCGGACGAGCTGGCTAAAATAGCCTCGGGGCGAACAACGGTTCCCCCGGACGTCTTCTCCCGGGACCTGCATCAACCCTCCGTCAAGATCGACGACACG
TCCGAGCCCGAGGTACCCTCGGCTCAGTCCCAGGTACCCTCGGCTCAGTCCGAGGCACCCTCGGCTCCACCCGAGGCACCCTCAGCTCGGCTCGAGGCACCCTCG
GTTCAGCTCGAGGTACCCTCGACCCCCGAGGGTGAGGCACTGCGCGTCGAGGAGGAGCGAAGCGGGGTCACGCCTAATCGAAACTGGCAGACCCCGTACCTGC
AATATCTCCACCGAGGAGAGCTACCCCTCGACCGAGCCGAAGCTTGGCGGTTGGCGCAGCGCGCCAAGTCGTTTGTCTTGGTGGGGGACAGGAAGAAGCTCTAC
CACCGCAGCCCCTCAGGCATCCTCCAGCGATGCATCTCCATCGCCGAAGGCCAGGAGCTCCTACAAGAGATACACTCGGGGGCTTGCGGCCATCACGCAGCACCT
CGAGCCCTTGTAGGAAACGCCTTCCGACAAGGTTTCTACTGGCCGACGGCGGTGGCCGACGCCACTAGAATTGTCCGCACCTGCGAAGGGTGTCAGTTCTACGC
AAGGCAGACCCACCTGCCTGCTCAGGCTCTGCAGACGATACCCATCACTTGGCCTTTTGCCGTGTGGGGTCTGGACCTCGTCGGCCCCTTGCAGAAGGCACCCGG
GGGCTCCACGCACCTGTTGGTCGCCATCGACAAATTCTCCAAGTGGATCGAGGTCCGACCCCTAAACAACATCAGGTCCGAGCAGGCGGTGGCGTTCTTCACCAA
CATCATCCATCGCTTCGGGGTCCCGAACTCCATCATCACCGACAACGGCACCCAGTTCACCGGCAGAAAGTTCCTGGACTTCTGCGAGGATCACCACATCCGGGTG
GACTGGGCCGCTGTGGCTCACCCCATGACGAATGGGCAAGTAGAGCGTGCCAACGGCATGATTCTACAAGGACTCAAGCCTCGGATCTACAACGACCTCAACAA
GTTCGGCAAGCGATGGATGAAGGAACTCCCCTCGGTGGTCTGGAGCCTGAGGACAACGCCGAGCCGAGCCACGGGCTTCACGCCGTTCTTTCTAGTCTATGGGG
CCGAGGCCATCTTGCCCACAGACTTAGAATACGGTTCCCCGAGGACGAGGGCCTACGCCGACCAAAGCAACCAAGCTAGTCGAGAAGACTCGCTGGACCAGCTG
GAAGAGGCTCGGGACATGGCCTTACTACACTCGGTGCGGTACCAGCAGTCCCTGCGACGCTACCACGCCCGAGGGGTCCGGTCCCGAGACCTCCAGGCGGGCG
ACCTGGTGCTTCGGCTGCGACAGGACGCCCGAGGGCGGCACAAGCTCACGCCTCCCTGGGAAGGGCCGTTCGTCATCGCCAAAGTTCTGAAGCCCGGAACGTA
CAAGCTGGCCAACAGTCAAGGCGAGGTCTACAGCAACGCTTGGAACATCCAACAGCTACGTCACTTCTACCCTTAAGATGTTTTCAAGTTGTTCATATACCTCGCTT
CCACGCAAAGTTTAGTCATCAAGGAAGGGTCAGCCTTGCCTCGGCAAAGCCCGACCCTCCCTCGGGGGCTAAAAGGGGGGAACCCCCTCTGCGTCGAAATTTTC
CTCGAAAAAAGATCCTTTCTGCCAGAATGTCTTTCGTGCTTTTCGACTACTTCGAAAGTGGATCCTGAAAACGACGGAGTACACGTAAGCAGCCAAGGCTGACCG
AGCCGAGGGACTCCTACGCCTCCGGGATACGGATACCTCACTCATCACCTTCTGCGATAAGTAACTCACGTTCGGATAAGTGATTCCGCGGACCGAACAAGTCTTC

ACGTTCGAAAGCTCCTCTGCCGAAGCGATTCTTCGAGCCTTCTCGACCGCGTCGGCAACAGAACCCTATGGACGGGCAAGAGTGCGCGTAAGCGGCAAGGCCGA
CCGAGCCAAGGGATTCCTACGCCTCCGGGATACGGATACCTCACTCATCACCTTCTGTGAGAAGCAACTCTCGCTTGCACAGACAATTCTGTTACCGACGAAAAAG
TCCAGATACTCGAAACAAGAGGAGAAGAAGCGCAGCTTTACAACACAGCGAGGGTGTGTTTGGGCCTCAGCGGCCGCAGAAAACACACGCTACAAGATAATCC
GATCTTGCAGGCTCGGATCTTGACGGCTGAAGGGAGCAGCAGCACCCTCGGCGTCGACTACACCTTCGGCGAGGTCCGACCTAGCCTCCGACGGCGACGTGGTC
CGAGGATCTCCACTCTAAAGGACGACTTCACCATCACGCCCGAGCCATCGCCGCCTGGGTCTCCTCCAAGAATCCGGCTCGAGCAGGCGGCTCGGCTGGTCACCC
CGAGGCCTCGGCCAGCTGTCCCCTGAAGACATCAGCCCGGCCCGAGGCCTCGGCAGATCGACTCCAGCGTCGGTCCCGCTAACGGACGACCCGGCCAGGCTTCG
GCCGACCAAGTCTTCTTTTCGAGCCAACTCTGCCTCTGTCCGTGCTGACACCGCTACCCCTGGCCTCAGCTCATCGAAGAGCGGCCGAGGGGTTCCTTTAACTAAG
CAAGAGAAGCCTCGGACAGCAAGGCCGACCGAGCCGAGGGACTCCTACGCCTCCGGGATATGGATACCTCACTCGTCACCTTTGCCCGGGGCGACTCACGCTTG
GTGAAGCGGTTCAGACAACCAACAGGCGAGTCTTAGTGCTCGAAAATGAGGAAAAAACATGGCTCCGCGCCAAAAATACATACACGTTCAGACCCCGACATCCA
CAATGAACGAAAAGACCGGCATTCAAGGTGCCATTACAAACGGAACTCCGGTTCCACCTCCGCAGGTACGAACAACCCCACACGATTGGGGGGCCTGCGGAGCA
ACAGAAGACCGACGAACGGCACGCCGTCACCCGCTCCAGCAGCGACGACGACGACGACTTCTGCTCCGGGGGGCCGAACAGCAGCAGCGATGACCTCAGGGC
GGATGCTGCTGCCAGGAGGCCCCCGTCCGTGCCCAAACTCGTGAGGCAAGGACGGGCAGAAGGCCGTAGAGTTGGAGGTCGGTCCGTAGGCGGCCCCGGCTAT
CTCATCGACGCAAGAACCTCTTCCAGCTGGCGTGGCGGAAGGCGGCGCCGGGAGCGGCTCCGAAGCCACTCGAATCCGAGAGCCGGGCACGCGGCAGCTGCC
AGCGCCACGAACGGCGAACGCCCTTCCCCCTGATCACTGAGGGAAGGAGCGGGCCACCGCCCACGCAGGGGCCGACCCCAACTCGGCACACTCCCCTCCCCAG
CACTGGTGATGAAAATCCTTGAGGCTGAGGGAGGGGCAGAGGCCGCAGCCCGGCTTTCTTTCCCCCGCCATCGAACTGGAGGTCACCGTCTTGGGTGACCGCCG
GCGGAGGGGTGCAGCCGGGCTGCATGATGAAAATCCTTGAAGCCAAACGATGGCTGAAAGGTACCAACTCCCACGGAGTTGTGTTCCTCCAACGACAAGAAGG
AAGGACTGCGGGCGTCCCCTATCCGGGGGCTCGGAAGGTGGAAAGACACGATGCATAAGGGAGCGCGTAGACATGGTCGCCTTTCAAGGGGGTCACCCCCCTT
TTAAAGGCGACTCTCCCTACTTGCGTCCCCAGCCGTCGTGGGCTGAGTCTTCTCCAACACACTCCAAGGTCCTCCCCCTACGGCACGGGGGCTGGGTCCCACACG
ACATGCAAGCTGGCCCAGGACGGAAGAAGCCAAACCGCCGCGCGGTGCATGCAACCGCCCAGTGGTTACGAGCATTCCTCCGCTTTCGCCCAGACCGGCGG
GCGAAAGGGCGGGCAGCCATGCAGGCGTCATGCAACCGCGCCAAGTGGGCGCGCCCCTCCGACTTCCAACGCACCCAGCGTGGGGGCCCACGCCCACGCGTCA
TGCAACCGGCGCGCTGGATGCTACGTGCGAGCAACTGCACCGCCATTCGCACCACTGCCACGCCTCCTCGACTGCGGAACCAGTACCGCGACTCGAGGCGACCC
AGCGCACGACCCAGCAGCGCCAGCCTGGCGCGATGGTCAATACGGCCAAAAATGGGCCGGCAGTAATGGCGGTGGCAGGCGGGCAGGATCAGCGGTAACGTC
GTCAGCCAGGCTTACGTCCCATCCTGGGGTAGTGAGAGAACCCTCTCTCACGGCGTGAAGACGACGCGCCCGTGTTCCGTTCCTCGAACGGCTCGCGCACGCGC
AACGACTGCCCCGCGAACCACTCGCCCCGTCGCATTAACTCCGCGGCGGGACAAGCGGCGCCTCTGGCAGGGGAAGCGAGCGACGCTTCGCCTCCGCCATAATG
ACCATGTCAAAAAAGGTACATCGCGTCATTCGATTTCGTATCCTTTTCCTTCTCCTCTTTCTCTCTCTTACAACAGGGACCGGGAAAGGGGGATACCCCGTAAAGGA
TCCATCTCCGTGAAGGAAACAGGCTCCGAGCCTCCCTACTGATCAGAGGTTCGAAGGCTGGCCCCTCGGAGGGGTTTAACAGCCGCCTCAGAGCGCGTGGGCTC

CACACCTACTACTGGTCAGAGGTTCGAAGGTCGGCCCCTCGGAAGGGTTCAACGGCCGCCTCAGGCCACTCGGGCTCCGCGCCCACTACTGATCAGGGGTTCGA
AGGCTGGCCCTCGAAGGGTTCACAACCGCCTCAGACACAGAGCGAGGGATGACCATGGGTACGTTCGATACATAACCAAGGCTCGGGCTACGCTCCCGAGGTAC
CCTAGGACATTTCCGAGACCAGCGGGAACGATCTTGTAACAGAATCCCATCAGAGGGAGGCATCGAGCCCTCGGACCCCGTCGACAGGGGGACCGGGTCCGGC
AGATCACCCGCAGGTACTTTTGGGCGCGCCTCTGGGCCTCTAGCCGACCCCTAACAAATGGGGCACGAACGTCCGCTCGGATTACCCGCCAGCAGCTCACTGGAA
ACACCATGTTCGGCGCCCACCGAGGGCAACATGGCGCTTTCCCACCTCCTCCTTGCGGAAAGGCGACGCAGGGGCGTATGTAAAAAAGTCGAGTATGTCCCTGAT
CGTCCTCTCGCCCTGTGCAGAGGCTCGGGGGCTGCTCTCGCAAACCCGACTCCGGCCAAACCGTTGACAGCGTCAACATACCAGCCCGAGAACTTGGGACCCGA
CCGTGCACCCGGGCTACGGCCAGCTCGCATGAGGGAACGACCAGACCAGCCGAAGCATTGCGCGAGGCATTAAGACCTCGGAGGAGTCAAACCACTCCTCCGA
GGCCTCGGGGGCTACACCCGGCGGGTGCGCTCGCGCGCACCCACCGGAACAAAACGCAACCGAGAAAGGCTGGTCCCCTTGCAAAAAAGTGCGACAAAAGCC
TCCAAGCGAGTGTTAACACTCCCTTCGAGGCTCGGGGGCTACTGTCGGGGACCATAATTAGGGGTACCCTCATTACTCCTAATTCTCAGCTGGTAACCCCCATCAGC
ACAAAGCTGCAAAGGCCTGATGGGTGCGATTAAGTCAGGGATCGGTCCGTTCGAGGGACTCGATCGCGCCTCGCCCGAGCCTAGCCTCGGGCAAGGGCAGCCA
ACCCCGGAGGATCTCCGCCTCGCCCGAGGCCCCCGTCTAGCGACGAACATACTTCCGGCTCGCCCGAGGCCCAGTCTTCGCCAAGAAGCAACCCTGGCCAAATCG
CCACGCCAACCGACCAGATCGCAGGAGCATTTAATGCAAAGGTGGCCTGACACCTTTATCCTGACGCGCGCCTTTCAGTCGACAGAGCCGAAGTGACCGCAGTCA
CTTCGCCGCTCCACTGACCGGCCTGACAGAAGGACAGCGCCGCCTGCGCCGCTCCGACTGCTGCGCCACTCGACAGAGTGAGGCTGACAGGCAGTCAGGCCCG
GCCTCAGGCACCATAGGAAACTCCGCTTCGCCCGACCCAGGGCTCGGACTCGGGCTCAGCCCCGGAAGATGGCGAACTCCGCTCCGCCCGACCCAGGGCTCAG
ACTCGGGCTTAGCCCCGGAAGACGGCGAACTCCGCTCCGCCCGACCCAGGGCTCGGACTTGGGCTAAGCCCCGGAAGACAGCGAACTCCGCTCCGCCCGACCT
AGGGCTCGGACTCGGGCTAAGCCCCGGAAGACGGCGAAATCCGCTCTGCCCGACCCAGGGCTCGGACTCGGGCTAAGCCCCTGAAGACGGCGAACTTCGCTCC
GCCCGACCCAGGGCTCGGACTCGGGCTCAGCCCCGGAAGACGGCGAACTCCGCTCCGCCCGACCCAGGGCTCGGACTCGGGCTCAGCCTCTGAAGACGACGA
ACTCCGCTTCGCCCGACCCCAGGGCTCGAACTCAGCCCTGGCCTCCGCCAACGGTCTCCGCCTCGCCCGACCCAGGGGCTCGGACTCGACCTCGGCCTCGGAAG
ACAGACTCGACCTCGACCTCGGAGGAGCCTCCACATCGCCCAACCTAGGGCACGGACCGACCACGTCAACAGGAGGCGCCATCATTACCCTACCCCAAGCTGACT
CAGGCTACGGGGAACAAGACCGATGTCCCATCTGGCTCGCTCCGCCAGATAAGCAATGATGGCGCCCCGCACGCTCTATGACGATGGCGGCTCTCAGCCCCCTTAC
GGAAGCAAGAGGACGTCAGCAAGGACTCGACAGCCCCGACAGCTGTCCTTCCGCCAGGCTCCAGCGCTCCTCCGACGGCCACAACACCACACGAACCGGGTGC
CAAAACCTCTCCGGCTGCCACGACGGCATGTACTTAGGGCGCTAGCTCTCCTCCGCTAGACACGTAGCACTCTGCTACACCCCCCATTGTACACCTGGATCCTCTCC
TTACGCCTATAAAAGGAAGGACCAGGGCCCTCTTACAGAGGGTTGGCCGCACAGGGAAGAGGACGGGACAGGCGCTCGCGTGAGGCCGCTCGCTCCCTCTCCC
GCGTGGACGCTTGTAACCCCCTACTGCAAGCGCACCTGACCTGGGCGCGGGATGAACACGAAGGCCGCGGGATTTCCACCTCTCTCTCACGCCCGTCTCCGGCCG
CCTTTTCCCCCCTTCGCGCTCGGCCTCGCGCCGACCCATCTGGGCTGGGGCACGTGGCGACATTTCACTCGTCGGCCCAGGGACCCCCCGGTCTCGAAACGCCGA
CAAACATACACCACGGTATATGTCGGAGAGGAAATTCTCTGGTCGGGTGGCGGAACGCACCCACCCTAAATCCTAAGATGAGGAGGGGCCTAAGCGTTTTGCCTG

CTAGATGGAATCGGGAAGAACACAAGAACACACCAAGGTTTAGAGTGGTTCGGGCCGTGGAAGCATAATACCCTACTCCACTGTGTGCTGTATTGAGCTTGTATG
AACTTGTAAGTCTGAGAGTAAGTGAGCTTGTGTGAGCGTAAGTTGGGTCTGCCTTGTAACGTCGTGTGCCCTCCCTTTTATAGCTCAAGGGGGCACATACAAGGAT
ACTGAGCCCCGACATGTGGGCCCAGGGGCAAAATGGAAGGAAGTATCTACTGCCTGTAACGCTAGGGTGATCTCCGAGTGCCATAATGTCCGTAGCGCATATAGTA
TTGATATGATGCGCTGCTTCCTCAGTAACGCGCGAGTGATGATGAGCATAGTGTATGCTGTAGCACGAGCGTACTGCCTGCCAACGGAATGGACAGGCACGCCACC
TGCAGGATGGTTTGGTCGCCGCTCGTCAGCGGAGTGGACAGGGCACATTAAATGCCGAGGTGGCACATCGCCTGCCAGTGGAGTGGATAGGGCTCATTAAATGC
TGAGGCGGCACATCACCTGCCAGTTTGACAGGTGCCGCGCTTTATCCGCAATAAATGCAGAGAACGCGCGGCCCAGAGGCCTTACGTCACGCTCCGTCCGCTGGC
TTACGTCATGGGCAGTGGGCCATGTGGCAGCTCGGGTCTCCGCATGAGCGGGGAGCAGAAGCGTACATGGTATGGTCCGGACACGTGTCAGCTCCGAACCCTCG
CCTGGCCTTGATTAAGGCCTGGGTATTCTTTGTTTCAGAATCCCGGGACCCCGCTGTGAGTGGCCCGGACCCCCCATAGAGGGGTCCGGGACCCGTCCAAGGGGT
CCAGTTTGTACCCGTGGAGGTCCTGGACCTTGCCCGGAGGTCCGGTCCGTATATGCAAGGGTCCGACACTTTCCCATGGGGGTCCGGACTCACTGTTGATACCTTG
GAGGATATTATCTTCCCTAGCCATGTGGCGGCCCCGGAGCTGTCCACATGGTGGGGTCGGGTGCTGTTTACCACGCGACTAGAGATAGCCGTGCGGGCACTGCGT
CTTCATACTGTAGTAAGGGGTACCCCCTGTTTCAGGGTACCGACAGTGGCCCCCGGGCCCACCTCAGTGGAGGATACGAGCCTGCAGGTGGGGCCAAAGCTTGAT
TGGCGATTGGCGCGCCGCTTCCGTGCGCTCGCTGACGTAATCACTACCAGCCCGCCTTCGGTCACGCCAACTGCCAAGTCTGTCCCCACGGCTGACTGACCCGCG
ACCCCCGCACCTGATAGTTTTGTCAGGCCACGCGCGAGGCGCCTCGATACTGTTGCATTGGTTTTAAAAATTCACCTTCTCTGTCTTCTGCGGTGGCCCCGAGGAG
GCGCAGTACTGGCGCGAGCGGCGGTTCGACTTTCCTGCACCCGACAACTGACGCCCAAGGAATATGACTCGTGGGCCTAGGCCCCTGTGTCAGAGACTGGGCCG
TTGGTTTTGGTGGAGATGAAGAGGTGCACTACCGCGGGGCGGTGGTTCGCTCCTCGCGTGGCGGTTTGCCTTCTCTGCACCCAGGAACTAATGCCCAAGGAGGA
TGACTCGTGGGCCCGGGCCCCCGTGTCAGAGACTGGGCCGTTGGTTTCGGCGGAGATGAAGAGGCGCACTACCGCGGGCGACGGTTCGCTCCTTGCATGGCGG
TTTGCCTTCTTCGCACTCGGAGACCCGGACACCAGCTGTATGACTTGTGGGCCCAAGCCCCCGTGTCATAGGCTGGGTTGCATTGGTGCATGTCGGGCGAGGTTC
TCTGCGGCGTCTCGGGCGCGAGCAAGGGAGTCTTCCTTGAAAAGGGATTCACCCCGCGTGCAGTGGTTATGCTTTCACATTTTCTCCCAACTGCTGCGCCCTTTCG
CCTTCGAGCTCTCCGCGCCCCTCTGCATTTGCGCTCCTCTGCTTTCCACCGCCGTAGTCGCCATGGCCTCGCTAGTCCATCCCGAGCGCTTCTAGTCCGAGGTGGCG
CTCAACTTTATGCGCAACCTGCTCGGATAGGGTGTGCTGGCGTTCGCCAGAAGGATCCACGCCAGCGCCTCTTCCCTCGGCGATCTCGCCGCCGGGGAGTTCGTG
CTCTTCGTCTCCTACCTCTCCTGCGGGTTGGCGCTGCCGATCTTGCCTTTCTTCCTACTGCTGCTGGGGGAGCTCGGCCTCCAACTTCAGCACTTCACGCCCCACTC
CATCCTCTAGGTGGCCATCTTTGCCCACCTCTGCGAGATGTTCGTGGGGGCGGCGCTTCTTCCGCCAGCGCTCAGATGAAAGGGTGTTACGCATCGACCTTATGGA
GAAAGCGAGTTGAGCTACGGCTCTATCTTCGCGCGAATTTTACCAGCAACTCATGGTACGCCTCCATGAACTGGTCCAGCTCTGGCTCCGTCTTCAGCGGCCAGGC
TGCCGAGCGTCGTGCACTGCCGCACCTCCATATCTTTGGCTTTAATGGCCTCGACGTCGCCTCCAACGATGAGGGCTCCTTCGCCTTGATGCATGCATCGAGGAGG
CCGCCGCCGCTGGCAAGCTGCAGCACCGGGTTGCCGCTGGCGCCGCTGTTTCCAGTGGTCGCCACCGTGTTCGGGGTGAGCGGCAGGCCTGCAAGCCACCGTG
GAGACGCTGCCCGGGAGGTCGTACAAGACGCCGTGGAGACGGCGGTCGCACGCTTCCAGTGCGGCCCTGTTGATGTCGAGTAGTTCATACCTGTAGAAATGAGG

CTAGGACTCCGCTTGAAGGCAGATGTAATAACTTTGTTTTTGTAAGATACTTTTGGGAACTATCAATTAAATAGCTCTTATCTGTATGGCATTTGTTCTTGCTGTGTGT
GGCGTTACCAACTCCTCTCCTGGCACTTGGCCCCCCTGGGATGTAGGCTCAACGTGTCAAGGCTGGAGACCAGCATACCTAAGAAAGAGTTGGTGACCCGGCCCC
CAGAGGGTAGCCTCGACTGGGACCTGGACCTACACATAGCCTTGGCTAAGAAGTGTTAGTAGCACACCCATAGGCCCATCGTCTGGTATCAGCTATCCTTTGATTCA
CGCAACAGGACCTGCAAGATTTAGCCTAGGAAGCCAAGCCATGTGCCCGAACCCCTTGGATCACAGCTCCAGATACTAGGACACCCATCGCAGAGTGGTGGAGC
GTGCAGGCTTAGGGTACGGGACTAGGCTAAGCGGCTACACGGCTCCGGACCACCCCAGGAGACGTGCGTCCATTCTCTAGAACCAGACCCTAGGTTCCCGGACC
CATAGGGCTATAGTTCCAAATACTAGGATACCCGTCGCAGAGTGGTGGAGCGTGTAGGCTTAGGGTACGGAACCAAGCTAAGCGGCTACACAGCTCCGGACCACC
CCAGGAGACGAGTACCCCTTCTCTAGAACTGGCTTCCAGGCTGTCGGACCCCCCTACGGGGGGCTCCCAAACTGCAAGCCTGACTACTCAATTCGGATGTCATTAT
ACCAGCAAGGGTGGGAACTGTATGGGTGGGTTAGATAAAAAATAATATCCGGAAACTTGTAGCAGGATAAAACCATCACAGAAGCACATCTGAGGGGGTAAATCC
TTTCTTTATAACTCGATATGCATGGGTGCAGATCAACAGGCCGGGGCTTATGAGGGCGGACCTCACCGGGCTGGCATACACATATGCACAACCTAGTTAGAAAAAG
AAGAAACCTCAACCCCATTAGACTTGCTCCTATGGGTAGAACTTACGGAGATGCTGTATGTTCCAAGGGTTGGGAAGAGGCACTCCTTCAGCTGTGGCAAGGCGG
ACATACCCGGGTCGGCATACTTCCGTCACCTTGAAGGGTCCTTCCCAACTGGAGGGGAGTTTGTGGAGCCCTTCTCGGTTTAGAATTTGCCTTAGGACCGGGTCCC
CGACCCTGAGCTCCCTACTATGCATGAACCGCTGGTAGTAGCGCCCGAGCGCTTGGTTGTAACGTGCATTTTGGATCGCCGCTTGCCATCTGCGTTCGTCGATGAA
GTCCACATCCTCCCGCCGTAGCTGTTCCTGCATAGACTCATCAAACGACTGGACCCATGGGGAGCCCATAAGGATTTCCAGGTAAAGGCAGGCTTCGGGCCCGAA
GACCAGGGAGGACGGAGTCTCCTAGGTAGCCCGGCTAGCTGTCGTCACCGGAGTCGGAGTCTCTGGTGAAAACATCCTTCAGGACCCCCTCGGGTGACTGATAC
CCGAGGTCCCGCTCTCCCGTGGCCACCTCGCCGTCGTCGACCCTTTCCTTGCCAGGCCGGCGACGAGGTGGGGAGCCATCCTTGGAAGTCTGCTCGCGCCGCTC
GCTGACGCGCTTCGCGAGCTCGATGATCTCGCGACACTCTGCGGCGCTGTGGCGACTGTTGGGGTGTACAGGGCATGAGCCACTGTTACACCTCTGCGGCCGTG
GGCGCTTGCTGCGCTAGCTCCGGCCCCCAGTCGCGGCTGCGACGACTGGAGCAGCAGGCAGCGGCCTCCCAAGGCCGTGGTTCTTCTTCTTCTTTTTGCCGTCTT
GGGTGACGGCACCAGAGCCACCCGTCTGGGCGACTCCGGTTTGTGGTGTCGAGTGCCATGCACAGCCCTCGGCAGCTCTGGCACACTTGTCAGCCAGAGTGAA
GAGTGTGGTGACAGTCTCCACGTCATGTGTGGCTAACTTCTCCAATATCTTTTCATCACGTACCCCCTGGCGGAAGGCGGTGATGATGGAAGCATCGGAAATACGA
GGTATAGTGCCTCGTACCTTGGTGAGTGGGAGATAAACGCCCGGAGAGTTTTCCCGGGCTCCTGCCTCACTGCGTGGAGGTGGGCCTCCACGCCATGCTGCTGAT
AAGCGCTGGCGAAGTTCACTGTGAACCGCACGTAGAGCTCTTCCCAGGAGTAAATCGATCCTGGGGCAAGGTTCATGAGCCAAGTCCGGACTGGCCCAGACAAG
GCAACATGGAAGTATGTTGCCATTACGGCGGTGTCTCCACCGGCTGTCGTAATGGCGGTGACGTAAACCTGCAAGAATTCTGACAAATTTGACATACCGTCGTACTT
CTTCGGTAGGTGCGGCCGGAACTTGGATGGCCAAGTCGCCGTGCGGAGATGATCCGCTAGTACGGCGCAGCCCACGCCGGCCAATGGAACACCCGCTTGGATCC
AAGCGCCTGTTGGAGTCTGCGGTGCAACCACAACGAAGTCGTAGTCGAGGTTGCGACCCTCGACGTTTTGTCGGCGCTCACGCGCCTTCTCCAGAGAGACACGG
GCATCCTCGCCTGCACGCCTGCGGTTGAGTTCCGCCCGGAGGTTATCAGTCCTTCCCAGAGAGGCTGGAGCGCCCTCGCCCGCTCGTCGGCGGTTGAGCTCCGCC
CGCAGGTCCTCGGTCGGTGTAGCCCTCACCGAGGGCGAGCGCACTGACACCGACGCCTCATGTTGACGCCGGGATGACCGAGGCCTGGGCCTGGCTGAGCCAG

AATGCGCCATGCCGAGCAGACGATCGACATCATCACGCCACTACTTCATGGGTGCCGGGGAGGCCGTGGAGCTAGGAGGGTGGCGCAGCAATTCCCTAGCCGCA
GATAGCGCCCCAGGCGCAGCCCTTGACGCCCTAGACGTTTGCGCGGGAGTGTGCTGTCGCGCAGAGTGCACAGTAGCAGCTGCACTAGGCACAAGGAGGTTGG
TGGCCCGCGGTGTGGATGAGGCAGCTGCCTCCTCCACCATGAAGTCCTTGGGCATGAAATCATGGTGCTCGACGATGTGGACCATGGCACCGAGCAGAAAAACA
AGCAAAAACCTAAAACCTAAGGCCCCTACCCGGCGCGCCAAATGTCAGAGAGGAAATTCTCCGGCCGGGTGGCGGAACGCACCCGCCCTAAATCCTAAGATGAG
GAGGGGCCTAAGCGTTTTGCCTGCTAGATGGAATCGGGAAGAACACAAGAACACACCAAGGTTTAGAGTGGTTCGGGCCGCCGGAGCGTAATACCCTACTCCAC
TGTGTGCTGTATTGAGCTTGAGAGCTTGTATAAACTTGTAAGTCTGAGAGTCTAAGTGAGCTTGTGTGAGCGTAAGTTGGGTCTGCCTTGTGACGTCGTGTGCCCT
CCCTTTTTATAGCTCAAGAGGGGTACGCACAAGGATACTGAGCCCCGACATGTGGGCCTAGGGGCAAAATGGAAGGAAGTATCTACTGCCTATAACACCAGGGTG
ATCTCCGAGCGCCATAATGTCCGTAGTGCATATAGTATCGATATGCTATGCTGCTTCCTCGGTAACGCGCGAGTGATGATGAGCATAGTGTATGCCGCAGCACGGGCG
TACTACCTGCCAACGGAATGGATAGGCACACCGCCTGCAGGATGGTTTGGTCGCCGATCGTCAGCGGAGTGGATAGGCCATATTAAATGCTGAGGCGGCATATCGC
CTGCCAGTGGAGTGGACATGGCTCATTAAATGCTGAGGCGGCACATCACCTGCTAGTTTGACAAGCGCCACGCTTTATCCGCAATAAATGCAGAGGACGCGCGGA
CCAGAGGCCTTACGTCAGGCTCCGCCCGCTGGCTTACGTCACGGGCAGTGGGCCACGTGGCAGCATCGGGTCTCCGCCTGAGCGGGGAGCAGAAGCGTATATG
GTATGGTACGGACACGTGTCAGCTCCGGACCCCCGCCTGGCCTTGATTAAGGCCTGTGTATTCTTTGTTTCGGAATCCCGGGACCCTGCTGTGAGTGGCCCGGACC
CCCCACAGAGGGGTCCGGGACCCGTCCCAGGGGTCCGGTTTGTACCTGTGGAGGTCCTGGACCTTGCCAGGAGGTCCGGTCCGTATATGCAGGGTCCGGCACTT
TCCCATGGGGGTCCGGACTCACTGTTGATACCTTGGAGAATATTATCTTCCCTAGCCACGTGGCAGCCCCGGAGCTGTCCACGTGGTGGGGTCGGGTGCTATTTAC
CACGCGACTAGAGATAGCCACGCGGGCACCGCGTCTTCATACTGTAGTAAGGGGTACCCCTGTTTCAGGATACCGACAATATATCTCCGTGTATGTCCCTTGTGATCT
CATCTATCACCAAAGCGAAAAGATAAGGGCTCAAAGCCGACCCTTGATGTAGTACTATGTTAATTGGAAAGTCATCGGTGTCTCTATCACTTGTTCGGACACTTGTC
ACAACATTAGTGTACATATCTTTAATAAGATTAATGTACTTTGTTGATACTTTGTGTTTTCCAAGGCTCACCACATTACACTCATAGGTACTTTGTCATAAGCCTTCTCCA
AGTCTATGAAGACAATATGCAGGTCTTTCTTTTGTTCTCTGAATCTCTCCATAAGTATGTGACATGAAATGGTTCAAATGAATGATTTTTTGTGTTTAATACTCTAAAGT
AGTGATCCGCTATTGATAAAAAATTGAGTGCTGACAGACGGTGCATTTCTAGTGTTTCGCCAAAAAATAAAATAAAATAAAATCTATAGTAGTCTAAATGGATGTAGTC
TCTCTATTTTATTATATTTTAATGCATAAATTATCAAATACAGAAACTAAAACTTCTATTTTAGTTTTTGTATTTAGTAATTTAGATACCAAAATAAAATAAAATGAATGTA
CCAAAAATTTGTTCTTAGAAACTATACACCCCTTGTTTGCTGCTGACCACAATAAATACTTCTACACGCACTTTTCAAAAGCTGAGTAGCAGGATGAGCGTGACGAG
CCTCATCTCCCTAGCAAACCAAGAACAGAAGTCATCCATCCGTTTTGCTCCGTAGACGAGAGCGAGACAAACGGTTGGCGAATAGTCAGCCGAGCCAGCAGCAGC
AGCTTGCTCCGCCGACGTTGCAATTCCGGGAATGCTGGAGGGCGTGCTCGATAGATTTCAGGGGCCGGAGCGCGAGTAAACCACGAGAAAAGATAGTGGCTGA
GATTTCTCGCGGCGCTCGCCGCGAGAGCGACCCCCACAGGTCGCCGCCCCTCCGCGGCCCGCGCAGCGCACAGCATACAATACAGTCAGCACACGCAGCAGCTG
CCACCACAGCGAACAGCAAAGCACCTCCGACTCGGAGTCGCGGCGAGAGCCGAGAACCTTCTCATTCATCTTCCTCTTCCATCCATCCTGCCTGCCTGCTTGCCTC
CGTATTCCAGCTAGATCTCCGGGCAGCCGATTGGCCGAGAAGTTTGTGGTTGTGGCGGCGGCGGAGAGGGAAACGGGGAGCGGGAGGGCGGCGCGGATGCCA

CTGCTGCGCCGGCGCCGGCGGGGCCGGTGATGCCCGCCGTGGCGGTCGCTTGAGGTGGGTGGAATCGGGCGTCGTTGTGCGGTACTTCGTGACGTTCACTGTC
ATTAATGAGGTACCTGACGCTCGAATGGGTGCGCGGGCTGGTGCAGGCCGCCGTGCGTGGGAGGTGGACGGGGGAGCGAGGCGGTGCGGACCGAGGTTGCGT
TGGGTTGGATTCGATTGGGCTGCGTCTGCTGAGGTACTACTGTTTCCCTACCTAAAATTCACGGACCTCTGTTTCTAGTCCACGAGACATGCTCCCCCCTCCCTCCCG
GATTACTACTGTTCTTTTGGCTTGCAGAAAATCACTTGTCAAAATGTTCGATGGTGCACTTCGTTCTTCCTGGGCTACGTGTCTTTTCGATGCTTTAATGTCGTCATGA
TTTCTTGTGTGCAATTTGAGAATCTGAAGAGTGGTAACGGACAGTAGTTGTAATGAGCTATGCCGCTTCTCGCTGTGTTTCTTTTGTCGAGGACGGCGGTTGGGAT
TTGTTCGTAGCTGTGTGCTTGGTAACTTCAGGGGGTGAGTGTTTTCTTGGCGAGGCTAGCAAGATATTGTTAGGCTGTTTGGTTCCATGACTAAAGTCCATATCACA
TCTGATGCTTGAATATCATTTACGAGTATTAAATATAGTCTAATTACATAGAGAAACACTAAACGATGAGATATATTTATTAAGTCTATTAATTCATGATTAACAAATATTT
ACTATATCATCACATGAACGAATCATGGATTAATTAGATTTAATAGGTTCATCTTATCGTTTAGTTCTCATTTGTATTATTAGTTTTGTAATTAGACTATATTTAATACTTCT
AAATAGCATCCAAACATCTGATGTGACAGGGGCTAAAATTTAGCTCCTTGATCTAAATAGCCCTTGACTATGTTGGGTGAATTAAAACCCTAACTAGGGTTAGGATGT
TGTATTAATAGGTAGAAACATATATGGGCTGGGCCTAAGCCCAATAGAGTACACATGTCGAACACCCCCGCAAATGCAATTCTAGGTATTACATATGTTGAGATTGAT
GGAATAAGTCATGTGGTCTTCGTGTGGAGGACAGTCCGCACAACATCTCTGCTATCCGCACAACATCTCTGCTGTAGGACAACAGCAACAACAGTTTTTGACTGCA
AGGACTACGGCAGACAACACAGCTTGACTGCAAGGACTGCGGCAGACAGCACAACAGGAGTCTTTTGACCGCAAGGACAGCGTGCTGCAGGCATCTCCTTTTTC
TTTTTCCAGCTTTTGGAGGACAGCAGCTTGCTGCAGTTTTCTCTTTTGACTAGAGTACAACAACACCTAGTGGTTAGTGCAGTTTGTAGGACAGCATATTTGCAACT
CTAGGAAGCATATGTTGCAGCCCCTTGGGCTATAAATATGTATCCCAACCCTTAGACGAGTATGACATTGTGTAGTGTTTGAGGAAATAAACAGAAAATTGCCCTAAC
TCATAGTGTCATCCTCTTGATGAGAGTTAAAGTCCCTCTACTTACAATTGGTATCGGAGCCAAACTATCCTGCAGCCTAAACATCTCTTGCTCATCTTCTTCCCGCGCC
TCTCCCAACACTCAGTCGGCAGCAAGAGCTCCAACTGTTCCTTCCTCTCCTGCTCAGACGAGCAACCTTTCGTCTGAGACAGCCTCTTCCACACGCAGCGACCCCT
CACTAGCCCATCATGTCCCTACGCTCGGTCACTTCGAGTGCGCGGCGCCAGCAGGAAACCGAGGTCGCCGTGGCACAAGAACGAGAGCGAGCAGCAGCAGCGA
CTGCAGCGACAGCGGCGAGGGCAGCACGGCTGGCGGCAGCGGAACTGGCAGCAGCGAGAGCGGAAGTAGAAGCAGCGGAGGCGGCGAATGCTGCACGTGC
GGCGGCAGCAGTGCTTGAGGTTCTGCGCGGTAGTAGAGCTGGCAGCTCTGCTTCTGTCGACGACAACACCGACGAAGAGCTCAGGCTAGCGAGGAAAGCAGCG
CGAGAGCAGACGGCACAGTGGGCAGCCGCGCACCCCCATGGGGGCGCGCGTGGCGGCGGCCCAGATAGGCGTCAACGCGCTGACGGCGCTCCGGGCGAGGG
CGCACGCGGTGGCAGCCCAGACGGGCGTAGACGCGCCGGCGGTGCTCCCGGCGGCGGCTACCGGGTCGACGGAGATCGCGGCCTCTACAGGCGGCGCGACTC
TCCCTCCCCGGATCGGTACCATGGTCGCCGCATGGCCCACGCCATTGTCAGGGACATCGGTCCCGGCGGTGGGTGGCCTACCCTCACCAAGACCAACTACGTCGA
GTGGGCCGCAGTGATGAGGGTACGGCTCCAGGTTCGCCAAATGTGGGAAGCAGTTCGGTACGACGACGTCGACTACCACGAGGATCGGCGGGCGCTTGATGCC
CTCATTGCTGCAGTCCCGTCTGAGATGCAGTTTTCGCTTTCCCAGAAGCGGACTGCCAAGGAGGCCTGGGACGCCATCGCTGCGACCCGCATCGGCAGCGACCGT
GCCCGCAAGACCACACTGCAGGCACTTCGCAAGGAGTGAGAGAACCTGACCTTCAAGCCAGGTGAGGATGTTGATGACTTTGCTCTCCGCCTCAACACTCTGTTG
CAGAAGATGGTGCAGTTCGGCGACGACACCTACGATGAGGAGAGAGCTGTTGAGAAGCTTTTCCGTTGCATCCCCGAGAAGTACAAGCAGATTGCTCGCTCGAT

CGAGTCTCTGCTAGACCTCTCCACGATGACAATCGAAGAGGCGATAGGTCGTCTCAAGGTGGTCGACGGCGACGAACCACAGGCTCCCTCTGGGCCTATCACTATC
GGCGGGAAGCTACATCTCACTCGGGAGCAGTGGGAGGCCTGCTAGGGTGACCAGAAGAAGGGGGAGTCCTCCTCGACACGAGGCCGCAAACGCGGCAAGCC
GCGCAAGGCGCGTGGAGGCGCCCAGGCCGGGGCGTGAGGACATGCTGAGGGTGGTGCCCGTGGAGGTGCCCAAGGCGGCGCCGTCGGCAACAAGAAGCCG
GCACGAGACGACGGCTGTCACAACTGCGGCAAGCTTGGCCACTGGGCCAGGGACTGTCGACAGCCACGACGTGGCCAGGCCAACGTCGCACAGGCGGAGGCG
GAGGAGGAGGCTCTGCTCCTAGCACATGCAAGCATCGAGCTATCTCCAGCGGCACTCCTCCACCTTGATGAGTCGAAAGCACGCGCTTTGCTCGGCGACGGCCCT
AACAAAGACATGATCGAAGGATGGTGCCTCGACACCGACGCCACTCATCACATGACCGGCCGACGGGAGTTCTTCACCGAGCTTGACTCTAGCGTCCGAGGCTCC
GTCAAGTTTGGGGACGCCTCCGGCGTAGAGATCAAGGGCGCCGGCTCAGTCGTCTTCACCGCCGCATCTGGTGAGCACAGGCTGCTCACCGGAGTCTACTACATC
CCCGCGTTGACGAACTCTATCATCAGCTTGGGGCAGCTGGATGAGAACAGTTCGCGCGTGGAGGTCGAGCACGGAGTCATGAGGATCTGGGACCCCTCTCGTCG
CCTTCTTGCCAAGGTACGCAGGAGTCCAAATCGGCTATACATCCTCAATGTGAAGGTGTCACAACCTTGCTGCCTTGCTGCTCGTCGAGACGACGGGGCATGGCA
GTGGCACGAGCGCTTCGGGCACCTTAACTTCGAGGCCCTGAAGCGGCTCAGCGCCAAGGAGATGGTACGAGGCCTGCCGTGCCTTGACCATGTGGAGCAATTCT
GCGATGTCTGCGTGTTGACAAAGCAGAGACGACTCCCCTTTCCCCAGCAGTCGAGCTTCCGAGCCAAGGAGAGGCTCGAGCTCGTGCATGGGGACTTGTGTGGC
CCGGTGACACCAGCCACACCAGGAGGACGACGCTACTTCTTGCTGCTTGTCGACGATCTCTCCCACTACATGTGGGTGATGATCCTTGGCAGCAAGGGAGAGGCT
GCGAACGCCATTAGGCGTGTGTAGGTCGCTGCGGAGGCGGAGTGTGGCCGCAAGCTGCGCGTGTTGCGCACCGACAACGGCGGCTGAGTTCGCGTCGTACTGC
GAGGATGAGGGCGTTCAGCGCCACTACTCTGCGCCGTACAGCCCGCAGCAGAACGGCGTCGTCGAGCGGCGCAACCAGACGGTTGTGGGGATGGCTCGGGCTC
TCCTCAAACAGAGGGGAATGCCAACTGTCTTCTGTGGAGAGGCGGTGGTGACAGCGGTCTACATCCTCAACCGCTCGCCCACCAAGGTACTCAACGGGATGACA
CCGTACGAGGCTTGGCATGGGCGCAAGCCGGCGGTCTCTCACCTACGGGTCTTCGGCTGCCTCGCGTTCACCAAGGAGCTTGGCCACATCGGCAAGCTCGACGA
CAGGAGCACCCCAGGGGTGTTCATTGGCTACGCGGAGGGCTCGAAGGCCTACCGCATCCTTGACCCAGGAACACAGCGTGTGCGCACGACACACGACGTAGTG
TTCGACGAAGGGCGAGGATGGGCGTGGGACAAGGCGGTGGACGACGACACGACTTCGACGTACGACGACTTCACCATCGAGTACGTCCACTTTGAGGGAGCTG
GAGGAGTAGGCAACTCTTCTCCGAGCAGGTCTACCCCAGCCCCCAAGTCTCCACCGCCTCCAGCGCCACGCTCTCCAGCTCTGGCTACAACGAGCTCTTCACCACC
ACGCACTCCAGCCACGACTCCGGCTACAGCGAGCTCTTCACCACCACGTACTCTAGCACCGACGGTACCCTCTCCGGGAACGTCCTTTCCGACACCAGCTCGTGTC
GAGCACGACCCAGTGGAGCTCGTGACCCCGCTCTCCCGCGACGAGGAGCGCGTCGACGCGTGCTACGACGGCGAGCCGTTGCGGTATCGAAGGGTGGAGGGC
CTTCTCATCGACCCGTCGGTGCCGGGCCCTGCGTCTCGCATTCTGGCAGGAGAGTTGCATCTTGCATGCGACGATGGTGAGCCTCGGTCTTTTGCAGAGGCCGAG
AAACATGCGGCTTGGCGTGTCGCGATGCAGTCGGAGATGGACGCGGTTGAGACGAACCGTACTTGGGAGCTCGCTGATCTCCCTCATGGTCATCGCACGATCACC
CTTAAGTGGGTGTTCAAACTGAAGAGGGATGAAGCCGGCGCCATCGTCAAGCATAAGGCTCGCTTGGTGGCACGCGATTTCTTGCAGCAGGAGGGGATCGACTT
CGACGATGCCTTCGCCCCTGTAGCACGGATGGAATCCGTGCGACTCCTCCTCGCGCTGGCAGCCCAGGAGGGCTGGCATGTTCATCACATGGATGTCAAGTCAGC
GTTTCTTAACGGCGACTTAAAGGAGGAGGTCTATGTACACCAACCGCCAGGTTTTGCGATCCCTGGCAAGGAGGGCAAGGTGTTGCGCCTGCGCAAGGCCCTCT

ACGGCTTGCGACAGGCACCAAGGGCGTGGAATGCCAAGCTGGATTCCACGCTCAAGAGGATGGGCTTCATGCCAAGCCCGCACGAGGCGGCCATCTATCGGCG
GGGCAATGGAGAAAATGCCCTGCTGGTGGGTGTCTACGTCGATGACTTGGTGATCACCAGCGCCAAGGATGCAGAGGTGGCAACGTTCAAGGAAGAGATGAAG
GCCACCTTCCAAATGAGTGACCTAGGGCATCTCTCCTTCTACCTGGGGATTGAGGTGCACCAGGGAGACTCCGGGATCACACTTCGCCAGACCGCCTACTCCAAG
CGCATTGTTGAGCTGGCTGGGCTCACCGACTGCAACCCAGCTCTCACTCCGATGGAGGAGAGGCTGAAACTGAGTCGCGACAGCACGACGGAGGAGGTGGACG
CTACACAGTACCGGCGTCTTGTGGGGAGCCTTCGCTACCTCGTCCACACACGGCCTGACTTGGCATACTCCGTCGGCTACGTTAGTCGGTTTCTGCAGCAACCGAC
GACGGAGCATGAGCAGGCTGTGAAGAGGATCATCCGCTATGTTGCGGGGACTCTCGACCACGGTCTCTACTACCCGAGGTGCCCTGGGGAGGCACGCCTTGTCG
GTTACAGCGACAGCGACCACGCCGGCGACATCGACACTAGCAAGAGCACAAGCGGGATCCTCTTCTTCCTCGGCAAGTGCCCCATCAGCTGGCAGTCGGTCAAG
CAGCAGGTGGTGGCCATGTCCAGCTGCGAGGCCGAGTACATAGCGGCCTCCACCGCTTCAACTCAGGCACTCTGGCTTGCTCGCCTGGTCGGTGATCTCCTCGGG
AGAGACGTTGGAGCAGTGAAACTTCGGGTGGACAGCCAGTCCGCCTTGGCATTGGCCAATAACCCCGTGTTCCATGAACGGAGCAAGCACATCCGGGTGAGATA
CCACTTCATCCGTGACTGTTTGATAGAAGGGAGCATCCATGCGCGCTACATCAACACCAAGGATCAGCTTGCCGACTTGCTCACCAAGCCCCTTGGGAGGATCAAG
TTCCTTGAGCTTTGTTCCAGGTCTAGGATGACTCAACTTTCCCACAAGGCGACGCACAAGACTTAGGGGGAGAATGATGGGATAAGTCATGTGGTCTTCGTGTGG
AGGACAGTCCGCACAGCATCTCTGCTGTAGGACAGCAGCAACAACAGTTTTTGACTGCAAGGACTGCGGCAGACAGCACAACTTGACTGCAAGGAATGCGGCA
GACAGCACAACAACAATCTTTTGACTGCAAGGACAGCGTGCTGCAGGCATCTCCTTTTTCTTTTTCCAGCTTTTGGAGGACAGCAGCTTGCTGCAGTTTTCTCTTT
TGACTAGAGTACAACAACACCTAGTGGTTAGTGCAGTTTGTAGGATAGCATATTCGCAACTCTAGGAAGCATATGCTGCAGCCCCTTGGGATATAAATATGTATCCCA
ACCCTTAGACGGGTATGACATTGTGTAGTGTTTGAGGAAATAAACAGAAAATTGCCCCAACTCATAGTGTCATCCTCTTGATGAGAGTTAGAGTCCCTCTACTTACA
GAGGTTGGAACGAAACTTCAAGAACACCGTGGATGCGAGCCCCTTGGTAAAGATGTCGGCAAACTACGAAGTTGGTACGCGAACATCGTCGACGATGACACTCT
CACGTGAAGTGGAGTTCGATCTCCACGTGCTTCGTGCGCTGACGCTGCAAGGGGTTGGTGGAGAGATAGACTGTGCTTACATTGTCATAGTAGACGAGTGTGGCG
CGTGGGAGGGGGCTGTGAAGCTCCTGGAGCAGCTGACGTGTGATATCCTAGCCCCTGGGATGGTGACCGCCCTGATGCAGTTAACCAAGGTCACCGCGACTCTG
CCCGCAATGGCAGCCACCGGCGTAGAAGCAGCGACGGCGTAGATCCGCGGCCGACGCAGATTTCTGGTGGGAGCAGCGGATCCGCGGCCCTGCTTGCAACGGC
GGCGGTCAGCGCAAATTTCTGGCGGAAGTAGCGACGACGCAGATCCAGATCCGCGTCCCTTGTCCCAGACGGCGGACGACACAAAAGCCGATGATGAAGGGCG
CAACAGGCGGACAACAGGCAGACGACGCAGAAGCGGCGACGGCGCAGATCGGCGTGCAGGGCGCAGCAGGCGGACGACGCAGAAACGGCGACGGCGCAGA
TCCGGATCCGCGGCCCTTGCGGCGCAGAAGTCGACGATGCAGGGCGCAACAGGCGGATGGCGTAGAAGCGGATGGGGGAAGGCCGCGACGGCGGCAGAGGA
GGGAAGGGGGAAGGCTGCGACGGTGGCTGAGGATGTGCGACGGCAGGCCGGAGGGCTGGCTGCGATGGGCGGATAGACGGCAAGGCCGCGACGACGGCTG
AGGATACGCGATGGCAGGCCGGAGGGCTAGCCGCGATGGGCGGACGGCGCAGCAACGGCGCCCAAAAAAATGGACCAGGGCCGGGTTTAGACGTAAACCCTA
ACCTGCTCTGTTACCATGTACTAACCTCGATCAGGCGAGACAATAACCCTCTTGGGTACTGTAGCATATATATAGGCAGACAAGAATATATGGGCTTTATGGGCCTTA
ACAAGACTGACATTCAGTCTGCTAGTCAGACTGGCAGACAGACGTCAGAACAATCTTTTGACCCTGACACTGGTTGAACCGCCCTAGGACCGGTTCAACCGGTTT

TGACTAGAGAGGTTCGGAGAGAAAACCCATGTTGAACTGGCGCTGGGGCAGGTTCAACTGGTTTTAACCAGAGAGCTCCTCAGTTCAAGTCAAGTTGAACTGTC
CCGAAGACCAGTTCAACTCTTTTGAGTAGGGAGGTTCTCCGTCTCCCAAAGCTCACTCCAGTTGAACTCACAGAGTGTCCAAAAGATTTGGTTTTCAAAATAGCTT
TTGAATTTTAGAGGCTTGAGATTTAACAAACATCATTTCCTTGGCTAAAACTACTTAGAAAAAATTAAATCCTGCGACTCAATAAATATAGATATTTTGCATGACCGTC
GCATGGAATCCCCATCAAGGCACCATATGATGTATATCTGTTGTCCTTGATACTTCCTTTTTCTTTCTTAACTAAGGTTAATACTCCTTTACACCTTTAAACAAACTGTTC
ATACGTTAGGAGCAAACTTTGTTAGAGACCTTATTTGTTTTATCATTTAATCACCAAAATCCTCAATTGGAGTTGATTGCACTTACACATCATCTGTATACAGTGATAAT
CTTGTCTTCACAATAGCATCTTCCAAATTATACAACAACTCCTCTTCTGCCACTTTAAAAAGGCTGCTGAGGACATCCATCACCAGGACAAAGAGCATTGGAGAAAG
GGCCCCCCTTGCCTTAATCCTCTGTGATGTCTGATCTGGTCAACTGGAAACCCATTTATTAATACTTGAGTTGATGAGGTGGCTAGAAGACTAGATATAAGATCCCTC
CATCTATTACCAAAGCCAAGATGGGACAACACCTCAAGGAGAAAGGCCCAGGAAACTGAATCAAATGCCTTTGTTATATCCGATTTCAGAAAAGGCTAGCCACTTT
TTCCCTATGTACTGACTTGATAGACTGTTGGAGCAGCATATAGTTATAATGGATGGATCTTCCTATGACAAAAGCACCCACTCTGGTTAGCTTCCACCAACTTGTTGA
GAAGAGGTGCTAGTCTATTGGCAAGGAGCTTTGTGGTAAGTTTAGCAAAGCTGTGAATGAGACTGATGGGTCTGTAATCACCAGCTGTCATGGCATTTGTTTTCTT
GGGGATCAAAGTAAGATAGGCTATGTTCAGTAGCCACAATTTTTGTGCATTACCTTGATGTAGGGTAGTGAGAGCAACCACCAAATCTGCCTTAAGGATAAGCCAG
CAAGTTTTGTAGAATCTCCCAGTATAGCCATCATGCCCAGGTGCTTTATCAGACGGAAGACTGGCTATAGTATCCCAGACTTCCTTTTCTGTAAAGGGCTGCTCTAGA
TCAGACAAGTCTGCTCCATTTCTGTGGCATTCTTCTAAATTCAGTGTTAGCCTTCTCTGGGGGGCATAACCCATCAAATTAGAGTAGAACTCATGCAACACTTCCTAT
TTCTGCTCAAGAGATGTGGCCACTCTCTCTCCCATCCATTAAGCTGGTGATAGACTTTTTGATATGGCCCAGCCGCATAGAGTTTATTTTAGGAAACACTGAGTTGAT
TGGCTGTGATTATTTGGTAGCAAATAGTCCCTTTTTGTAATGGTAGCATTAGTTACCTTATTGAGCATTACATGGCTCTATAGTTTCTATGTTTAGGAAAGAGATTGTG
GGGAAATAAAAGGGAGAAAGGCAGAAGAGAAAAGGGACATTCTTGTTCCACAAATTCCAGTCTCAGGCGTCGCCGACGATAGACGGCCGGTGGTTCCGTCAAC
CGCTACCATAGGTCGTCGGCGAGCTATCCCGGTGCCGCCCCAGCCTACTCGATCTGGGCTGCGGCCTGGAACTCGTTCCTATCAACGGGAACACCCATATCAACTG
GTATCAGATTCAGGCGGTTCTGGTGCATCCCTTCCCATGGCTTCCGCTGAGGATCGCGTGGGGCATCTGGAGGCATCGGTCGCGCAACTGGCGGTTGCTGTGCAG
GACATCCAGTCCAACATAGCAGAGCTCGCCAAGGTTGTCGCCGACTCAGTGCAGCTGACGGGTGGCGAGGCGTCGGGGGTGGACGGCCGACACCGTCCTCAAC
AGCAGGGACGCCGGGACGGACGAGACGCCGACGCGATGGGCGGTGAGCGTGGCGGTCGTCGCGGGTTTCCACACGCGCCCCGTTACGACTTGCCGCTGTTCG
ATGGATCGGAGGACCCATTGCTGTGGCTGCGTCAGTGCGAGCGGTATGGGCGGCTGCATGACATTCCAAGGGAGGAGTGGGTGCAGATGGCCGGCATGCATCTG
CGCGGCGAGGCTCAGGAGGTTTTGGACAATTGGGAAACTGAGGATCAATTCATTTCTTGGCCGGAGTTCTATGACACCTTCTTGCAGCGCTTCACACGGCATTCA
ACCTCGGGACCGCTGGGAGATCTGGCCAATTTGAAACAGCACAACAGCCGAGCAGACGTATATGCAAGGGAGTTTCAGCGACTTCTTGCCCGCACACCAAGGTT
GTCTCGGCAGGATCAGATCGAGCTCTTTACCGCGGGGTTATCGTCCACCCTCGCGGCCGAGGTCCGTCGGGATCGCCCCCGGGACTTGGCTGACGCCATGGGTTT
GGCTCGTGAAGCAGAACGGCGGGAAGATCTCTTTCTTGCCAAGGTGGCCCAGGCCACGCGGTCACGCGGGTCTCGTAGCACATCTTGGGCATCGGGCACCGATC
CATCGGCAAGTAATCGGGCGACGGCGCCGTCATCCAGTGCCCTCCCGGTGGCGCCTGGGGGATCCAAGCTGGAAGGGGGTCGAGCGTTGACGACGGCTTCACA

GACTTCAGGGACTGTTCCCACTGCTCCACCATATCGTCGTCGTCTCAGTCCTGCAGAAATGTCTGACCGAAGGGAGAAGGGTTTCTGTTATAACTGTGATGAGGTT
TACTCGCGTGGACATCGTTGTCAGCGCCTGTTCCATTTGGAAGTGCTCAGCGAGGGTGAAGAGGATGATTCACTGGAGGAGGAACCAACCCTATCTGCTATAGCC
ATGAAGGGTCTGAAGGCCAGTCGCACTATGCAGGTCATTGTAGTCATGGGTGGACAACGTCTGGTGGGCTTAGTGGATAGTGGTAGCACCCATAACTTTATTAGTA
GCCATGCTGCTTCTGTGGTGCAGATCACACAATCGTCAGGGACTGGACAGCAGGTGCAGGTAGCCAATGGCGCTCGGTTGCCATGTCTTGGGCGGTTGAAAGAT
GTCCAGTTCTGCATTCAAACTCACATCTTCTCAGACTCCTTTTTGGTGATTCCCTTGGAAGGTTTTGATGTTGTTTTGGGGGTGCAGTGGCTGCAGAAATTGGGTG
ATATCAACTGGAATTTTGATTCCCTCCAAATGGCCTTCTCTTTGGATGGGGGCCACGTGAGTTTGCAGGGCATCACACCTCATGGGCGGCCAGTACATCTGAACAG
TCTTGTAGTGGGTAGAGACCCTCGGTTGCAGGAGATCTTGACTGCATTTGATGATCTGTTTCAAGAACCGCAGGGCCTGCCACCCTCTCGTCAGTGTGATCATCGC
ATCTGTTTGAAGGAAGGGTCTGACCCAGTGGTGGTAAGGCCGTATCGCTATCCCCACCGCTAGAAGGATGAAATTGAGCGTCAGTGCACCGACATGTTGGCACAA
GGTATTATTCGGCCCAGTCGTTCTCCATTCTCTTCACCCGTGCTTCTAGTTCGCAAGGCAGATGGTTCTTGGCGGTTTTGTGTGGATTATCGTGAACTGAATGCCACA
ACTATCAAAGACAAATTCCCAATTCCGGTAGTGGAAGAACTGCTTGACGAGTTGCATGGGGCTATATTTTTCACCAAACTTGATTTAAGATCGGGCTATCATCAAGT
CCGTATGCATGCTGCAGATGTGGAGAAGACGGCATTCCGCATTCACCATGGGCATTTTGAATTTCTGGTTATGGCATTTGGCTTGTGTAATGCCCCTTCTACCTTTCA
GGCCTTAATGAATGAGGTGCTCTCTAAGTTCCTTCGCAAATTCGTGTTGGTTTTCTTCGATGATATTCTTGTATACAGCCCCTCCTGGGAGGATCATCTGCAACATCTT
TCTTCAGTTTTGGCTCAACTCCGTCATCATCAATTATATCTGAAACAGTCCAAGTGTGCTTTTGCTCAAGCAACAGTGGGTTATCTGGGACATGTCATTTCAGAAAAA
GGTGTGGAAGCTGACCCAAACAAGATTCAGGATGTGGAGCGATGGCCTATTCCCATGTCAGTGGCAGATATACGGAGTTTCTTGGGTTTAGCAGGGTATTACCGG
CGCTTCATTCATCATTATGCATCGATCGCAGAACCTCTCACTACTCTTCTTCGCCGTCAACAATTCCACTGGTCTGATGAGGCGGCAGCAGCCTTCCAGCGCCTGAA
GCATGCTCTCCAAACTGCTCCAGTCCTCCGTCTTCCAGACTTCAATATTCTTTTTGTGGTTGAATGTGACGCCTCTAGTACGGGGATTGGAGCAGTGCTACAACAAG
AAGGGCATCCAGTGGCTTATTACAGCCGTAAACTAGCAGATCGACATGTGAAGCTTGCAGCTTATGAAAGGGAATTAATTGGGTTATCAAAGGCTATTCGTCATTG
GCGGCCATATTTGTGGGGGCGCCCCTTTGTTGTTCGCACTGATCATGCAAGTCTGAAATTTTTACTTGATCAAGCTTTGGTGACTCCGCCGCAACAACACTGGCTCA
ACAAGTTAATGGGGTTTGATTTCTCGGTGGAATACAAACCAGGACGGGCTAATGTGGTCGCGGATGCGCTGTCTCGCATCCATGACCCACTGGGCACATGTGCCG
CTATTTCATCCCCTACTGTGCTGTCAAGGGAGGTATGGGAATCAGAACTGAGGAAGGATCCGACCATTCAGCAGCGTGCTGCTCAACTTCTCACCACAGCCCGGG
GTCTTGAGGTAGTGCCCAATGGTAGTCTCTGGTCAACCCATGGGGGTCTTGTCTTCCGCAAAGATCGGTTGTTTATTCCAGCAACATCACCTCTTCGGCCCCAAGTG
GTGGCTGCTTATCATGATGTGACTCATGAGGGAGTGCATAAAACATTACATCGTATTAAGCAAAGTTTCTTTTGGGAGAATCTGCGTCGGTCTGTAGAGGAGTATGT
CCAGAATTGTCTTGTTTGTCAGCAGCATAAAGCAAGCACTCTAGCGCCAGCTGGCCTTCTGCAACCCTTGCCAATACCTCATCATATTTGGGCTGATATCTCAATGGA
TTTTGTGGTTGGTCTCCCAAAGTCTCAGGGGAAGACGGCTTTGTTGGTGGTGGTGGATCGATTATCAAAATATGGCCATTTTATTGCCTTCAAGCGGCCTGCTACA
GCCTCGGTGGTTGCTGATATTTTCTTTGACCAGATTGTGAGACTACATGGGATGCCAGAGTCAGTCGTCTCCGATCGGGATCCAGTGTTCACTAGTTCCTTTTGGCA
GAATCTTTTCAAGCGTTGCGGCACACAGCTCCTCACTTCTTCCTCTTATCACCCTCAGACGGATGGCCAGACAGAGGTGGTTAATCGTATTATTGAGATGTATCTTCG

CTGCCTAGCTGCCGAGAGGCCCTCCACTTGGGTCCACTGGTTACCATGGGCTGAATTTTGTTACAACTCCTCCTATCATTCTGCTCTCAGACTGACTCCCTTCGAATT
GGTGTATGGCCGCAAACCCCCTCCGGTGGTTCCTTACAGCCAAGGATCTACCCTTGTGGATGCGGTTGATAGCCAGCTGGTGGATCGTGATGCCTTATTGGATAGA
GCTCGTCATCACTTGTTGACTGCCCAGAACAGGATGAAACAGCGTTATGATCTCCATCACCGTGAGGTCTCTTATGCTGTGGGTGATTGGGTGTGGTTAAAGCTCCA
AAGTCATCGTCAACTCTCGGTTCGCCCGGATCACTATCACAAGTTGGCGCCCAGATTTTTTGGACCTTACCAGGTGGTCAGTCAAGTGGGCAACGTGGCATATCGT
CTCGCCTTACCAGCAACAGCTAAAATACATGATGTATTTCACGTCTCCATGCTGAAACCTTTCAAAGGGCCTCCTCCATCCACCACACCAGACCTTCCTGAAGATGTA
TCCTACCCCTCTCCAGAATCTGCACAACCAACTCCTCACAAGATACTGGCTCAACGCTTTGTTCACGGGCACCGGCAGTTCTTGATTCAGTGGACCGGACAGGATC
TTGAAGAAGCTACTTGGGAGGATGCTCTGGACTTCCGTCGGGCGCACCCAGATTTCAAGCTTGAGGACAAGCTTGCTGTCGAGGGGGGGAGTATTGATATGGCC
CAGCCGCATAGAGTTTATTTTAGGAAACACTGAGTTGATTGGCTGTGATTATTTGGTAGCAAATAGTCCCTTTTGTAATGGTAGCATTAGTTACCTTATTGAGCATTAC
ACGGCTCTATAGTTTCTATGTTTAGGAAAGAGATTGTGGGGAAATAAAAGGGAGAAAGGCAGAAGAGAAAAGGGACATTCTTGTTCCACAAATTCCAGTCTCAG
GCGTCGCCGACGATAAACGGCCGGTGGTTCCGTCAACCGCTACCATAGGTCGCCGGCGAGCTATCCCGGTGCCGCCCCAGCCTACTCAATCTGGGCTGCGGCCTG
GAACTCGTTCCTATCAACGGGAACACCCATATCACTTTTCTTCTTGTGGTAACAGGCAGGCCTACATATGAAAGAAGGATGTGTTGGCATCTCCCTCTTTCAAGTATT
GTACTCTAGATATGAGTCTTGCTATGGTTCTCTCTAGGGAAGCAAGGACCATGCACTGATGTTTGAGTTTTCTTAATAACCATAACTCTTCTGGGGTTAAAACTCTGC
TGTCCTGAGCCATCTCCAATCTGTGTAATATCTCTCTTGCTATGCCTAGCTGGGATTTAACATTGCCCACTGTTTTATGCCGCAATGATTGTAGGTCTCTGGTTAGCCTT
TTGAGCTTTAGAGAGATCCTCTCCAGATGGCCACAATCCTGAGTTGTCTGGTTCCAGGAATTTGATACCACCTCTAGAAACCCTAGCATTTTGGGCCAGAAGATCTC
AAAATGGAATCTCATCTTCCCCTTATCTTCTTTCAGTTTTAGGACTAATGGACAATGGTCAGAGTCCTCTGAGGCCTTACTGATTAAAATATAATCTGGAAACAGCTCC
TCCCAGCCAGCTGTACAGAATAGATGATCTAGCTTGACAAGAGTGGGAACCTCTCTCTGGTTGGACCAAGTATATTTTCTTCCTATCAAAGGGATTTCCTTCAGTTCC
AAATCATTTATCCATCTTCTGAATCTTCCCAGCATGGCCCTGTTGATATTGGTGTTATTCTTATCTTCGGAGCTGTAAATCTGGTTGAAATCACCTGCCAAGATCCATGG
TCCTTCACAGCCTACCCTGATTTCTCTTAACTCTTGGAGAAAATCTTGCTTAAGGTTATCCTGGTGTGGCCCATACACCCCTGTGAACCACCATTGTGGACCTGAATT
CTCCTGTAGTTGTATGGAGACTGAGTACTCTTTTACTAAAGGATTGCTGGAAGTAAAGACCCCATCTCTCCAAGCCACTAATATGCCCCTCTTGCTCCCTGTGCTAGG
CTAAATACAAAACCACTGTAACCAGTACCAAGGATGGATAGGACATCAAATGCTGAAATGTTGCTCATTTTGGTTTCTTGCAAGCAGACTAGAGATGGTTTGATATC
CAAGATCAGGGTCTTAACATTATCTCTCCTAGCTCTACTGTTCAATCCTCTCACATTCCAAATCAGGAAGTTGCACTGGTTCATTGAGAAAAAGAAGCTCGACCAAAT
GAACAGATCTTAGTTTATGCCAACTCAGGTGCTTCCCACCCAAACAGAGCTGCTAAGGCAGCAATGTGTTCTCTGGAGAGGGGTGAATTGTACATTACAACATATTT
GTTGGCATCATCCATGGAGATGTTGTCTTGATCATCACAAAAACCCAACTTCCTACATACATTGGCTACTGCTTCAGTGCCTAGCTCAGGTGGAATCTTGGCCACCCT
TCTAGATCTCCTAGGCTTGAAATTGCTTGGGAGGGTCTTCTTTCTGTGCTTGGGGATTGAAGGAGTGATCATCAAACCATCAACGACCTTGATTATGTGTTGTCTGA
ATTCTTGTAGGGGGGTTGAGGCTGTCGGACTCTCTACCTGAGTTCGTTCAGGTAGGATTTCTCTTCTTCTTGAGTATACCTTATAGTATGGGGGCTTTGCTTGCAATCT
TTTCTTGATGCTTTCCCAGGGGTCTCTAGTCTGCTGCACAAGGATAACAGGAAGCCTTAGAGGTTCCTCTCTATTTGGCAATTCCTCATTGTTCATCAAAGTTGTAAC

ATTCTCTGCTGCTCTAACACTTGGGCACACTGGGTCCTGCCTAACTGGTGTTGAGTCTTGGTCTGTTTCTTCAGCACTTGGGCACACTGGGTTCTGATCAACTGACT
ATTTGCCGCCAATCGCCTCCATATTCGGTCTCTCCTCTGAAGCAGTGTCATTAAGGACCATTTCATTGCATTGGGATGGAATTGGAGGGGCTAGCTTCATTAATTCCA
CGACAGATGGGTCCCCCGAACCTTCCTTGTATGCTGGGACATCACAATTGAATGAGGCGGGCTCGGCTACTTCAATTGGACGGGTGGTTTCAAGGCTGGGGCGTC
AGGCGAGGCTTCAATTGAAGAGGTGTACGCCACTGGTGGAGAAATCGAAGCGGGCGCCTCCACCGAGGAGTCCAGTAGCGCGTCCGCTTCAGCTGCCCCACCA
ATCGCCACACTGGCTTTCTAGCAGGAGACGATAGCTGCTTTCGTCGTCGCTTGCCGTGGTCCTGGTCGCGATCGTCGCCGGAATTAGCTGCCGGCGGTGGCGGGG
AGGGGCTCCCGCCCATGGACGCACCGATCGTATCACCCGCATGGAGATTGGATACACCAGCGTGCGATGACTCAGGGAATCGTCACTGGCGCCTATCTGCGACTCC
TACGCATGAAGATCCAATTCCACCGCCACTTGCTCCGACTTGTCACACCATGCTCTCAGCTTGAACACCGATAGTTTGATGCGTTCCTCAGACTCTGGGTGTACGTCT
CGGATGAGGCAGTGATCATCGAGAAGCTGCTCCGCCGTTTCCAACCCCCAAAGTCGTGCTGGGATTCCTCTCAATTCGATGTCAACTAGCACTGGCAGAGCTCCCC
CGCTGACGGCCATGGCTTGTCTGGACCATGTCTGATGTGAAGACGGATGGTGGGAAAGACGAAAGGCTGGTCTCCATTGTAGACCTGGCATGCTACCACTTCGTT
GGGGAGGAATGCGACGCATCAATTCGATGCAGCACGACGGAGGTCGAGCGTCTGTGCCTCGAGGTTAAACCTCACGGTGAGCGCGTCAGACACTTCCATGGCGC
AACCAAAATTCACTTGGCTAATGACGGAGATGATCAGGGCCCGTCGAAGATTATCCTCTGCTTGCATGATCTTCCCGGTGTGTCTGATGATCCACCTAGGCCCGCCT
TCCTCTTGAACATGGCTCTGTTCTGCCTGCCGCTGGGGGAGTTCAGCGACTGCTGCTTCATGATCATCGTTGCCACTCCGACTTTCGTTCTCTTCCTCCCCTGTTCGC
GGCGTGTGCGCCTCTTCTCCCTCCCGCCGCCTCCATCTCCTCCAACCTCTACCTTGTCCGGTATCTTGTCCTCAAAGCCCGCCATCCCGGCAACCTGGGTGATTGGAC
GCCACACCAGGGAGCGTCTTGGTTGGGCGCGCCTGACCGCCGGTGGGGGCACTCGTAAGCATGATGCTCTGGCAGCCGACAACAGAAGCACCTCACTAGCCGA
TGACACTCAGTCGCGTGTTGGGTTGAGGAGAAGCAATTGAAACACTTCCCCCTAAGGTTAGATGACACTATCTTAGGAAGAGGAGGGGGTTTGGGAGGGCGGA
GGCCATTATCTTCCACTTGGCCTTGGATGGGAGAAGGCTTGGGCAAGCGCAGGGTTTCCTTGTATGACGCTCTTCGCCGAGTGTACTTCGGGGTAACAACGATGG
AGGAGGCTGGGCTCCTGTCGTCGCACCACCTTAGGGCCTTGGAATGACCGGTAGCCAACGGAATAGGGGATGTCCCCGACGCAAGGGAGAAGGGAGTGGCCCC
CGAGCCTGGAGTAGGAGTAGGCACCAACGTGGGGCTGTGGGGGGTTGAGGCGCTGGCGAGGGAGGGGAGTTTAGGGGTCGGGAAGCACATACTGAAGGCTT
GTCAGGTTGCTGGTTACTTTTTTCTTGTAAGTAAATATCCCTAGATACATATGTAAAAAAGGAACTATGAATTTCATGTACTTTAGAGGGCTCCAACACTTGACTTTTG
TGTGCCTCAGGTTTGTGTTTTAGGGATGGTGTGGCTGAATGGAAACTCCATCTTATGCCTTGCACCAGAAGTGTTTGCTAAAGTTAATAAAAAAATATTGCTTCTCG
ATTAGTTGCCCAGGATCTTCACCTCAAATCGTGGGTTAGAGACATCAGCTCACCATATCAACTATGTCAAATGTGATATCCTCTGTTCTGGTGTTGTTTAGCCGAAGG
TTACTGGCATACTTATTTTTCCAAGTGTTACTATTTGCTTCTCTTCGAAGCCACAAAGGGGATTCATGGCTTGCATCCGATCATCAGGTTCTTGCATCTGCATGAAAGC
TTTTGCAAAAATGATAGCTGCCCAACTGCCTGTGTCCACTAAGACATTGTGGACCACAAAACCTTTTATGACACATGATATCACCATGACATCATCGTGTGGGTAATC
CTTGAGGTAAAGGTCTTCTTGAGAAAAAGGTGATTAGGATATGAGACCATTTGGATCTTATGTAGGGCCCTTGGATCCCAACATGCTGCATCCTTCTCTGCGCTTCC
TTCTTCTGCCTCTTGTTGCTGGGCACAAAGCTTGATCCTCTGTGATATGGAGGATAAGTTGTTTTCGACCTTGTGAAGAAGCACCAAAGCTAGCGTTGCTTGTTTTG
TTCTTCGAAGTTGCGAGTTAATTGGAAGTGGGCGCCAATGTTGGTCACCTATTCATATTCCTATGAAGAACCAAAACAGGGCAAGACAATTAAAGTAACGATGCCT

TCCTTTAATCCACTCCTTGTGAAGAGGTTGACTAGAGGAAGAATGTCTGGATGGACGATAATAAATAAGGAAACAAAGGTATAAAGACATCCGATATAAGTCGATAT
GTGTATTCTCTTCTTATTCCTTTGTCACGAAAGGTTACAATGATACCTTGGAGAATCCAGACAATGGTCCGAAGCCCTATACATATGAACTGCAAGATCTACTGAGGG
TATCATTGTCTTTTCCTTCCAAAGACGTAAGGTGCACGTCACCTGATCTCCACTTCGCCTCCCTGTTGTTGTTCCAGATCCGCTAAAGAGCTTTGGTTAACTGTTGAT
GTCGAAGCCTTCACTTCTGGTTAGCAACCTTCGGTTGAGGCACGCCGTGACGAAGGTTAATCTGAAAATAGATCACAAAACAACTTGAAACTTGTTAGTGTGAGG
ACCTTCGGCTAAGGCATACTCCTAACACCCATTAGACGATCAAAGGCTGAGTAATACTCATCAATAGACATATCATGTTGCTCAATGATATGAGTTTGCTGCATAAAG
GTGTGTAAGAGTGTACCACTATCCTGAACAAAGCGTTCCTTCAAATGTGACCAAATAGCTTTAGCAGTTGTGAATTTGACAAACTCATTATCATGGATGGTTTGCTAT
TCACTATTGCAACCATAACCTTGCCATCATTAGTTTGCCATGTTTTGGTCGCTGTAGCATTGCTACGATCATCAGCAAGAACCGGTGGAGCTTCAGTCAGGTGAAAG
AGTAAACTACGAACTCTCAATACAGTCTGAACACAAAAGGCCCACTCGGGGTAGTTCTGACCATCAAACACGATATTGACAACTATAGTATTGATTGACATAACGAT
GAAAAAGCAACTGAATTTATAAACAGAGAACTGAGTACCAAATCACCGAAGGCAGATCAGATCCAACCGGTTACTAGATGTTCTGAGTTGGAGCACATGAGTGTA
GGGTACCAGACTGTGCACAATCCTGACAAAATACAACTGGAAAGTCCCAGCAGCAGCAGCCCCAGCGACAAGACGACTATGGTGAAAACGTTGCACCCGACGAC
CAACACAGCAAATGACAAAGGAGGTGGCCAAAGTGCCCTACAACCAGGACAGATTGGTTCTAGTCGACGAAGAACTTTGGTCCAGTTTGACCAGATGATGGTGC
GGGAGCCCCAAGCGGACGCGAGCGCATGCTAGAGATGAAGGCGTGCAACGATGCTCCCCGGCGAACGCATATCGAGCGGGGAAGATCTGTTGGTGGTGGTCGC
GACACCCAGACAGACGTGCTGCTGAGCGGTGTCGCCGCAGGTGGTGAGGACGACCCGCGACAGATGTGGCGCGACGACGCCGAGGGTGAAGCGACCGCGGG
AAAATGATCGCGACGACCGAAAAAATGGGTGGGGTTAGAGGGCGGGATTAAAGCTAATCTTAACCCTAACCTGCCTTGTTACCATGTTACCTTTGGAAATGACGA
AGGATAATAAGTCCATAGGGGTTGTAGAGATCATATCTATAGGCCAACATGCCTTAGCAATAGGATGAGCCTAATGGGCCTTAACAATATGAGCTATAGATCTTAAATT
TTGTGGCTGAAAAATAGTGCGTTACTAGCCCTAAAACACCCAGGTGTTGTATAGACATTCTAAAAAATGTAGTCATGTGGTAACTGTTTGATGTATATAGATGGGTCT
CTATGTTTTCTGTATTTCCCCATTGTATAAGGGGTTTAGTGCATATTTACAAGTGCTATTCATAACATGGTACCAGAGACAGAAATTAGGGTTAGATTTTTTCTCTAAAC
CTAATCTTTTCTTTCCTACGCACTTGCCTGCTCGCTGTGGTTCTGTTCACATTTCGGTGTCTTCTTGGCGACTCTCCAATCGCGTGGCTTCATACAGGCCATCGCGTTC
GTCGCCTCGTCATCCACGACACCGCACACTCATCTATTGCATGTTGTAGAACAGTAGTCATAAAAGCTGAAGGATCGTAACGATCAGTAGGATGCCGAAGACGATTA
CTACTTCTAAGAGACGACTCTGGTGTAGAAGTAGGAGAAGAAATCAACTCAATAGGAGAGGACAAATCAATAGGAGCAAATGACATGATTATTACAGGGGAATAG
GGGATGATCCTCAATTTATTACCTTTTTGTGAAGGCACGTCTTAGTGAATAGTTTCTTATGTCCGATCTTGGTCCTCTTTTGTTTTTTCTTGGGATTGAGGTTTCCTCTA
CTTCGGAGGGCTTCTATCTCTCTCAGAAGAAATACATTCAAGGGCTTCTTAATCGACTTCTCTTACTAATCACCGTACTATCGAGACTCCCATGGAGCTCAACGTTTG
TCCTCGTGCCACTGATGGTAAGCCTCTTTTGGATCCTACTCGCTATCGTCACATTGTAGGTAGCCTAGTCTATCTTGGTGTTACTCGCCCTGACTTTTTGTATCTTTGTG
CATATTCTGAGTTAGTTTGTTTTTGCCCCCACTTAGCTTCATTACAATAATCTTATTCATGTTCTATGTTATCTTCGTGGGATTATTTCTCGGCGTCTATTCTTTTCTTGCT
CTAGCTCCTTGCAGCTTCATGTCTACTGTGATGCGACTTGGGCTAGTGATTCCTCACATCATCAGTCTCTTTCTGTCTATTGTGTTTTTTGGTGGTTCCCTCATTGCTTG
GAAGACTAAGAAGCAGTTAGCAGTTTCCTGTTTGAGTACAGAGGCTGAGTTGCATGCTATGGCTCTTGAGGTGGCAGAGGTGACTTGACTATGGTGGTTGCTTGT

TGATTTTTGTGTCTATACCGATTCTCTTTTTGTCTGACAGTACTGGTGCTATTAGCATTGCTCGTGGTTCAGTAAAGCATGAGCTTACTGAGCATATTGGTGTTGATGG
AATAAGTCATGTGGTCTTTGTGTGGAGGACAACTCTGCAACAGCATGTGTAGGATAACATGGTGGAGGACAACATCTCTGCAGCAGTCAAAAGCAACAGTCTTTT
GCTGTAGTGTGGCAGCAGCACACACTCCAGGCAGTGCAGTGCACAGCAGCAACAGTGCAGTCTTTTTGACTGCGCTGTGGCAGCAGCAGGCAGGCAGTTCAGT
GCAGTCTTTTGACTGCACTTTAGCATCTAGAGGACAGCACATATGTGCTGCAGTGTGTGGTGTAGTGTGTAGTGTATTCTAGTGCAGCCCCGAGGGCTATAAATATG
TGTCCCCAACCCTTCTAAGGGTATGGCATTGTGTAGTGTTTGAGGAAATAAACAGAAAATTGCCCCAACTCATAGTGTCATCCTCTTGATGAGAGTTAGAGTTCCTC
TACTTACAATTGGTATCAGAGCCAAACTATCCTGCAGCCTAAACATCTCGTGCTCATCTCCTTCCCGTGCCTCTCCCCACACTCAGTCGGCAGCAAGAGCTCCAACTG
TTCCTTCCTCTCCTGCTCAGACGAGCAGCCTTTCGTCTGAGACAGCCTCTTCCACACGCAGCGACCCCTCACTAGTCCACCATGTCCCTACGCTCGGTCACTTCGAG
TGCGCGGCGCCAGCAGGAGGCCGAGGTCACCGCGGCACAAGAACGCGAGCGAGCAGCAGCAGCGGCTGCAGCGACAGCGGCGAGGGCAGCACGGCTGGCG
GCGGCGGAACTGGCAGCAGCGAGAGCGGAAGTAGAAGCAGCGGAGGCGGCGGATGCTGCACGTGCGGCGGCAGCAGAGCTCGAGGTTCTGCGCGGCAGTA
GAGATGGCAGCTTTGCTTCTGTCGACGACAACACCGACGAAGAGCTCAGGCTGGCGAGGGAAGCAGCGCGAGAGCATGCGGCACAGTGGGCAGCCGCACATC
CCCATGGGGGCGCGCGTGGCGGCAGCCCAGATAGGCGCCAACGCGCTGACGGCGCTCCGGGCGAGGGCGCACGCGGCGGCAGCCCAGACGGGCGAAGACGC
GCCGGCGGAGCTCCCGGCGGCGGCGACAGAGATCGCGACCTCTACAGGCGGCGCGACTCTCCCTCCCCGGATCAGTACCATGGTCGCCGCATGGCCCAGGCCAT
TGTCAGGGACATCGGTTCCGGCGGTGGGTGGCCTACCCTCACCAAGACCAACTACGTCGAGTGGGCCGCGGTGACGAGGGTACGGCTCCAGGTTCGCCACATGT
GGGAAGCACTTCGGTACGGCGACGTCGACTACCACGAGGATCGACGGGCGCTGGATGCTCTCATTGCTGCAGTCCTGCCCGAGATGCAGTTTTCGCTTTCCCAGA
AGCGGACTGCCAAGGAGGCCTGGGACGCCATCGCTGCGACCCGCATCGGCAGTGACCGTGCCCGCAAGACCACACTGCAGGGACTTCGCAAGGAGTGGGAGA
ACCTGGCCTTCAAGGCAGGTGAGGATGTTGATGACTTTGCTCTCTGCCTCAACACTCTGTTGCAGAAGATGGTGCAGTTCGGCGACGACACCTACGATGAGGAGA
GAGCTGTTGAGAAGCTCTTCCGTTGCATCCCCGAGAAGTACAAGCAGATTGCTCGCTCGATCGAGTCTCTGCTAGACCTCTCCACGATGACGATCGAAGAGGCGA
TAGGTCGTCTCAAGGTGGTCGACGGCGACGAACCACAGGCTCCCTCTGGGCCTATCACTATCGGCGGGAAGCTACATCTCACTCGGGAGCAGTGGGAGGCCTGC
CAGGGTGACAAGAAGGGAGAGTCCTCCTCGACACGAGGCCGCAAACGCGGCAAGCCGTGCAAGGCGCGTGGAGGCGCCCAGGCCGGGGCGCGAGGACATGC
TGAGGGTGGTGCCCGTGGAGGTGCCCAAGGCGGCGTCGTCGGCAACAAGAAGCCGGCACGAGATGACGGCTGTCACAACTGCGATAAGCTTAGCCACTGGGCC
AGGGACTATCGACAGCCACGACGTGGCCAAGCCAACGTCGCACAGGCGGAGGCGGAGGAGGAGGCTCTGCTCCTAGCACATGCAAGCATCGAGCTATCTCCAG
CGGCAATAGCCGCAGCGGCACTCCTCCACCTTGATGAGTCGAAATCACGCGCTTTCCTCGGCGACGGCTCCAGCAAAGACATGATCGAAGGATGGTGCCTCGACA
CCGGCGCCACTCATCACATGACTGGCCGACGGGAGTTCTTCACCGAGCTTGACTCTACCGTCCGAGGCTCTATCAAGTTTGGGGACGCCTCCGGCGTAAAGATCA
AGGGCGCCGGCTCAGTCGTCTTCACCGCCGCGTCTGGTGAGCACAGGCTGCTCACTGGAGTCTACTACATCCCCGCGTTGAGGAACTCTATCATCAGCTTGGGAC
AGCTGGATGAGAACGGTTCGCGTGTGGAGGTCGAGCACAAAGTCATGAGGATCTGGGACCCCTCTCGTCGCCTTCTTGCCAAGGTACGCAGGAGTCCAAATCGG
CTATACATCCTCAATGTGAAGGTGGCACAACCTTGCTGCCTTGCTGCTCGTCGAGACGACGGAGCATGGCAGTGGCACGAGCGCTTCGGGCACCTTAACTTCGAG

GCCCTGAAGCGGCTTAGTGCCAAGGAGATGGTACGAGGCCTGCCGTGCCTTGACCATGTGGAGCAATTTTGCGATGTCTGCGTGTTGACAAAGCAGAGACGACT
CCCCTTTCCCCAGCAGTCGAGCTTCCGAGCCAAGGAGAGGCTCGAGCTCGTGCATGGGGACTTGTGTGGCCCGGTGACACCAGCCACACCAGGAGGACGACGC
TACTTCTTGCTGCTCGTCGACGATCTCTCTCGCTACATGTGGGTGATGATCCTTGGTAGCAAGGGAGAGGCTGCGAACGCCATCAGGCGTGTGCAGGTCGCTGCG
GAGGCGGAGTGCGGCCGCAAGCTGCGCGTGCTGCGCACCGACAACGGCGGCGAATTCACGGCGGCTGAGTTCGCGTCGTACTGCACGGATGAGGGCGTTCAG
CGCCACTACTCCGCGCCGTATAGCCCGCAGCAGAACGGCGTCGTCGAGCGGCGCTACCAGACGGTTGTGGGGATGGCTCGGGCTGTCCTCAAACAGAGGGGAA
TGCCGACTGCCTTCTGGGGAGAGGCGGTGGTGACAGCGGTCTACATCCTCAACCGCTCGCCCACCAAGGCACTCAACGGGATGACACCGTACGAGGCTTGGCAT
GGGCGCAAGCCGGCGGTCTCTCACCTACGGGTCTTCGGCTGCCTTGCGTTCACTAAGGAGCTTGGCCACATCGGCAAACTCGGCGACAGGAGCACTCCGGGGG
TGTTCATTGGCTACGCGGAGGGCTCGAAGGCCTACCGCATCCTTGACCCAGGAACACGGCGTGTGCGCACGGCGCGCAACGTAGTGTTTCGACGAAGGGCGAG
GATGGGCGTGGGACAAAGCGGTGGACGACGGCACGACTCCGACGTACGACGACTTCACCATCGAGTACGTCCACTTTGAGGGAGCTGGGGGAGTAGGCAACTC
TTCTCCGAACAGGTCTACCCCAGCCCGAAGTCTCCACCGACTCCAGCGCCACGCTCTCCAGCTCCGGCTACAACGAGCTCTTCATCGCCACGCACTCCAGCCACGA
CTCCGGCTACAGCGAGCTCCTCACCACCACATACTCTAGCACCGACGGTACCCTCTCCGGGAACGTCCTCTCCGACACCAGCTCGTGTCGAGCACGACCCAGTGGA
GCTCGTGACCCCGCTCCCCCACGACGAGGAGCGTGTCGACGCGTGCTACGACGGCGAGCTGTTGCGGTATCGAAGGGTGGAGGGCCTTCTCATCGACCCGTCGG
TGCCGGGCCCGACGTCTCGCATTCTAGCAGGAGAGTTGCATCTTGCATGCGAAGATGGTGAGCCTCGGTCTTTCGCGGAGGCCGAGAAACATGCGGCTTGGCGT
GCCGCGATGCAGTCGGAGATGGATGCGGTTGAGACGAACCACACTTGGGAGCTCGCTGATCTCCCTCTTGGTCATCGCGCGATCACCCTTAAGTGGGTGTTCAAA
CTGAAGAGGGATGAAGCCGGTGCTATCGTCAAGCATAAGGCTCGCTTGGTGGCACGCGGTTTCTTGCAGCAGGAGGGGATCGACTTCGACGATGCTTTCTCCCCT
GTGGCACGGATGGAATCCGTGCGACTCCTCCTCGCGCTGGCAGCCCAGGAGGGCTGACATGTTCATCACATGGATGTCAAGTCGGCGTTTCTTAACGGCGACTTA
AAGGAGGAGGTCTACGTACACCAACCGCCAGGTTTTGCGATCCCTGGCAAGGAGGGCAAGGTGTTGCGCCTACGCAAGGCTCTTTATGGCCTGCGACAGGCACC
GAGGGCGTGGAATGCCAAGCTGGATTCCATGCTCAAAGGAATGGGCTTCACGCCAAGCCCGCACGAGGCGGTCATCTATCGGCGGGGCAATGGAGGAAGTGCC
CTGCTGGTGGGTGTCTACGTCGACGACTTGGTGATCACCGGCGCCAAGGATGCAGAGGTGGCAGCGTTCAAGGAAGAGATGAAGGCCACCTTCCAAATGAGTG
ACATGGGGTATCTCTCCTTCTACCTGGGGATTGAGGTGCACCAGGGAGACTCCGGGATCACACTTCGCCAGACTGCCTACGCCAAGCGTATTTTTGAGCTGGCTG
GACTCACCGACTGCAACCCAACTCTCACTCCGATGGAGGAGAGGCTGAAGCTGAGTCGCGACAGCACAACGGAGGAGGTGGACGCTACACAGTACCGTCGTCTT
GTGGGGAGCCTTCGCTACCTCGTCCACACACGGCCGGATCTAGCATACTCTGTCGGCTACATTAGTCGGTTCTGTAGCGACCGACGATGGAGCACGAGCAGGCTG
TGAAGAGGATCGTCCGCTATGTTGCGGGAACTCTTGATCACAGTCTCTACTACCCGAGGTGCCCTGGGGAGGCACACCTTGTCGGGTATAGCGATAGTGACCACG
CCGGCGACATCGACACCAGCAAGAGCACAAGCGGGATCCTCTTCTTCCTCGGCAAGTGCCCCATCAGCTGGCAGTCGGTCAAGCAGCAGGTGGTGGCCATGTCC
AGCTGCGAGGCCGAGTACATAGCGGCCTCCACCGCTTCAACTCAGGCGCTCTGGCTTGCTCGCCTGCTCAGTGGTCTCATCGGGAGAGACGTTGGAGCAGTGGA
ACTTCGGGTGGACAGCCAGTCCGCCTTGGTGTTGGCCAAGAACCCCGTGTTCCATGAACGGAGCAAGCACATCCGGGTGAGATACCACTTCATCCGTGACTGTTT

GGCAGAAGGGAGCATCCATGCACGCTACATCAACACCAAGGATCAGCTTGCGGACTTGCTCACCAAGGCCCTTGGGAGGATCAAGTTCCTTGAGCTTTGTTGCA
GGTCTGGGATGACTAAACTTTCCCACAAGACGACGCACAAGTCTTAGGGGGAGAATGATGGAATAAGTCATGTGGTCTTTGTGTGGAGGACAACTCTGCAACAG
CATGTGTAGAATAACATGGTGGAGGACAACATCTCTGCAGCAGTCAAAAGCAGCAGTCTTTTGCTGTAGTGTGGCAGCAGCAAGCACTGCAGACAGTGCAGTGC
ACAGCAGCAACAGTGCAGTCTTTTTGACTGCGCTGTGGCAGCAGCAGGCAGGCAGTTCAGTGCAGTCTTTTGACTGCACTTTAGCATCTAGAGGACAGCACATAT
GTGCTGCAGTGTGTGGTGTAGTGTGTAGTGTATTTTAGTGCAGCCCCGAGAGCTATAAATATGTGTCCCCAACCCTTCTAAGGGTATGGCATTGTGTAGTGTTTGAG
GAAATAAACAGAAAATTGCCCCAACTCATAGTGTCATCCTCTTGATGAGAGTTAGAGTCACTCTACTTACAGGTGTCGATGCTTAATATACACGAGCATAGGTTTAGG
ATGATATCATTGATCTTCGCCTTTAGAGCTTCAGCTGGCTGATTTCTTCATCAAGACACAAACTAGAGCTCAACATTGATTCTATCTCTTCAAACTCAATGTGGTTGAC
CCACCTTGAGTTTGAGGGGTTGTTAGATGTATATAGATGGGTCTCTATGCTTTCTGTATTTCCCCATTGTATAAGTGGCTTAGTGCATATTTACAAGTGCTATTCATAAC
AGTAATTTACATTTCAAAGGAAGCACATGAATATTTAAATGGTGTTGCTCTATCTTAACGCTTTTATGCAGAGTAAGGACAATTGGATCAATTTAAGTAGGTGAGTGG
AATTGATCCTTTTGAAGTTTCTGACCCCAGTTCAATTCTACAGGTTTTCTCGTGTAAATTTGACACTACTTTGGCTGCTCTTTTAGGTCTGGGCACACAAATTTGAAC
TATTAACGCATTGGGAGTGCTTGTGTGTTAGAGGTCTAGGAGGAGCTATGAGGTACTACTTCCGTCAGGTCCAGCGTTTGGTTGGAGATATGGAAGGAGGAGCTC
TCTTGTTTATAGATGACCTCAACTCCAAGTGACAAAGTGAACAGCTGTTGAACTTCCCGTCTATTTCTGTTTCTTTTTGCTGGATTTGGAGATGGAGGTGAATTCCTC
TGGTGAGGAAACGGTGATAAAGGTGAGTTGTTCTACAATGTGCTTCTTTGATTTATGGTAAAAAAGGAACATTTTATCACTGTTCTTTGTTGGAAACAAAATCACTT
TGTTTCTCTTATATATTAACACAGGTAGTTTCACTTCACACGGCTTGACTGACCTTCATAACCAAAGCTACATAAAAACTAGCTTTCTATACATTATACACTAGCTTGAT
CTTCACAACCAAGCCCTATCTTCTCTATCTGGCTCACATAGCTTCAACTTTTGTTGGGCTGCAATAATCTGCTGCTTGCTGGTGCCTTTACCTCTTATTTATAGCTGAAA
ATAGAGTAAGAAAACATCCCTAGTGGCTTCCCTAGAAATTACCTAGTGACTTTCCTAGAAACTAAGAGGAAGATTAGAGTGTGATGACTCACTTCACTGTACAGATT
TTGACTCATTAACTAGACCCACTAACAAAAGACCATCAGCAGCTAGGATATGCTACACTGGTTTCATGTCTTCATCCATACATGACAAAATTACTCCTACATTCTTTGT
AGGTAAGGAAGCCATACACAATAACCAAGCAGCGGGAGCGGTGGACAGAGGCTGAGCACAAACGGTTCCTTGAAGCCTTGAAACTTTATGGCAGAGCATGGCA
GCGCATAGAAGGTAAAATATCCATAGCGGCTGTATCCAAGTAGTTTTATGCTATTATTTCCTGACTTCATCACTTGTAACCACTGAGGATTATTCATGTGGATTTAGTTT
TCCAATTGATACTGATTGCAATTTTTTTGTTTCATCAATCTCCATAGAGCATGTTGGGACAAAGACGGCCGTGCAGATCAGAAGTCACGCTCAAAAGTTCTTCACCA
AGGTTCTTTCTCTATCTGATTGGCTAGTGCTCTTGTTTGGTTCAGTCTAGTTGCATTGTCTTTATATTTGCTGATAATGCTAATGACTTTTGTAGTAATTTCCAGTTTTTA
GTGTCTTTACATTAATGCATTATTTTTTCTACTTACATATGTGTGCCACAGTGATGGGTTTGAAAAGTATGAGCCATGTATGATTTATTTGTTTTGACCTCAGTGCTCTAT
TTATTAACTGATCAAATAAATCCATATCGAAATTGAAAAAAACATGAAATTTGCTTATACCTGGTCCATTTATTTACGTATGCTTGTTGTCACTGAGCTTATAATCATCTA
TATTGTGTAATATGACCGAAGTACCAATAGTGGGATGGAAACTGTAGGAAATTGTAGTACTTGGTCTATTGATAGGAGATGTGGTACAACTTATAGACTCACAGGAC
CCTAACCCTAATGGGCCGACAGCCCAACAGTGGTGTCGGCCCACTCACACACACAGTCTAACATCCCCTGCAGTCGCAACGGGGGCACCACACATGATGAGACTG
GAGTAGAAGGCGAAGGTAGGAGCCGACGGGTTGAAATCCCCCACAGTCGCAGCGTCGTGAGGGTGCGGATGTTGCGACTGGAGTAAGACCGATGTGTGCTCCA

AGAAGACGATAGCCCTTAGATGTCGAGGTAGCCGAAGCCGAGGTGGTCGCGGTCGGGAGACACGCAGCAGAAGCCTGTTCTTCGGGAGGGGTCGACGTTCGA
GCGTCAACGATCGGCGAGGGCGACACAACAAAAGAGCACCAGCAGGTCGACCTTCTTACTTCTTCGATTGTCCGGACGTTGAGGAGCCCCGCCAGGAAGGCCG
ACGACAGCGCACGCATCTGCGCTGGTCATGGTGGTCGTGCCTGCGACAAAATAGAAGGGGTAACGGCGGATCCGGCAGGGAAGACCACGACAATGACAGATCC
AGTCGGGAAGACGTGATCCGGTCAAAGGGACGGCAGATCGAGCCAGGAAGGTCACAACAACGGTGAATAAGGCCAGGAAGGCCGTCGCAGCGACGGATCCAA
CGAAGGCGATGAAGGGGAGGGCGACAGGGTGGTTCCAGCCGGGTAGGGGCCTGCATCGGTCCTGTCAGGAAGGGTTGACGCCGGCGACGGGAGAGGCGCG
AAGAGAGAGGCGCTGCTAGGCGGCTCAGGCGAGGGCAGGGGGCCGCGTTGATGGCGTCGCTGATCCTAGAACTGGCTAGCCAAGGGGGAGAGGAAAGAGAG
CCGCGACCAGGAAGGTGGCGACGCGAGCCTAAGCGAGGTAGAGGCGCTCGACGGCGGCGCGACCTGGGGTCGTGCGGCGCTTTTGCGGCCGAGGGGAAGGG
AGGGGGGCCTCGGGCGACGGCCTCCATGGCCGGCGAGGAGAGGAGCTCGGTGGAGGCCAGCGAGGAGGCTGACGGAGGACGTAGTCGAGCTCGGCCGCTG
CTCCACTGGAGGGCGCGCAGGCAGGCGAGCTCGGCCACCACCGCCGCTACTCCACGGGAGGGCGCACAGACAGGCGAGTCTCGCCGTCGCCGCCGCTCCACTG
GAGGGAGGAGGCTCAAGGCACAGTGGGCTAGGAGAGGAGAGAGGGGAGGCTAGCTGCCGGCGGGTGGGAGGCCGCCCCCGGGAGGGAAGGTTGAGCCTC
CCGGGGGCAGTGCTTCTGCTGGAGGCGGCTATGGGTTGGGAGCAGCCGACGACTGCTGGGGGTTGGGAGGAAACCCTAAACCTAACCTAGTCTGATACCATGTA
GGAAATTGTAGTACTTGGTCTATTGATAGGAGATGGGGTACAATATATAGACTAACAGGACCCTAACCCTAATGGGCCGGCAACCCAACAGTGGTACCGGCCCACT
CACACACACAAAGTCTAACAGAAACCTATTAGACTGAAATTGAGACTATACATTCAACAGCAGTAGCAGCAGCAGCAACAACAAAACCTTTTAATCACAAGCAAGT
TGGGGTAAGCTAGAGTTGAAACCCAACAAAAACCACAAATCAAGGCGAGACTAGATTGTATATGACAAATATTTAGGCTGCAATAAGTTGCTTGAAAGCTAGATTT
GATGAAGACAAAAACCAACTTAATAATAAACACATAACATGATATGAGCAACAAAGAACTATTCAAGTTTCTTTTCATGCCCTAAACTGGAAACTGCCCCTTCTCTGT
TGTTTTTTCCCTGCTGCACGACCTGTCTGTCCTGAGCCTTTTAGGGGCTAGGCTGTGACGGGGTTAATTTGAGAGCTCACCACTTATACAGTTGTCTGTTCATGATTT
ACCACCCTGGATCACTTTTATTGCACAGATGATCCCAAATGTCAGTGGGATGGGCCGATGGGGTGCCAAATCCTGAACCAGCCAATCTTAAGAGTCTTCATGGAAC
AAAAAGTGTTGGCTCAATGAGGTTGCATTGTCCCTCTCTGTGGCATATTTGCCAATTCCATGGAAGGGAAAGGATGGCAAGGTTAATATTTTGAGAAATACGATCTC
TAGGTTTTCAAAGCTCATATATAGGACGCAAAGTGTAAAATGCATAAACACCAAGACAGGAGTGTTGTTCTGAAAAGAATATTTTTAAAAAGAGTAAAATGCATTAG
CGGTCCTTGAACTTGGCATGTTGTGTCAAAGTCCCTGAACTCTCAAAACTAGGAAAACAGGTCTTCGAACTTGACCGGCCATTCAAAACTGGATTTGCTCAAACCG
GATGCCGATGTGGCAATGCCACGTTGAAGTCACTTGAGTTCTGGAACTTGATATGTTGTATCACTTGAGTCACCGAACTTGGCATTATGTGTCACCTGGGTCTCCGA
AGTTTCATTAGTACGCTTCAGCGTAGCACGCCACATCGACATCTGGTTTGAGCAAACCCGATTTTAAATGGCCGACCAAATTCGAGGACCCATTTTACTGGTTTTGG
ACTTAAGTGACACAACATGCCAAGTTAGAGAAGCGCTAATGCATTTTACTCTTTTAAAAAGGTCCTCTCAGCTCTATACTTATATTTTGGTTGCATGGTTGCACCAAC
ATGTATGGTTGCACCAGATATGTTCCCATGAGTGCTTAACATTTTACCTCTTTATAAATTGACTTGTAGTTTGAGCTGGGAAGAGATGGATGAGTGGGGGGAAAATC
CATCCTTCAACCTCCACTCAAGTGAAGCAAATGTTGGAATTTGGAGCAGGCATTCTAGCTGTCACTAATCCACTATGCTAATGTATTATTTTTCTGGAAAGTTGTGTTT
CGCCGTATTTTTCTAGGAGAGCAATTATTTGGTCAATCTAGAACTCGTAAGGCCTTAGGCCGCAACATTGAGAGCCTAAAAGGTTAAAACACCACTTTGATACTGAT

ACGTCATTATGGTTTGGGGGCATTAACTAGTTCATACCTAGGCCTTCCTTAAACCATCACATTTCAGGTGCACCACATTATTTGTTCTAAACCAACGCTGTAGTCAGTA
ATCGCAATCATATAGCTGTGAAACTATTGTTTGACTTTTGTGGGCTGAACCACCATGTGTGGGCTTCTTTTTGGATCTGGTTTAGTGGTAGCCAATTTAAAACAAAAT
TTCACTTTTGTTTGTGCAAACTTGTGATAGTAATGGAAGGAGAAGCAGGTGAAGTACAAAGTTGACATGGGAAGGGGCAGTAAAAGGAGACTTGAAAGGATGA
AATAATTAAAGATTTAGCCTTGAATAGGAGCGAATGTAAAACAACTATTCACGTGCTTGAATCTTGATTTGTGGCTTTTGCTGGGTTTCAACTCTAGCCTACCCCAAC
TGCTTGAGACTAATTTTTTTTGTTCTGTTTGTGCAAACTCGCAATAAGCAACAGACACACAGAGGTCTGGTAGTTCATTTCTTTTGATTTTCCTCATTTATATTTCCGG
TAATATCAATTTAATCCACCCAAATGCCATAAGATTCCTCTAGCTGTAATCTCAAAAGCCCTATGTTGCATTCCTGCTTGGTCAATGATGATGGGCATCATGGTTGACAC
TCCAAGTTTGAACTGAACCTCGCCATGGCAATCTGAGGCCAGGAGATAATATTTCTGACATATAAATTAACTTCAAAGATTTCACATAAAACCATAAATAATATTTACT
TAATTAACGATGAAGGAAAACTGTTTGATTCTTTTTCAATTAAAGAATCTCCATGCCTTTTTCAGTTAAATTTGATTTTGATTTTTTCTGTTGTCTGTCAGGATCATTTA
AGAGAAACAGGAGTGTTTCTTTTCTCGAACACTAGGAGAGTTGTGTATCTTTATGTAAGAAAAAAAGTCTTACAAAAAGTTTCTAAGGGTACTTGCTTGCCCTTGC
TATAAATATAGCGACCTTGACCTAGACCTCACATAGGCAGCAGGGCTAGCCCAGTAAGAAATACCCGTGGTCACTGGTCAGTGTTGCCTGCTAGGGTTTCTGTCTTG
CCTGTCTAGTTAAATTGCCTTTTGCCCAGTTGAGCTGGCTACATGGTTTTCAGGTTAATTTCTATGGGTTTTGGTAGTAAATAATAAATAAAATTTCCATACGAAGCTA
TTTTTCCTTAGTCTGTTCTTATCCTTCTCTAGAGAAGCATGTATGCCACTCAACCAGCACTATCACCTCTTTCTCTAGTCTCATAGAAGGAATGCCATTAACTTATAAGA
CAAGCTGAATCAATGATATCTACTTATTAGGGTTATTAAAACGCTGTTACAAACTGTTTTTTGCCAGAATTTTCTATATGATGTTGCAGAAATATGCATGCTTTACTGAA
CAAGGTCTGTCTAACCACCTTTGATACCTTGTATCTGGAACATTTCTGAGAAGCTTATGGGCAAAGTCTGAAGATTCTTGTTTTTTTGGTAGTTGACCCTGTTAGAGT
GGGCGCACTAACCAGTTCAATCCAAAAGCTTAAGCTGATGGGAGAAAGTAGACAATCCATTTATACACTTCAACACCCCCACTCACGTGCAGCCAGATAGAAAGG
CGCAAATGCAGCCAAAGGGAAAGTCGCAAACGTGGAAATAAATAGGTGGAGGCACAAATAAAGCCTCTGCCAGGATTCGAACTCGAGAATTCTGGCTTTGATAC
CATGTTAGAGTGGGTGCACTAACCGGTTCAACCTAAAAGCTTAATGTGATGGGAGAAGGTAGACAATTCACTTATACACTTCAACATGTCCATTCATTATCAAGAGT
GAATAAGTCTTATAGTGTTCTACCAATGCAGTCTGGCTGATCAACTTCTGAAGCAGATAACAAGAAGCAGTTGGGGTGGGGTCCGAGGACCTATGCAAGTTTAGAA
TCATTTTGTTTTTGGTGGGTGGGGGCAGGGTGTAGTTGATACAAACTAGTGGGGTAAAGGGGTTTACTTGGCTGTGACTTATTATATGCTTGTACTATATATTGTCTA
ATTGCTTTCACAAGTGTCTCTTTTGATTTAGTCCAAATTTGCTAGCAGTGCCAGGTTTGCACCAAGTGCTTTTATCTTTATAATGTAGGCTAGAGGTTTTGAGTAGTTA
TTATTGCTATATTTTCATGGAAGGTTGTTCATAAAGAAAGGAAATGATGCTTAAAAACCGGCTATGATTTGCTTCGCCCCTTCATGGCAAATATTAAATATATAAACAG
TAAAAGATAGAACTTCAGTACATTGTCTGTATTTTATTCCATTCATTCACATGTTCTTCACCACCCAGTTTCATCCTGCAAGTGCTGCAATGTAGTTCTGTAGGAGGTG
AAGGTTTCAGACATTTTTGTACCCTACGTCCCTACCTCTGTCCTTGTCATTGAGCCACTCGCCAACATACAACCTCCGTTCTGGCAGGTCTGCAGCTAGCCCCCCCC
CTCTCTAGGTATGAGGAAATTGTTAGAATACCCGTTAGGGTTTGGAACTCTCCCTGTGTAATTACCCTCTCGCCCCCTCTGTAATGGGCCTGGCCTAACTTCAAACCT
ATTAATATATCACTCCACCCTATTCTAGGGTTAGGTTTCCAATATGGTACCACAACCAGTTTAGTTTATTTTTTTAGCCGCCACCTTCCCTTCCGCGCCCGTGCCGGCAT
CGGTCGCCACCGCCACCCCATGGCGGACCTCCCTGGAGCGCGCCCTTCACCGTCGCCGGCCACAACTTTGGCGCGGACGAGCCCGACCTTGGACTTCACGGCGG

CCATCTCCTCTCCACAGGCGCCCCCTCTCCTACGCCGCCGTGGCCGCCCACAGCATCGCCGGCAGCGCCTTCCCCACACCGGCGGCCCCCTCCCTAGCGGCACCCT
CCCCAACACCGCCACCATGGCCCACGCGGCTGCCACGCCCGAACACAGTGCTGCCGCAGCCGACTCCGTTGCTGGTGCTTGCGCTGCCGGACTCAGTCTCCCGG
GCTATCCGACGTGCACAGGTGCCCTCGCACTCGCTCCGTCGCCGAACTGTGTCTCCCGGCAGCCGCCCCTGGCTTCCAAACCACTCGCCCACCGATCGTCGACATC
ACTTGGGCTCCCACGGCGCCCGCTGCCGACTCCACGCTGGTCGCTGCCATCGCCACCACCCAGCCCGTTGTAGCCGCCTCTCAAGAACGTCAATGCACCGCTTCCC
TCGCTTGCGAGTAGGAACGTGCTATGGGTTAGGCCTTGACCACTCAGTTGGCCACTGCTCAGCGTCTTCTCCACGTCCACGGCAGTTCGCTCCCCTGTTGTGCCAA
CCTCGGGGGGGGGGGGGTCCCGAGTTCCCCCACACCTCCAGACTCAACGCCGACACCATCGACGCGCTCCACGCCCAAGCCGCTGGGGTGCACAACATACAGTC
CCTCGTGTTCGTCGTGTTGGACCTGACGTCCTCCCACTACCCTCGCTGGGGCGCTCAGGCGGTCCTCACCCTTCGGCGGTTCGCCCTCGCTGACCACGTCCTCAAC
GACCCCGTCGCCCCGCTATCTCCATCCTGGGTCCAGATTGACAACGTGGTCCTATCGTGGCTCCACAACACCATCACCGTCGAGCTGCAGGACATCATCCGTGACCA
GTCGGACACCACTCGTCGGGCTTGGCTCGCTCTCGAGGGCCAGTTCCTCGGGAGGCTCGGGCGCTCCACCTCGATGCCTAGTTCCACATGTTCTCTCAGGAGGAC
CTCTCCATGGGCGAATACTGTCGCTAGATGAAGGGCATGGCGGATTCCCTCCGTGACCTCGGCGAGCCTGTCGCCGACCGCACACTGGTGTTGAACCTTCTACATG
GCCTCAGCCCCCGGTACGACCACCTGAAGGCTCTCATCAAGCGGACCTTGCCCTTTCCCACCTTCCATTCCGTGCGGAACGAGCTTCTCCTCGAGGAGCTCACCAA
GACGCTTGAGGCACCCGCTTCGGCCCGGACACTCTACAACGCTACTCCTGGTGGTCAGGTGTCTTCCGGGGGACAGGCCTCTCGCACCTCGTCGACTGGGGCCC
CCACTCGTCCACCTGCCATAGCCCCTACGACCCCTCATCCGACCTCCACCACCGACGGCAGCCATCGCCCCTGCAAAGGTGGACGTGGGGGTGGCAGCTCCACCC
GTGGAGGTTCCACCGGCCGGGGTGGCGGCCAGGGCTAACCATCGTTCTACAACCCCTAGACCGACACCCTCTCCATGTGGCCGGGTCAGGCCCCAGTGCCTCCC
GTTCTCTGGCTCCAGCGCCGGCTCTTCTGACTGCGCCCGCCCTACGACACACCTTCACCGCCTGCCTATAGCCTGCCTCCTTACGGCGTGGCCCCGATGACCCGACC
CCGCCTCAGCTCATGCCTCCGGGGACCCACACCATGACTCCCTGGTCGTCGCTCGCTGGAGGCTGGGACCAAGCCTCTCTTGCCGTCGCCTACAGCACCTTGCGTT
GGCCCCAACCAGCCCTGACTAGGTCATCGACTCCGGTGCTTCCTACCACACCACCCCCACCGCAGGCATGCTATCTCGCTCTCACCCACCCCATTCCTCCCACCCCG
CCTCGATCGTCGTTGGAAACAGTTCCACTCTACCGGTCACCTCAGTAGGTGCCTCGGTTCTCCTCGGACCGTTCTATCTCAATGACGTTCTTGTAGCCCCCCACATCA
CCCACAATCTTCTTTCTGTTCGTCGGTTCACCACCGACAATTCTTGTTCTATTGATTTTGACCCCTCTGTTTTTTCTAAGGATCTGGCCACTAGGACCCCTCTTGATTG
CTCTGACAACACCGGGCCCCTATACACGCTCCGACCTTCCTCCACCGGCGCGTCTCCACTACCCGTCATGGTCTCCACCACCACCTCCACCACTTGGCATCGTCGTCT
CGGCCACCCAGGACCTGACGTCATGACCAAACTTTTCAGTTGTCTTGATTCATCTTGTGATAGGGGACATTTTGAGGGTCTCTGTCATGCTTGTCAGTTAGGCCGAC
ATACTCGTCTCCCATTTACTACCTCTTCTCGAGCTGAGCAGGCTTTTGACCTGGTTCATTGTGATCTTTGGACCTCCCCTGTACTCAGTCTTTCTGGATATAAATACTAT
TTGGTGATTTTGGATGATTTTTCCAATTTTCTATGAACTTTTCCTCTTCAATTGAAGTTCGACACATTCACCACCCTCACACATTTCTTCGCCTGGGTCTCCACCCAGT
TCCGTCGCCCGGTCCGTGCCCTACATTGCGATAATGGCCGCGAGTTCGACAACCACGCCTCCCGCTCGTTCTTTCTCACCAGTGGCGTTCAGTTGTGTCTCTTGTGC
CCCTACACCTCTGCCCAGAACGACTGGGCCGAGCACATGATTCGCACCACCACCAATATGATTTGTTGCCTTCTCTTCCAGGCATCTCTCCCTGCCAGCTACTGACCA
GAGGCCCTACACACCGCCACCCACCTCCTCAACCGCCTCCCATCGAAGGCGCTGAACCACCCCACCCCCACTTCGCCCTGTACGGCACAACCCCCTCCTACGACCA

CCACCGCGTGTTCAGCTGTGCCTGCTATTCCAACACTTCCGCTACTGCTCCTCATAAGCTGTCTCCCCGCTCCGCTCGATGCCTCTTCCTTGGGTACTCCCCTGACCA
CAAGGGGTATCGTTGTCTTGACCTCAGCTCCCATCGCATCATCATCTCCCGTCACGTCGTCTTCGACGAAGATGTGTTTCCCCTTGCTGGCTCCTCCCCAGCCACTGA
TCTCGACTCCCTCCTTGAGTCTGATCAGAGTACCCATCCCCCCAGGCACTTCGCCTCGCACCGTTGCCCGCGCCTCGCGCGGCCCCGTCGACCTCGCTCGCGCCAC
ACGCGGCTTCGATACCCCGGCTCGCACTTCTCCCCGCGCCACGTGCGGCCCCGTTGCCGGTGCCACGCGCGACCTCGTCGACCCCGCCCGCGCCACGCGTGGCTC
CGTCGACCCCGGCCCCATCGACGAGCGGGACTCGCTTCGCTGACCCCGCCCTCATCTACCACTGCCACGGGAGCACTCCTCCCTCGGCGCCCATGGATCCGAGCC
CCTCGACGAGCGCGACCCGATTCACCGACCCCGCCGTCGTCTACCATCACCGCGAGCCAGCCATGCCCGCGGCCCCGGCTCCTCGCTCTGAGCCGTCGGTGTACC
ACTCGGTCGCCATTCACCGCGACCCTAGGCACATCCACCTGATGGTGACTCGACGCGTCGCAGGTGTCCTTCGGCCCGTTGACAGGCTGATTCTGGCCGTTGATAC
GACCACTACTCCTCCGGACGCCTCCTCGGTCCCCTCCTCCGTTCGCACCACCCTCGCCGACCCACACTGGCATCGGGCCATGGAGGAGTATGCGGCCTTGCTGGCC
AACCACACCTAGGACCTAGTGCCACGTCCCCCGGGCACCAATATGGTCACCGGCAAGTGGCTCTTTTGCCACAAACTGACTTCGGATGGCTCCCTCGACCGCTACA
AGGCCCGTTGGGTCCTTCGGGGCTTCACCGAGCGCCCGGGAGTAGACTACGACGAGACCTTCAGCCCCATCGTCAAGTTCACCACCGTCGCATCGTCCTCTCCTT
CACCCGGGACTGGGCGATCCATCAGCTCAACGTCAATAATGCTTTCCTCCATGGCACTCTGACGGAGACTGTCTACTGCAGCCAGCCCACCGGATTCGTCGACACC
GCTCAACCAGATGTGGTCTGTCGGCTGAATCGCTCCCTCTACAGCCTCAAACAGGCGCCGCGAGCTTGGTACAGCCGCTTCGCCTCCTACTTGGCCTCCACCGGCT
TCGTCGAGACTAACTCGGATACGTCACTCTTCATCTACCGGCGTGGCGAGGACGCCATCTACCTCCTCCTCTACGTCGACGACATTGTGCTCACGACATCCACCGCC
GATCTCCTACAGCAGACGATCATCGCCCTCCAGCGGGAGTTTGCGATGGACCTAGGGCCCCTCCATCACTTCCTCGGCATTACCGCCAAACGTTGGCCCTAGGGTC
TCTTCCTGCACCAGCGCTAGTATGCCATCGACATCCTAGAGCGGGCTGACATGTCCGACTGCAAGCCCTGCTCCACGCCTGTCGACACTCGTGTGAAGCTCTCTGA
GGACGACGGGCCCTCGGTCGCCAACGCGACGTCTTACGCTTGACTGGCGCCCCCCAGTACCTCACCTTCTCCCGGCCCGACATCGCCTACGCCGTTCAACATGTGT
GCCTACATATGCACACCCCACGGGAGCCCCATCTCACCGCTCTCAAGCGGATACTGCGCTACCTCCGCTGCTCCCTCGATTACGGCCTGCTCCTCCGACAATCCCCA
ACATTGGAACTCGTGGTCTACACCGACGCTGATTGGGTCGGCTGCCCCGACACGTGTCGGTCCACTTCCGGCTATGCCATTTTCCTGGGCGCCAACCTCGTCTCTT
GGGCCGCCAAGTGGTAGCCTGTCGTCTCCCGCTCCAGCGCTGAGGCCGAGTACTGCGCGGTGGCCAACGGCGTGGCGGAGGCCTCCTGGATGCACCAGCTCCTC
CACGAGCTCCACAGTCCCCTTTAGCACGCCACCCTCGTCTACTGCTACAACGTCAGCGCCGTCTACCTCTCCACCAATCCCGTGCAACATCAGCGCACGAAGCATGT
GGAGATCGACCTGCACTTCGTTCGCGAGCGTGTCGCTGCAGGTGACGTTCGGGTTCTCAGCGTCCCCACCATGTTGCAGTTCGCCGACATCTTCTCCAAGGGGTT
ACCGACGAGTGTATTCCTAGACTTTCGGTCCAGTCTCAACATCTGTACAAGATAGAGTTATGACTGGGGGGTTAGAATACCCGTTAGGGTTTGGAACTCTCTCTGTG
TAATTACCCTCTCGCCCCCTCTATAATGGGCCTGACCCAACTCCAAGCCTATTAATATATCACCCCAACCTATTCTAGGGTTAGGGTTTCCAATATAAATGAAGCTCTGG
AGTCATGTATTATTAGTAAGGATTAGCCAGGTGCTACCTAGCACCCTTTTTTTCTTTGCAGTGGCGGATCCAGGGTCCAATCCCTGTGGAGCAAAGCTTTACACAGA
TATACACAAGATAGATTAATTGCATGTTAATTGACAAGTGTTATGTAGGGGTTTAACATGTTGTGGCAAAACCTGTGGCCATGTGGCCCCACAACTTAAGTGTAGATA
TGCCACTGTTTCTGCGAATTGTCCTCAGACAAGCTTATAGCCAGCAAATCCCTTTCCATCCAGCTGCCATTTGCTAGTGTAAGAGAATGAAGCGCTTGTTTTATTGCA

TAGAGGAGGGTTACAATATATAGAGACACAGAAACCCTAACTCTAATGGGCGGACGACCCAATAGTAGTGCCGACCCACCTACACATAGGACACACTGTATAACATC
CCCCCGCAGTCGCAACAGGGGCACCGCACACGATGAGATTGGAGTAGAAGCCGAAGGTAGGAGCCGACGGGTTAAAATCCCCCCACAGTCACAACGTCTGGAG
GGTGCGAATGTTGTGGTTGGAGGTAGAGACCGATGTGCACTCCAAGAAGATGATAGCCCCTGGATGCCGAGGTAGCTGAAGTCGAGGTGGCGGTGGTCGGGAG
ATGCGCAACGGTAGCCTGTTCTTTGGGAGGGTCGACGTTCGAGCGTCAACGATTGACAGGGCGACAGAAAAGGGACAACAACAACAGAACGTGAACCTTCTTG
CTTCTTTCTGCGTCCGGTGGTCGAGGAGCCTCGCCAGGGAGGCCGACGGCAGCGCACGCGTCTGTGCCGGTCAGGGTGGCTGCGCCCGCGGCAGAATATAAGG
GGTATCGACGGATCTGGCCGGTAAGGCCATGACAGCGATGGATCCAGCTAGGAAGACGACAATATGACTAAAGGGATGGCAAATCCAGCCGGGAAGGTCGCGAC
AACGATGGATCCGGCCGGGAAAGCCGCGGCAGCGACGGATCCAGGTGTGGCGGCAATGAAGAAGGGGTAGGCGGCGAGTCAGGTATAGCCGGGCAGGGGTC
TGTGTCGAGCCTAGCAGGGGGTTGTGACACCAGCGATGGAAGAAGCGCGACGAAGGTAGGAGGCACTGTTGGGTGGAGCAGGCGAGGGCAAACAACAACCT
ACTCGCCGGAACAAGAAACATGGCGACGCCTACAACCGTGGGGGAGGAAGGAGAGGGAGTGTGCAGGCCGACGGGGGAGGTCTAGGTCACGATGGCCTGCC
CTGGAAGCGGCGTGCTGGACATGCCAGCGGCAGCGTGGATTCGGGCGGACGACTTAGCCCCTGACCTCCTTCTTTGTTCACGCCGGTGCAGNNNNNNNNNNN
NNNNNNNNNNNNNNNNNNNNNNNNNNNNNNNNNNNNNNNNNNNNNNNNNNNNNNNNNNNNNNNNNNNNNNNNNNNNNNNNNNNNNNNNNNNNNNNNNNNNNC
CACCTACACATAGGTGATAGGACACACACTGTCTAACATCTAGTCTGTAGATCAGGAAAAATACAGTGCACTTTCACATCTTAAGTTCTTTTAAAGTTCTCTAATATCC
TTCTGTTGGTAATTCTTTCTCCACATGATTGATATCTGCAGTTTTGCATTTTTCTCATTCCATAACTTGAACTTTCGAGCATAATTTTGCAATTTGGTATAACCTATGATAT
GAAAGTTTCAGATAATGGACAGGGGAAATGTCAAACTTGATAAAAAAACCTTGGTTTAGCTGTGAATTCATGTGATCATGTCCTTGCTAAATGGCATTAAACTATTC
CACTTATGTTGCATGATTTGGACATTGTTTTTCTATAAGCAGCACTATGTTCAGTCATAATTGGTGCAAACTAGAAAGAAATGTGCTATCCTACTGGTTTCTGATTGGT
TGGAATCTATAAGCTGACTTATTTTGTTATCTTGTAAGGTTAAATATATTTGGAGACTAACAAAATCCAGTCACTGACAACAGTTTGAAGTCCTTAATTATTCCCTTCAA
TCATGTAGTTTTATATTCAATGCCTGTACCTAACAAAATAGGAAATTGCAGTTGGAAAAGGAAGCTATTAACAATGGTACTTCTCCGGGGCAAGCCCATGACATTGA
CATACCGCCACCACGGCCTAAAAGAAAGGCTAACAGTCCATATCCTCGAAAAAGTGGTCTCAGCTCTGAGACACCAACCAAAGAACTTCCAAGTGACAAGTCAAC
AAAACCAAATATGCCCTTGAGCAATGAGAATGTACTAATGGCAGGTGATGCATCTCTTCAGGTAAATTTTCCCTGGTATTTTGCTTTCCTAGGAATACATGTTATCCTC
TGTATAAGTATCTTTAATTTCCTTTAATTGTAAACTCATTTGCATCCAGAAATTTCAAAGGAAGAAGTTGTCTGGAAAAGAAAGTTGCTCGGAAGTTCTTAATCTCTT
CCGTGATGCCCCATCTCCATCATTTTCTTCAGTTAACAAAAGCTCTTCAAATCATGGTGCACCGATTGAGGCAAGTAAAACAAAAATCCGAGATATGACCATTATGGA
AAATAGTTCTCTTAACCCCAACATGCAAGAGGATGTAAAGGAAATCAATGATCAGGAGATGGAAAGGCTTAATGGTACCCAAATCAGCTCTAAATGTGAACACTCT
CATGAGGGATATTTGGACATCTCAATGCAACAAATGAAGCTAAAGCCAGAGTCTGTGGAGACAACAGATGTGGACAAACAAACCGCAAGAGCTTCACACTCCCTA
GCGGAGATAACTGGGACAACTAGCATTCCGGTCACTGCAACTGAAGAAACTCATTCTGTTCTAACAAGTGATCAAGTGGGAATCAATGGAAGCATGAACCCATCC
ATCCATCCAATGTTTCCTGCAGATCCAAAATTTGATAGCAGTGCCACACCACAGCCTTTTCCTCATAATTATGCTGCCTTTGCTCCAATGATGCAGTGCAACTGCAACC
AAGATACCCACAGATCATTGGTCAACATGTCATCCACCTTCTCCAGCATGCTTGTTTCCACGTTGTTGTCAAACCCTGCCATCCATGCAGCTGCCAGGCTCGCAGCAT

CATACTGGCCAGCAGCTGAAGGTAACACTCCGATTGATCCGAATCAAGAAAATCTTGCAGATGGTGTTCAAGGAAGGAGCATAGGGTCTCCTCCAAGCATGGCTT
CTATTGTAGCAGCTACAGTTGCTGCAGCATCTGCATGGTGGGCAACACAAGGTCTTCTCCCTTTCTTCGCCCCACCCATGGCTTTTTCATTTGTGCCAGCTCCCAGTG
CCGCCTTCCCCACAGTTGATGTTCCACGACCTTCAGAGAAAGATAGAGATTGCCCAGCTGAAAATGCACAGAAGGAATGCCAAGAAGCTCGAAAACAGGGACAG
CTTGAAGGTTTCAGAGTTGCCGCTTCTTCAGAGTATGATGGGAGTGGAAAAGGCGAGGTGTCTGTCCACACAGAGTTAAAGATATCTCCTGTCCAGAATGCTGAT
GCCACGTCTGCCGCAGGAGCTGACACGACTGATGCATTCATGAATAAGAAAAAGCAGGACCGCTCTTCATGCGGTTCTAACACACCTTCAAGTAGTGATGTAGATG
TGGACAATGTCCCTGAGAAGGAGGGCAATGCTAATGAGAAGGCGAAGCAAGCCTCCTGCAGCAACTCTTCAGCTGGTGACACTAACCACCGCAGATTTAGAAGC
AGTGGAAGCACAAGTGATTCATGGAAGGAAGTTTCCGAAGAGGTTGTAATCTACCAACACTATGCCAATTCACTCTGCCCAAACCTTTGAGCATGCCGCACTGCCT
TTCTAATTCACTTTTTTTGTTGGCAGGGTCGTCTGGCTTTCCATGCGCTGTTCAGTAGAGAAAAGCTTCCGCAAAGCTTTTCTCCCCCACAAGCAGAAGGCTCGAA
GGAAGTTGGCAAGAAGGAGGAAGATGAAGTCACCACAGTGGCAGTTGACCTCAACAAGAGTACCACAAGCATTGATCATGACCTCGACACAATTGGTGAGCCA
AGGGCTTCCTTTCCCAATGAACTGTCGCCCCTGAAGCTGAAATCGCGCCAAACAGGCTTCAAACCATACAAGAGATGTTCTGTGGAAGCGAAGGAGAATAGGGT
GCCGGCTAGCGACGAGGTTGGTACCAAGAGGATTCGTCTTGATAGTGAAGCATCAACATGAGCGATTTCTACATGGTTACCGCTCGGACTTCCTCTGCCGCCATGA
AGTTTATGACAAAGTTATATTTACATCTAAACTAGTATAGGACTTAAAGACCTTGCAATGCAATGCTTCATGAAACTGTTGTGTGCTGTATTTTAGTACTTGTGAAGCA
GCCCGGTAGACTGCTTTTAAACCTTTATCTCGTAGACTCTGATTTAAGCACCAAGCAACTATTGAACTTTATATTTTTGGCGGGAAGGAAAATAATTATTTTTTGATTT
ATTTTGCAGGGTCATGCATTTACATAGCACTCTTGATGCAAAAGTTTATTTCGTAAAGTTGTAGTGGAATATGCTGAGTGAGAAGGTGAACTATTTATGACTAATATC
GTCCCTGTCCTAGCTAATCGCCCTACTCGACTAATAATCCTCGCCCCTTCTCCTCTATCTCGCTCCACTAATAATTTGAAAGTATTGTTCCCTGTAATTCTTGTTTGTTTC
TCATGTATGTGCTTCCATTCATTTTTAGTATGTCCTTAGTCTTTGACAGTGCCATAAATAACTTTAATTGTTTCTGCTGTGGGGTATTCATATAATACACACACATGAGAA
CCTGCGACCATTCAGTGAAGTGCTCCCTATCTACCAACTATTCTTTAATTCAATAATCATTTCTTTTAGTCTTGCATATAGATCATTCTTTCATCCAGTGTTTCACTATTTT
AGTTTTTTTATATTATGAATTTGTAGAGTTTTCTTGGTTGGACCTGCAACCTTTCAGTTTCAGTCCTCTGTTAAGAAACTAGATGGGTCGGCAATTAAACAACAACAT
GTTTTATCGTCTGTTCTTGCGACCTTGAGATTTTTTTTAAAAAAAAATCAACAATTCATTGCAGGCAGCTGTAAATGAAATGTTTCTGTTGTCTGCTGAAACTGGATT
CACCAGTTAATTTCCAAGGTAGTTCTCTCTGTGTATATATGTTGGAGAATTCAGACAAGTACTACCACTTTGTTTCAGGGAATGGTCTTTTACCATGTTGCATGCTTCA
GCTTTAGATGAATTGCTGTGGTTACAGTTTTGTCCATGCTGCATAATGTTTGCCTATAGATATGCATTGAAACTTTAAATAAACCTATACAAAAATTCCACTTGGTATTT
GAATTTGCCAATCAATTAATGTAAACAGATAGCTATATAAGCTGCTGCTACTTTTATTTGTCATACTGTCAAGGGCGTCGAGCCCGAGGGTAGGAGGACCACGGCTC
CGAAGATCGTAGCCGCCGTCCGTGTTGCCGACCGGGTTATGCCCCCAGGAACGGCGTTCTTCGTCAATGGGTTATGCCCCCTGTTGCCGGTTTGAGAGAGAGAGA
CAAGGATGATGGGATAAATCTTTCTTCCCAAATCACGAGTGTTTACAGGGAATATAAGGCCATAGGCCAGGACTAATAAAGGAAAGCCACCAATTAACTTTTATTCA
TTCTAAATCTAGCCGCTAGGCCCGACTGCCTGCTGGATCAGGCGCTCTACGCGCTCAGTCGCGCGCCGTACGTCGCGTGCCCTGTGACGCCGGGTGTACGTGTTTC
CGTACATCACATCTCTCCCCTCCTCGAGAGGCAGCTCGTCCTCGAGCTGAAAGAGTGGGTAGGACGCGCGGAACGTGTCCAAGTCCTCCCAGGTGGCCGATGCCG

GAGAGGCTCCCTGCCAGCGAATGAGGACCTGCCGGACTCCCCTGAGCAGCCGCATCTTGACTGCTTGCTCTGGCTCTGGAATAATGGCGCCGTGGCGAACCAGA
GGTAGAGCTGGAGGCTCTTGCGGAGGCGCGCCATGGAACTTCTTGAGAAGCCCCACGCGGAAGACGTCGTGGAGGCGAGCTTGAGGAGGCAGGGCCAGGCG
GACAGCCACGGTGTTGATGCGCTCGACGACGCGGTAAGGGCCGTAGAACCGCGGCTTTAGCTTGCCAGCCGACGAGTCGGGCATGGAGGCAGTCGGGCGCTG
GTGTAGACGAAGCAGCACCCACTCGCCCACCTGTACGTCGATGTCGCGGTGTAGACGATCATAGTGACGCTTCTGGACAGCCTGGGCCTGCTCCAGCCGGTGGCG
AATATCTGCCAGGAACTCTGCGCGCTCCTCCAAAGTCCTGGCCACCGCAGGCACCCGCGTTGCCCCATGCTCGTAGGTGCGCAGTGAGGGAGGGTCGCGGCCATA
GACGACGCGGAACGGCGTGTCGCGCAGTGACGACTGAAAGGCGGTGTTGAAGATGTATTCCGCCCATGGGAGCCAGCGCAGCCAGTCGCGGGGTCGGTCTCCT
GTCAAGCACCGGAGATACATGATGATGACCTTGTTCGCCGACTCGGACTGGCCGTCTGACCGGGGATGAAATGCCGTGGTCATCTGCAGCTTGGTGCCGCTCAAG
CGCATCAGCTCGCTCCAGAAGTTGGAGGTGAACACCGAGTCCCGGTCAGAGACGATCGACTGGGGAATGCCGTGTAGTCGGACGATGTCGGTGAAGAATGCCTG
CGCCACAGACTCGGCCGTGTACGGGTGCGCCAGGGGAATGAAATGTGCATATTTGCTGAAGCGGTCCACCACCGTGAGGATGACGGTCTTGCCACGGACGCGAG
GCAGCGCTTCGACGAAATCCATGGCGATGTCCGTCCATACACCTTGTGGCACCGGCAGGGGCAACAACAAGCCGGCCGGGTGCTGATGGTCGGGCTTGTTGCGT
TGGCAGACTGCATAGCCACGAATCAGATCCTGCACCGCACGTTTCATGTTAGGAAAATGGAAGTCGCGCCGGAGTCGGTGCAGGGTGCGCTGCACGCCCTCATG
GCCTTCCTCGTGGACAGCCACCAAGACCTCCTGGAGCAGCGGGGAGGCTGGCGGAATGTACAGCCGGCCAGCATAGTGGACGAGGCCGTCGGTTAGCGCCCAT
GGCCGCGACCTGGCGCCGCTACTGATGTTGTGCTGCAGGGCAGCGAGTGTCGGGTCGATGGTGTGCGCCTGACGAAGCCGATCGATGAAGTCGAAGCGTGGTC
CGGACAAGGCCAGCACCAAGCCCTCCTCCGGTGTGTCGCGGCGTGAGAGCGCGTCCGCGACGGCATTGGTGTGCCCCGACTTGTACTCGACGGAGAAGTCGAA
GCCGAGAATTTTGCCCACCCAGTTGTTGGGGACTTGTTCTCAAACGCTATGAATCAAGAACAAGGCAACATAAAATGTTAAATGATAATGTCCTTCGTCCAATGAAG
CATTATTCCCTCGGGGATAATGCACTCTGGACGAAGGCGAACGACAATACGATTACGAAGGTTGAGTTTTCGTAATTGTACGTTAATAAATTGTACGAAATAACATAA
GATATAGAGTATCAAAAGTAAATGAATCATATTATATCCATTCAATATATTTAAGTATTGGATACAATTATACCTCTGCCTTGACAAAGGTTGGTTCTCAAATGATGAGAT
GGTAAAAAATCTGAAAAACCGTGAACAGTAGAGGAATACTGTTCACTATTTATAGGCACAGGACACAGCCTGTGAGAATTTACAACCATGCCCTTCATGAAAGTTT
ACAATAACGACTCAAACGCTTATGGACTAAAAGGTCTTTCTACTTTTAAGTCGGTTCATAATTCCGAAGCTTTATGAAGAGCAAGCTTCGGTCATCGCACGCGGACA
GCTTCAGCTGAAGCTGCTTCTTCCTTCAAGACCTTCGGCGCGACGAAGCATAGTCCCAACAGTAGCCCCTTTCGCGGCGCTAGATCGTTTTTCGTAACGAGCTTGA
TCCGTGAAAAGAAAAAGTCTCTTAAGCTTCGGGAAGCCGAAGGTCCAAAAAACACCTTCCCTGAGCTCGTTGTCGAGAAACGATTCAGTTTCCGAGCGCGTAGC
GGTCCCACCTTGCAGAGTTACTGTTTGGTCTCTGCAGTCCACCGCGCAGCGAGTGCAAGCGGCTGTCCGCCTGGTGTAAAAATCCTGGCGCTTCGCCTTCTTACCT
GCAGTACTATATAAACAGACGAGTAGGTGTGAAGTTACCACAGCATTCATTGCTATTTGCACTGTTTTGCTGCCAAAATTTTTAACCATAGCCGAAGCTTGACTCTCG
GAATCGAACGAAGCTCCAGCTTGAAGCCTGCTTCATCAGAAGAAGAAACTTCGGAAGAAAAAGTATTTAAATCCCACAAATTCAGAATTAAATGGCCAGAGTGCG
CTCTACCGCTAGGGTTGAGCGTGAGGGAGGCGGAGCTGAAGGCTCGGAGACTGTTCCCATCTCCGAAGCAATGCAGCGATCCGGACTGGTAACTTCGGAAGAA
ATCCCCACTGTTGAAGCAGAACAAGCAACTGCCGAAGCAGAAGAAGAGAACATTGAAGAAACTGATCCCGAAGATGATTATCGTATTGCCATGCCGAGTAAACCT

AGTCACTTGGACTTCGGAAAGTCGACTATTTCAAAAGCTGATCTCTCCAAAATGGTAAAGTCGGGCTATTTTAGTGAAAATCAGAAGAAGCTACTTCGCTTCGGGG
GGGAAGAAACTACCCCGAAGCCAGAGAAAGATGAAATAGTTTTTTTCAAGAGCTTTCTAAAGGCTGGGTTGAGATTCCCTCTGAATGGGATTATTGCAGAAATATT
GAATAGGTTTGGAATCTACTTTCATCAGCTGACTCCTAACGCTATCGTTAGGCTCAATGTTTATATCTGGGCCCTCCGAAGCCAAACGGTGGAACCGTTTGCGGACA
GCTTTTGCCGAGTTCACGAGCTGCACTATCAGACGAAGGCTAGAAAAGATGGAATGCATGACAATTTTGGTTGCTATAATTTTGCTTATCGGAAAACCACAAAGTT
TCTTGTAATCAGCTACCGAAGCAAATGGGCAGCATGCTGGAAGTCGGAGTGGTTCTATGTCAAGGTTGATGATGACAAGGAGAAGTTTGTGCAAAGTCCACTTAA
ACTAATCTTCGGAGAAACCCGACCTCGTTGCAACATGACACCAGAAGGTCCAACCCAACAAGTGTTAGATGAGTTCAGAATTATTTCAGAGCATATCAGTACAAGA
GACCTGGTACAGGAGTTTTTGGCTTTTAGGGTTTTTCCTAGTTTAAAAGAATAGAAAATGCCGAAGCTAGAGGGGGAGAAAAAAGAAGGAGAACTTGTGCGATT
ACCTTACTATTTCAAGTTCAAGAAATACTTTAAAACACCTTGCCAAGAGTGGCTTGATACAATTGAAGTAATGTGCAATGAAATACTCGGCAATTACTCCAAAAAAG
AAGATCAGTTGATGACTGCGGCCTTCGGCACCCGTCCGAAGCGAAGATTGAACCGAGTGCTGGATGCCTTGGGTTTTGAATACCCTGACTACGAAAATCTGAATA
AAAGTGTCGAAGGCCGGAAAAGAAAAAGGGTAGCCGAAGCTTCAGATAAAGATGAGAAGGAACCAGCAAAAAAGAAGAATATTCCGAAGAAAAAGAAAGTAT
TATCTCCGAAGCAAAAGATATCCGACAAAGAGGAGACCCCCGTATCACCCTCTGCCACTGACGTGGAAGAGATTTTGAAGGTAATGACTGAATCCCTGCCTATGAA
GCTAAGTCCATTGGGGCCTCAACTGACGAAGTTTTTTCAGAAGGAAAAGGAGTCCGAACAAACGAAGAAATCAACTAAAACTAAAAGACAAAGAATCATCGCAG
TGACAGAAGTCATTGACAAGACACCACCGAGAGCTTCGGCTCGGAAGATGCCAGAGGCTGAAGGATTAACAAATATTGAAGTTGCGCCTTCGGAGATGGCGACT
ACCGAAGCTGCTTCAACCGAAGATTTGAACTTGGAAAGCACAATTGAGCATATCGATAAAATGCTGCTAGACATGGCCACAGAAGAAGCTACCACTGCCGCCGAA
GAGGCCATGGCCGCAGCTTCTGGGAAAGAAAAGGAAATTGCTGACGAAACTTCAGAGGACGAAGCCTTCATGTTCCAAAATTTAGTTGGACAAGAATTGTCAGA
GGCCGAAGTGGAAGAGCTTAAAGAATATGCCAAATCTTGCGGTTATAAGCCAGGAGCACTCCTCTTCGGGGGAATTGACGATGAAAAATTGAACTGTATCCGAGA
CCAAACTGGAGCTAAGATCATCGGTACTCTATCAAAGAGTATCGGTTTTCCGAAGCTGGAAACGGACATCAGCCGCTACCGACGACAACATATCGTTGGTAGTTTAT
TTTATTCTAATTTCAAGGTAAACTACTTTTCCCTTGACTTTTATTGTTTTTAATGATGAAGACATGTCTAACGAAGATTGTTTACGTGCAGAGCATGCTGCTGAGCAAG
GCTTTGAAAATGCAGCAAGATTTAGAAGACAAAAAACACGAAGTTATAATTGAAAGCTTAGAGAGCAAAATAAAAGAGCAATCAGCTACTGTTGAAAAGAAAGA
CTTCGAGCTTCGGACAACTGAAGGTTTATTGGCAGAAGCCGAAGCTAAAGTAGCAGAATTGAATTCGAAGCTTCTCTGTCAATCTGAACAATTTGAACAGGAAAA
CAAGAACTTAGTGTGAAACTTGAAGCCGAAGTTCAACAAAATTCAGATTTGAAAAAATTATTGGCAAACCTTCAAGAAAAATGTTTGGAATTTAGCAACAAATGCA
TTCAACGACTGAGAAAAATTTTCCACTCAGTTGGAGCTAGCAGTGAAAAATTCACCCCGTCAGCTGAAGATTTACCAAAGATTTTTGAACATATCGAAGGTGAAAT
TGATGAGCTTGACGAAGTTATAGCTGGGCACGGTGACTTCTGCGCCTGGGTAGCTTCTCGGGGCACTACTGCAGCTTTCCTAAAAGCTGGCTGCGATCATGAAAA
GATTGTCAATAGGCCCAATTTCACTTTATCACCATCAATTCTAGATGACATTCCTGATCTCGCCCGAAGCATCTCTAATAGATTTGTAAAAATGATATGGACAAAAGGC
GGGCGAGAAAAGGCTGGAGACGAAGCTCGGAGTCACCTTGAACCAGTAAGAAATCATGCCTTGTGCTTACCTTTTCCTTCAAGCTTGGTTTTGACCCTCAACAAC
TTAATTTATGTAGGATGACGAAGCTGAGACCGATGCTTAAAAAGCATGACGCCGAAGCTGAGGCCCCATGAAGATCAATAGGAGTAGACTGTAGACAGACTTAAG

AAACTTTTGAGATAACTTTTGTAAATATGACTAAACTTTTTTTAATGAATTCTGTTCATACCTTGTAATATGCTTTTACCTTTGCATTAATGTATTAGATGTGCTTTGATG
TGGACGAAATTATCCTTTTGAGCCGAAGGCGAAAAAAACACCTTCCCTTCTTTTCGTACACATCGAAGCATTAAAAACAACTTTTCCTTTTTTTCCGAAGCTCTTCTT
TTTGTACACAACGAAGTACAAAAGACAGCTTTTCCTTTTTGCCGAAGCAACCACTTATGCTATGATGATGGTTATCCTATATATGCCTAGATGAATGTTTATGAATGCA
AATGTTATGATGTAATATGATGTGCAAATGAATGTCCAAACACATATACGAAGCCATAATCACAACCGTTATTTCCTTAGAAACAATCACATATCAGCGCTGACTTTTC
GCTGTAAGCTCTGCATTCCCTTAGGAACGACTTTGGAGCTTCTTCGCCTTTTACTTTCGGCGGAATCAGCGTTGACTTTTCGCTGTAAGCTCTGCATTCCCTTAGGA
ACGACTTTGGAGCTTCTTCGCCTTTTACTTTCGGCGGAATCAGCGTTGACTTTTCGCTGTAAGCTCTGCATTCCCTTAGAAACGACTTTGGAGCTTCTTCCCTGTTTC
CTTTCGGCACTCGATGGTGCGTTCTCAGCTTTTACATTTACATCTTTGGGGGATTTTTCGCTTATAGAGCTAAAAAAGAAAATTACATGTGATGGCCCCATTAAAAAC
CTTTCTCCCCCTTCAGAAAGGAAAAGGGTGCCATGAAAGAAAAAATAAAAATATAAAAAATTACATCAAATTATACATAATATCGCCGAAGCTCATCCGCATTCCAG
GATCTAGGAATGTCGTTGCCGTCCATATCCTTCAATCTGTATGAACCGGGTCTTGACGAAGATACTACCAAAAAAGGTCCCTCCCATTTCAATTGCAACTTGCCCACT
GTATCTGGGTTAGCCACTCTCCGAAGCACCAAATGTCCTGGCTCAATATTTTTCAGCCGAACCTTTCTATCACGCCATTTGATTGTTTCGGCTTGATATTTATTGATGT
TCTCCACAGCTTGAAGTCTGATCCCTTCTATAGCATCTTTTTCAACAGAATGATCAGCTTCAGGATCTGACTCTGCCGAAGCTACTACTCTTATCGATCCAGTTTTAGC
TTCCTCCGGAGTTATTGCTTCGTCACCAAACAATAATTTGAATGGGGTAAAGCCTGTTGACCTTGATGTTGTCGTATTGTGGCTCCATACCACTTTGATTAATTGATCT
GGCCACTTTCCCCTGGGTTGGTTGAAGATTAACTTCATTATTCCTGTCATTATGATGCCATTGGCTCTTTCAACGAGTCCATTTGACTCCGGATGCCTGGCTGATGCA
AAATGGATCTTCGTGCCGATTTGATCACAGAACTCTCTGAAAGCTTCGGAGTCGAACTGTGTTCCATTATCTACAGTGATGGCCTTTGGTACTCCGAAACGACAAAC
AATATTCTGCCAGAAAAACTTTTGAATGGTGGCCGAAGTTATTGTGGCTAAAGGCTTTGCCTCAATCCATTTAGAAAAATATTCCACAGCCACTACAACATACCTTAA
GTTTCCTTGGGCCGGTGGTAACGGACCTAACAAATCAAGACCCCACCTTTGCAATGGCCAGATGGGTTGTATCAGCTGAGTTAGAGACGAAGGTTGTTTTTGATCT
CTTGCACATTTCTGACAACCTTCGCACTTTTGAACTAATTCCGCTGCATCCGAAGCTGCCTTCGGCCAATAAAACCCTTGACGAAAAACTTTTCCAAGTAACGGCCT
AGATCCAATGTGAGATCCACACAAGCCTGCATGTATTTCTTTCATCAACTCTATGCCTTCAGCTCTGGATAAACACTTGAGTAGTGGAGCACAAACTCCATGCTTGTA
CAACTCCCCTTCTATCATGACATATGGACGAGCTCTTGCCTCTATCCTCCTGTTATAAGTTTCGTCATCTGAAAGGAAATTACCCTGAAGGTAAGAGATGATCTCAGTT
CTCCAGTCTTCACTATAAACAGGAGATATATTGAGGACTGCTCTTTCAAGAAGTTCCACTGAAGGTGCTTTTATTGTTTCGAAGAACACATCCGAAGGTAAGGGCA
GCCCCTGTGCTGCTGACTTAGCTAGCAAATCAGCATACTCATTTTGTCCTCGAGGGATATTTTTGACAGAAAATCCTTCGAAGGAAGCTTCAATTCTTCGGACCGTG
TCTAGATATTTTTCAAGCTTCGGATCTTTAGCCTTGCAACTCTTGTCAACATGACCCGAAACAACCTGGGAATCAGTTTTAAGAATGGCCCTTCTGATTCCCATTGCT
TTTAATTTCCGAAGGCCCAAAAGCAGGGCTTCGTACTCAGTAATATTGTTTGTACAACTAAAATCGAGTCTCGCCGCATAACAAGTTTTGACTTTGGATGGTGAGAC
CAACACAGCGGCTGCTCCCGCTCCAAAGGTTCCCCAAGACCCATCGCAAAACACTGTCCATACTTCGGCATCTTTATTTGTTTCTTCATCCTGAGCCCCTGGCGTCC
AATCGGCAATGAAATCTGCCAACGCTTGAGACCGGATCGAGGATCTATGCACATAATCAATGCAAAATTCATTGAGCTCTGCAGCCCATTTTCCAATCCGTCCAGTA
GCTTCTCTATTTCTCATAATATCCTTCAACGACTGCGAAGAAGGAACAATAATATTGTATGCTTGAAAATAATGCCGAAGCTTCCTGGATGCCATCAAAACAGCATATA

ATACCTTCTCCAATTCTATATAGTTTTTCTTTGATACACTAAGAACTTCAGATACAAAATACACTGGAACCTGCTTCTTGACTTGGCCATCAATCTTCTCCTGGACAAGT
GCTGCACTTACCGCTGAGTGCGAAGCTGCCACATATAACAACAAAGGAGCCCCTGGCGTTGGTGGAGTTAATGTTGTTAAATCTATCAAATATTGCTTCAGTTCCTC
GAAGGCTTTTTGTTGGCTTGGTCCCCATTGAAAGACTTCGGCTGATTTCAGCACTTCGAAGAATGGTAAATTTCTCTCTGCTGATCTGGATATGAATCTATTGAGAG
ATGCCAGCCTCCCTGTCAATCTTTGGGCCCCCTTTTTTTGTAGTTGGTGGCTCCATCCGAAGTATAGCTTCAATTTTACTTGGATTAGCTTCAATTCCCTTTGTTGAAA
CCAAGCATCCAAGAAATTTTCCCTTCTTTACTCCGAAGACACATTTTTCTGGATTCAACTTTAGACCAGCTTGTCTAAAACTGGCGAAGGTCTCCTGCAAATCAGCA
ATATGATTTTCCTGTTTCGTGCTTTTTACAATGATATCATCAACATAAGTTAGCACATTTCTGCCTATCTGAGACTGGAGAACCTTCGCAGTCATTCTGCTGAAACTTCC
TCCAGCGTTCTTGAGACCCTCAGGCATCCGAAGGTAACAATATGTTCCACTAGGGGTTATGAAACTGGTCTTCGGCTCATCCTCCTTCTTCATCCAAATTTGATGATA
GCCTGAATAACAATCGAGAAGACTCATAAGCTCCGACGAAGCTGCTGCATCAACTAAGGAGTCTATCCTTGGCAATGGGAATTCGTCCTTCGGACAAGCCTTGTTG
AGATCCGTAAAATCGATACACATCCGCCATTTGCCATTAGCCTTTTTTACCATAACAGTGTTAGCTAGCCATTCTGGGTACTTTACTTCTCTGATAACTCTTGCACTGA
GGAGCCTTTTGTCTTCATTCCGAGCACCTTCGGCCTTGTCATCAGACATTTTTCGAAGCCTCTGCTTTCTGGGTCTGAAGGATGGGTCAACATTGAGCGAGTGTTC
AATAACATCCCTATTAACTCCGCAAAGATCATTAGCTGACCATGCAAAAACATCTTTGTTGTTGAACAAAAACCTTATCAAGGTTCTTTCCTGTTCTTCGGATAACTGA
GAGCCCAACAGCACCTTCTGTTCTGCTATGTCCTCACATAAGAGCATGGGCTTCGGCTGATCTGCTGAAGCTGCTTTCTCCCTTCTGAATTTGTACTGTTCACAAGCT
TCAGCTCCATCTATATTATGGATTGCTTTTGAGTCAGTCCAGTTTCCCTCGGCCTTTCTGGCAGCTTCTTGACTTCCATGAATAGCAATGGGTCCTTGATCCGAAGGTA
TCTTCATGCAAAGGTAAGCAGGATGAAGGATTGCTTCGAAAGCGTTGAGGGTGCCACGACCAATAATTGCATTGTAAGGATATTCCATGTCAACAATATCAAACAC
AACTTGCTCGGTTCTGGTGTTGTTGATGAATCCGAAGGTCACTGGCATGGTGATCTTGCCCAGTGCTACAATCTGTCTTCCTCCGAAGCCACAGAGAGGGTGTGTA
GCATCATGAATCTTATCTTCTGGCTCTTGCATTTGTCTGAAGGCCTTAGCAAATATGATATCAGCTGCACTGCCTGTATCAACCAAGACATTGTGGACCAGAAACCCT
TTGATGACGCAAGAAATAACCATAGCATCATTATGAGGAAAATCCTTGAGCTGAAGATCCTCCTGGGAGAAGGTGATTGGAACGTGGGACCACCTTGACTTGATG
AAGGGTCCCTGCACTCCAACATGTTGTACCCTTCTCTGCGCCTCCTTCTTTTTCTTTTTGTTGGCTGGCTCTGAGCACGAACCACCTGTTATTGGGAGCAGCAGCTT
CGGGGCCGAAGCAGCTCCAGCTTGATTGTTGGACGAAGCCATCAGCTCAAAAAAGTGGAAGTGAGTTCACCGGAGGTGGGCGCCAATGTTGGGGACTTGTTCT
CAAACGCTATGAATTAAGAACAAGGCAACATAAAATGTTAAATGATAATGTCCTTCGTCCAATGAAGCATTATTCCCTCGGGGATAATGCACTCTGGACGAAGGCGA
ACGACAATACGATTACTAAGGTTGAGTTTTCGTAATTGTACGTTAATAAATTGTACGAAATAACATAAGATATAGAGTATCAAAAGTAAATGAATCATATTATATCCATT
CAATATATTTAAGTATTGGATACAATTATACCTCTGCCTTGACAAAGGTTGGTTCTCAAATGATGAGATGATAAAAAATCTAAAAAACCGTGAACAGTAGAGGAATAC
TGTTCACTATTTATAGGCACAGGACACAGCCTGTGAGAATTTACAACCATGCCCTTCATGAAAGTTTACAATAACGACTCAAACGCTTATGGACTAAAAGGTCTTTCT
ACTTTTAAGTCGGTTCATAATTCCGAAGCTTTATGAAGAGCAAGCTTCGGTCATCGCACGCGGACAGCTTCAGCTGAAGCTGCTTCTTCCTTCAAGACCTTCGGCG
CGACGAAGCATAGTCCCAACACCAGTGGTGTTGTGGTATGGTGGATAGCCTCTGATCCAGCAAGTACTTGAGGCTGTAGTGATCGGTCTTGACTTCGAAAAGGCG
CCCCCAAAGGTAGGGCCTCCAATGGCGGACCGCCTGGACGAGGCCGATCAGCTCCCGCTCATAGGCGGCGAGAGCGCGGTGACGAGGCGCCACGGGACGACT

GAAGAAGGTCATGGGGTGGCTGTCCTGGACCAGCACCGCCCCGAAGCCATGGGACGAAGCGTATTCGACGACGAACGTCTTGGAGAAGTCCGGCATGGCGAGT
ACCGGAGCCAACGTGACCGCCTCCTTCAGAGCGGCGAAGGCAGCCGTCGTCTCGTCCGACCACAGGAACCCGTCCTTCTTGAGCAGGGCGGTCAGCGGCGCGG
CGATTGTGCCATAATTGAGCACAAACTTCCGGTAATACCCGGCGAGATCGAGGAAGCCGCGAACCGCGCGTGCCGACCACGGCTGCTGCCAGTCCCGCACCGCCT
GCACCTTGGCGGGGTCCATGGCGACTTCGGCCGCCGAGACGACGTGTCCCAGGTACGCGACGGAGGAGGCCGCGAAAGCACACTTGGAGCGCTTGACGAAGA
GTTGGTGCTGCTGAAGCGCGCCGAAGACAGCACACAGGTGACGGAGGTGGTCCGCCCATGTGCGACTGTAAATCAAAATGTCATCAAAAAACACAAGCACGAA
GCGGCGGAGGAAGGGACGCAGGACATCGTTCATCAGGGCCTGGAACGTGGCGGGAGCGTTGCACAGCCCGAACGCCATGACCAAGAACTCGTAGAGGCCGTC
ATGTGTGCGGAACGCCGTCTTGTGCACGTCACCCAGCCGCATGCGGACTTGGTGGTACCCGGAGCGCAAGTCGAGCTTGGTGAAGAAGCACGCCCCATGGAGCT
CGTCCAGCAGCTCGTCGACGACGGGGATAGGGAACGCGTCCTTGATTGTGAGCGCGTTAAGTGCCCGGTAGTCGACGCAGAAACGCCACGACCCGTCCGGCTTC
TTCACGAGGAGGACCGGGAACGAGAACGAGAATGCGAAGTCGCTGCGACGCACGATGCCTTGTGCGATCATAGCTGCACATTGTCGCTCCAACTCATCTTTGTGG
GCCGCCGGGTAACGGTACGGCCGGACCGCCACCGGCAGCGTGCCCGACTTAAGAACGATGTTGTGGTCACGCGCACGCGGCGGTGGCAGTCCGGAGGGCTCG
GCGAAGAGACCGCTGAACGCGGTCAGCAGCTCCTCTAGCAAGGCTGCGGGCGCCGTGATGTTGTCGAGGCGCGGTGTGCGCCGGGCGGCCACGTCCTCCCAGC
AGACAGGGCGGCCATGGCGTGTGAAGGCCACAGTGCGCGTGGTGAAGTCCCACACCATCTTGCCTAGCGTGACCATCCACTGGGCGCCGAGGACGACGTCGTA
GCCGGCCAGCGGCATCACGTAGAGGTCGACGCAAAAGTCCTCGCCTGCGATGGTGATCGGGGCCTGACGGAGAATGCTCGGGCAGGCGATGCGCTCGCCATTC
GCCACGGTGGCCGTGAGGCGTGGGTTGGTCTGAACAGCAAGCCCCATGCGTGACGCTGCGGCCTCGGCGATGAAGTTATGCGTGGAGCCAGTGTCTAGAAGGG
CAGTCAGCATCACGGCGCCCACTTGCACGCGCACCTGCATGGAGTCACAAATGGGCATACCAGCCACAGCGCGAAGGGAGAACAATGGTGCGGAGGTGTCGAC
GGCGTCGGCCGCCTGGGCAGCGTCCTCCAACTCGAAGCCGTCAATGTAGAAGATGCGGCGGCACACCCGATTGTGCCCGCGGGAGTACGTCTCATTACAATTGTA
ACACAGGCCAAGACGACGACGTTCCGCCTGCTCCTCCGTCGTAAGGCGCTTGGCAGCCCCAGCCGCGCCGCGGGGAGGAGGTGCCGGGAAGGCCAACTGCTG
TGGCGGGGCTGGAAGGACCGGCACATGGAGCACCGGAGGCGCGCCGGCCGCTGGCAGCGCTGGCCGTGGAGGTGGAGCGGGCAGGGTCGCGCGCGGGGCT
GCCCGGCCGAGGAGGGGACTGATCCGGTCCAGCTCAATCAGCTCCAGCGTCCTGGCCAGGCTCATGGCTGCGGCCAAGGACTCCAGCGCGTGAACGCGGACCT
GATGGCTTAGCGGCAGCAGCAGCCCGCCGGTGTACAATTGTACGCGCTGGCTTTCCTGCAGCCGGCCAGCACGCGGAAGAAGCGCCTGGAAGCGGTTGGAGTA
TTCTTCCACCGATCCCGTGCGTCGGCACTCCGTCAGCTCGAACAGCGGTGCCGATCTCAACACCGGTCCAAACCGGAGGTTGAGGAGTTCCTTGAAGCGACCCCA
GGGCGGCGTGCCTTCATCCTCCTAGAGTTGGAGGAACCACAGCTGGGCCACGTCGTCCATGTTGTAAGACGCCATCCACACCTTTTCCTCCGGCATGGTGCGTTGT
TGTCGAAAGTATGACTCACACTTGTTGATGAAGATCAGCGGGTCGGACTTGCCGTCATAGCGCGGGAACTCCATCCTCTGGAACTTCGGCGGGTGATCCGTGCGG
TGCTGCCCATCTTGGTGGCTGCCGGAGTCGGCCGAGGAGGTGACCTTCTCCTGCATGCTATCCATGTCGGATTGCATCTTGGACATGTTGGAGGACAGGGTCTGTA
GCATCTTCATGACGTCGGCCATGGAGGGTTGAGCGCCGTCACCCATGGTGTTCAATCTGGACGTAGCGGTGGACGGGGAGGTGGGGGCTGGTGTTGTCGGCTG
GAAGGGTGGTGCGGCTACGGTGGATGAAGGACTGGAGGTGGTGGTGGAGGATCGCCGACTTCCTGATACCAGGTTGTCAAGGGCGTCGAGCCCGAGGGTAGG

AGGACCACGGCTCCGAAGATCGTAGCCGCAGTCCGTGTTGCCGACCGGGTTATGCCCCAGGAACAACGTTCTTCGTCAACGGGTTATTGCCCCCTGTTGCCGACT
TGAGAGAGAGAGACAGGGATGATGGGATAAATCTTTCTTCTCAAATCACGAGTGTTTACAGGGAATATAAGGCCATAGGCCAGGACTAATAAAGGAAAGCCACCA
ATTAACTTTTATTCATTCTAAATCTAGCCGCTAGGCCCGGCTGCCTGCTGGATCAGGTGCTCTGCGCGCTCAGCCGCGCGCCGTACGTCGCGTGCCCTGTGACGCCG
GGTGTACGTGTTTCCGTACATCACACATACTAAGAATTCCCTTGATTCATAGTAGTTGCATGCTCTCCTGTGTGATATTTGTGGATGACCTATTGTTGACACTAATTCGG
GCATTGTAACATAATTACAATCAATATTTTTGAGAAATATATTGCAACCTTTAGGCAATATACGCATGTTTTTGCCTGTCATGTTGTTGGTTGTAGTTAAGTCCATTATTG
TTACTCTTTGGAGTCTGTGCTGTTGGGGTTGGGTAGGTATGTGAGTCTGTTGTTGCCTCCACAAGTCTCCAATAAATCACACAAGATGAGAGCTGTTGCTGGTGTGT
CCTACCTCGTCATTCTTTGACGGTTGCTATTCAGGTAGCCCTTGCAATAAATTAAGACAGGGGAAGAGAGAAGGAAAGGGGAACAATGAGGAAATGCTCATATGG
CTCTGGAATTCAATAAATTAGTCATATAATGATATTGGTCCAAAATTTAAGTTTTGGCTTTGGCCTAATTAGAAGAATGTAACCATGAAGGGTCTTATTTTCATTCATGC
TCTACGCAGACAATAATATGAGGTGAAGGATGAAATAATACCATAAGATTTGATTATGGAATGTGTTTTTTTGGTTAAACACCTAAATTATTGAGTTATCGGCTTAATT
CATGTGGCTGTTAATGCTTAGTATCATGACCGTTCTGTTGAACTCAAAAGACACTCAAGTGGATTAAAAACAATACGTGGTGAACTGATAAAATGGTCAATAATACAT
GTTCCAAACAATTCACAATAAACTAACAATTGTGGAAATGCCAACGAAATTATACCTTGTTTACTATCCTAAAAAAATATACTGCGATTTTTGTTTAGCACACTGAAAT
TATGATACGATAACCATGAATGGTTAAGATCATTTTTAATAGCACCAAGCAGTATATAAGCTATCTATCTCCCTTGAAACATGATATCAGCGCCGTACTAAAATAGTCGG
ATGTTAATTTATTTTCACTGTAATGCACGGGCGTATAATGCTAGTAGCGCAGAAAATGTTGGAGCATTCTCATGTTTGCTGTCGGCGGTAGGTTGTGTACAAAAAGTA
CTCAAACAAATATTTCACTTCCACTTTAACTTGTATTATGTTCTCATATGGACTAAAATTAATTTAGTCGCCTACATGCATGCACCTGCATATCCATTATTATATTGGGAG
GAGCCTATTTTTCTATACAACTGGACTAGATAACTTTATGTTTCCTATTATGTTTGTTAAACCACTAGCAGTATTCATGTTGCAATTAAAAACTAACATGACTGATGATG
ATTTGACTTTTTGCCTAGTTTAAAGAATGCGCTATTTCAAAATAGTTCAGATCAATATATGGATAACATCTGGGGGAATTGTAAGATTCTAGGCTTGGCCTTGTTTTGT
TTGTTGTTTTTCATCTATATTAGTATATGTATATACGTGGTCATCATGTTGCCATGCATGACACTTTTGTCAACTGAAATACTTTTTCTGTTTAGTTGTTCGTTTGACTTTA
ATCAAAATGACTTCTACTGTTTCTATAATGTTTTGTCTCTATGGATTGCAGTTACAGTAGTCCGAACAGTTGGCTTTAATTTGAAGCGACTCACACAAAAATTATTGAA
TATAAGTATGTGATGTTATCCACTTGCTTGATCAAAGGGAAAAGGTAGCAGGGGTTGGGAAGAACATGCTGAACACGCGTCCACTTGCAAGCATGGCAGAACTTT
AGTTCTAATGGTTCCTGCTCTTTTTACAGGCAGTTTAGATGATCCATATAATTGTGCATGTTGCATGCAAGTTGCAACAGCATACATTTGTAAACGGATCTCGCGTACA
TATAATCGCCTGTTGCCAAGGGATAATCTTTCGTATACTTAGTATAGTCGGTCGAGGGATTTCTTGATTTCCTAAAGAAAGAAAAATTAAAAGGAAAATAAAAAAACT
AGTTTTTGAGATGGCATCTAGGTCAAGACATCTCGGTCTTAGCTCAATTAGAAAGTAAAAAACGGATTCTAATAAGAAATTCCCAAATTAAAAATTCGTTTGTTTTAG
GCAATTAATGAATTCATCAAGTAAGTATGTGTCTTATATATATGTCTAAATTTATCATTATTCATTTGAATATTGAAAGTCGGCTAGAGGGGGGTGATTAGGCAAATCTG
AAATTTATAAACTTTAAGCACAACTACAAGCTGGGGTTAGCGTTAGAAATAAAGTCGAGTCCGAAAGAGAGAGTGAAAACAAATCACAAGCAAATAAAGCAGATG
ACACAGTGATTTGTTTTACCGAGGTTCGGTTCTTACAGACCTACTCCCCATTGAGGTGGTCACAAAGACCGGGTCTTTTTCAACCCTATCCCTCTCTCAAACGGTCA
CTTAGACCGAGTGAGCTTTCTCCTTAATCAAACGGGTCACTTAGACCCCTCACAAGGACCACCACAACTTGGTGTCTCTTGCTTTGATTACAAGTGTCTTGAGAATA

AGAATGGGAAGAAGAAAGCACGATTGCAAAAGTCAAGCGACAAGAGCGACAAATAACACACAGATCACTGTCTCTCTCAAGTCACTAATCACTAATGATCACTTG
TCTTAATTGTGGAACTTGGAGAGATTGGAAGCTTTGATTGTGTCTTTGAATGGATTGCTAGCTCTTGTATTGAATGCAGAGGATTGGAATGCTTGGTTCAAGTGAAT
GGAGGTGGTTGGGGTAGTATTTATAGCCACCAACCACTTCCTAGTCGTTGCTCCAATTCTGCCAACCGCGGACGGTCCGCGCCCCTGGTCCGGACAGTCCGCCCCT
GCACATCAACGGCTGAAAACGCAACGGTCAGCAGTAACGGCTATATCAACGACTATTTTACATTTAATGCGTCGTCAGATGTCAGATAAAGGCAGTCGCGGATGGT
CCGGTCGTGCACCCCGGACGGTCTGCGAGGACACTATAATTCATTGTACCGAACCTGTCACCTTCGGGTTTTTCGGTTTTTCACCGACCGGACAGTCGACTTAGAA
TAGTTGTAGATGAACTTATGCACGTGTGAAATGATCAGCTAGGCAAACTGGTTAGTCCACAAGGTTTGTGATGGTCATCAAACACCAAAATCAATTATAGGAAATGT
TGAGACTATTTCCCTTTCAATCTCCCCCTTTTTGGTGGTTGATGCCAACACAAACCAAAGCAAATATAAATTGTAGAAACGTAACTAGTTTGCATTTTGACAGATGTG
CATAAGTTATTTTGACATGAAAGCATTTCTAAGTATATAAGTTTGATTTGATATTTAACATTTTGGACCACATTTGCACCACTTGTTTTGTTTTTGCAAATTCTTTTGGA
AAATCTTTTCAAGGTTCTTTTGCAAATAGTCAAAGGTAAATGAATAAGATTGTGAGAAGCATTTTCAAGATTTGAAATTTTCTCCCCCGGTTTCAAACGCTTTTCCTT
TGACTCAAACAAAACTCCCCCTTAATGAAATTCTCCTCTTAGTGTTCAAGATGGTTTTACTAATTGAAGGAAGATCAAAATATTTTAGGTACCAATTTTTGAAAAACT
CCTCCTTAAAATATAGATACCAATTAAGATAGAATTTCTTAGAGGAATACTAATTTGAAAGATACCAATTGAAGACATTTAACAATTTAGACTTGTTTCGAAAATTTTT
GAAATTGGCGCGTGGTGGTGCGGTCCTTTTGCTTTGGGCTAATACTCTCTCCCCCTTTGGCATTAATCGCCAAAAACGGAGTCTTTAGAGCCCTTCTTACTTTCTCCC
CAATGGTACAAATGAATATGAGTGAAGATTATACCAAAGTGGAGAGTGATGCGGAGTGACGGCGAAGGGTAAATAATACCGATAGAGTGGAGTGGAAGCCTTGTC
TTCACCGAAGACTCCATTTCCCTTTCAATCTATGACTTAGTAGAAATTCTCTTGAAAACATATTAGTCATAGGCATAAAAGAGATATGATCAAAGGTATATAAATGAGC
TATGTGTGCAAAGATTTAATCAAAGTTCCTAGAATCAAGAATGTTTAGCTCATTCCTAAGTTTGGTAAAGGTTTTCTCATCTAATGGCTTGGTAAAGATATCGGCTAGT
TGTTCTTTGGTGCTAACATAAGCAATCTCAATATCCCCCTTTTGTTGGTGATCCCTCAAAAAGTGATACCGAATGGCTATGTGTTTAGTGCGGCTGTGTTCAACGGGA
TTATCCGCCATGCGGATAACACTGTCATTATCACATAGGAGAGGGACTTTGGTTAATTTGTAGCCATAGTCCCTAAGGGTTTGCCTCATCCAAAGCAATTGCACGCAA
CAATGGCCTGCGGCAATGTACTCGGCTTCGGCGGTAGAAAGAGCTACTGAATTTTGTTTCTTTGAAGCCCAAGACACCAGGGATCTTCCCAAGAACTGACAAGTT
CCTGATGTGCTCTTTCTATCAATTTTACACCCTGCCCAATCAGCATCTGAATATCGTATTAAATCAAAAGTGGATCCCTTGGGGTACCAAAGACCAAACTTAGGTGTAT
GAACTAAATATCTCAAGATTCTTTTCACGGCCCTAAGGTGAACTTCCTTAGGATCGGCTTGAAATCTTGCACACATGCATACTGAAAGCATAATATCCGGTCGAGATG
CACATAAATAGAGTAAGGATCCTATCATCAACCGGTATACATTTTGATCTACAGATTTACCTCCCGTGTCGAGGTCGAGATGCCCATTTGTTCCCATGGGTGTCTTGAT
GGGCTTGGCATCCTTCATCCCAAACTTGGTGAGTATGTCTTGAATATACTTCATTTGGCTGATGAAGGTTCCCTCTTCGAGTTGCTTGACTTGAAATCCTAGAAAATA
CTTCAACTCCCCCATCATAGACATCTCGAATTTTTGAATCATAATCCTACTAAACTCTTCACAAGTAGATTTGTTAGTAGACCCAAATATGATATCATCAACATAAATTTG
GCATACAAACAAATCATTTGCAATAGTTTTAGTAAAGAGAGTAGGATCAGCTTTGCCGACTTTAAAACCATTAGTGATAAGGAAATCTCTTAGGCATTCATACCATGC
TCTTGGGGCTTGCTTGAGCCCATAAAGCGCCTTAGAGAGTTTATAAATGTGATTAGGGTACTCACTATCTTCAAAGCCGAGAGGTTGCTCAACATAGACCTCTTCCT
TGATTGGTCCATTGAGGAAGGCACTTTTCACGTCCATTTGATAAAGCTTAAAGCCATGGTAAGTAGCATAGGCAAGTAATATGCGAATTGACTCAAGCCTAGCTACG

GGTGCATAGGTTTCACCGAAATCCAAACCTTCGACTTGTGAATATACCTTGGCCACAAGTCGGGCTTTGTTCCTTGTCACCACACCATGCTCATCTTGCTTGTTGCG
GAAAACCCACTTGGTTCCTACAACATTTTGATTAGGACGTGGAACTAAATGCCATACCTCATTCTCGTGAAGTTGTTGAGTTCCTCTTGCATTACCAACACCCAATCC
GAATCTCGTAGTGCATCCTCTACCCTGTATGGCTCAATAGAGGAAACAAAAGAGTAATGCTCACAAAAATGAGCGACACGAGATCGAGTGGTTACCCCCTTATGAAT
GTCACCGAGAATGGAGTTCACGGGGTGATCTCTTTGTATCGCTTGGTGCACTCTTGGGTGTGTCGGCCTTGGACCCTCATCATCTTTCTTGTCTTGATCATTGGCATC
TCCCCCTTGATCATTGTCCTCCTCTTGAGGTGGCTCTTCTTCTTGATCTTCATTTTCATCATCTTGAGCTTGATCCTCATCTTGGGTTGGTGGAGATGCTTGCATTGAA
GATGGAGGTTGATCTTGTGCTTGTGGAGGCTCTTCGGATTCCTTAGGACACATATCCCCAATGGACATGTTTCTTAGCGCGACGCATGGAGCCTCTTCATCATCTAG
CTCATCAAGATCAACTTGCTCTACTTGAGAGCCGTTAGTCTCATCAAACACAACGTCACAAGAAACTTCAACTAGTCTAGTGGACTTGTTAAAGACTCTATATGCCCT
TGTGTTTGAGTCATAACCAAGTAAAAAGCCTTCTACAACCTTAGGAGCAAATTTAGATTTTCTACCTCTTTTAACAAGAATAAAGCATTTGCTACCAAAGACTCTAAA
ATATGAAACATTGTGTTTTTTACCGGTGAGGAGTTCATAGGATGTCTTCTTAAGGATTCGGTGAAGATAGAGACGGTTGATGGCGTAGCAAGCGGTGTTGATTGCT
TCCGCCCAAAACCGGTCCGAAGTCTTGTACTCATCAAGCATGGTCCTCACCATGTCAAGTAGAGTTCTATTCTTCCTCTCCACTACACCATTTTGTTGAGGTGTGTAG
GGAGAAGAGAACTCATGCTTGATGCCCTCGTCCTCAAGAAAGCCTTCAATTTGAGAGTTCTTGAACTCCGCCCCATTGTCGCCTAATCTTTTTGATCCTTAAGCCGA
ATTCATTTTGAGCCCGTCTCAAGAATCCCTTTAAGGTCTCTTGGGTTTGAGATTTATCCTGCAAAAAGAACACCCAAGTGAAGCGAGAATAATCATCCACAATAACT
AGACAGTACTTACTCCCGCCGATGCTTATGTAAACTATCGGGCCGAATAGATCCATGTGGAGTAGCTCAAGTGGCCTGTCGGTCGTCATGATGTTCTTGTGTGGATG
ATGAACACCAACTTGCTTCCCTGCTTGACATACGCTACAAACCCTGTCTTTCTCAAAATGAACATTTGTTAGTCCCAAAATGTGTTCTCCCTTTAGAAGATTGTGAAG
ATTCTTCATCCCAACGTGGGCTAGTCGGCGATGCCAGAGCCAACCCATATTAGTCTTAGCAATTAAGTAAGTATCGAGTTCAGCTCTATTAAAATCAACTAAGTATAG
CTGACCCTCGAGTACTCCCTTAAATGCTATTGAATCATCACTTCTTCTAAACATAGTTACACCTATATCCGTAAAAAGACAATTGTAGCCCATTTTGCATAATTGAGAAA
CTGAATGCAAGTTGTAATCTAAAGAATCTACAAGAAAAATATTGGAAATAGAATGGTCAGGAGATATAGCAATTTTACCAAGTCCTTTGACCAAACCTTGATTTCCAT
CCCCGAATGTGATAGCTCTTTGGGGATCTTGGTTTTTCTCATAGGAGAACATTATTTCTCCCCAGTCATGTGGTTTGTGCACCTGCTATCAATGATCCAACTTGAGC
CCCCGGATGCATAAACCTACAAAACAAGAGTTTATGCCTTATTCTTAGGTACCCAAACGGTCTTGGGTCCTTTCACATTAGAAACAAGCACCTTGGTTACCCAAACA
CAAGTCTTGGAGCCTTTGTGTTTGTCCCCAACATATTTGGCAACTACTTTGCCTGATTTGTTAGTTAAAACATAAGAAGCATCAAAAGTCTTAAATGAAACATTAGGT
TCATTTGATGCAATAGGAGATTTCTTTTTAGGCAATTTAACATGAGTGAATTGCCTAGAACTAGATGCCTCACTCTTATACATAAAAGCATGATGAGAGCCAGAGTGA
GACTTCCTAGAATGAATTCTCCTAATCTTATGCTTGGGATAAGCAGCAGGATATAAAATATAGCCCTCGTTATCCTGAGCCATGGGAGCCTTACCCTTAACAAAATTAG
ACAATTTCTTAGGGGCATTAAGCTTGACATTGTCTCCCTGTTGGAAGCCAATGTCATCCTTAATGCCAAGGCGTCTCCCACTATAGAGCATACTTCTAGCAAATTTAA
ATTTTTCATTTTCTAAGTCATGCTCATTAATTTTAGCACTAAGTTGAGCTATGTGATCATTTTGTTTTTTAATTAAAGCTAGGTGATCATGGATAGCATCAACATTAATGT
CTCTACATTTAGTACAAATGGAAACATGGCTAACAGTAGATGTAGAGGGTTTGCACACATTTAATTCATCAATCTTAGCATGTAACATGGCATTCTCATTTCTAAGATT
GGAAACAATATCATTGCAAACATTTAAATTCTTGGCCTTAGCAATTAATTTATCATTTTCAATCTTAAGGCTAGCAAGAGATTCGTTCAATTTATCAATCTTAGCAATTT

AACTAGCATTATCCTTTTTAAGATTGACAATCGAATCATAACAATCATTTGATTTTTCAACCTTAGCAAATAAATCACGGTTCTCATTCCTAAGGTTGGAAATGGTGTC
ATGGCAAATGCTAAGCTCCTTAGTCAAATTTTCATTCTTTTCTATTTCTAGAGCATAAGAATTTTTTACCTTAACATGCTTTTTATTTTGCTTAATAAGGAAGTCCTCTT
GGCTATCCAAGAGTTCATCCTTATCATAAATAGCACTAATTAATTCATTCAATTTTTCCTTTTGTTGCATGTTTAGGTTGGCAAAAAGAGTAAACAAATCATCCTCATTA
TCACTAGAACTAGCCTCATCACTAGATGTTGCATATTTAGTAGAAGCTCTAGATTTTACCTTCTTCTTTTTGCCGTCCTTTGCCATGAGGCACTTGTGGCCGACGTTGG
GGAAGAGGAGTTGACGGCGATGTTTGCGGCGTCCTTGTCGGAGGAGGAGTCGGTGGAGCTCTCATCGGAGTCCCACTCCCGGCATATATGGGCATCGCCGCCCT
TCGTCTTGTAGTACCTCTTCTTTTCCCTCCTCTTTCCCTTCTTGTCGTCATCCCTATCACTATCACTAGATAAAGGACATTTAGCAATAAAATGACCAGGCTTACCACAC
TTGTAGCAAACCTTCTTGGAACGGGGTTTGTAGTCTCTTCCCTTCCTTTGCTTGAGGATTTGGCAATGATGATTAGCGCCATGTCCTCATTGTCGAGCTTGGAGGCG
TCGATGGGGAGCCTACTTGATGTAGATTCTTCTTTCTTCTCCTCCGTCGCTTTGAATGCGATGGGTTGCACCTCGGGTGTGGAGGTGGCGCCTCGCTCAATGATTTG
TTTGGAGCCTTTGATCATCAACTCAAAGCTCACAAACTTTCCTATAACCTCCTCGAGAGACATTAGCTTGTATCTAGGATCACCACGAATTAATTGTACTTGTGTAGG
ATTAAGAAAAACAAGTGATCTTAGAATAACATTGACCATTTCATGGTCATCCCATTTTGTGCTCCCGAGGTTGCGCACTTGGTTGACCAAGATCTTGAGCCGGTTGT
ACATAGCTTGTGGCTCTTCTCCTTGGTTGAGCATGAACCGACCGAGCTCCCCCTCGATCGTTTCCCTCTTGGTGATCTTGGTCACTGTTGGGACCATGCTTCGTCGC
CGAAGGTCCTGCAGAGAGATACAGCTTCGGCTGAAGCTGCTCGTACGTGACGCCGAAGGCATCATTCATGAAGCTTTGATAATACAACATATTCGAAGGAGAAGG
GACGAACCGACTTAAAGATAAAATGACCTTTTAGTCCATAAAGGTCTGAGTCAATGTTGTAAATTTTTATGAGGGGCATGATTGTAATTCCTCACAGGCTGTGTCCT
GTGCCTATAAATAGTGAACAATATTCCTTTACTGTTCACGCATTCCTGCAATTGTAATCGCATCACTTAGAAATTATTCTTCGCCAAGACAGAGGTACAAATGTATCTAA
CATTTGAATACATCAAATTTGTTTAATATGAAAATGATGATTATTTATAATTTACTTCTTATTAATGCTTCATATCTCGAATTATTTTATACAACCTCTGGGAGTTCAATTAC
GAAGATCCAACCTTCGTAATTGTTTCATCTTTGACCTTCGTCCGAAGTTCATTAAATCTGTAAGGAAATAATGCTTCAGCGGACGAAGGGCATTAACATTTAATATTT
TATGTTGCCTTGTTCTTAATTCATAGCATTTGAGAACGAGTCCCCAACATTGGCGCCCACCTCCGGTGAACTCACTTCCACTTTTCTGAGCTGATGGCTTCGTTCAAT
ATTCAAGCTGGAGCTGCTTCGGTTCCGAAGCTGGTACTCCCGATAACAGGCGGTTCATGTTCAGAACCAGCCAACAAGAAGCAGAAGAAGGAAGCACAGAGAA
GGGTACAACATGTCGGGGTGCAAGGACCCTTCATCAAGTCAAGATGGTCTCACATTCCTATTACCTTCTCTCAAGAGGACCTTCAACTCAAGGATTACCCACACAAT
GATGCTATGGTTATCTCTTGAGTTATCAAAGGATTTCTGGTCCACAATGTTTTAGTTGATACAGGCAGCGCATCTGATATCATATTTGCTAAGGCCTTCAGACAGATGC
AAGAGCCATAAGATAAAATTCTTGATGCCACACATCCCCTCTGCGGCTTCGGAGGAAGGCAGATTGTAGCACTCGGCAAAATCTCAATGTCAGTGGCCTTCGGATT
CATCAACAACACAAGGACTGAACAAGTTATGTTTGATATTGTTGACATGGAGTACCCTTACAATGCAATCATTGGTCGTGGCACTCTTAATGCCTTCGAAGCAATTCT
GCATCCCGCTTATCTTTGCATGAAGATACCTTCGGACCAAGGGCCCATTGCTATTCATGGAAGTCAAGAAGCTGCCAGAAGGGCCGAAGGAAATTGGACAGACTC
AAAAGCAATCCATAATATAGATGGAACTGAAGCTTGTGAACAATACAAGTTCAGAAGGGAAAAAGCTGCTTCAGCTGACCAGCCGAAACCCATGCTCCTATGTGA
GGATATAGCAGAGCAGAAGGTGCTATTGGGATCTCAATTGTCCGAAGAGCAGGAGAAAACCTTGATAAGGTTTCTGTTCAACAATAAAGATGTTTTTGCTTGGTCA
GCTAATGATCTTTGTGGAGTTAACAGGGATGTTATCGAGCATTCGCTCAATGTTGATCCATCCTTCAGGCCCAGAAAGCAGAGGCTTCGGAAAATGTCTGAAGACA

AGACCGAAGGTGCTCGAAATGAAGTAAAAAGACTCCTCAGTGCCGGAGTTATTAGAGAGGTAAAATACCCAGAATGGTTGGCTAACACTGTTATGGTGAAGAAG
GCCAATGGTAAATGGAGAATGTGTATCGATTTTACTGACCTCAATAAAGCCTGTCCGAAGGACGAATTTCCATTGCCAAGGATAGATTCCTTAGTTGATGCAGCAGC
TTCATCAGAGCTTATGAGTTTGCTGGATTGCTATTCAGGCTATCATCAAATATGGATGAAGAAGGAAGATGAACCAAAAACCAGCTTCATAACCCCTAGTGGGACAT
ATTGCTACCTTCGGATGCCTGAGGGGCTTAAAAATGCTGGAGGAAGTTTCAGCAGAATGACAGCGAAGGTCCTTCACTCTCAGATAGGAAGAAATGTGCTAACTT
ATGTTGATGATATCATTCTAAAAAGCACGAAGCAAGATAACCACATTGCTGATTTGCAGGAGACCTTCGCTAATTTTAGACAGGCTGGTCTAAAATTGAACCCAGAA
AAATGTGTCTTCGGAGTAAAGAAGGGGAAATTTCTCGGTTGCCTAGTCTCAACAAAGGGAATCGAGGCTAATCCAAGCAAAATCGAAGCAATACTTCGAATGGAA
CCACCAAGTACAAAAAAGGGGGCTCAGAGATTGACGGGAAGGTTGGCGTCTCTCAATAGATTCATATCTAGATCAGCAGAGAGAAACTTACCATTCTTCGAAGTG
CTGAAGACAGCCGAAGTCTTTCAATGGGGACCAATCCAGCAGAAGGCCTTCGAAGAGCTGAAGCAGTATTTGATAGATCTCACAACATTAACTCCACCTACGCCA
GGGGCTCCTTTGTTATTATATGTGGCAGCTTCACACTCAGCGGTAAGTGCAACACTTGTTCAGGAGAAGCTTGAAGGCCAAGTTAAGAGGCAGGCCCCAATATATT
TTGTATCCGAGGTTCTTAGTTTATCAAAGAAAAATTATACAGAGTTGGAGAAGGTACTGTATGCTGTCTTGATGGCCTCCAGGAAGCTTCGGCACTATTTTCAAGCC
TACAACATAATTGTTCCCTCTTCACAACCTCTGAAGGATATTATGAGGAACCGAGAAGCTACTGGAAGGATTGGAAAATGGGTTGCAGAGCTCAATGAATTTTGTAT
TGATTATGTTCATAGATCTTCGATTTAGTCCCAGGCGTTAGCAGACTTCATTGCTGACTGGACGCCAGGGGCTCAGGAGGAAGAAACAAATAAAGACGCCGAAGC
ATGGACAGTGTTTTGCGATGGGTCTTGGGGAACCTTCGGAGCGGGAGCGGCTGCTGTGTTGGTTTCACCTTCCAAAGTTAAAACTTGTTATGCGGCAAGACTTGA
TTTTAGCTGCACAAATAACATTGCCGAGTATGAAGCTCTGTTTTTTGGTCTTCGGAAATTAAAGGCAATGGGAATCAGAAGGGCCATTCTTAAAACTGATTCTCAAG
TTATTTCTGGTCATATTGACAAAAGTTACAAAGCAAGAGACCCGAAGCTTGAAAAGTATCTAGATACAGTCCGAAGGATTGAGGCTTCCTTCAAAGGTTTCTCTGTC
AAAAATATTCCTCGTGGAGAGAATGAATACGCTGATCTATTGGCTAAGTCAGCAGCCCAGGGGCTGCCTATGCCTTCGGAAGTATTTTTTGAAACAATAAAAGCAC
CTTCGGTTGAGCTTCTTGAAAGAGCAATCCTTAACATATCCCCTGTTTATAGTGAAGATTGGAGAACTAAGATCATCTCTTTCCTTCAGGGTAATTTTCTTTCAGACG
ACGAAACTTATAACAGGAGAATAGAAGCAAGAGCTCGACCATATGTCATAATAGAAGGGGAGTTATACAAGCGCGGGGTCTGTTCCCCATTGCTCAAGTGCTTATC
CAGATCTGAAGGTATAGAATTAATGAAGGACATACATGCAGGTCTGTGTGGATCTCACATTGGATCTAGGCCCTTGCTTGGGAAAGTTTTTCGCCAAGGATTTTATT
GGCCAAAGGCAGCTTCGGATGCAGCGGATTTAGTTCAAAAGTGCGAAGGTTGTCAGAAATGTGCAAGAGATAAAAAACAACCTTCGTCTTTAACTCAATTAATAC
AACCCACTTGGCCGTTGCAAAGGTGGGGTCTGGATTTGCTAGGACCATTACCACTAGCACAGGGGAACTTAAGATATGTGGTGGCAGTGGAATATTTTTCCA
AATGGATTGAGGCGAAGCCCTTAGCCACAATAACTTCATTTACTATTCAAAAGTTTTTTTGGCAAAACATTGTTTGTCGCTTCGGAGTACCGAAGGCTATCACTGTG
GATAATGGGACACAGTTTGACTCTGAAGCCTTCAGAGAATTTTGTAATCAAATTGGTACGAAGATCCATTTTGCATCAGTTCGGCACCCAGAGTCAAATGGACTTGT
CGAAAGAGCCAATGGGATCATAATGACAGGAATAATGAAGTCAATCTTCAATCAGCCCAGGGGAAAGTGGCCAGATGAGTTAATCAAAGTGGTGTGGAGTCATAA
CACAACCATGTCAAGATCAACAGGCTTTACACCCTTTAAACTATTGTTTGGTGACGAAGCAATAACCCCAGAAGAGGCCAAAACAGGATCAATAAGGACAGTAGCT
TCGGCAGAGGGCGAAAACGAAGCTGATTACTCTGTAGAAAAAGATGCTATTGAAGGGATCAGACTTCAAGCTGTGGAAAACATCAATAAATATCAAGCTGAAACA

ATAAAATGGCGTGATAGAAAAGTCAAGCTGAAGAACATTGAACCAGGACATTTGGTACTTCGAAGAGTAGCTAACCCTGAAACAGTAGGCAAATTACAGCTAAAG
TGGGAGGGCCCCTTTTTGGTAGTATCTTCGTCAAGACCAGGTTCTTACAGATTGAAGGATATGGACGGCAATGACATTTCTAGATCGTGGAATGCAGATGAGCTTC
GGCGATATTATGTTTAGTTCGATGTAATTTTTTATATTCTTTTTTGTGGCACCCTTTTCCTTTCCAAAGGGGGAGAAAGGTTTTTAATGGGGCCACAACATGTAATTTT
TCTTTTTCTTATTTCAATTCTATAAGAGCGATATCCCCCAAAAATGTAAATGTAAAAGCTGAAAATGCACCTTCGAGTGCAAAGGAAAAAGAAAAGAAAACAGGGA
GAAGCTCAAAAGTCGTTCCTAAGGGAATGCAGAGCTTACAGCGAAAAATAAACGCTGATTCCGTCGAAAGTAAAAGGCGAAGAAGCTCCTAAGGGAGGCTTGC
AGCGAAAAATAAACGCTGATTCCGCCGAAAGTAAAAGGCGAAGAAGCTCCTAAGGGAGGCTTGCAGCGAAAAATAAACGCTGATTCCGCCGAAAGTAAAAGGC
GAAGAAGCTCCTAAGGGAGGCTTACATCGAAAAGTCAACGCTGATACACCGAAAAGTAAACGGTGAAGCCGAAAAGTAAACGGCAAAAAGATTTGTATCATTCT
TGAGGGGATGAAGGGCCGTGCTTATGGTTTCTGAACATGTGTCCTAATATTCATTTGCACATTATATGTCATCATGTCATTTGCATTCATAAACATCCATTTAGACATAT
ATAGAATCATCATCATGCTACACAAAGAAGACATGTGCTTCAGAAAATAAGGAAAAGATTCGAAGAAAAATTTTTTATGCTTCGGTGTGTACGAAAAGAAGGGAA
GGTGTTTTTCGCCTTCGGCTCAAAAGAGAAGTTTCGTCCACAGCAAAGCAGATTGAACACGGATAAGGGAGACAAAATATATTACAAGGTATGTACAAATTTACAT
GAGTTGTTCCATATCTATATTACAAGAGTTTCTCCAAGAATTTTTGCAGGAAAGCACATTATAAGCAACTTTAAGCTCCAGCTAAGGCTTTGTTTTCAATTTTGTTAAA
CATCAGCATTGTCAATCTTCAGCTTCGTCATCCTGTATATACCAAACAGCAGAGTAAGAAAGGTACAAATTTCACAGGAGAAGGTAAAAGTAACAAACATAAGTTTC
TTACCGGCTTAAGATGGCTTCGAGCTTCGTCGCCTGCCATTTTCCGCCCGCCACTTACCCAGATCTGGGTCATGAATCTATTTCCGATGCTTCTTGCTAAGCTTGGGA
TATCTATCAAATCTGAAGCTGACAAGCTGAAGTTTGGTCTGTTCACAATGTTTCCATGCGTGCAGCCAGCCTTCAAAAAAGCAGTAGCCGTGCCCCGAGAAGCTAC
CAGGGCGCAGAAGTCCGCGTGCCCACCAATAACTTCGTCGAGGGCATCAACTTCGCCTTCAATATATTCTAATGTTCTTGGCAGATTTTCAGCTGAAGGTGTAAATT
TTGAAGAGCTAGCTCCGACAGAGTGAAACACGTCCTTCAGCCGCTGCACGCATCGGCTGCCGAAGCTTAAGCATTTCTCCTGAAGATCTTTAAAGGATTTCTGCAA
ATTTGAGTATTTATCCACTTCAGCTTTCAGGCTTGCTTCAAGATATTTTTTTTCTTGCTCGAATTTTTCTGATTGTCGGAGCAATTCATTCTTTAATTTGTCATTCTCAGT
TTGGACTTCAGCCAAAGAGCCCTCCATGGTCTGAATAACAAAATCCTTCTTTTCCAGTGCACCTTCGTGCTCTTTGACCATGATTCCAAGGTCTTGGATTATAAGGTC
TTTCTTTTTCAGATTGGCTTCGTGATTTTCAACTTTTTCAGCCAAATCTTGAATGACGGCTTTGTTTTTCTCTTCTTCCAGATCTTGTTGCATTTTTAGAACCTTGCTTA
ATAATATGCTCTGTCGAAACGATCTTCGTTAAAACGTGACCTAAAAGATAGTAATAATAGTAATAAAATAACAAAAATATCCGTTACCTTAAAGTTAGCATAAAGCAAG
CTTCCGGCGATGTGGTGTCGTTGATAACGGCACAAGTCAGTCTCCAGCTTCGGCAAGCTAATGCTTTTGGAGAGGGTTCTAACAATTTTGGCCTCAGTACGGTTCC
GTAGGCATCTTAGTTTCCCTTCGTTCACCCCACCAAATAGCATAGCCTCTGATTTATATCTGCAAGATAGGGCATATTTTTTCAACTCCTCTTTCTCAGCGTCTGTTAAC
TCTTGTCCAAGCAAATCTTGAAAGTTAAAATCTTCTTCTTCCGAAATTTCATCAATTTGCTCTTTTCCCTTTTCAGTTGCCGTGCTCACCGCAGCTGCAGTAGTTTCTT
CTTCCGCCATTTTCAGGAGTATATTGTCAATAACCTCAAGTGTGGTTTCCAGGTTCGACTCTTCGGTGGTCCCGGCTTCGGCTCCGGTAGCTTTGGCTTCGCCGGTC
TCAGCTTCAGTGGTTTCCACTCCAGTAGCTTCGGCTGCGGCGCCTTCGGCTTCGGCAGTCTCGGCAAAGACAATTTTTGACACCGTCACCGGTGGCGGTGTCTGA
TGGATCACATCTGTCACTTGAATAATTCTCCGTTTCTTCAGCTTCACAAGACTTTTTGCTGCCGAAGCTGCCTTGTTCCTCTGAAAAAACTTCGTCAGTTCTAGTGCC

AGCGGACTTAGCCTGATAGGCAGGGGTTCAGTCATTACCTTCATAATCTCTTCTACTTCAACAGCAGAAGGAGAAGCAGGAGTATCTTCTTCTCGGACCGGCGTTT
CTGGCTCTGGGGATGCACTTTTTCTCTTTCTTGTAGTAGCCACCTTCGGCTCAGGATTTAACTCTTCAACGCTGACATTTTCAGAATCTTTCTTTTTCTTTTTGGTTAC
CTTGACGGCTTCTTCGTCAGTAGCGCTGACAATCCTTTTTCGCTTCGGGTCTCCAGCATCTCCGCCCAGTCGGCCGTAGTCAGCATATTCAAATCCTATGGCATCAAA
CACCCTGTTCAGCCTTCGTTTCGGCCGAGTGCCGAAAGCCGCTGTCATCAGTTGGTCCTCTTTTTTAGAGTAATTCCCAAGAATTTCATTACTCATTATTTCAATTGTA
TCAAGCCATTCTTGGCAGGATACTTTAAAATATTTCTTGAACTTATAATAGTAGGGCAGTCGGACGAGTTCCCCCTCTTTCTTTTCCCCTTTAAGCTTCGGCATTGTCC
ACTCTTTCATAGTTGGAAAAACCCTGAAGGCCAGGAATTCCTGCACTAAATCTCTAGTGCTAATATGTTCTGCAATAATTTTGAATTCGCCCAGTGCAGCCCAAGTTG
GACCGTCTACTGCCATCTTGCATCGGGGTCTTGTTTCTCCGAAGATTAGTTCAAGAGGGCTCTGAACCAGCTTCTCTTTATCTTCGTCAACTTTGACATAAAACCATT
CTGATTTCCAGCCTGCCGGCCATTTGCTTCGGTAGCTGATCACGGGAAACTTCGTGGTTTTCCGATAAGCAAAATTATAACAACCAAAATTTTCATGCAATCCATCTT
TTCTGGCCTTAGTTTGGTAATGCAGCTCATGAACTCGACAGAAGCTGCCGGCAAACGGCTCCACCGCCTGGCTTCGGAGAGCCCAGATATAAACACTGAGCCTGA
CAATCGCGTTGGGAGTCAGTTGGTGAAAATAGATACCAAATCTTTGCAGCACATCAGAGATCATCCTATTTAAGGGGAATCTCAACCCAGCTTTTAGAAAGCTCTTG
AAAATAACTATTTCATCCTTTTCTGGCTTCGGGGTAGTTTCTTCCCCCCCGAAGCGAAGCAGCTTCTTCTCATTCTCACTGAAATAGCCCGACTTCACCATTTTGGAG
AAATCAGCCTTAGAGACAGTCGACTTTCCGAAGTCCAAATGGCTGGGCTTGCTTGGAACGGCAATATGGTAATCATCATCGGAATCAGCTTCCTCAATATCTCCTTC
CTCCGCTTCGGCAGCAACTTGTTCTGTATCGGCAACAGTCTGCTCTGTCTCGGCAGTGCGGATTCCTCCCGAGGTCACCAGTCCGGATCGTTGCATCGCTTCGGAG
ATTGGAACAGTCTCCGCTCCTTCGGCTTCGCCTCCCTCACGCTCAACTCTAGCGGTAGAGCGCACTCTGGCCATTTAATTCTGAATTTCTTGGGAACTAAAAAACTT
TCTCTCCCGAAGCTTTTTCTTCTGACGAAGTAGGCTTTAAACTGGAGCTTCGTCTGATTGCGAGACTCAAGCTTCGGCTATGGTTAAAAATTTTGGCAGCAAAACA
GTGCAATAGCAATGAATGCTGTGGTAACTTCACACCTACCTGTCTGTTTATATAGCACTGTAGGTAAGAAGGTGAATCGTCAGGATTTTTGCACCAGGCAAACAGCT
GCTCACACCCACTGCGCGGTGGACCGCAAAGATCAAATAGTAACCCTACAGGGTGGGACCGCTATCGTTTCTCGACAACGAGCTCAGGGAAGGTGTTTTTGGACC
TTCGGCTTCCTAAAGCCTAAGAGACTTTTTTCACGGATCAAGCTCGTTACGAAAAACGATCTAGCACCGCGAAGGGGCTACTGTTGGGACCATGCTTCGTCGCCGA
AGGTCCTGCAGAGAGATACAGCTTCGGCTGAAGCTGCTCGTACGTGATGCCAAAGGCATCATTCATGAAGCTTTGATAATACAACATATCCGAAGGAGAAGGGAC
GAACCGACTTAAAGATAAAATGACCTTTTAGTCCATAAAGGTCTGAGTCAATGTTGTAAATTTTTATGAGGGGCATGATTGTAATTCCTCACAGGCTGTGTCTTGTGC
CTACAAATAGTGAACAGTATTCCTTTACTGTTCACGCATTCCTGCAATTATAATCGCATCACTTGGAAATTATTCTTCGCCAAGACAGAGGTACAAATGTATCTAACATT
TGAATACATCAAATTTGTTTAATATGAAAATGATGATTATTTATAATTTACTTCTTATTAATGCTTCATATCTCGAATTATTTTATACAACCTCTGGGAGTTCAATTACGAA
GATCCAACCTTCGTAATTGTTTCATCTTTGACCTTCGTCCGAAGTTCATTAAATCCGTAAGGAAATAATGCTTCAGCGGACGAAGGGCATTAACATTTAACATTTTAT
GTTGCCTTGTTCTTAATTCATAGCATTTGAGAACGAGTCCCCAACAGTCACCTCGTCTCCTTCGTGCGCGGTCTTTAGTACGTCCCAAATCTCTTTAGCACTCTTCAA
CCCTTGCACCTTATTATATTCCTCTCGACTTAGAGAGATGAGGAGTATAGTAGTTGCTTGGGAATTGAAGTGCCGGATTTGGGCGACTTCGTCCGCATCATAATTTTC
ATCCCCTACGGATGGTACCTGTGCTCCAAATGCAACAATGTCCCATATGCTTGTGTGGAGTGAGGTTAGGTGATACCCCATCATATCACTCCACCTACTATAATCTTCA

CCATTAAAGACTGGTGGTTTGCCTAATGGGACAGAAAGTAAAGGAGTATGTTTAGAGATGCGAGGGTAGCGTAGGGGGATCTTACTAAATTTCTTGCGCTCATGG
CGCTTAGAAGTGACGGACGGCGCGTCGGAGCCGGAGGTGGATGGCGACGAAGAGTCGGTCTCGTAGTAGACCACTTTCTTCATCTTCTTTTTCTTCTCGCCGCTC
CGATGCGACTTGTTGTGTGAAGGGGATCCCTTCACCTTGTTATCGGACTCCCCCGATGGAGCTTTCCCGTGGCTTGTGGCGGGCTTCTCGTCGGTCATCATCTCCTT
CTTGACGTGATCTCCCGATATCACTTCGAGCGGTTAGGCTCTAATGAAGTACCAGACTCTGATACCAATTGAAAGTCGCCTAGAGGGGGGTGAATAGGCAAATCTA
AAATTTATAAACTTTAAGCACAACTACAAGTCGGGGTTAGCGTTAGAAATAAAGTCGAGTCCGAAAGAGAGGGTGAAAACAAATAACAAGCAAATAAAGTGGATG
ACACGGTGATTTGTATTACCGGGGTTCGATTCTTGCAAATCTACTCCCCGTTGAGGTGGTCACAAAGACCGGGTCTCTTTCAACCCTTTCCCTCTCTCAAACGGTCA
CTTAGACCGAGTGAGCTTTCTCCTTAATCAAACGGGTCACTTAGACCCCTCACAAGGACCACCACAACTTGGTGTATCTTGCTTTGATTATAAGTGTCTTGAGAATA
AGAATGGGAAGAAGAAAGCACTATTGCAAAAGCCAAGCGACAAGAGCGCCAAATAACACACAGATCACTGTCTCTCTCAAGTCACTAATCACTAATGATCACTTGT
CTTAATTGTGGAACTTGGAGAGATTGGAAGCTTTGATTGTGTCTTTGAATGGATTGCTAGCTCTTGTATTGAATGTAGAGGATTGGAATGCTTGGTTCAAGTGAATG
GAGGTGGTTGGGGTTGTATTTATAGCCACCAACCACTTCCTAGCCGTTGCTCCAATTCTGCCAACCGCGGACGGTCCGCGCCCTGGTCCGGACGGTCCGCCCCTAC
ACATCAACGGCTGAAAACACAACGGTCAGCAGTAACGGCTATATCAACGGCTATTTTGCATTTAATGCGTCGTCAGATGCCAGATAAAGGCAGTCGCGGACGGTTC
GGTCGTGCACCTCGGACGGTCCGTGAGGACGCTATAATTCATTTACCGAACTCGTCACCTTCAGGGTTTTCAGTTTTTTACCGACTGAACGGTCCGCGCCTGAGG
CCGGACGGTCCGCACGTCATCTCGGACGGTGCTTGCTTTTCCTCCAGACGATCCGTAGTGTAGACTTGGATTTTTGTATTGGTTCTATCCGAGGGTTATCCTGGTGT
CGCGGACGGTGCGCCGCAAAGGCCCGGACGGTCCGCGCTTAGTCTATTTTTCCAAAAAGCTTCTCCTGTCCGGAATAATCTACGGTATTCCGGATAGTCGACTTAG
AATAGTTGTAGATGAACTTATGCACCTGTGAAATGATCAGCTAGATAAACTGGTTAGTCCACAAGGTTTGTGATGGTCGTCAAACACCAAAATCAATTATAGGTAATG
TTGAGACTATTTCCCTTTCAAATATAAACATAAAACTACTTCATCGGTTCTAAAATAGAAGTCGTTTTAGCCTTTTGACAAATTCATTCAATAATTGATATATGTGTCTAA
ATTTATTGTCATTTATTTAAATATAGACATAAAAATGAAGACCTAAAACGACTACTAATTTGGTTCATAGGGAGTAAGAGCTAAAACAAATACTATTTTGGGATGGATG
GAGTATGTATTTCTATGAATATAGTTACCTTTATTATTCTCTGTCCAAATTGAGTAAATAATTACAAATAGTTACTCTCGGCGCACCTGCATATATATTTCTCCGGTACTGC
AGTATTCTATATTTCTAAGAGTTTGGCTATGACGATTCAGGCTAGACGACGCAGCAAAACCATGTATGATGATTGTATCAATACCTATCCAAGCGGCCAAATCTCTCAA
TTTCTTTACCAACGGACTCATAAAGAAGTGGTGGTGTTACGTAGATCCAATTGACATAGTCACTTAGGTAGTGTTTAGATGGAGGGACGATGAAGGATGAAGATGC
TGGATGGGATGAGATCATTCTAACTTGACCCATGTTTGGATGAAGGTCTAGAGTCATGATCAAGGAATATTCTTCAAAAATAGTGGATGAGCCTATCCCTCGAAAAA
TAAAAGACGGGGTCAGCCCAGAGTTGATCCCGTCCCATCCCTCGTTGACACCGAACCAAACACTGCCTAAGCAGTGTCGTTGCCCTTTGATCTCACCACTTGGGTG
CTCAAGCACTGTCAGACTGATAAGGTGATTGGGCGATTGGCTCTGGTCTCTTGTCCAACGTAACTTGGCGCTACTGCGAAAGCTTGCATGCCTGCAGGTCGACTCT
AGA

Supplementary Table 3 Coding sequences of *ZmCCA1a* and *ZmCCA1b* in maize inbred lines CML288 and HZ4.

A and B: CDS of *ZmCCA1b (Zm00001d049543)* in maize inbred line CML288 and HZ4 respectively.

C and D: CDS of *ZmCCA1a (Zm00001d024546)* in maize inbred line CML288 and HZ4 respectively.

A

ATGGAGATGAATTCCTCTGGCGAGGAAACTGTGGTAAAGGTAAGAAAGCCGTACACAATAACGAAGCAGCGGGAGCGGTGGACA
GAGGCTGAACACAAACGGTTCCTTGAAGCCTTGAAACTTTATGGCAGAGCATGGCAGCGCATAGAAGAGCATGTTGGGACAAAGA
CAGCCGTGCAGATCAGAAGTCACGCTCAAAAGTTCTTCACTAAGTTGGAAAAGGAAGCTATGAACAATGGTACTTCTCCGGGGCA
AGCCCATGACATTGACATACCTCCACCACGGCCTAAAAGAAAGCCAAACAGTCCATATCCTCGAAAAAGTTGTCTCAGCTCCGAGA
CACAAACCAAAGAACTTCCAAATGACAAGTCAACAAAACCAAATATGCCCTTGAGCAATGGGCATGTAAAAATGGTAGGCGATGC
ATCTCTTCAGAATTTTCAAAGGAAGGAGTTGTCTGAAAAAGGAAGTCGCTCGGAAGTTCTTAATCTCTTCCGTGATGCCCCATCTG
CATCATTTTCTTCAGTTAACAAAAGCTCTTCAAATCATGGGGCACCCAGGAGGACCGAGGCAAGTAAAACAGAAAGCCGAGATAT
GTCCATCATGGAAAATAATTCTTTTAACCCCAACACCCAAGAGGATGTAAAGGTGATCAGTGATCAGGAAATGGAAAGGCTTAATG
GTATCCAGATCAGATCTAAATGTGAACATTCTCATGAGGGGTATTTGGACATCTCAACGCAACAAATGAAGCTAATGCCAAAGTCTG
TGGAGACAACATATGTGGATGAACAAACTGCAAGAGCTTCACACACCCTAGCAGAGAGCAACGGGACAGCTAGCGTTCCAGTGA
CTGTACCTGAAGGAACTCATCCTGATCAAACAAGTGATCAAGTGGGAATCAATGGAAGCATGAACCCATGCATCCATCCAATGGTT
TCTGCAGACCCAAAATTTGGTAGTAGTGCCACACCACAGACTTTTCCTCATAATTATGCTGCCTTTGCTCCAATGATGCAGTGCAAC
TGCAACCAAGATACGTACAGGTCATTCACCAACATGTCATCCACGTTCTCCAGCATGCTTGTTTCCACGTTGTTGTCAAACCCTGCC
ATCCATGCAGCTGCCAGGCTCGCAGCATCATACTGGCCAGCAGCTGAAGGTAACACTCCTATTGATCCGAATCAAGAAAACCCTGC
AGATGGCGTCCAAGGAAGGAACATAGGCTCTCCTCCAAGCATGGCTTCTATTGTAGCAGCTACAGTTGCTGCAGCATCTGCATGGT
GGGCAACACAAGGTCTTCTCCCTTTCTTCGCTCCACCCGTGGCTTTTCCATTTGTACCAGCTCCTAGTGCTGCCTTTCCCACAGTTG
ATGTTCCACGACCTTCAGAGAAAGACAGAGATTGCCCAGTTGAAAATGCACAGAATGAATGCCAAGAAGCTCGAAAACAGGTAC
AGTTCGAAGGTTTAAGAGTTGCTGCTTCTTCAGAGTCTGATGGGAGTGGAAAAGGCGAGGTGTCTCTCCATACGGAGTTAAAGCT
ATCTCCTGCCCAGAATGCTGATGCCACACCTACCACAGGAGCTGGCACAAATGATGCATTCAGGAATAAGAAAAAGCAGGATCGCT

CTTCATGCGGTTCTAACACACCTTCAAGTAGTGATGTAGATGCGGGCAATGTTCCTGAGGAGGACAATGCTAATGAGAAGGCGAAG
CAAGCCTCCTGCAGCAACTCTTCAGCTGGTGACACTAACCACCGCAGATTTAGAAGCAATGGAAGCACAAGTGATTCATGGAAGG
AAGTTTCCGAAGAGGGTCGTCTGGCTTTCGATGCGCTGTTCAGTAGGGAAAAGCTTCCGCAAAGCTTTTCTCCCCCACAAGCAGT
AGACTCGAAGGAGGTTGCCAAGGAGGAGGAAGATGAAGTGACCACAGTGGCAGTTGACCTCAACAAGAATGCCACAAGCATTGA
TCATGATGACCTCGACACAATGGATGAGCCCAGGGCTTCCTTTCCCAATGAATTGTCGCACCTGAAGCTGAAATCGCGCCGTACAG
GCTTCAAACCATACAAGAGATGTTCTGTGGAAGCGAAGGAAAACCGGGTGCCGACTAGCGACATGGTTGGTACCAAGAGGATTCG
TCTTGATAGCGAAGCATCCACATAA

B.
ATGGAGATGAATTCCTCTGGCGAGGAAACTGTGGTAAAGGTAAGAAAGCCGTACACAATAACGAAGCAGCGGGAGCGGTGGACA
GAGGCTGAACACAAACGGTTCCTTGAAGCCTTGAAACTTTATGGCAGAGCATGGCAGCGCATAGAAGAGCATGTTGGGACAAAGA
CGGCCGTGCAGATCAGAAGTCACGCTCAAAAGTTCTTCACTAAGTTGGAAAAGGAAGCTATGAACAATGGTACTTCTCCGGGGCA
AGCCCATGACATTGACATACCTCCACCACGGCCTAAAAGAAAGCCAAACAGTCCATATCCTCGAAAAAGTTGTCTCAGCTCCGAGA
CACAAACCAAAGAACTTCCAAATGACAAGTCAACAAAACCATATATGCCCTTGAGCAATGGGCATGTAAAAATGGTAGGCGATGC
ATCTCTTCAGAATTTTCAAAGGAAGGAGTTGTCTGAAAAAGGAAGTCACTCGGAAGTTCTTAATCTCTTCCGTGATGCCCCATCTG
CATCATTTTCTTCAGTTAACAAAAGCTCTTCAAATCATGGGGCACCCAGGAGGACCGAGGCAAGTAAAACAGAAAGCCGAGATAT
GTCCATCATGGAAAATAATTCTTTTAACCCCAACACCCAAGAGGATGTAAAGGTGATCAGTGATCAGGAAATGGAAAGGCTTAATG
GTATCCAGATCAGATCTAAATGTGAACATTCTCATGAGGGGTATTTGGACATCTCAACGCAACAAATGAAGCTAATGCCAAAGTCTG
TGGAGACAACATATGTGGATGAACAAACTGCAAGAGCTTCACACTCCCTAGCAGAGAGCGACGGGACAGCTAGCATTCCAGTGAC
TGTACCTGAAGGAACTCATCCTGATCAAACAAGTGATCAAGTGGGAATCAATGGAAGCATGAACCCATGCATCCATCCAATGGTTT
CTGCAGACCCAAAATTTGGTAGTAGTGCCACACCACAGACTTTTCCTCATAATTATGCTGCCTTTGCTCCAATGATGCAGTGCAACT
GCAACCAAGATACGTACAGGTCATTCACCAACATGTCATCCAGCTTCTCCAGCATGCTTGTTTCCACGTTGTTGTCAAACCCTGCCA
TCCATGCAGCTGCCAGGCTCGCAGCATCATACTGGCCAGCAGCTGAAGGTAACACTCCTATTGATCCGAATCAAGAAAACCCTGCA
GATGGCGTTCAAGGAAGGAACATAGGCTCTCCTCCAAGCATGGCTTCTATTGTAGCAGCTACAGTTGCTGCAGCATCTGCATGGTG
GGCAACACAAGGTCTTCTCCCTTTCTTCGCTCCACCCGTGGCTTTTCCATTTGTACCAGCTCCTAGTGCTGCCTTTCCCACAGTTGA
TGTTCCACGACCTTCAGAGAAAGACAGAGATTGCCCAGTTGAAAATGCACAGAATGAATGCCAAGAAGTTCGAAAACAGGTACA
GTTCGAAGGTTTAAGAGTTGCTGCTTCTTCAGAGTCTGATGGGAGTGGAAAAGGCGAGGTGTCTCTCCATACGGAGTTAAAGCTAT
CTCCTGTCCAGAATGCTGATGCCACACCTACCACAGGAGCTGGCACAAATGATGCATTCAGGACTAAGAAAAAGCAGGATCGCTC
TTCATGCGGTTCTAACACACCTTCAAGTAGTGATGTAGATGCGGGCAATGTTCCTGAGGAGGACAATGCTAATGAGAAGGCGAAGC
AAGCCTCCTGCAGCAACTCTTCAGCTGGTGACACTAACCACCGCAGATTTAGAAGCAATGGAAGCACAAGTGATTCATGGAAGGA
AGTTTCCGAAGAGGGTCGTCTAGCTTTCGATGCCCTGTTCAGTAGGGAAAAGCTTCCGCAAAGCTTTTCTCCCCCACAAGCAGTAG
ACTCGAAGGAGGTTGCCAAGGAGGAGGAAGATGAAGTGACCACAGTGGCAGTTGACCTCAACAAGAATGCCACAAGCATTGATC
ATGATGACCTCGACACAATGGATGAGCCCAGGGCTTCCTTTCCCAATGAATTGTCGCACCTGAAGCTGAAATCGCGCCGTACAGGC
TTCAAACCATACAAGAGATGTTCTGTGGAAGCGAAGGAAAACCGAGTGCCGGCTAGCGACATGGTTGGTACCAAGAGGATTCGTC

TTGATAGCGAAGCATCCACATAA

C.

ATGGAGGTGAATTCCTCTGGTGAGGAAACGGTGATAAAGGTAAGGAAGCCATACACAATAACCAAGCAGCGGGAGCGGTGGACA
GAGGCTGAGCACAAACGGTTCCTTGAAGCCTTGAAACTTTATGGCAGAGCATGGCAGCGCATAGAAGAGCATGTTGGGACAAAGA
CGGCCGTGCAGATCAGAAGTCACGCTCAAAAGTTCTTCACCAAGTTGGAAAAGGAAGCTATTAACAATGGTACTTCTCCGGGGCA
AGCCCATGACATTGACATACCGCCACCACGGCCTAAAAGAAAGGCTAACAGTCCATATCCTCGAAAAAGTGGTCTCAGCTCTGAG
ACACCAACCAAAGAACTTCCAAGTGACAAGTCAACAAAACCAAATATGCCCTTGAGCAATGAGAATGTACTAATGGCAGGTGATG
CATCTCTTCAGAAATTTCAAAGGAAGAAGTTGTCTGGAAAAGAAAGTTGCTCGGAAGTTCTTAATCTCTTCCGTGATGCCCCATCT
CCATCATTTTCTTCAGTTAACAAAAGCTCTTCAAATCATGGTGCACCGATTGAGGCAAGTAAAACAAAAATCCGAGATATGACCATT
ATGGAAAATAGTTCTCTTAACCCCAACATGCAAGAGGATGTAAAGGAAATCAATGATCAGGAGATGGAAAGGCTTAATGGTACCCA
AATCAGCTCTAAATGTGAACACTCTCATGAGGGATATTTGGACATCTCAATGCAACAAATGAAGCTAAAGCCAGAGTCTGTGGAGA
CAACAGATGTGGACAAACAAACCGCAAGAGCTTCACACTCCCTAGCGGAGATAACTGGGACAACTAGCATTCCGGTCACTGCAAC
TGAAGAAACTCATTCTGTTCTAACAAGTGATCAAGTGGGAATCAATGGAAGCATGAACCCATCCATCCATCCAATGTTTCCTGCAG
ATCCAAAATTTGATAGCAGTGCCACACCACAGCCTTTTCCTCATAATTATGCTGCCTTTGCTCCAATGATGCAGTGCAACTGCAACC
AAGATACCCACAGATCATTGGTCAACATGTCATCCACCTTCTCCAGCATGCTTGTTTCCACGTTGTTGTCAAACCCTGCCATCCATG
CAGCTGCCAGGCTCGCAGCATCATACTGGCCAGCAGCTGAAGGTAACACTCCGATTGATCCGAATCAAGAAAATCTTGCAGATGGT
GTTCAAGGAAGGAGCATAGGGTCTCCTCCAAGCATGGCTTCTATTGTAGCAGCTACAGTTGCTGCAGCATCTGCATGGTGGGCAAC
ACAAGGTCTTCTCCCTTTCTTCGCCCCACCCATGGCTTTTTCATTTGTGCCAGCTCCCAGTGCCGCCTTCCCCACAGTTGATGTTCC
ACGACCTTCAGAGAAAGATAGAGATTGCCCAGCTGAAAATGCACAGAAGGAATGCCAAGAAGCTCGAAAACAGGGACAGCTTGA
AGGTTTCAGAGTTGCCGCTTCTTCAGAGTATGATGGGAGTGGAAAAGGCGAGGTGTCTGTCCACACAGAGTTAAAGATATCTCCTG
TCCAGAATGCTGATGCCACGTCTGCCGCAGGAGCTGACACGACTGATGCATTCATGAATAAGAAAAAGCAGGACCGCTCTTCATGC
GGTTCTAACACACCTTCAAGTAGTGATGTAGATGTGGACAATGTCCCTGAGAAGGAGGGCAATGCTAATGAGAAGGCGAAGCAAG
CCTCCTGCAGCAACTCTTCAGCTGGTGACACTAACCACCGCAGATTTAGAAGCAGTGGAAGCACAAGTGATTCATGGAAGGAAGT
TTCCGAAGAGGGTCGTCTGGCTTTCCATGCGCTGTTCAGTAGAGAAAAGCTTCCGCAAAGCTTTTCTCCCCCACAAGCAGAAGGC
TCGAAGGAAGTTGGCAAGAAGGAGGAAGATGAAGTCACCACAGTGGCAGTTGACCTCAACAAGAGTACCACAAGCATTGATCAT
GACCTCGACACAATTGGTGAGCCAAGGGCTTCCTTTCCCAATGAACTGTCGCCCCTGAAGCTGAAATCGCGCCAAACAGGCTTCA
AACCATACAAGAGATGTTCTGTGGAAGCGAAGGAGAATAGGGTGCCGGCTAGCGACGAGGTTGGTACCAAGAGGATTCGTCTTGA

TAGTGAAGCATCAACATGA

D.

ATGGAGGTGAATTCCTCTGGTGAGGAAACGGTGATAAAGGTAAGGAAGCCATACACAATAACCAAGCAGCGGGAGCGGTGGAC
AGAGGCTGAGCACAAACGGTTCCTTGAAGCCTTGAAACTTTATGGCAGAGCATGGCAGCGCATAGAAGAGCATGTTGGGACAAA
GACGGCCGTGCAGATCAGAAGTCACGCTCAAAAGTTCTTCACCAAGTTGGAAAAGGAAGCTATTAACAATGGTACTTCTCCGGG
GCAAGCCCATGACATTGACATACCGCCACCACGGCCTAAAAGAAAGGCTAACAGTCCATATCCTCGAAAAAGTGGTCTCAGCTC
TGAGACACCAACCAAAGAACTTCCAAGTGACAAGTCAACAAAACCAAATATGCCCTTGAGCAATGAGAATGTACTAATGGCAGG
TGATGCATCTCTTCAGAAATTTCAAAGGAAGGAGTTGTCTGGAAAAGAAAGTTGCTCGGAAGTTCTTAATCTCTTCCGTGATGCC
CCATCTCCATCATTTTCTTCAGTTAACAAAAGCTCTTCAAATCATGGTGCACCGATTGAGGCAAGTAAAACAAAAATCCGAGATA
TGACCATTATGGAAAATAGTTCTCTTAACCCCAACATGCAAGAGGATGTAAAGGAAATCAATGATCAGGAGATGGAAAGGCTTA
ATGGTACCCAAATCAGCTCTAAATGTGAACACTCTCATGAGGGATATTTGGACATCTCAATGCAACAAATGAAGCTAAAGCCAG
AGTCTGTGGAGACAACATATGTGGACAAACAAACCGCAAGAGCTTCACACTCCCTAGCGGAGAGAACTGGGACAACTAGCATTC
CGGTCACTGCAACTGAAGAAACTCATTCTGTTCTAACAAGTGATCAAGTGGGAATCAATGGAAGCATGAACCCATCCATCCATCC
AATGTTTCCTGCAGATCCAAAATTTGATAGCAGTGCCACACCACAGCCTTTTCCTCATAATTATGCTGCCTTTGCTCCAATGATGC
AGTGCAACTGCAACCAAGATACCCACAGATCATTGGTCAACATGTCATCCACCTTCTCCAGCATGCTTGTTTCCACGTTGTTGTCA
AACCCTGCCATCCATGCAGCTGCCAGGCTCGCAGCATCATACTGGCCAGCAGCTGAAGGTAACACTCCGATTGATCCGAATCAA
GAAAATCTTGCAGATGGTGTTCAAGGAAGGAGCATAGGGTCTCCTCCAAGCATGGCTTCTATTGTAGCAGCTACAGTTGCTGCAG
CATCTGCATGGTGGGCAACACAAGGTCTTCTCCCTTTCTTCGCCCCACCCATGGCTTTTTCATTTGTGCCAGCTCCCAGTGCTGCCT
TCCCCACAGTTGATGTTCCACGACCTTCAGAGAAAGATAGAGATTGCCCAGCTGAAAATGCACAGAAGGAATGCCAAGAAGCTC
GAAAACAGGGACAGTTTGAAGGTTTCAGAGTTGCCGCTTCTTCAGAGTATGATGGGAGTGGAAAAGGCGAGGTGTCTGTCCACA
CAGAGTTAAAGATATCTCCTGTCCAGAATGCTGATGCCACGTCTGCCGCAGGAGCTGACACGACTGATGCATTCATGAATAAGA
AAAAGCAGGACCGCTCTTCATGCGGTTCTAACACACCTTCAAGTAGTGATGTAGATGTGGACAATGTCCCTGAGAAAGAGGGCA
ATGCTAATGAGAAGGCGAAGCAAGCCTCCTGCAGCAACTCTTCAGCTGGTGACACTAACCACCGCAGATTTAGAAGCAGTGGAA
GCACGAGTGATTCATGGAAGGAAGTTTCCGAAGAGGTTGTAATCTACCAACACTATGCCAATTCACTCTGCCCAAACCTTTGAGC
ATGCCGCACTGCCTTTCTAATTCACTTTTTTTGTTGGCAGGGTCGTCTGGCTTTCCATGCGCTGTTCAGTAGAGAAAAGCTTCCGC
AAAGCTTTTCTCCCCCACAAGCAGAAGGCTCGAAGGAAGTTGGCAAGGAGGAAGATGAAGTCACCACAGTGGCAGTTGACCTCA
ACAAGAGTACCACAAGCATTGATCATGACCTCGACACAATTGGTGAGCCAAGGGCTTCCTTTCCCAATGAACTGTCGCCCCTGAA

GCTGAAATTGCGCCGAACAGGCTTCAAACCATACAAGAGATGTTCTGTGGAAGCGAAGGAGAATAGGGTGCCGGCTAGCGACG
AGGTTGGTACCAAGAGGATTCGTCTTGATAGTGAAGCATCAACATGA

Supplementary Table 4 Primers used in this study

	Gene ID	Primer Name	Primer Sequence (5' to 3')
For cloning of *ZmCCA1b*	Zm00001d049543	ZmCCA1b-FP	ATGGAGATGAATTCCTCTGGCG
		ZmCCA1b-RP	TTATGTGGATGCTTCGCTATCAAG
For cloning of *ZmCCA1a*	Zm00001d024546	ZmCCA1a-FP	ATGGAGGTGAATTCCTCTGGTG
		ZmCCA1a-RP	TCATGTTGATGCTTCACTATCAAG
For cloning of cDNA sequences of corresponding genes to pGBKT7 or/and pGADT7 vectors	AT1G01060	pGBKT7 AtLHY-FP	ATGGCCATGGAGGCCGAATTCCCGGGAATGGATACTAATACATCTGGAGAAGAAT
		pGBKT7 AtLHY-RP	TGCGGCCGCTGCAGGTCGACGGATCCTCATGTAGAAGCTTCTCCTTCCA
	AT1G01060	pGADT7 AtLHY-FP	ATGGAGGCCAGTGAATTCCACCCGGGAATGGATACTAATACATCTGGAGAAGAAT
		pGADT7 AtLHY-RP	ATCTGCAGCTCGAGCTCGATGGATCCTCATGTAGAAGCTTCTCCTTCCA
	AT2G46830	pGBKT7 AtCCA1-FP	ATGGCCATGGAGGCCGAATTCCCGGGAATGGAGACAAATTCGTCTGGAG
		pGBKT7 AtCCA1-RP	TGCGGCCGCTGCAGGTCGACGGATCCTCATGTGGAAGCTTGAGTTTCC
	AT2G46830	pGADT7 AtCCA1-FP	ATGGAGGCCAGTGAATTCCACCCGGGAATGGAGACAAATTCGTCTGGAG
		pGADT7 AtCCA1-RP	ATCTGCAGCTCGAGCTCGATGGATCCTCATGTGGAAGCTTGAGTTTCC
	Zm00001d024546	pGBKT7 ZmCCA1a-FP	ATCTCAGAGGAGGACCTGCATATGATGGAGGTGAATTCCTCTGGTG
		pGBKT7 ZmCCA1a-RP	CAGCTCGAGCTCGATGGATCCTCATGTTGATGCTTCACTATCAAG
	Zm00001d024546	pGADT7 ZmCCA1a-FP	GTACCAGATTACGCTCATATGATGGAGGTGAATTCCTCTGGTG
		pGADT7 ZmCCA1a-RP	CAGCTCGAGCTCGATGGATCCTCATGTTGATGCTTCACTATCAAG
	Zm00001d049543	pGBKT7 ZmCCA1b-FP	GGCCAGTGAATTCCACCCGGGTATGGAGATGAATTCCTCTGGCG
		pGBKT7 ZmCCA1b-RP	CAGCTCGAGCTCGATGGATCCTTATGTGGATGCTTCGCTATCAAG
For cloning of cDNA sequences of *ZmCCA1a* to binary vector pCambia-1300-221	Zm00001d024546	ZmCCA1a-ox-FP	TTGGCGCGCCATGGAGATGAATTCCTCTGGCG
		ZmCCA1a-ox-RP	CGGGATCCTTATGTGGATGCTTCGCTATCAAG

	Gene ID	Primer Name	Primer Sequence (5' to 3')
For qRT-PCR of corresponding genes	Zm00001d024546	ZmCCA1a-RT-FP	AAAGAAAGTTGCTCGGAAGTTC
		ZmCCA1a-RT-RP	TTGTTTTACTTGCCTCAATCGG
For qRT-PCR of corresponding genes	AT2G46830	AtCCA1-RT -FP	CCGATGCATATCCCTGTGCTC
		AtCCA1-RT -RP	GTGTTCTTGCTCTCGGCTCT
	AT1G01060	AtLHY-RT -FP	TGCAGTTCCAACTCCAGCAA
		AtLHY-RT -RP	CGCTATACGACCCTCTTCGG
	AT1G22770	AtLGI-RT -FP	CTGTTTAAACTGGGAAGCTCAC
		AtGI-RT -RP	GGGACAAGGATATAGTACAGCC
	AT5G15840	AtCO-RT -FP	GGAGATAGAGTTGTTCCGCTTA
		AtCO-RT -RP	CCATGGATGAAATGTATGCGTT
	AT1G65480	AtFT-RT -FP	CTACAACTGGAACAACCTTTGG
		AtFT-RT -RP	TGACAATTGTAGAAAACTGCGG
	AF168884	18S-RT-FP	CCTGCGGCTTAATTGA
		18S-RT-RP	GTTAGCAGGCTGAGGTCTCG
	AT3G18780	ACT2-RT-FP	GAAGTACAGTGTCTGGATCGGTGGTT
		ACT2-RT-FP	ATTCCTGGACCTGCCTCATCATACTC
	AT1G29920	Cab2-RT-FP	CAAGTTTGGAGAGGCAGTTTG
		Cab2-RT-FP	ATCAAAATAACTTGTGTGGCCC
