# Supplementary material for: ZmCCA1a on Chromosome 10 of Maize Delays Flowering of Arabidopsis thaliana
Source: Front Plant Sci. 2020 Feb 20;11:78. doi: 10.3389/fpls.2020.00078 (PMC7044342; doi:10.3389/fpls.2020.00078)
Supplement: Supplementary file 2 [file DataSheet_2.pdf]

ZmCCA1a on Chromosome 10 of Maize Delays Flowering of *Arabidopsis thaliana*

Yong shi[1†], Xiyong Zhao[3†], Sha Guo[1], Shifeng Dong[1], Yanpeng Wen[1], Zanping Han[4], Weihuan Jin[2*], Yanhui Chen[1*]

[1]College of Agronomy/National Key Laboratory of Wheat and Maize Crop Science, Henan Agricultural University, Zhengzhou, Henan, China

[2]College of Life Sciences, Henan Agricultural University, Zhengzhou, Henan, China

[3]Crop Research Institute, Anhui Academy of Agricultural Sciences, Hefei, Anhui, China

[4]College of Agronomy, Henan University of Science and Technology, Luoyang, Henan, China

† These authors contributed equally to this work.

*** Correspondence:**

Yanhui Chen, Agricultural College, Henan Agricultural University, Zhengzhou, Henan, China

e-mail: chy9890@163.com

Weihuan Jin, College of Life Sciences, Henan Agricultural University, Zhengzhou, Henan, China

e-mail: Jinjinwh@163.com

Keywords: *maize, ZmCCA1,* overexpression, circadian rhythms, photoperiodic flowering

```
                         *                               *                             *
ZmCCA1a_CML288  MEVNSSGEETVIKVRKPYTITKQRERWTEAEHKRFLEALKLYGRAWQRIEEHVGTKTAVQIRSHAQKFFTKLEKEAINNG   80
ZmCCA1a_HZ4     MEVNSSGEETVIKVRKPYTITKQRERWTEAEHKRFLEALKLYGRAWQRIEEHVGTKTAVQIRSHAQKFFTKLEKEAINNG   80
Consensus       mevnssgeetvikvrkpytitkqrerwteaehkrflealklygrawqrieehvgtktavqirshaqkfftklekeainng

ZmCCA1a_CML288  TSPGQAHDIDIPPPRPKRKANSPYPRKSGLSSETPTKELPSDKSTKPNMPLSNENVLMAGDASLQKFQRKKLSGKESCSE  160
ZmCCA1a_HZ4     TSPGQAHDIDIPPPRPKRKANSPYPRKSGLSSETPTKELPSDKSTKPNMPLSNENVLMAGDASLQKFQRKELSGKESCSE  160
Consensus       tspgqahdidippprpkrkanspyprksglssetptkelpsdkstkpnmplsnenvlmagdaslqkfqrk lsgkescse

ZmCCA1a_CML288  VLNLFRDAPSPSFSSVNKSSSNHGAPIEASKTKIRDMTIMENSSLNPNMQEDVKEINDQEMERLNGTQISSKCEHSHEGY  240
ZmCCA1a_HZ4     VLNLFRDAPSPSFSSVNKSSSNHGAPIEASKTKIRDMTIMENSSLNPNMQEDVKEINDQEMERLNGTQISSKCEHSHEGY  240
Consensus       vlnlfrdapspsfssvnksssnhgapieasktkirdmtimensslnpnmqedvkeindqemerlngtqisskcehshegy

ZmCCA1a_CML288  LDISMQQMKLKPESVETTDVDKQTARASHSLAEITGTTSIPVTATEETHSVLTSDQVGINGSMNPSIHPMFPADPKFDSS  320
ZmCCA1a_HZ4     LDISMQQMKLKPESVETTYVDKQTARASHSLAERTGTTSIPVTATEETHSVLTSDQVGINGSMNPSIHPMFPADPKFDSS  320
Consensus       ldismqqmklkpesvett vdkqtarashslae tgttsipvtateethsvltsdqvgingsmnpsihpmfpadpkfdss

ZmCCA1a_CML288  ATPQPFPHNYAAFAPMMQCNCNQDTHRSLVNMSSTFSSMLVSTLLSNPAIHAAARLAASYWPAAEGNTPIDPNQENLADG  400
ZmCCA1a_HZ4     ATPQPFPHNYAAFAPMMQCNCNQDTHRSLVNMSSTFSSMLVSTLLSNPAIHAAARLAASYWPAAEGNTPIDPNQENLADG  400
Consensus       atpqpfphnyaafapmmqcncnqdthrslvnmsstfssmlvstllsnpaihaaarlaasywpaaegntpidpnqenladg

ZmCCA1a_CML288  VQGRSIGSPPSMASIVAATVAAASAWWATQGLLPFFAPPMAFSFVPAPSAAFPTVDVPRPSEKDRDCPAENAQKECQEAR  480
ZmCCA1a_HZ4     VQGRSIGSPPSMASIVAATVAAASAWWATQGLLPFFAPPMAFSFVPAPSAAFPTVDVPRPSEKDRDCPAENAQKECQEAR  480
Consensus       vqgrsigsppsmasivaatvaaasawwatqgllpffappmafsfvpapsaafptvdvprpsekdrdcpaenaqkecqear

ZmCCA1a_CML288  KQGQLEGFRVAASSEYDGSGKGEVSVHTELKISPVQNADATSAAGADTTDAFMNKKKQDRSSCGSNTPSSSDVDVDNVPE  560
ZmCCA1a_HZ4     KQGQFEGFRVAASSEYDGSGKGEVSVHTELKISPVQNADATSAAGADTTDAFMNKKKQDRSSCGSNTPSSSDVDVDNVPE  560
Consensus       kqgq egfrvaasseydgsgkgevsvhtelkispvqnadatsaagadttdafmnkkkqdrsscgsntpsssdvdvdnvpe

ZmCCA1a_CML288  KEGNANEKAKQASCSNSSAGDTNHRRFRSSGSTSDSWKEVSEE.............................GRLAFHAL  611
ZmCCA1a_HZ4     KEGNANEKAKQASCSNSSAGDTNHRRFRSSGSTSDSWKEVSEEVVIYQHYANSLCPNLACRTAFLIHFFCWQGRLAFHAL  640
Consensus       kegnanekakqascsnssagdtnhrrfrssgstsdswkevsee                             grlafhal

ZmCCA1a_CML288  FSREKLPQSFSPPQAEGSKEVGKKEEDEVTTVAVDLNKSTTSIDHDLDTIGEPRASFPNELSPLKLKSRQTGFKPYKRCS  691
ZmCCA1a_HZ4     FSREKLPQSFSPPQAEGSKEVGK.EEDEVTTVAVDLNKSTTSIDHDLDTIGEPRASFPNELSPLKLKLRRTGFKPYKRCS  719
Consensus       fsreklpqsfsppqaegskevgk eedevttvavdlnksttsidhdldtigeprasfpnelsplklk r tgfkpykrcs

ZmCCA1a_CML288  VEAKENRVPASDEVGTKRIRLDSEAS                                                        717
ZmCCA1a_HZ4     VEAKENRVPASDEVGTKRIRLDSEAS                                                        745
Consensus       veakenrvpasdevgtkrirldseas
```

Supplementary Figure 1 Sequence alignment of deduced amino acid sequence of ZmCCA1a in maize inbred lines CML288 and HZ4

Amino acid residues identical between ZmCCA1a in CML288 and HZ4 are highlighted in black. Conserved sequences of Myb domains of the *CCA1* and *CCA1-like* genes are enclosed in the red box. Arabic numerals at the right of sequences show positions of the last amino acid residues of corresponding proteins indicated at the right.

```
                                      *                    *                    *
ZmCCA1b_CML288 MEMNSSGEETVVKVRKPYTITKQRERWTEAEHKRFLEALKLYGRAWQRIEEHVGTKTAVQIRSHAQKFFTKLEKEAMNNG   80
ZmCCA1b_HZ4    MEMNSSGEETVVKVRKPYTITKQRERWTEAEHKRFLEALKLYGRAWQRIEEHVGTKTAVQIRSHAQKFFTKLEKEAMNNG   80
Consensus      memnssgeetvvkvrkpytitkqrerwteaehkrflealklygrawqrieehvgtktavqirshaqkfftklekeamnng

ZmCCA1b_CML288 TSPGQAHDIDIPPPRPKRKPNSPYPRKSCLSSETQTKELPNDKSTKPNMPLSNGHVKMVGDASLQNFQRKELSEKGSRSE  160
ZmCCA1b_HZ4    TSPGQAHDIDIPPPRPKRKPNSPYPRKSCLSSETQTKELPNDKSTKPYMPLSNGHVKMVGDASLQNFQRKELSEKGSHSE  160
Consensus      tspgqahdidipppprpkrkpnspyprksclssetqtkelpndkstkp mplsnghvkmvgdaslqnfqrkelsekgs se

ZmCCA1b_CML288 VLNLFRDAPSASFSSVNKSSSNHGAPRRTEASKTESRDMSIMENNSFNPNTQEDVKVISDQEMERLNGIQIRSKCEHSHE  240
ZmCCA1b_HZ4    VLNLFRDAPSASFSSVNKSSSNHGAPRRTEASKTESRDMSIMENNSFNPNTQEDVKVISDQEMERLNGIQIRSKCEHSHE  240
Consensus      vlnlfrdapsasfssvnksssnhgaprrteasktesrdmsimennsfnpntqedvkvisdqemerlngiqirskcehshe

ZmCCA1b_CML288 GYLDISTQQMKLMPKSVETTYVDEQTARASHTLAESNGTASVPVTVPEGTHPDQTSDQVGINGSMNPCIHPMVSADPKFG  320
ZmCCA1b_HZ4    GYLDISTQQMKLMPKSVETTYVDEQTARASHSLAESDGTASIPVTVPEGTHPDQTSDQVGINGSMNPCIHPMVSADPKFG  320
Consensus      gyldistqqmklmpksvettyvdeqtarash laes gtas pvtvpegthpdqtsdqvgingsmnpcihpmvsadpkfg

ZmCCA1b_CML288 SSATPQTFPHNYAAFAPMMQCNCNQDTYRSFTNMSSTFSSMLVSTLLSNPAIHAAARLAASYWPAAEGNTPIDPNQENPA  400
ZmCCA1b_HZ4    SSATPQTFPHNYAAFAPMMQCNCNQDTYRSFTNMSSSFSSMLVSTLLSNPAIHAAARLAASYWPAAEGNTPIDPNQENPA  400
Consensus      ssatpqtfphnyaafapmmqcncnqdtyrsftnmss fssmlvstllsnpaihaaarlaasywpaaegntpidpnqenpa

ZmCCA1b_CML288 DGVQGRNIGSPPSMASIVAATVAAASAWWATQGLLPFFAPPVAFPFVPAPSAAFPTVDVPRPSEKDRDCPVENAQNECQE  480
ZmCCA1b_HZ4    DGVQGRNIGSPPSMASIVAATVAAASAWWATQGLLPFFAPPVAFPFVPAPSAAFPTVDVPRPSEKDRDCPVENAQNECQE  480
Consensus      dgvqgrnigsppsmasivaatvaaasawwatqgllpffappvafpfvpapsaafptvdvprpsekdrdcpvenaqnecqe

ZmCCA1b_CML288 ARKQVQFEGLRVAASSESDGSGKGEVSLHTELKLSPAQNADATPTTGAGTNDAFRNKKKQDRSSCGSNTPSSSDVDAGNV  560
ZmCCA1b_HZ4    VRKQVQFEGLRVAASSESDGSGKGEVSLHTELKLSPVQNADATPTTGAGTNDAFRTKKKQDRSSCGSNTPSSSDVDAGNV  560
Consensus       rkqvqfeglrvaassesdgsgkgevslhtelklsp qnadatpttgagtndafr kkkqdrsscgsntpsssdvdagnv

ZmCCA1b_CML288 PEEDNANEKAKQASCSNSSAGDTNHRRFRSNGSTSDSWKEVSEEGRLAFDALFSREKLPQSFSPPQAVDSKEVAKEEEDE  640
ZmCCA1b_HZ4    PEEDNANEKAKQASCSNSSAGDTNHRRFRSNGSTSDSWKEVSEEGRLAFDALFSREKLPQSFSPPQAVDSKEVAKEEEDE  640
Consensus      peednanekakqascsnssagdtnhrrfrsngstsdswkevseegrlafdalfsreklpqsfsppqavdskevakeeede

ZmCCA1b_CML288 VTTVAVDLNKNATSIDHDDLDTMDEPRASFPNELSHLKLKSRRTGFKPYKRCSVEAKENRVPTSDMVGTKRIRLDSEAS  719
ZmCCA1b_HZ4    VTTVAVDLNKNATSIDHDDLDTMDEPRASFPNELSHLKLKSRRTGFKPYKRCSVEAKENRVPASDMVGTKRIRLDSEAS  719
Consensus      vttvavdlnknatsidhddldtmdeprasfpnelshlklksrrtgfkpykrcsveakenrvp sdmvgtkrirldseas
```

Supplementary Figure 2 Sequence alignment of deduced amino acid sequence of ZmCCA1b in maize inbred lines CML288 and HZ4

Amino acid residues identical between ZmCCA1b in CML288 and HZ4 are highlighted in black. Conserved sequences of Myb domains of the *CCA1* and *CCA1-like* genes are enclosed in the red box. Arabic numerals at the right of the sequences show positions of the last amino acid residues of corresponding proteins indicated at the right.

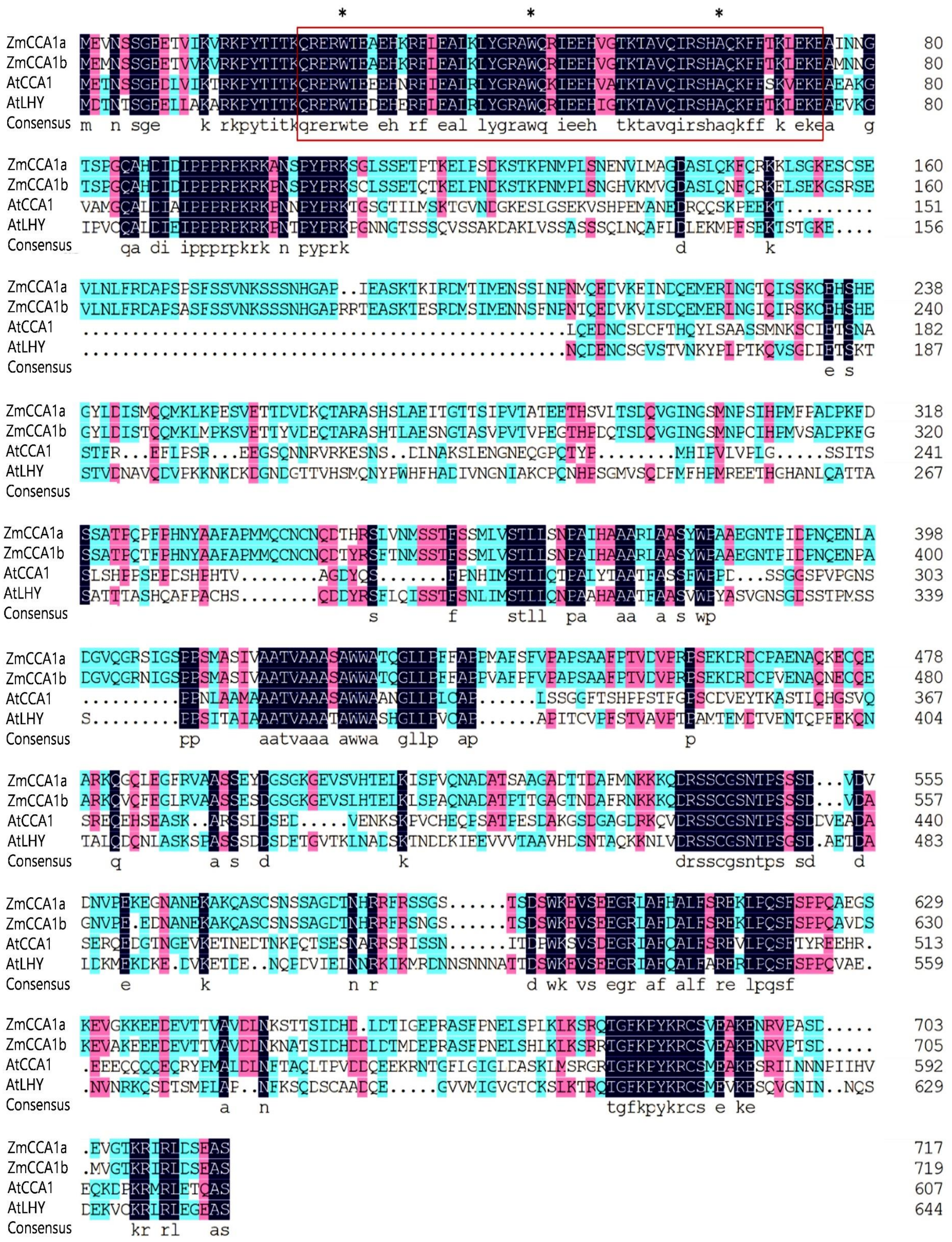

Supplementary Figure 3 Alignment of the deduced amino acid sequence of ZmCCA1a with those of ZmCCA1b and AtCCA1/AtLHY

Amino acid residues identical to those in ZmCCA1a are highlighted in black. Asterisks indicate the positions of the conserved tryptophan residues in the majority of Myb domains. Conserved sequences in the Myb domains of these proteins are enclosed in the red box. The numbers on the right indicate the positions of the last amino acid residue in each row.

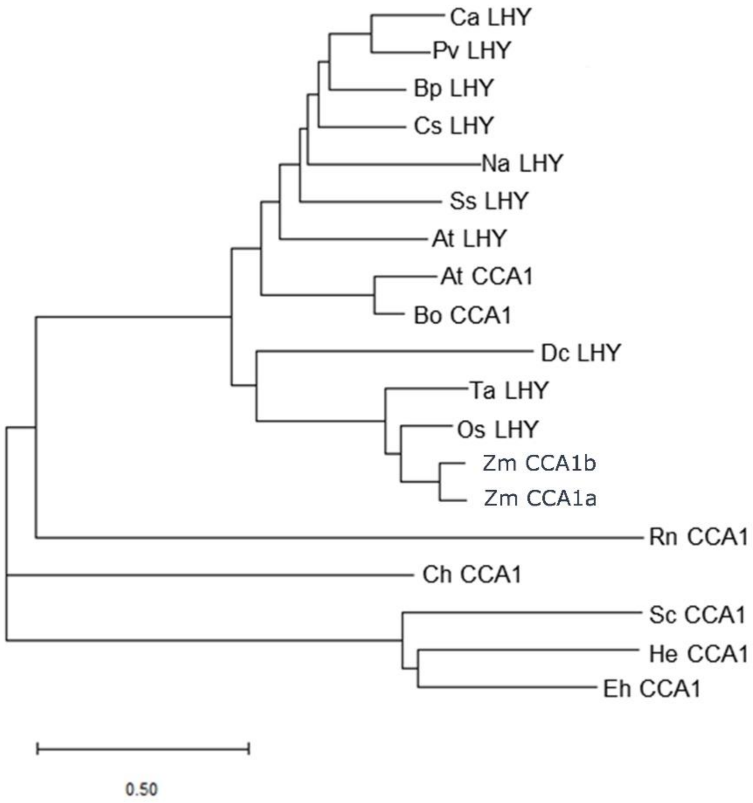

Supplementary Figure 4 Phylogenetic analysis of ZmCCA1a and ZmCCA1b proteins from different organisms

Amino acid sequences were aligned using ClustalW and the phylogenetic tree was generated by the neighbor-joining method based on 1,000 bootstrap repetitions by MEGA X (https://www.megasoftware.net/). The scale bar represents the estimated number of amino acid substitutions per site. The deduced amino acid sequences were downloaded from NCBI (http://www.ncbi.nlm.nih.gov/). Gene identifiers for the deduced amino acid sequences are: ZmCCA1b: Zm00001d049543; ZmCCA1a: Zm00001d024546; AtCCA1:819296; AtLHY: 839341; OsLHY: 4344703; CsLHY: 1187396845; BpLHY: 942430200; TaLHY: 320091615; CaLHY: 830260220; PvLHY: 21213868; SsLHYGI:1600707748; DcLHY: 1315698992; NaLHY: 1102143005; BoCCA1: 1047389776; RnCCA1GI: 2564001; ScCCA1: 1023944721; HeCCA1GI: 1178017302; EhCCA1: 1174014980; ChCCA1: 1129247100.

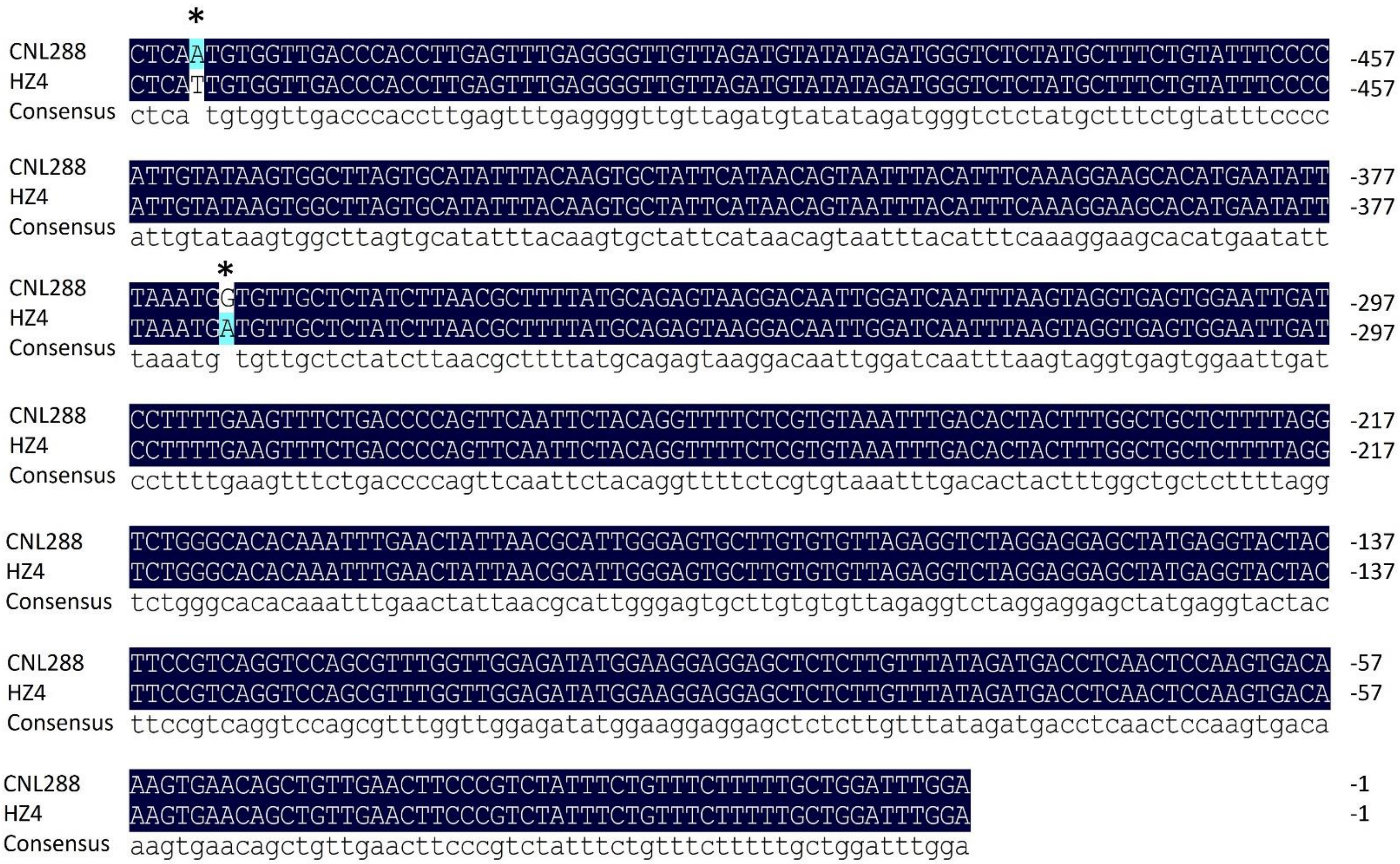

Supplementary Figure 5 Sequence alignment of *ZmCCA1a* primers in CML288 and HZ4

DNA sequences identical between *ZmCCA1a* primers in CML288 and HZ4 are highlighted in black. Asterisks indicate nucleotide diversity between the two DNA sequences. The numbers on the right indicate positions of the last nucleotide upstream of the translation initiation site ATG.

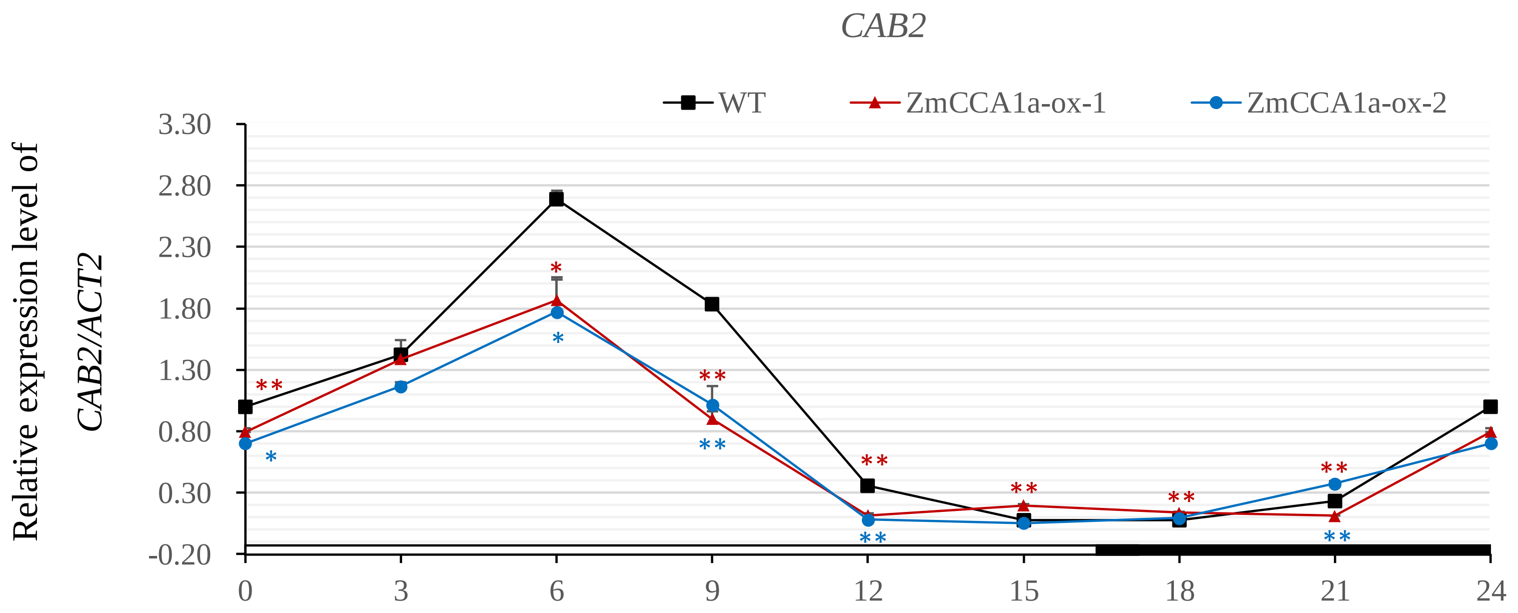

Supplementary Figure 6 Diurnal oscillations in accumulation of *CAB2* transcripts in WT and *ZmCCA1a-ox* lines

Arabidopsis seeds were grown on MS plates under long days (LD) for 11 days. Seedlings (at least 10 plants for each line) were harvested at the start of ZT0 of day 12 and thereafter at 3-h intervals for 24 h. Experiments were performed three times with similar results. Relative transcript levels were normalized to the expression level of *CAB2* in the WT at ZT0. Expression levels of *CAB2* are shown as the average of three repeats. *ZmCCA1a-ox-1* and *ZmCCA1a-ox-2* indicate two lines of transgenic *Arabidopsis*. Day and night conditions are indicated with white and black bars on the horizontal axis. Error bars indicate standard error (n=3). Student's *t* test was used to analysis the significant differences in expression levels of target genes between WT and *ZmCCA1a-ox* lines. Significance levels of $p < 0.05$ and $p < 0.01$ were marked with * and **（red for *ZmCCA1a-ox-1* and blue for *ZmCCA1a-ox-2*）, respectively.
